# Transcriptome analysis reveals the encystment-related lncRNA expression profile and coexpressed mRNAs in *Pseudourostyla cristata*

**DOI:** 10.1038/s41598-021-87680-3

**Published:** 2021-04-15

**Authors:** Nan Pan, Muhammad Zeeshan Bhatti, Wen Zhang, Bing Ni, Xinpeng Fan, Jiwu Chen

**Affiliations:** 1 School of Life Sciences, East China Normal University, Shanghai, 200241 China; 2 Shanghai Key Laboratory of Regulatory Biology, Institute of Biomedical Sciences, School of Life Sciences, East China Normal University, Shanghai, 200241 China; 3 Department of Biological Sciences, National University of Medical Sciences, Rawalpindi, 46000 Pakistan

**Keywords:** Biochemistry, Cell biology

## Abstract

Ciliated protozoans form dormant cysts for survival under adverse conditions. The molecular mechanisms regulating this process are critical for understanding how single-celled eukaryotes adapt to the environment. Despite the accumulated data on morphology and gene coding sequences, the molecular mechanism by which lncRNAs regulate ciliate encystment remains unknown. Here, we first detected and analyzed the lncRNA expression profile and coexpressed mRNAs in dormant cysts versus vegetative cells in the hypotrich ciliate *Pseudourostyla cristata* by high-throughput sequencing and qRT-PCR. A total of 853 differentially expressed lncRNAs were identified. Compared to vegetative cells, 439 and 414 lncRNAs were upregulated and downregulated, respectively, while 47 lncRNAs were specifically expressed in dormant cysts. A lncRNA-mRNA coexpression network was constructed, and the possible roles of lncRNAs were screened. Three of the identified lncRNAs, DN12058, DN20924 and DN30855, were found to play roles in fostering encystment via their coexpressed mRNAs. These lncRNAs can regulate a variety of physiological activities that are essential for encystment, including autophagy, protein degradation, the intracellular calcium concentration, microtubule-associated dynein and microtubule interactions, and cell proliferation inhibition. These findings provide the first insight into the potentially functional lncRNAs and their coexpressed mRNAs involved in the dormancy of ciliated protozoa and contribute new evidence for understanding the molecular mechanisms regulating encystment.

Ciliated protozoa are a diverse group of eukaryotes that are commonly found in different ecosystems in Earth’s biosphere^1^. They are found at various trophic levels of the food chain, which makes them important for the proper functioning of the ecosystem^2^. In addition, ciliates are single-celled organisms with highly specialized cell structures as well as a dual nature as “cells” and “animals”, which make them important models in many biological research fields, such as cytology, genetics, developmental biology and evolutionary biology^1^. Ciliates have great adaptability and various survival capabilities, and transforming into dormant cysts under adverse conditions is the one that has attracted extensive attention^3–8^. Studying the cystic state of ciliates has historically been an important aspect for exploring the relationship of structure and function and the formation of and mechanisms controlling cell patterns in eukaryotes^9^. To date, a great deal of morphological data has been accumulated on ciliate encystment at the microscopic and submicroscopic levels, and these data have shown the basic dedifferentiation processes of cellular patterns and offered new insight into ciliate phylogeny^3,10–12^. With the intensification of research, increasing studies have been performed on the gene/protein expression and biochemical changes related to cyst formation in ciliates^1^. For instance, Grisvard et al. measured differentially expressed (DE) genes during the encystment–excystment cycle of the ciliate *Sterkiella histriomuscorum* by DNA microarray analysis. The 16 most commonly observed DE genes were upregulated in cysts and excysting cells^7^. Chen et al. identified 26 DE proteins in dormant cysts of the ciliate *Euplotes encysticus* by comparative proteomic analysis. Among these DE proteins, 12 were specifically expressed proteins, and 14 were DE proteins^5^. Gao et al. found 6 specific proteins in dormant cysts of *Pseudourostyla cristata* (*P. cristata*) by using a shotgun LC–MS/MS approach^6^. More recently, two studies identified mRNA expression profiles related to the encystment of *Colpoda aspera* and *P. cristata*^4,8^. These two studies not only analyzed DE genes related to cyst formation but also identified the signaling pathways that may facilitate encystment, such as the cAMP, mTOR, PI3K/AKT, calcium and AMPK signaling pathways. The data from these molecular studies offer an in-depth view of the regulatory mechanism of cellular patterns during encystment and the function of organelles.

LncRNAs are no less than 200 nucleotides in length and lack substantial protein-coding capability. In recent years, important regulatory functions have been established and extended to study various cellular processes^4,8^. Increasing evidence has demonstrated that lncRNAs play notable roles in a broad variety of biological processes, such as the modulation of epigenetic, transcriptional or posttranscriptional gene expression and the stress response^13–15^. However, to the best of our knowledge, the role of lncRNAs in ciliate encystment has not been studied.

In the present study, *P. cristata,* which can form massive cysts in culture, was used as the experimental model to study the potential function of lncRNAs in ciliate encystment. High-throughput sequencing (HiSeq), quantitative real-time PCR (qRT-PCR) and bioinformatic techniques were used to determine the expression profiles of lncRNAs in dormant cysts versus vegetative cells, and DE lncRNAs in the encystment process were analyzed. In addition, the roles of DE lncRNAs and their coexpressed genes in the encystment mechanism were addressed. The current research work shows, for the first time, lncRNAs that regulate ciliate encystment, contributing to a better understanding of the complicated molecular mechanisms regulating encystment stress resistance in ciliates.

## Results

### Results of sequencing and characteristics of transcripts

**Figure 1:**
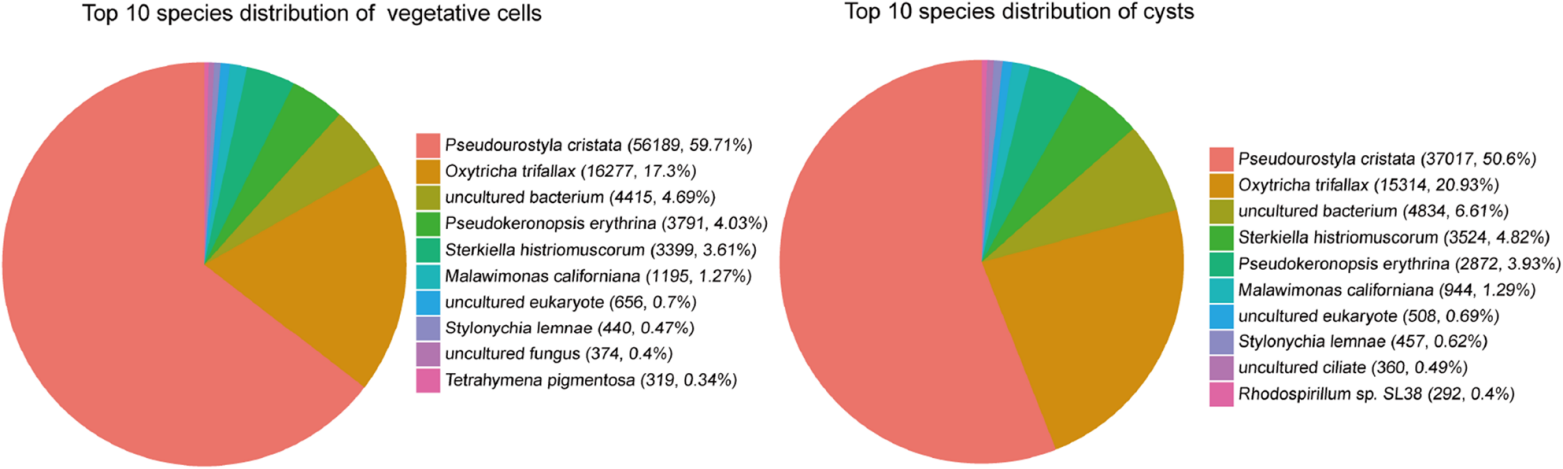
Pie chart indicating the statistics of the contamination test of the top 10 species. Vegetative cells are shown on the left, and cysts are shown on the right. This analysis confirmed that the samples were not contaminated.

The complete experimental design is shown in Supplementary Fig. S1. A total of 98,557,186 and 99,463,640 raw reads were generated from the databases of vegetative cells and dormant cysts. After removing the low-quality reads, adaptor sequences, and heterogeneous sequences and performing quality control, 96,986,214 and 97,885,228 clean reads were obtained for the vegetative cell and dormant cyst libraries, respectively (Table 1). The Q30 percentages of the reads were 95.46% in vegetative cells and 95.57% in cysts in our experiments; thus, the preprocessed read and base detection findings were reliable. Next, assessment of the read contamination indicated that the samples were not contaminated (Fig. 1).

**Table 1:** Pretreatment quality of sequencing data.

Sample	Raw_reads	Raw_bases	Clean_reads	Clean_bases	Valid_bases	Q30	GC
bn	99,463,640	14,919,546,000	97,885,228	13,975,031,425	93.67%	95.57%	37.45%
yy	98,557,186	14,783,577,900	96,986,214	13,884,155,104	93.92%	95.46%	38.36%

Dormant cysts are indicated as bn, whereas vegetative cells are indicated as yy.

**Figure 2:**
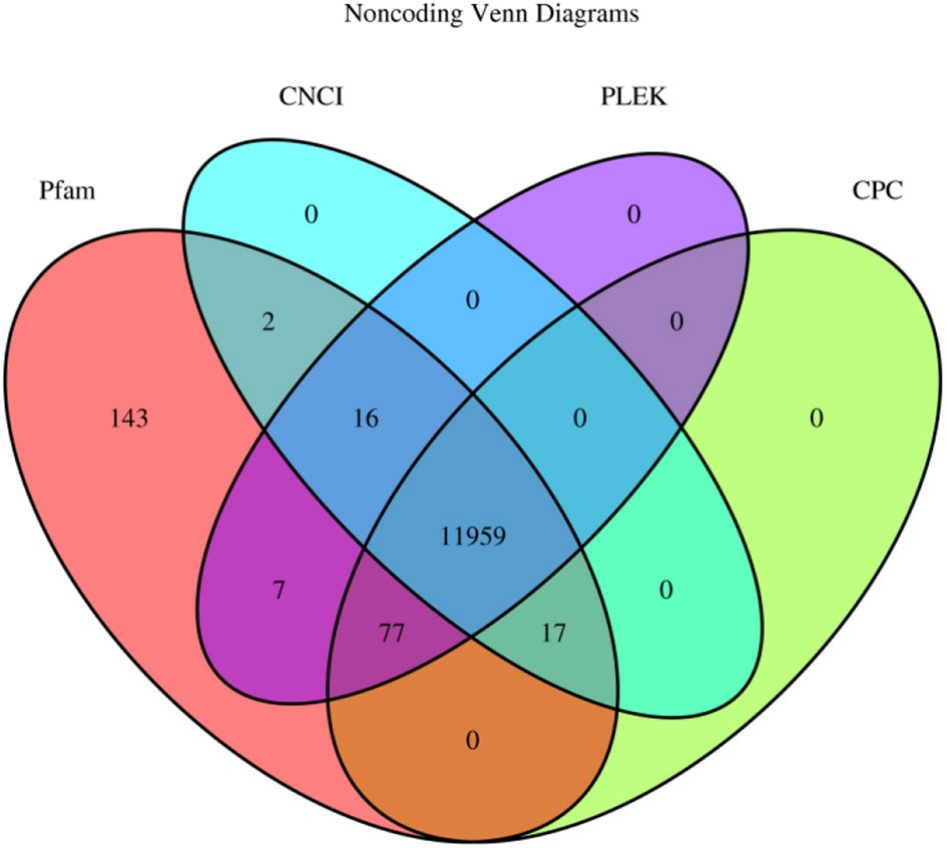
Venn diagram of lncRNA transcripts. The Venn diagram shows the numbers of filtered potential lncRNAs identified by the Coding Potential Calculator (CPC), the Coding-Non-Coding index (CNCI), the protein families database (Pfam), and predictor of lncRNAs and messenger RNAs based on an improved k-mer scheme (PLEK).

**Figure 3:**
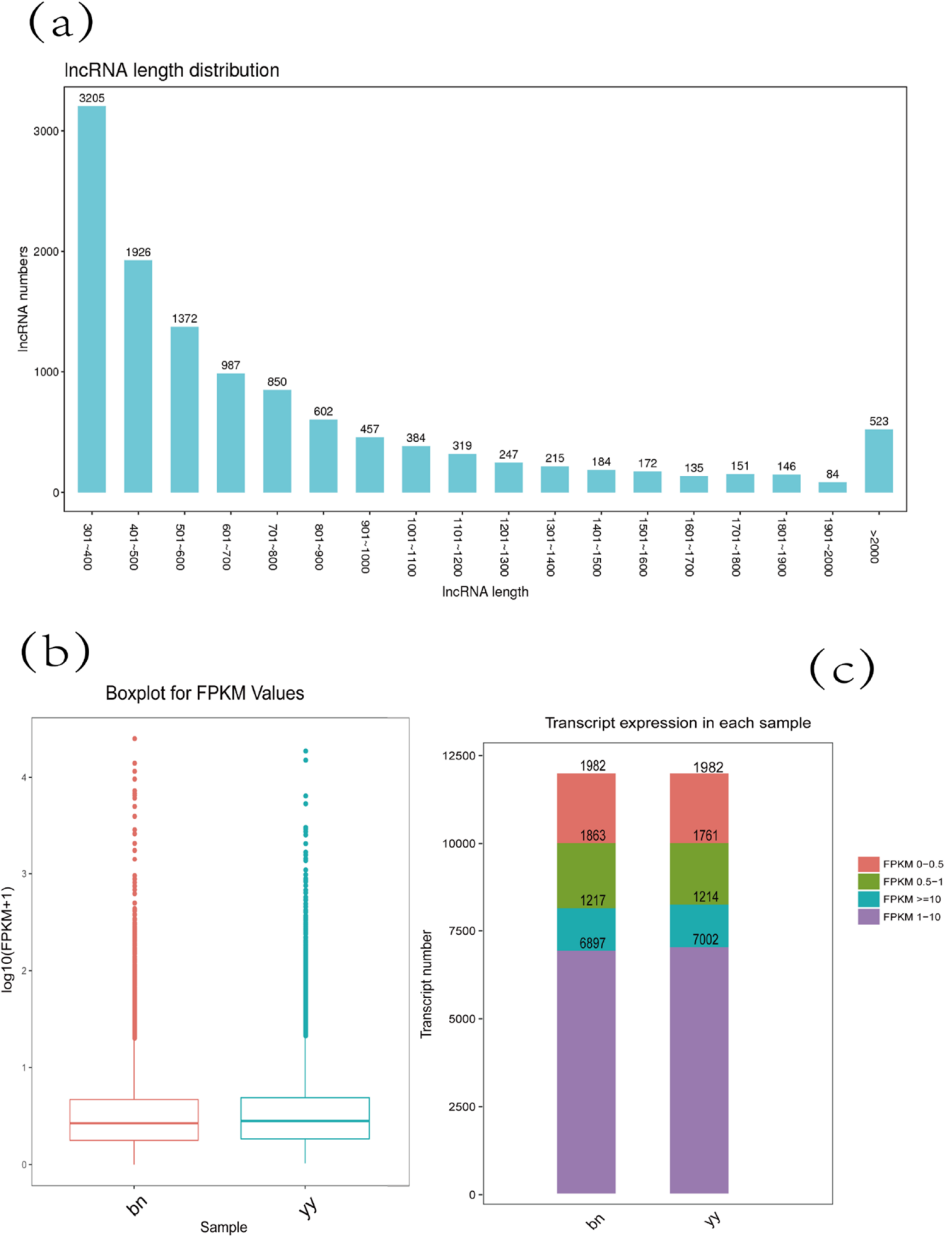
The features of lncRNA transcripts in *P. cristata*. (**a**) The length distribution of the lncRNAs. The x-axis shows the transcript length, and the y-axis shows the numbers of transcripts with different lengths. (**b**) Box plot of the transcript FPKM values in vegetative cells (yy) and cysts (bn). The y-axis shows the log10 (FPKM + 1) values. The maximum, upper quartile, median, lower quartile and minimum are indicated from top to bottom. (**c**) Bar chart showing the distribution of expression levels (FPKM values) in the samples. The different colors in the figure represent different ranges of FPKM values. The x-axis shows the sample type (vegetative cells (yy) or cysts (bn)), and the y-axis shows the number of transcripts.

To differentiate lncRNAs from protein-coding transcript units, three filtering processes mentioned in the system were used. We identified 11,959 accurately expressed lncRNA transcripts (Fig. 2). In addition, the results showed 11,959 lncRNAs, with an N50 of 919 bp and an average length of 768.49 bp. The maximum and minimum lengths of unigenes were 15,355 and 301, respectively (Table 2). Transcripts with a length of 301–400 bp accounted for the highest number of lncRNAs (3205), and transcripts containing 1901–2000 bp accounted for the lowest number (84) (Fig. 3a). We observed that the data were roughly symmetrical in the box plot of lncRNA expression levels (Fig. 3b). Due to the differences in the number of genes expressed and the distribution of gene expression values in the samples, we divided the expression values in the samples into different intervals and generated stacked histograms to display the data (Fig. 3c). Then, we found that nearly 16.57% of the transcripts had a relative expression level below 0.5 fragments per kilobase of exon per million mapped reads (FPKM); approximately 10.15% of transcripts in cysts and 10.17% in vegetative cells had a relative expression level above 10 FPKM.

**Table 2:** The statistical data for lncRNA sequencing.

Term	All (≥ 200 bp)	≥ 500 bp	≥ 1000 bp	N50	Total_Length	Max_Length	Min_Length	Average_Length
lncRNA	11,959	6842	2567	919	9,190,357	15,355	301	768.49

### Expression profiles of lncRNAs during encystment of P. cristata

A total of 853 DE lncRNAs were identified between the vegetative cell and dormant cyst libraries based on the criteria of *p* < 0.05 and |log2 FC|> 1. The IDs of lncRNAs are shown in Supplementary Table S1. Among these DE lncRNAs, 439 and 414 were upregulated and downregulated, respectively. A total of 785 of the DE lncRNAs were coexpressed in both groups. Twenty-one and 47 lncRNAs were specifically expressed in the vegetative cell and dormant cyst groups, respectively (Supplementary Fig. S2). Next, we constructed an MA map and volcano plot to further understand the overall distribution of the DE lncRNAs (Supplementary Fig. S3).

### LncRNA-mRNA coexpression network

**Figure 4:**
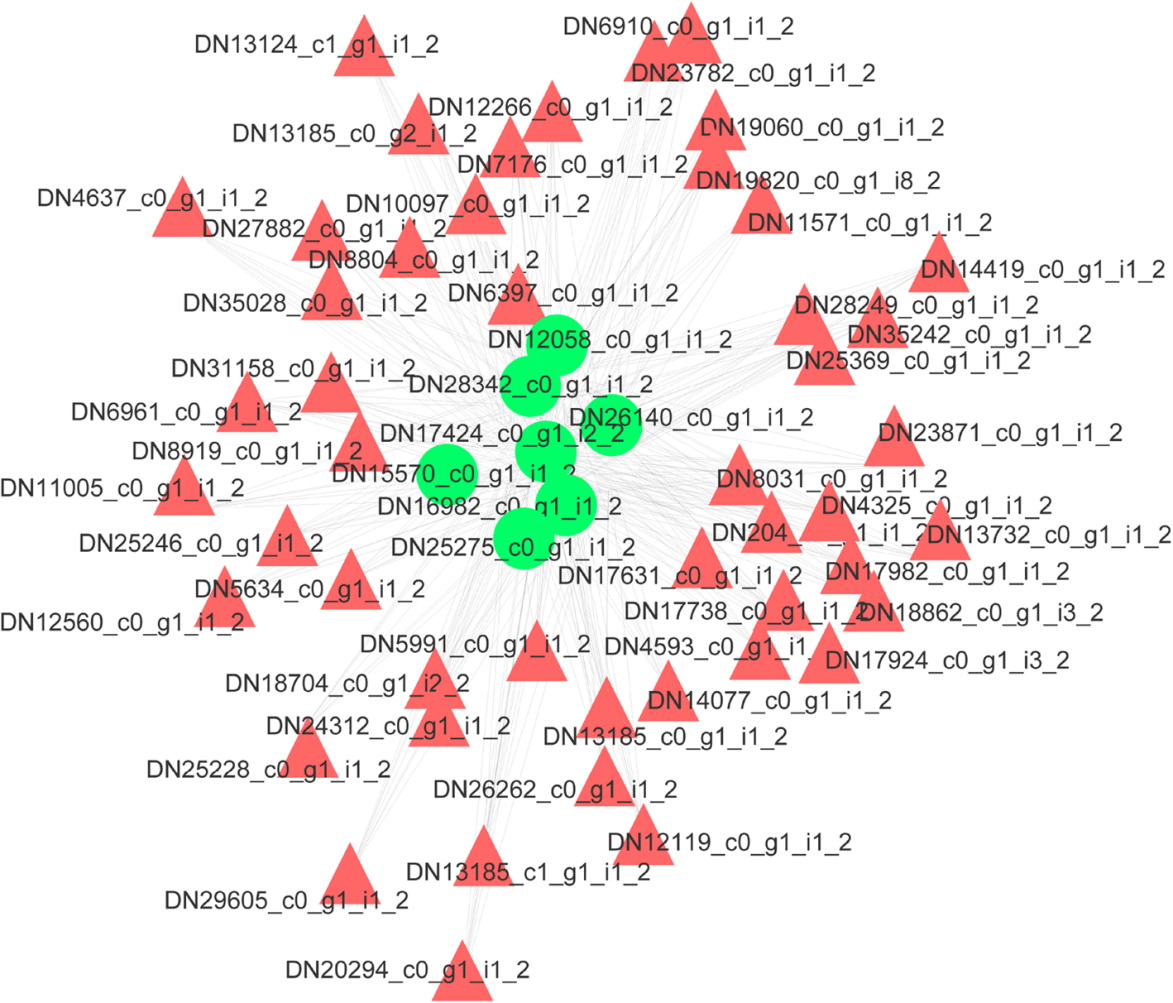
The seven network nodes with high COR values. The green circles represent lncRNAs; the red triangles represent the coexpressed mRNAs.

Most noncoding RNAs (ncRNAs) were not annotated and had an unknown function. We conducted correlation analysis between the ncRNAs and protein-coding mRNAs based on the expression of DE transcripts to predict the functions of the ncRNAs. To detect key lncRNAs and their potential functions in the encystment of *P. cristata,* we constructed a lncRNA-mRNA coexpression network and investigated the potential interactions between the lncRNA transcripts and mRNA transcripts. We constructed a coexpression network with the top 500 transcripts according to the *p*-value from low to high. There were more than 140 nodes in the network. Among these nodes, seven nodes with the highest correlation (COR) values were selected, and a coexpression network was constructed using R software (Fig. 4). The seven lncRNAs were DN12058_c0_g1_i1_2, DN25275_c0_g1_i1_2, DN28342_c0_g1_i1_2, DN16982_c0_g1_i1_2, DN15570_c0_g1_i1_2, DN17424_c0_g1_i2_2 and DN26140_c0_g1_i1_2. The mRNA transcripts coexpressed with these seven lncRNAs were involved in functions related to the ribosome and oxidative phosphorylation.

### GO and KEGG analysis of mRNAs coexpressed with lncRNAs

In order to explore the potential functional implications of the DE lncRNAs, we performed Gene Ontology (GO) and Kyoto Encyclopedia of Genes and Genomes (KEGG) functional enrichment analyses for mRNAs in the lncRNA‐associated network. We focused on three lncRNAs that were likely to be associated with cysts of *P. cristata* through bioinformatic analysis and comparative analysis of data from our previously published articles^8^. Among these three lncRNAs, the upregulated lncRNA DN12058_c0_g1_i1_2 (DN12058) had 184 coexpressed mRNAs, and the top 30 coexpressed mRNAs were identified as upregulated after screening of the COR values. The top 30 coexpressed mRNAs included *ATP1*, *ND4*, *EEF1A*, *ND5*, *hptG (HSP90 α)*, *ATP6*, *COX3*, *UBC*, *RPS13*, *petF*, *acpP*, *PABP*, *RPL7*, etc*.* (Table 3). The mRNAs coexpressed with the lncRNA DN12058 were enriched in a total of 409 GO terms. The top 30 GO terms are shown in Fig. S4. The term most enriched with the coexpressed mRNAs in the GO biological process (BP) category was autophagy. The most enriched term in the cellular component (CC) category was cytosolic small ribosomal subunit. The most enriched terms in the molecular function (MF) category was binding to mRNA. The mRNAs coexpressed with the lncRNA DN12058 were enriched in 81 signaling pathways. After screening the transcripts with an absolute fold change greater than 2, the coexpressed mRNAs were found to be significantly enriched in the proteasome, oxidative phosphorylation and ribosome pathways (Table 4).

**Table 3:** The top 30 mRNAs coexpressed with the lncRNA DN12058.

Num	Gene_id	Up_down	KEGG gene name	KEGG description	Pathway Pathway definition
1	DN13185_c0_g1_i1_2	Up	–	–	–
2	DN25246_c0_g1_i1_2	Up	ATP1	F-type H + -transporting ATPase subunit alpha	ko00190 Oxidative phosphorylation
3	DN204_c0_g1_i1_2	Up	ND4	NADH-ubiquinone oxidoreductase chain 4	ko00190 Oxidative phosphorylation
4	DN18704_c0_g1_i2_2	Up	–	–	–
5	DN17631_c0_g1_i1_2	Up	EEF1A	Elongation factor 1-alpha	ko03013 RNA transport
6	DN17738_c0_g1_i1_2	Up	–	–	–
7	DN17982_c0_g1_i1_2	Up	–	–	–
8	DN12119_c0_g1_i1_2	Up	ND5	NADH-ubiquinone oxidoreductase chain 5	ko00190 Oxidative phosphorylation
9	DN31158_c0_g1_i1_2	Up	htpG, HSP90 α	Molecular chaperone HtpG	ko04621|ko04151 NOD-like receptor signaling pathway|PI3K-Akt signaling pathway
10	DN11571_c0_g1_i1_2	Up	ATP6	F-type H + -transporting ATPase subunit a	ko00190 Oxidative phosphorylation
11	DN17924_c0_g1_i3_2	Up	–	–	–
12	DN6961_c0_g1_i1_2	Up	COX3	Cytochrome c oxidase subunit 3	ko00190 Oxidative phosphorylation
13	DN19060_c0_g1_i1_2	Up	–	–	–
14	DN26262_c0_g1_i1_2	Up	–	–	–
15	DN13185_c1_g1_i1_2	Up	–	–	–
16	DN11005_c0_g1_i1_2	Up	–	–	–
17	DN13732_c0_g1_i1_2	Up	–	–	–
18	DN18862_c0_g1_i3_2	Up	–	–	–
19	DN19820_c0_g1_i8_2	Up	UBC	Ubiquitin C	ko03320 PPAR signaling pathway
20	DN23782_c0_g1_i1_2	Up	–	–	–
21	DN23871_c0_g1_i1_2	Up	RPS13	Small subunit ribosomal protein S13e	ko03010 Ribosome
22	DN28249_c0_g1_i1_2	Up	petF	Ferredoxin	ko00195 Photosynthesis
23	DN4325_c0_g1_i1_2	Up	–	–	–
24	DN10097_c0_g1_i1_2	Up	–	–	–
25	DN12266_c0_g1_i1_2	Up	acpP	Acyl carrier protein	–
26	DN12560_c0_g1_i1_2	Up	–	–	–
27	DN14077_c0_g1_i1_2	Up	–	–	–
28	DN20294_c0_g1_i1_2	Up	PABP	Polyadenylate-binding protein	ko03018|ko03013 RNA degradation|RNA transport
29	DN24312_c0_g1_i1_2	Up	RPL7	Large subunit ribosomal protein L7e	ko03010 Ribosome
30	DN25228_c0_g1_i1_2	Up	–	–	–

**Table 4:** KEGG pathways enriched with mRNAs coexpressed with the lncRNA DN12058 after screening transcripts with an absolute fold change greater than 2.

ID	Pathway	ListHits	*p*	Enrichment_score
ko03050	Proteasome	4	0.001169779	4.972033898
ko00190	Oxidative phosphorylation	7	0.010064327	2.304916377
ko03013	RNA transport	4	0.011426821	2.924725823
ko04152	AMPK signaling pathway	3	0.059623733	2.029401591
ko04144	Endocytosis	4	0.07235503	1.783689291
ko04141	Protein processing in endoplasmic reticulum	3	0.212265617	1.264076415
ko03010	Ribosome	9	0.214253002	1.194881311

**Figure 5:**
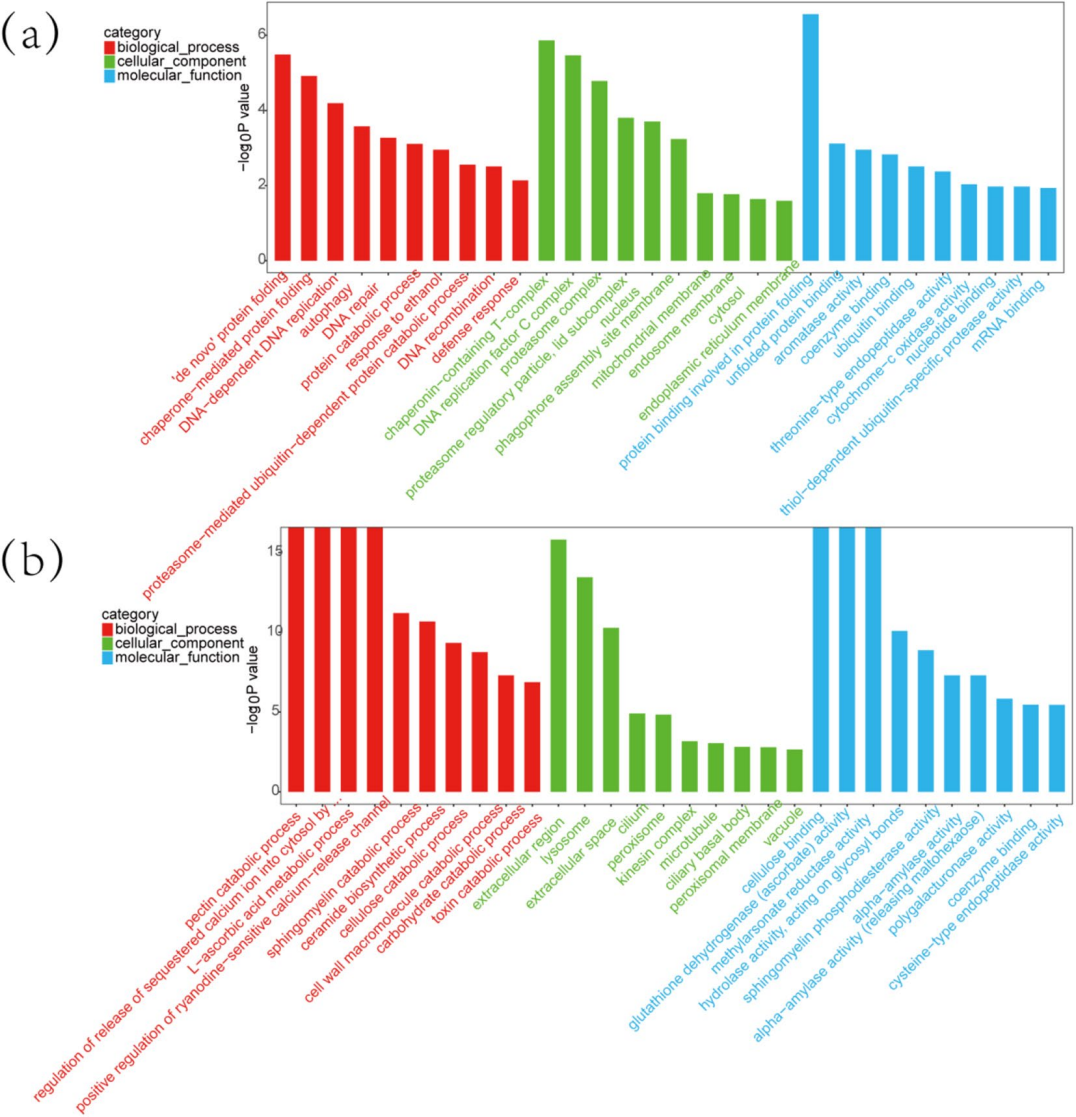
(**a**) Top 30 GO terms enriched with mRNAs coexpressed with the lncRNA DN20924. (**b**) Top 30 GO terms enriched with mRNAs coexpressed with the lncRNA DN30855. The ordinate shows the number of genes enriched in the GO term, and the abscissa shows the GO term name.

**Figure 6:**
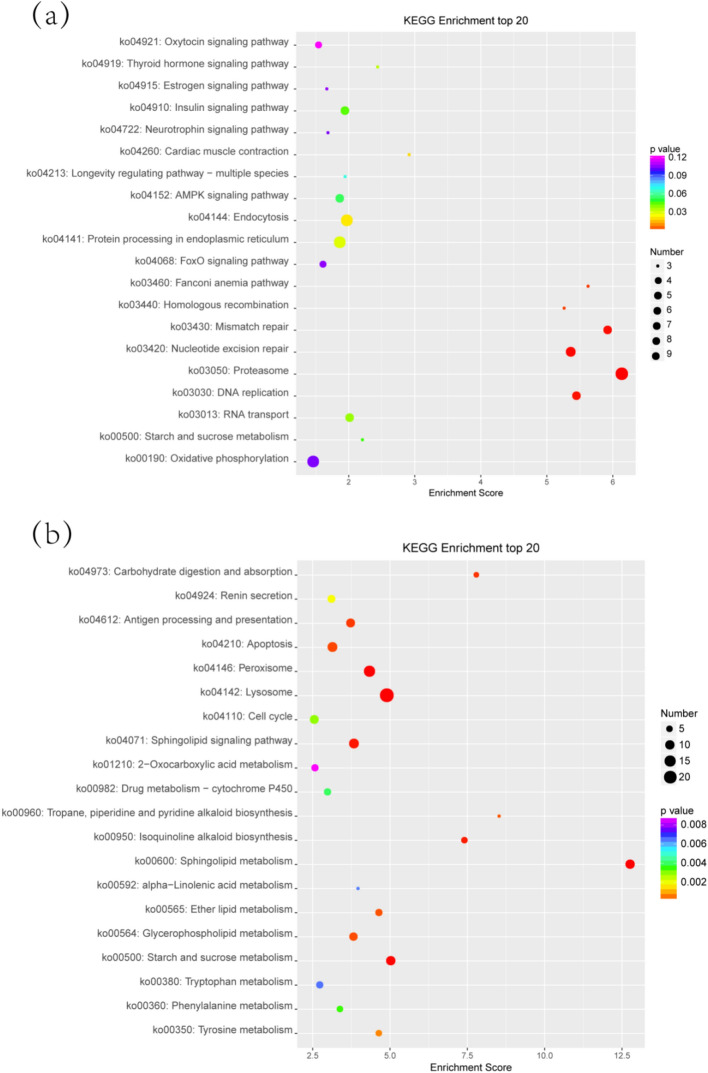
(**a**) Bubble plot of the top 20 KEGG pathways enriched with mRNAs coexpressed with the lncRNA DN20924. (**b**) Bubble plot of the top 20 KEGG pathways enriched with mRNAs coexpressed with the lncRNA DN30855. The x-axis shows the enrichment score. A larger bubble indicates a greater number of coexpressed mRNAs, and a smaller *p* value means greater significance.

In addition, we focused on the upregulated lncRNA DN20924_c0_g1_i2_2 (DN20924) and downregulated lncRNA DN30855_c0_g1_i1_1 (DN30855). the lncRNA DN20924 had 376 coexpressed mRNAs, and the top 30 coexpressed mRNAs were identified as upregulated after COR value screening. The top 30 coexpressed mRNAs included *ftnA*, *AGL*, *OSBPL5_8*, *ZIP9*, *TOP3*, *VAMP7*, *EIF1*, *UBE1*, etc*.* (Supplementary Table S2). A total of 869 GO terms were enriched with mRNAs coexpressed with the lncRNA DN20924. The top 30 GO terms are shown in Fig. 5a. The term most enriched with coexpressed mRNAs in the GO BP category was de novo protein folding. The term protein binding involved in protein folding was the most enriched term in the CC category. The most enriched term in the MF category was chaperonin-containing T-complex. The mRNAs coexpressed with the lncRNA DN20924 were enriched in 139 signaling pathways. After screening the transcripts with an absolute fold change greater than 2, the coexpressed mRNAs were found to be significantly enriched in the following pathways: proteasome, endocytosis and protein processing in endoplasmic reticulum (Fig. 6a).

The downregulated lncRNA DN30855 had 902 coexpressed mRNAs, and the top 30 coexpressed mRNAs were identified as downregulated after screening of the COR values. The top 30 coexpressed mRNAs included *GGH*, *TTLL11*, *MELK*, *amyA*, *ICK*, *ATP2B*, etc*.* (Supplementary Table S3). A total of 1287 GO terms were enriched with mRNAs coexpressed with the lncRNA DN30855. The top 30 GO terms are shown in Fig. 5b. The terms most enriched with coexpressed mRNAs in the GO BP category were pectin catabolic process, regulation of release of sequestered calcium ion into cytosol by sarcoplasmic reticulum, L-ascorbic acid metabolic process and positive regulation of ryanodine-sensitive calcium-release channel activity. The most enriched term in the CC category was extracellular region. The most enriched terms in the MF category were cellulose binding, glutathione dehydrogenase (ascorbate) activity and methylarsonate reductase activity. The mRNAs coexpressed with the lncRNA DN30855 were enriched in 150 signaling pathways. After screening the transcripts with an absolute fold change greater than 2, the coexpressed mRNAs were found to be significantly enriched in the lysosome, sphingolipid metabolism and peroxisome pathways (Fig. 6b). It was also found that the GO and KEGG annotations of the mRNAs coexpressed with the lncRNAs DN16982_c0_g1_i1_2 and DN15570_c0_g1_i1_2 were similar to those coexpressed with the lncRNA DN12058.

### Validation of lncRNA expression using qRT-PCR

**Figure 7:**
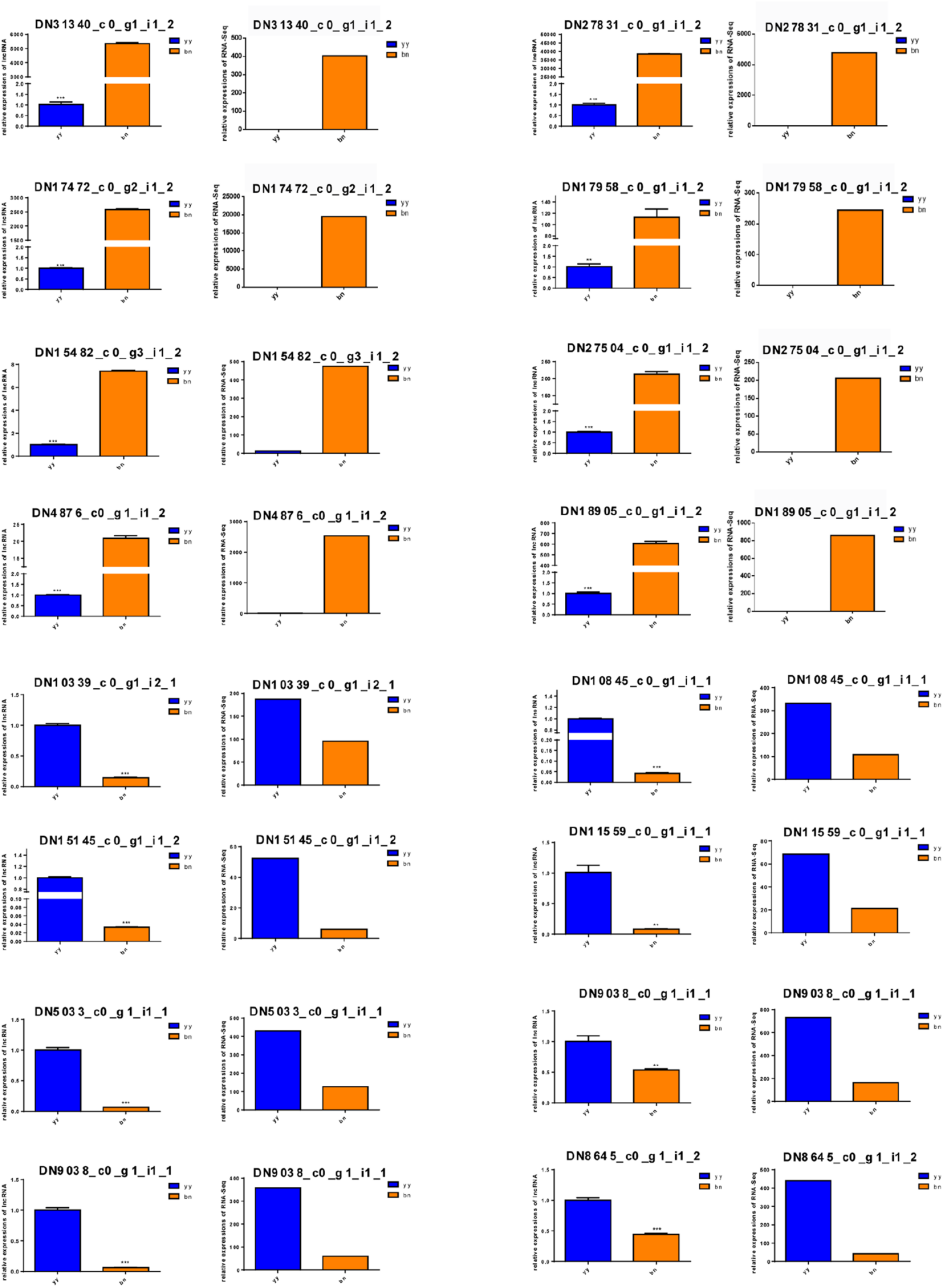
Validation of lncRNA sequencing data by qRT-PCR analysis. The left panel shows the qRT-PCR data, and the right panel represents transcriptomics data under the same lncRNA id. yy indicates vegetative cells, and bn indicates dormant cysts of *P. cristata*. Error bars represent the standard deviation of three repeats. Significant differences compared to the control group are indicated with *p < 0.05; **p < 0.01; ***p < 0.001.

To validate the HiSeq results, 16 DE lncRNAs were randomly selected for qRT-PCR analysis. As shown in Fig. 7, the expression patterns of the 16 DE lncRNAs evaluated by RNA-Seq and qRT-PCR were significantly consistent, with similar trends. Indeed, the fold changes in the expression levels of the DE lncRNAs as evaluated by qRT-PCR were not precisely the same as those shown by RNA-Seq. For example, the lncRNA DN31340 showed an increased abundance of 1304-fold in dormant cysts by RNA-Seq, whereas the observed increase in abundance in dormant cysts was determined to be 4292-fold by qRT-PCR. This difference in transcript abundance can be attributed to the differential sensitivity of RNA-Seq and qRT-PCR regarding normalization of gene expression data. These findings revealed that the detection of lncRNAs in *P. cristata* was extremely credible. In addition, correlation analysis was conducted using the correlation coefficient function in Excel to evaluate the qRT-PCR validation of lncRNA expression levels. The correlation coefficient was 0.892 (R^2^ = 0.892), further confirming the high reliability of the HiSeq results for the DE lncRNAs.

## Discussion

Research on the mechanism underlying cyst formation in ciliates has gradually advanced from the level of morphology to the level of molecular biology, but the research is still mainly focused on coding genes^4,7,8,16^. Studies on the molecular biological functions of lncRNAs in cyst formation in ciliates have not yet been reported. LncRNAs are considered to be widely distributed in the genome, to be significant regulators of gene expression, and to participate in a wide range of important cellular processes. The regulatory roles of lncRNAs have been a focal point of research in the life sciences^13–15^. In this study, for the first time, we identified DE lncRNAs between dormant cysts and vegetative cells. As discussed below, these up- or downregulated lncRNAs can engage in various encystment-related activities through their coexpressed mRNAs. Although the results are preliminary, the large number of DE lncRNAs can form the basis of further studies on the molecular mechanisms of lncRNAs in ciliate encystment.

### Regulation of lncRNAs and their coexpressed mRNAs

Among the 853 identified DE lncRNAs and their coexpressed mRNAs, we mainly focused on analyzing three DE lncRNAs and their coexpressed mRNAs. GO term analysis showed that the mRNAs coexpressed with the upregulated lncRNA DN12058 were significantly enriched in the term autophagy, and KEGG pathway analysis showed that the mRNAs coexpressed with the upregulated lncRNA DN12058 were significantly enriched in the proteasome signaling pathway. These data suggested that two pathways, *i.e.*, the ubiquitin–proteasome pathway and the autophagy pathway, were active in the degradation of proteins and organelles during cyst formation. These findings are consistent with the finding of Pan et al.^8^ that the proteasome system was activated to degrade excess proteins during *P. cristata* encystment. Furthermore, these molecular events corresponded well with dramatic changes in cell morphology, including rapid cell volume contraction, autophagy and autophagosome formation during the cyst formation process^8,17^.

Our results showed that the mRNAs coexpressed with the upregulated lncRNA DN12058 included *ATP1*, *ATP6*, *ND4*, *ND5*, *EEF1A*, *ND5*, *htpG* (*HSP90α*), *COX3*, *UBC*, *petF*, *acpP* and *PABP*.

*ATP1* and *ATP6* are ATP synthase subunits and key enzymes in mitochondrial energy metabolism in organisms, and they are involved in oxidative phosphorylation^18,19^. *COX3* is a cytochrome C oxidase (COX) subunit and plays an important role in oxidative phosphorylation through the mitochondrial respiratory chain^20^. ND4 and ND5 are NADH dehydrogenase subunits and two of the seven subunits of respiratory complex I (NADH dehydrogenase) that span the inner membrane of mitochondria. They participate in the first step of mitochondrial oxidative phosphorylation through the electron transport chain^21^. Ferredoxin (*petF*) is an electron carrier active in the electron transport chain in mitochondria^22^. It was speculated that the lncRNA DN12058 is likely to facilitate the oxidative phosphorylation process by orchestrating the upregulation of these coexpressed mRNAs and then providing the ATP necessary for the rapid morphological changes during cyst formation. HtpG, called heat shock protein-90α (Hsp90α), is a member of the HSP90 family^23^. Hsp90α is secreted by a variety of cell types in response to extracellular stress signals and plays an important role in cell repair^24^. Hsp90α also participates in a variety of key cellular reactions, including signal transduction, cell cycle modulation and transcriptional regulation^25^. These observations suggest that lncRNA DN12058 probably participates in the abovementioned functions to facilitate the development of cysts by upregulating the coexpressed HSP90α.

*EEF1A* is involved in cellular processes such as proteasomal protein degradation^26^ and cytoskeletal rearrangement^27^, which suggests that upregulated expression of *EEF1A* in tandem with lncRNA DN12058 increases protein degradation and cytoskeletal rearrangement during encystment to facilitate rapid changes in cell morphology and the formation of compact rounded cysts.

Several studies have shown that polyadenylate-binding protein (*PAB*) binds directly to the poly(A) tail of mRNA and plays a critical role in the initiation of eukaryotic translation and stabilization/degradation of mRNA^28,29^. Acyl carrier protein (*acpP*) is a fundamental component of fatty acid biosynthesis and is a cofactor in many other biochemical pathways, such as those mediating the synthesis of polyketides, phospholipids, and cell wall polysaccharides^30^. The polyubiquitin gene ubiquitin C (*UBC*) is considered to be a stress-protective gene and is upregulated under various stress conditions, which is probably a consequence of an increased demand for ubiquitin to remove toxic misfolded proteins^31^. Therefore, we speculated that the lncRNA DN12058 can increase the levels of encystment-related substances (proteins, cyst wall polysaccharides) as well as the ability of cysts to defend against stress.

Our results showed that the mRNAs coexpressed with the upregulated lncRNA DN20924 included *OSBPL5/8*, *ZIP9*, *TOP3*, *VAMP7*, *EIF1* and *UBE1*. *UBE1* is a ubiquitin-activating enzyme, and its upregulated expression was consistent with our previous findings^8^. Under adverse conditions, the expression of *UBE1* is often upregulated, and moreover, upregulated *UBE1* enhances the activity of the ubiquitin–proteasome system, which in turn degrades various abnormal and damaged proteins during cyst formation to increase the ability of cysts to adapt to adverse conditions. *VAMP7* is a v-SNARE protein in the longin family and is involved in various cell processes, including autophagy, cell membrane repair and secretion of lysosomes. It is also an important player in primary vesicle fusion^32^. Due to its different subcellular localization, *VAMP7* is involved in several membrane transport steps, including late endosome-to-lysosome fusion, Golgi-to-plasma membrane exocytosis, and lysosome-to-autophagosome vesicle transport^33–36^. Gu et al. observed that many membrane tubes were aggregated into tubular complexes in *P. cristata* cysts and speculated that their secretions are related to the formation of cyst wall precursors^8,16,37^. This observation implied that coexpression of *VAMP7* with the upregulated lncRNA DN20924 is important to promote vesicle transport of cyst wall precursor-related substances during cyst formation and to break down excess components through phagocytosis and lysosomal degradation to facilitate the formation of compact rounded cysts.

Overexpression of *OSBPL5/8* resulted in an increase in Ca^2+^ in the mitochondrial matrix during histamine stimulation and an increase in the concentration of Ca^2+^ in the endoplasmic reticulum-plasma vacuolar subregion^38^. Our previous publication also showed that an increase in the intracellular Ca^2+^ concentration can stimulate or promote *P. cristata* cyst formation^8^. Therefore, coexpression of *OSBPL5/8* with the upregulated lncRNA DN20924 probably promoted an increase in the intracellular Ca^2+^ concentration as well as Ca^2+^ signaling pathway activation and consequently stimulated cyst formation.

A large amount of evidence indicates that zinc is an essential metal that has many vital functions in eukaryotes and often exerts cell-specific effects on morphogenesis, differentiation and development. Zinc acts as a catalytic cofactor for more than 300 enzymes and also functions as an intracellular signaling molecule. Zinc deficiency can lead to various adverse effects on physiological processes. Zinc transporter member 9 (*ZIP9*) is a membrane-bound protein and an important member of the zinc transporter family that has essential roles in zinc homeostasis by regulating the transport of zinc across membranes into the cytoplasm. *ZIP9* participates in the regulation of intracellular zinc homeostasis and is involved in the processes of morphogenesis and differentiation in eukaryotes^39,40^. The coexpression of *ZIP9* with the upregulated lncRNA DN20924 may facilitate the transformation of vegetative cells to into cysts and maintain intracellular zinc homeostasis during encystment.

Recent studies have shown that DNA topoisomerases, including DNA topoisomerase III (*TOP3*), are ubiquitous enzymes that catalyze topological structural changes in DNA necessary for many steps in DNA processing, such as replication, transcription, repair and recombination. *TOP3* plays roles in maintaining genomic stability and participates in various cellular processes. *TOP3* can promote cyst formation by inducing gene expression in nonciliate protozoa^41,42^. Several studies have documented that nuclear changes, macronuclear chromatin reorganization and DNA modifications such as demethylation occur during ciliate encystment^43,44^. These results indicated that coexpression of *TOP3* with the upregulated lncRNA DN20924 stimulated cyst formation by promoting DNA recombination and transcription and inducing cyst wall protein gene expression in *P. cristata*. Upregulated expression of *TOP3* may also promote DNA repair and maintain genome stability to enhance the resistance of cysts.

It is worth noting that *EEF1A*, coexpressed with the upregulated lncRNA DN12058, and *EIF1*, coexpressed with the upregulated lncRNA DN20924, are a translation elongation factor and a translation initiation factor, respectively. Both of these factors are important players in protein synthesis. The synergistic upregulated expression of these two essential factors suggested that they stimulated the synthesis of encystment-related proteins such as cyst wall proteins and promoted encystment.

The lncRNA DN30855 and all of its coexpressed mRNAs, primarily *TTLL11*, *MELK* and *ICK*, were downregulated*.* Tubulin polyglutamylase (*TTLL*) can produce polyglutamine for an original posttranslational modification of tubulin. *TTLL* is widely found in protozoa^45^. Studies have shown that polyglutamine may play a key role in regulating microtubule structure or the interaction between microtubule-associated dynein and microtubules^46,47^. Considering that microtubules in the ciliature are absorbed during the encystment of *P. cristata*^37^, it was proposed that coexpression of *TTLL11* with the downregulated lncRNA DN30855 could result in poor microtubule transport functions and facilitate the entry of *P. cristata* into dormancy.

Maternal embryonic leucine zipper kinase (*MELK*) is a cell cycle-dependent protein kinase, and its expression is specific to proliferating cells^48^. Intestinal cell (MAK-like) kinase (*ICK*) plays a positive role in the regulation of cell proliferation and differentiation. Suppressed or downregulated expression of *ICK* can effectively inhibit cell proliferation and regulate the transition of cells from proliferation to differentiation^49^. Downregulation of the lncRNA DN3085 with coexpression of *MELK* and *ICK* may inhibit cell proliferation and in turn promote dormancy.

It is worth noting that KEGG analysis of the mRNAs coexpressed with the lncRNA DN30855 showed enrichment in signaling pathways such as lysosome and peroxisome. Downregulated expression of mRNAs in these signaling pathways results in decreased signaling pathway activity, leading to slowing of metabolism during encystment.

**Figure 8:**
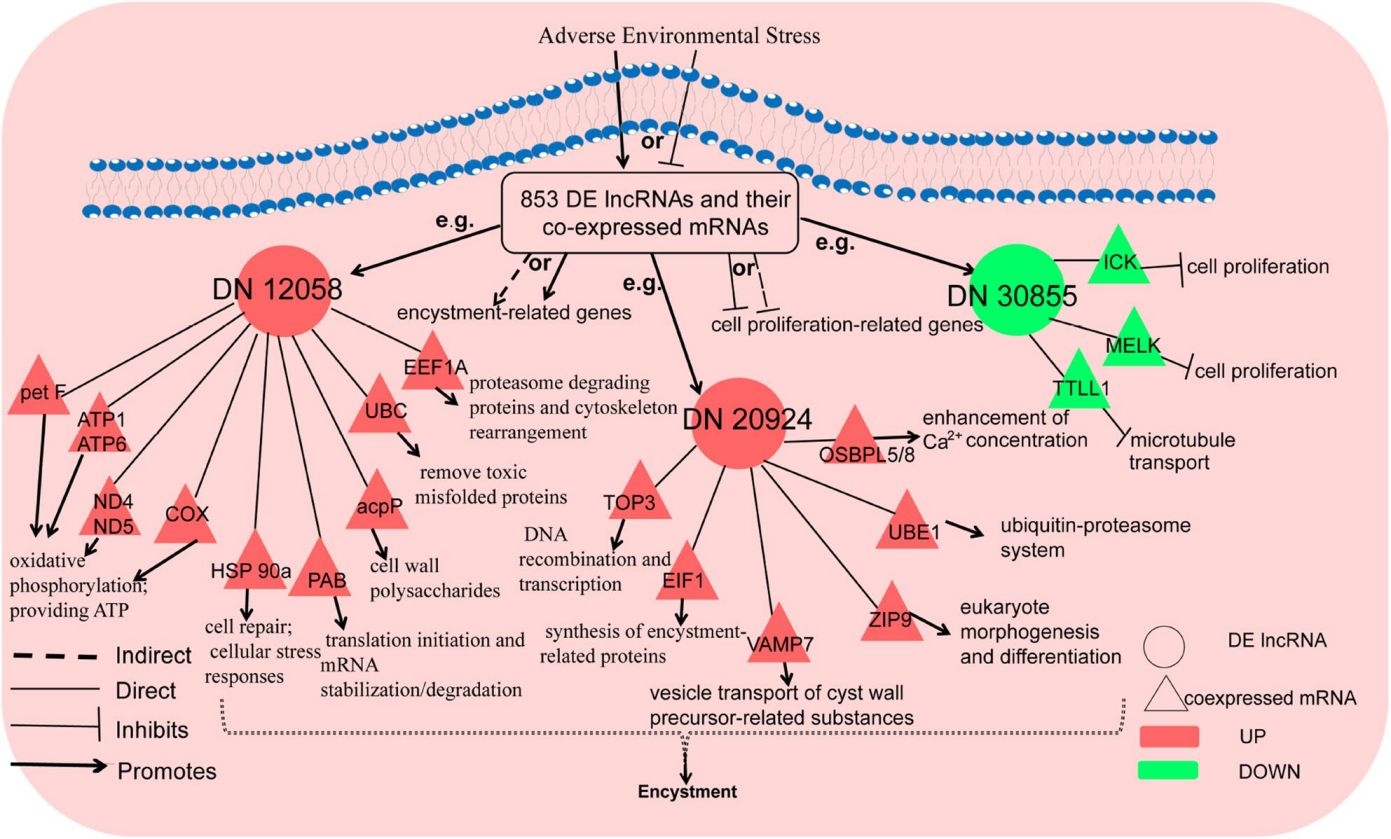
Schematic diagram of the hypothetical signaling network of the DE lncRNAs and their coexpressed mRNAs regulating encystment of *P. cristata*.

Based on the above results, we generated a schematic diagram of a proposed hypothetical signaling network of the DE lncRNAs and coexpressed mRNAs that regulate or promote *P. cristata* encystment (Fig. 8).

## Conclusions

In the current study, for the first time, we determined the lncRNA expression profiles of dormant cysts versus vegetative cells of *P. cristata* by HiSeq and revealed lncRNAs that were probably involved in the regulation of encystment. Three specific lncRNAs, DN12058, DN20924, DN30855, and their coexpressed mRNAs were studied. These lncRNAs may play roles in enhancing autophagy, protein degradation, cellular stress tolerance, synthesis of proteins required for encystment, intracellular calcium concentrations, and inhibition of cell proliferation via their coexpressed mRNAs during encystment. These three lncRNAs are likely to play synergistic regulatory roles through their coexpressed mRNAs to promote encystment. These findings provide new insights into the possible roles and detailed regulatory mechanism of lncRNAs in ciliate encystment and lead to a deeper understanding of the molecular mechanism underlying the resistance of ciliates to adverse conditions and dormancy in eukaryotes.

## Methods

### Cell culture and encystment induction

*P. cristata* (Ciliophora, Urostylida) was provided by Professor Fukang Gu from East China Normal University. *P. cristata* specimens were collected from paddy field water in southern Anhui, China, in May 2005. The ciliate cells were cultured in 10-cm Petri dishes with treated pond water and incubated at 25 °C. The pond water was filtered with filter paper produced from cotton linters and was then treated by autoclaving (LDZF-75L, Shanghai Shenan Co, Ltd., China) for 30 min at 121 °C. One or 2 boiled wheat grains were added to the culture to enrich bacteria, as the diet of ciliate cells. When the cultured vegetative cells reached a high density, the wheat grains were removed, and most of the vegetative cells formed cysts within 3–4 days. Both assays in our RNA-Seq study were performed with two biological replicates and technical replicates.

### RNA isolation, library construction, and sequencing

Approximately 2 × 10^4^ cells were collected and centrifuged at room temperature at 3000 rpm for 2 min for extraction of RNA. Total RNA was extracted using a mirVana miRNA Isolation Kit (Ambion-1561). RNA purity and quantification were evaluated using a NanoDrop 2000 spectrophotometer (Thermo Fisher Scientific, Waltham, MA, USA). RNA integrity was assessed using an Agilent 2100 Bioanalyzer (Agilent Technologies, Santa Clara, CA, USA). Quality-qualified RNA was processed with a TruSeq Stranded Total RNA with Ribo-Zero Gold Kit (Illumina, San Diego, CA, USA) to eliminate ribosomal RNA. Then, libraries were prepared using an Illumina TruSeq Stranded Total RNA LT-(with Ribo-Zero Gold)-Set A/B (Illumina, RS-122-2301/RS-122-2302, USA) according to the manufacturer’s instructions. After quantification using the Agilent 2100 bioanalyzer, the libraries were sequenced with the Illumina HiSeq X Ten platform. The raw data have been submitted to the Sequence Read Archive (SRA) (https://trace.ncbi.nlm.nih.gov/Traces/sra/sra.cgi?) database under accession numbers SRR11413602 and SRR11413601.

### Preprocessing of raw reads

To avoid data error, quality control of the raw reads was completed with Trimmomatic^50^ software by the following steps: (1) removal of adaptor sequences; (2) removal of low-quality reads; and (3) elimination of 3′ and 5′ low-quality bases via different methods. Then, the original amount of sequencing reads, effective quantity of sequencing reads, Q30 values and guanine and cytosine contents were determined and used to comprehensively evaluate the quality. Next, contamination of reads from the vegetative cells and the dormant cysts was assessed using BLAST and the NT library (fp://fp.ncbi.nih.gov/blast/db) with the criteria of an E value < 1e-10 and coverage > 80%, which confirmed that the samples were not contaminated.

### Prediction of lncRNAs

De novo transcriptome splicing was used to connect overlapping reads into longer sequences without relying on the reference genome, followed by splicing into transcripts through continuous extension. The assembled transcripts were selected for further analysis. Then, the retained transcripts were filtered according to the following process. (1) Transdecoder software was used to identify the coding region in the transcript. (2) The candidate sequence annotation information was combined to filter out the annotation sequences in the coding library. (3) The Coding Potential Calculator (CPC, version 0.9-r2, available online: http://CPC.cbi.pku.edu.cn)^51^, Coding-Non-Coding Index (CNCI, version 1.0, available online: https://github.com/www-bioinfo-org/CNCI)^52^, the protein families database (Pfam, version 30.0, available online: http://pfam.xfam.org/)^53^, and predictor of lncRNAs and messenger RNAs based on an improved k-mer scheme (PLEK, version 1.2, available online: https://sourceforge.net/projects/plek/files/)^54^ were used to filter transcripts with coding potential. Finally, the intersection of the CPC, CNCI, Pfam, and PLEK results was selected. The expression levels of the samples were calculated with the FPKM method^55^. FPKM, considers the impact of sequencing depth and unigene length on the fragment count and is currently the most commonly used expression level estimation method.

### Coexpression correlations of the de lncRNAs and mRNAs

To reveal the correlated functions of these DE lncRNAs, we performed coexpression analysis of the DE lncRNAs and their coexpressed mRNAs. The DE lncRNAs and their coexpressed mRNAs were used to construct lncRNA-mRNA pairs. The coexpressed mRNA in each lncRNA-mRNA pair can be selected to explore the main functional role of the lncRNA^56^. Therefore, by analyzing the functions of the mRNAs coexpressed with the DE lncRNAs, we can predict and judge their potential roles in cyst formation. Based on this approach, we analyzed and predicted the potential functions of the DE lncRNAs in the encystment of *P. cristata*.

To explore the functional roles of the lncRNAs, the coexpression correlations of the DE lncRNAs and DE mRNAs in vegetative cells and dormant cysts were analyzed according to the FPKM method. Then, the lncRNA-coexpressed mRNA pairs were analyzed using Pearson correlation analysis. A Pearson correlation coefficient ≥ 0.8 and *p* ≤ 0.05 were considered to indicate coregulation, and coregulated lncRNA-mRNA pairs were then screened for GO and KEGG pathway analyses.

### GO and KEGG enrichment analyses

Based on the coexpression analysis results, mRNAs coexpressed with lncRNAs were subjected to GO and KEGG enrichment analyses. LncRNA gene functions were predicted based on the GO and KEGG functional annotations of the coexpressed mRNAs. GO provides controlled and structured vocabularies that model BPs, CCs and MFs^57^. KEGG is a database containing 16 main databases roughly divided into systems information, chemical information and genomic information^58^. The most enriched GO terms might reflect potential functions of the lncRNAs. KEGG pathway analysis was also performed to understand the functions of the DE lncRNAs and their interactions with their coexpressed genes. These analyses revealed the kinds of roles that these DE lncRNAs play in the encystment of *P. cristata*.

### qRT-PCR validation of lncRNAs

To verify the reliability of the HiSeq results, we randomly selected and validated the expression of 16 lncRNAs in vegetative cells and dormant cysts by qRT-PCR analysis. Total RNA was extracted using a MiniBEST Universal RNA Extraction Kit (Takara, RR9767) according to the manufacturer’s instructions. Total RNA was transcribed into cDNA by using a PrimeScript RT Reagent Kit (Takara, RR036A) following the manufacturer’s guidelines. The primers for qRT-PCR verification were designed by Sangon Biotech (Shanghai, China). qRT-PCR analysis was performed using a TB Green Premix Ex Taq II Kit (Takara, RR820A). Briefly, the 20-μl qRT-PCR mixtures consisted of 10 μl of TB Green Premix Ex Taq II (Tli RNase H Plus), 0.8 μl of PCR forward primer (10 μM), 0.8 μl of PCR reverse primer (10 µM), 0.4 μl of ROX Reference Dye II, 2 μl of cDNA and 6 μl of nuclease-free water. The amplification reactions were incubated at 95 °C for 30 s, followed by 40 cycles at 95 °C for 5 s and 60 °C for 30 s. Glyceraldehyde-3-phosphate dehydrogenase (*GAPDH*) was used as the reference gene, and all of the primers used are listed in Supplementary Table S4. Quantification of lncRNA expression was performed using the comparative Ct method, and the specificity of the amplification products was evaluated by melting curve analysis. For each sample, reactions were performed in three independent wells. The relative gene expression levels were calculated by using the 2^−ΔΔCt^ method^59^. Student’s *t*-test was performed, and the results were considered to be significant at *p* < 0.05.

## Supplementary information

Supplementary Information

## Data Availability

Statistical analysis of the lncRNA data is described in the corresponding sections. Group comparisons were performed with independent *t*-tests. *p* < 0.05 was considered to be statistically significant. The raw Illumina sequences have been deposited in the Sequence Read Archive under accession numbers SRR11413602 and SRR11413601.

## References

[1] Xiong J (2019). Progress of protozoological studies in China: hot spots and new patterns. Scientia Sinica Vitae.

[2] Kaur H, Iqbal S, Inga E, Yawe D (2019). Encystment and excystment in ciliated protists: multidimensional approach. Curr. Sci..

[3] Martín-González A, Palacios G, Gutiérrez JC (2001). Macronuclear chromatin changes during encystment in the ciliate *Colpoda inflata*: Formation of crystal-like structures in the resting cyst chromatin and nucleolar condensation. Eur. J. Protistol..

[4] Jiang C, Wei W, Yan G, Shi T, Miao W (2019). Transcriptome analysis reveals the molecular mechanism of resting cyst formation in *Colpoda aspera*. J. Eukaryot. Microbiol..

[5] Chen J (2014). Proteomic approach to reveal the proteins associated with encystment of the ciliate *Euplotes encysticus*. PLoS ONE.

[6] Gao X, Chen F, Niu T, Qu R, Chen J (2015). Large-scale identification of encystment-related proteins and genes in *Pseudourostyla cristata*. Sci. Rep..

[7] Grisvard J, Lemullois M, Morin L, Baroin-Tourancheau A (2008). Differentially expressed genes during the encystment–excystment cycle of the ciliate *Sterkiella histriomuscorum*. Eur. J. Protistol..

[8] Pan N (2019). Novel insights into molecular mechanisms of *Pseudourostyla cristata* encystment using comparative transcriptomics. Sci. Rep..

[9] Xu R, Li J, Bai Q (2007). Advances in the studies on cysts of protozoans. Acta. Sci. Nat. Univ. Sunyatseni.

[10] Benčaťová S, Tirjaková E (2017). A study on resting cysts of an oxytrichid soil ciliate, R*igidohymena quadrinucleata* (Dragesco and Njine, 1971) Berger, 2011 (Ciliophora, Hypotrichia), including notes on its encystation and excystation process. Acta Protozool..

[11] Gutiérrez JC, Martin-González A, Callejas S (1998). Nuclear changes, macronuclear chromatin reorganization and DNA modifications during ciliate encystment. Eur. J. Protistol..

[12] Li Q, Sun Q, Fan X, Na W, Gu F (2016). The differentiation of cellular structure during encystment in the soil hypotrichous ciliate *Australocirrus* cf. *australis* (Protista, Ciliophora). Anim. Cells Syst..

[13] Qi R (2019). Comparison of LncRNA expression profiles during myogenic differentiation and adipogenic transdifferentiation of myoblasts. Int. J. Mol. Sci..

[14] Li Y (2019). Integrative analysis of the lncRNA and mRNA transcriptome revealed genes and pathways potentially involved in the anther abortion of cotton (*Gossypium hirsutum* l.). Genes.

[15] Zhou H (2019). LncRNA-cCSC1 modulates cancer stem cell properties in colorectal cancer via activation of the Hedgehog signaling pathway. J. Cell. Biochem..

[16] Zhang J, Sheng C, Tang L, Ni B, Gu F (2011). The ultrastructure of the extrusomes in *Pseudourostyla cristata*, a hypotrichous ciliated protozoan. Protoplasma.

[17] Wu, Y., Ji, L. & Gu, F. Advances in the study on resting cells in protozoa. *J. Zool*. **5**, 93–97 (2004).

[18] Hahn A (2016). Structure of a complete ATP synthase dimer reveals the molecular basis of inner mitochondrial membrane morphology. Mol. Cell..

[19] Muller K, Storchova H (2013). Transcription of atp1 is influenced by both genomic configuration and nuclear background in the highly rearranged mitochondrial genomes of *Silene vulgaris*. Plant Mol. Biol..

[20] You K, Wen J, Lee S, Kim D (2002). Cytochromec oxidase subunit III. J. Biol. Chem..

[21] Walker JE (1992). Sequences of 20 subunits of NADH: ubiquinone oxidoreductase from bovine heart mitochondria. Application of a novel strategy for sequencing proteins using the polymerase chain reaction. J. Mol. Biol..

[22] Vallieres C, Holland SL, Avery SV (2017). Mitochondrial ferredoxin determines vulnerability of cells to copper excess. Cell Chem. Biol..

[23] Prodromou C (2016). Mechanisms of Hsp90 regulation. Biochem. J..

[24] Wu R (2019). Hsp90alpha promotes the migration of iPSCs-derived keratinocyte to accelerate deep second-degree burn wound healing in mice. Biochem. Biophys. Res. Commun..

[25] Shelton LB, Koren JR, Blair LJ (2017). Imbalances in the hsp90 chaperone machinery: Implications for tauopathies. Front. Neurosci..

[26] Hamey JJ, Wilkins MR (2018). Methylation of elongation factor 1A: where, who, and why?. Trends. Biochem. Sci..

[27] Shiina N, Gotoh Y, Kubomura N, Iwamatsu A, Nishida E (1994). Microtubule severing by elongation factor 1 alpha. Science.

[28] Deo RC, Bonanno JB, Sonenberg N, Burley SK (1999). Recognition of polyadenylate RNA by the poly(A)-binding protein. Cell.

[29] Safaee N (2012). Interdomain allostery promotes assembly of the poly(A) mRNA complex with PABP and eIF4G. Mol. Cell.

[30] Jha JK (2007). Functional expression of an acyl carrier protein (ACP) from *Azospirillum brasilense* alters fatty acid profiles in *Escherichia coli* and *Brassica juncea*. Plant Physiol. Biochem..

[31] Bianchi M, Crinelli R, Arbore V, Magnani M (2018). Induction of ubiquitin C (UBC) gene transcription is mediated by HSF1: role of proteotoxic and oxidative stress. FEBS Open Bio.

[32] Daste F, Galli T, Tareste D (2015). Structure and function of longin SNAREs. J. Cell Sci..

[33] Pocard T, Le Bivic A, Galli T, Zurzolo C (2007). Distinct v-SNAREs regulate direct and indirect apical delivery in polarized epithelial cells. J. Cell Sci..

[34] Oishi Y (2006). Role of VAMP-2, VAMP-7, and VAMP-8 in constitutive exocytosis from HSY cells. Histochem. Cell Biol..

[35] Pryor PR (2004). Combinatorial SNARE complexes with VAMP7 or VAMP8 define different late endocytic fusion events. EMBO Rep..

[36] Advani RJ (1999). VAMP-7 mediates vesicular transport from endosomes to lysosomes. J. Cell Biol..

[37] Gu F, Ni B, Yang Z, Du B (2002). Ultrastructure of the vegetative and resting cyst in *Pseudourostyla cristata* (Ciliophora, Hypotrichida). Curr. Zool..

[38] Pulli I (2018). Oxysterol-binding protein related-proteins (ORPs) 5 and 8 regulate calcium signaling at specific cell compartments. Cell Calcium.

[39] Thomas P, Converse A, Berg HA (2018). ZIP9, a novel membrane androgen receptor and zinc transporter protein. Gen. Comp. Endocrinol..

[40] Huang ZL, Dufner-Beattie J, Andrews GK (2006). Expression and regulation of SLC39A family zinc transporters in the developing mouse intestine. Dev. Biol..

[41] Sun C, Weng S, Wu J (2020). DNA topoisomerase IIIβ promotes cyst generation by inducing cyst wall protein gene expression in *Giardia lamblia*. Open Biol..

[42] Hsieh MY (2014). DNA topoisomerase III alpha regulates p53-mediated tumor suppression. Clin. Cancer Res..

[43] Juan CG, Ana M, Sergio C (1998). Nuclear changes, macronuclear chromatin reorganization and DNA modifications during ciliate encystment. Eur. J. Protistol..

[44] Palacios G, Martin-Gonzalez A, Gutierrez JC (1994). Macronuclear DNA demethylation is involved in the encystment process of the ciliate *Colpoda inflata*. Cell Biol. Int..

[45] Alexander JE (1991). Characterization of posttranslational modifications in neuron-specific class III beta-tubulin by mass spectrometry. Proc. Natl. Acad. Sci..

[46] Boucher D, Larcher JC, Gros F, Denoulet P (1994). Polyglutamylation of tubulin as a progressive regulator of in vitro interactions between the microtubule-associated protein tau and tubulin. Biochemistry.

[47] Gagnon C, White D, Cosson J, Huitorel P, Cibert C (1996). The polyglutamylated lateral chain of alpha-tubulin plays a key role in flagellar motility. J. Cell Sci..

[48] Badouel C, Chartrain I, Blot J, Tassan JP (2010). Maternal embryonic leucine zipper kinase is stabilized in mitosis by phosphorylation and is partially degraded upon mitotic exit. Exp. Cell Res..

[49] Fu Z, Kim J, Vidrich A, Sturgill TW, Cohn SM (2009). Intestinal cell kinase, a MAP kinase-related kinase, regulates proliferation and G1 cell cycle progression of intestinal epithelial cells. Am. J. Physiol. Gastrointest. Liver Physiol..

[50] Bolger AM, Lohse M, Usadel B (2014). Trimmomatic: a flexible trimmer for Illumina sequence data. Bioinformatics.

[51] Kang YJ (2017). CPC2: a fast and accurate coding potential calculator based on sequence intrinsic features. Nucleic Acids Res..

[52] Sun L (2013). Utilizing sequence intrinsic composition to classify protein-coding and long non-coding transcripts. Nucleic Acids Res..

[53] Sonnhammer EL, Eddy SR, Birney E, Bateman A, Durbin R (1998). Pfam: multiple sequence alignments and HMM-profiles of protein domains. Nucleic Acids Res..

[54] Li A, Zhang J, Zhou Z (2014). PLEK: a tool for predicting long non-coding RNAs and messenger RNAs based on an improved k-mer scheme. BMC Bioinform..

[55] Mistry J, Finn RD, Eddy SR, Bateman A, Punta M (2013). Challenges in homology search: HMMER3 and convergent evolution of coiled-coil regions. Nucleic Acids Res..

[56] Kuang L (2018). Identification of long Non-coding RNAs related to skeletal muscle development in two rabbit breeds with different growth rate. Int. J. Mol. Sci..

[57] Boyle EI (2004). GO: TermFinder–open source software for accessing gene ontology information and finding significantly enriched gene ontology terms associated with a list of genes. Bioinformatics.

[58] Kanehisa M, Goto S (2000). KEGG: kyoto encyclopedia of genes and genomes. Nucleic Acids Res..

[59] Livak KJ, Schmittgen TD (2001). Analysis of relative gene expression data using real-time quantitative PCR and the 2(-Delta Delta C(T)) Method. Methods.

