# Supplementary material for: Transcriptome analysis reveals the encystment-related lncRNA expression profile and coexpressed mRNAs in Pseudourostyla cristata
Source: Sci Rep. 2021 Apr 15;11:8274. doi: 10.1038/s41598-021-87680-3 (PMC8050308; doi:10.1038/s41598-021-87680-3)
Supplement: Supplementary file 1 — Supplementary Information [file 41598_2021_87680_MOESM1_ESM.pdf]

SUPPORTING INFORMATION

Transcriptome analysis reveals the encystment-related lncRNA expression profile and coexpressed mRNAs in *Pseudourostyla cristata*

Nan Pan[1†], Muhammad Zeeshan Bhatti[2,3†], Wen Zhang[1], Bing Ni[1], Xinpeng Fan[1*] and Jiwu Chen[1*]

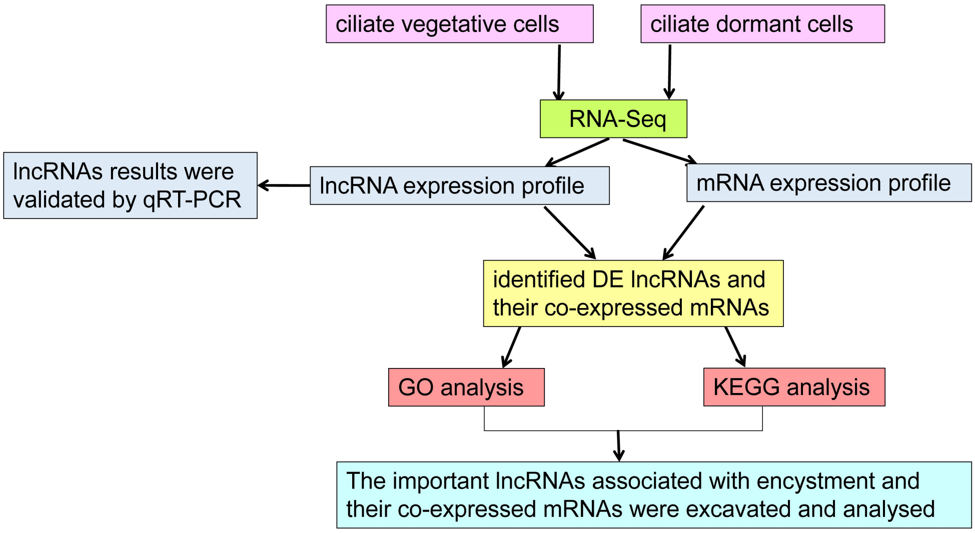

Figure S1. The total experimental design.

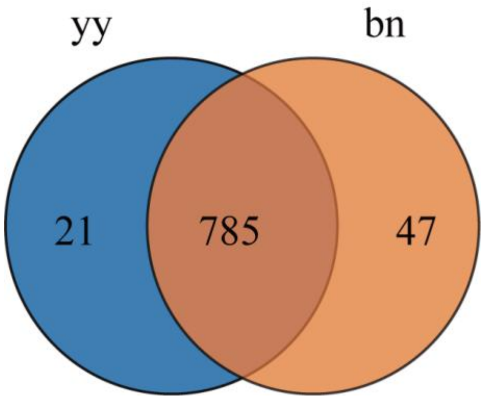

Figure S2. The venn diagram showing the similarities and differences in DE lncRNAs between vegetative cells (yy) and dormant cysts (bn) of *P. cristata*.

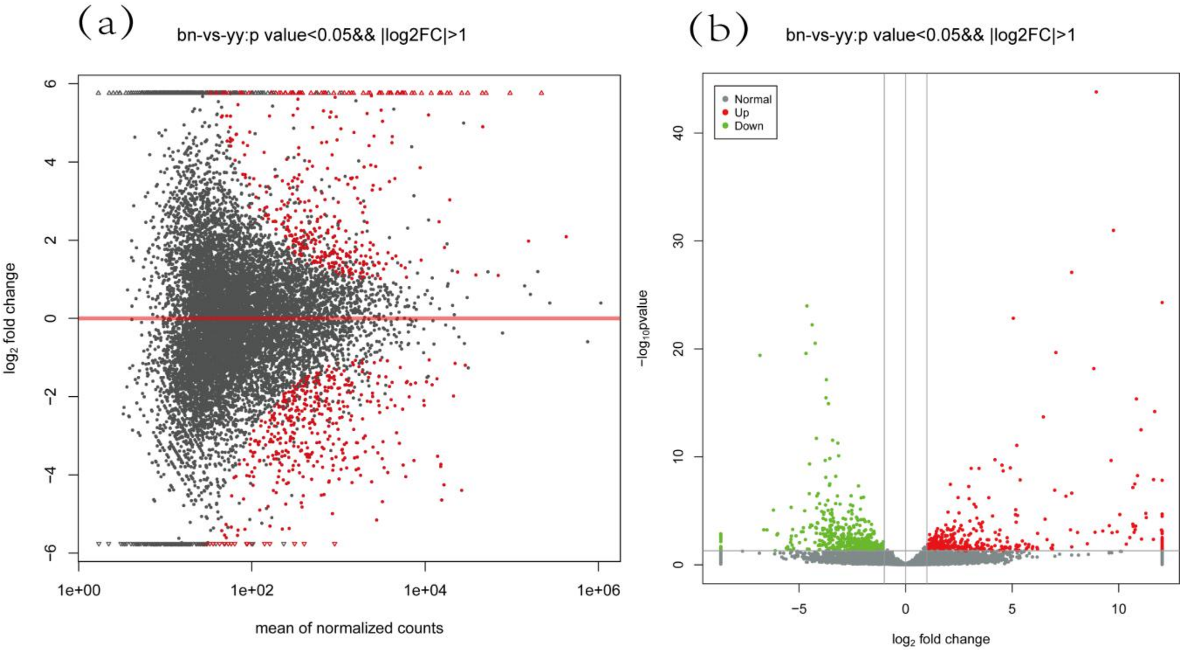

Figure S3. The MA map and volcano map of DE lncRNAs. (a) The MA map represented X-axis is the average of all sample expressions used for comparison after normalization, the Y-axis is log2 FC; The red color is labeled as the difference (depending on the difference screening conditions) DE lncRNAs. (b) The difference generated by the comparison is reflected in the volcano map, the gray color dots are

the non-differential DE lncRNAs, the red color dots are the upregulation of the significant difference unigene, the green color dots are the downregulation of the significant difference unigene; The X-axis is the display of the log2 FC, the Y-axis direction is display of log10 *p* value.

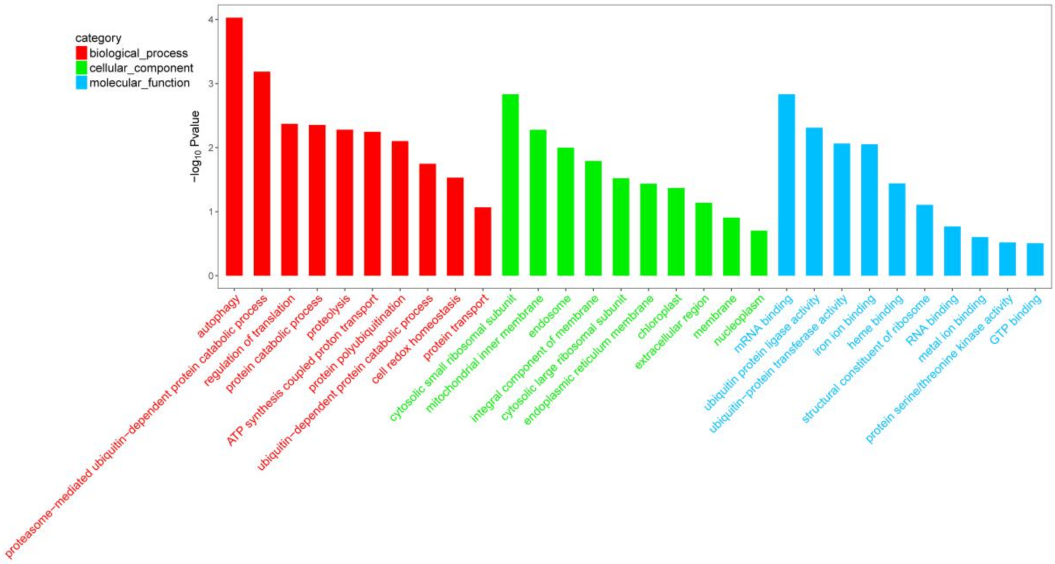

Figure S4. Top 30 GO entries map of co-expressing mRNAs with lncRNA DN12058. The ordinate represents the number of genes enriched by the GO entry, and the abscissa represents the GO entry name.

Table S1. The ids of lncRNAs

gene_id	bn	yy
DN10002_c0_g1_i1_1	1.804703	0.7947008
DN10006_c0_g1_i1_1	3.024669	3.896553
DN10006_c0_g1_i1_2	2.514154	0.4302523
DN1000_c0_g1_i1_2	0.5125491	0.3010197
DN10011_c0_g1_i1_1	0.1976822	3.71378
DN10012_c0_g1_i2_1	1.000453	4.072052
DN10013_c0_g1_i1_1	0.4540098	0.2977536
DN10013_c0_g2_i1_1	0.5466889	1.126105

DN10017_c0_g1_i1_1	3.536206	1.906137
DN10017_c0_g1_i1_2	0.2458439	4.79E-61
DN10018_c0_g1_i1_1	1.360945	1.373142
DN10019_c0_g1_i1_1	3.224977	4.440381
DN10020_c0_g1_i2_2	9.437958	11.56637
DN10022_c0_g1_i1_1	3.205251	8.878598
DN10027_c0_g1_i1_2	0.6107185	0.4217902
DN10028_c0_g1_i1_1	1.701877	11.36657
DN1002_c0_g1_i1_2	1.745012	0.3818844
DN10030_c0_g1_i1_1	0.6862685	4.703398
DN10033_c0_g1_i1_1	1.11395	5.24214
DN10034_c0_g1_i1_1	0.4817585	1.518689
DN10036_c0_g1_i1_2	0.8788006	1.656453
DN10037_c0_g1_i1_1	0.8660389	0.7100041
DN1003_c0_g1_i1_1	2.260077	1.527259
DN10040_c0_g1_i1_1	0.9163745	1.918764
DN10040_c0_g1_i1_2	1.10301	2.183874
DN10041_c0_g1_i1_2	0.5253087	0.3108586
DN10045_c1_g1_i1_1	0	1.791827
DN10047_c0_g1_i1_2	3.575915	0.989459
DN10048_c0_g1_i1_2	3.031891	1.558949
DN10051_c0_g1_i2_1	0.3302304	1.310491
DN10052_c0_g1_i1_1	0.6913024	0.3285718
DN10052_c0_g1_i2_2	7.13445	3.988401

DN10053_c0_g1_i1_1	1.835174	0.7004854
DN10053_c0_g1_i2_2	1.546022	0.2342928
DN10056_c0_g1_i1_1	0	0.848872
DN10057_c0_g1_i1_1	0.2566152	7.15E-14
DN10058_c0_g1_i1_2	7.531264	3.892498
DN10058_c0_g2_i1_1	28.09782	12.09239
DN10058_c0_g2_i1_2	10.91572	13.25205
DN10059_c0_g1_i1_2	2.382842	0.3639389
DN10060_c0_g1_i1_2	1.870426	2.534946
DN10060_c0_g2_i1_2	0.9562729	0.2642557
DN10062_c0_g1_i1_2	3.660243	1.868036
DN10064_c0_g1_i1_1	0.4138679	0.2333455
DN10064_c0_g1_i2_2	5.3641	1.326124
DN10068_c0_g1_i1_1	1.55532	0.4114866
DN10069_c0_g1_i1_1	0.5769564	1.941954
DN10069_c0_g1_i1_2	1.81302	0.2087457
DN10075_c0_g1_i1_1	0	2.053318
DN10076_c0_g2_i1_1	1.159935	3.941279
DN10077_c0_g1_i1_2	21.12076	21.1253
DN10079_c0_g1_i1_1	0.2287004	0.2083991
DN1007_c0_g1_i1_1	5.127198	3.945396
DN10083_c1_g1_i1_1	7.656196	4.892528
DN10084_c0_g1_i1_1	3.508883	6.198195
DN10088_c0_g2_i1_1	2.14269	1.09008

DN10093_c0_g1_i1_1	0.4828526	0.9067214
DN10095_c0_g1_i1_2	1.033485	1.996423
DN10096_c0_g1_i1_1	0.9774272	2.74545
DN10100_c0_g1_i1_1	2.595398	4.75383
DN10101_c0_g1_i1_2	1.848739	1.125023
DN10103_c0_g1_i1_1	1.06764	3.220373
DN10105_c0_g1_i1_2	6.062704	2.914676
DN10106_c0_g1_i1_2	2.964301	0
DN10106_c0_g1_i3_1	0.1336371	0.307448
DN10107_c0_g1_i1_1	1.152416	0.1732619
DN10111_c0_g1_i1_1	2.701914	1.336299
DN10115_c0_g2_i1_1	0.9026144	2.471689
DN10116_c0_g1_i1_1	0.7402394	0.8935033
DN10122_c0_g1_i1_2	2.950758	2.879059
DN10125_c0_g1_i1_2	5.075253	3.016957
DN10129_c1_g1_i1_1	0.7278572	1.596022
DN10130_c0_g1_i1_1	31.0705	4.174207
DN10137_c0_g1_i1_1	4.20209	15.35722
DN10143_c0_g1_i1_1	2.140826	0.4095896
DN10143_c0_g2_i1_1	0	0.3272233
DN10146_c0_g1_i1_1	22.83405	9.259015
DN10149_c0_g1_i1_1	2.320213	3.099285
DN1014_c0_g1_i2_1	1.524425	4.608072
DN10151_c0_g1_i1_2	5.623348	7.612873

DN10157_c0_g1_i1_1	3.915595	1.062002
DN10160_c0_g2_i1_1	2.181188	3.475978
DN10161_c0_g1_i1_1	1.647198	2.270547
DN10162_c0_g1_i1_1	2.861624	1.327618
DN10163_c0_g1_i1_1	0.9444536	3.005447
DN10170_c0_g1_i1_1	0.1729342	1.815868
DN10170_c0_g2_i1_1	0.3588445	1.942146
DN10172_c0_g1_i1_1	0.6703333	1.368391
DN10172_c0_g2_i1_1	0	2.273765
DN10176_c0_g1_i1_1	1.782893	24.49923
DN10176_c0_g1_i3_2	8.27925	8.069576
DN10180_c0_g1_i1_1	2.914699	10.33225
DN10186_c0_g1_i1_1	3.336681	2.296943
DN10191_c0_g1_i1_1	4.162599	2.008472
DN10196_c0_g1_i1_1	1.659664	1.068208
DN10197_c0_g1_i1_1	1.273648	0.7465177
DN10201_c0_g1_i2_1	0.980291	1.961174
DN10206_c0_g1_i1_2	2.068067	0.940724
DN10209_c0_g1_i1_1	1.003894	2.418409
DN1020_c0_g1_i1_1	0.840057	1.555136
DN10212_c0_g1_i2_1	0.9387755	1.050541
DN10214_c0_g1_i1_1	1.351341	1.131321
DN10216_c0_g1_i1_1	1.203007	1.988466
DN10217_c0_g1_i1_1	0.7082449	2.328153

DN10225_c0_g1_i1_2	0.1972718	0.1515375
DN10227_c0_g1_i1_1	0.78566	1.786603
DN10229_c0_g1_i1_1	10.33885	3.4805
DN10231_c0_g1_i1_1	2.039949	2.600915
DN10232_c0_g1_i1_1	0.1771602	1.533306
DN10233_c0_g1_i1_1	0	0.6637685
DN10237_c0_g1_i1_1	0.389463	3.93251
DN10238_c0_g1_i1_1	2.543499	1.008053
DN10242_c0_g1_i1_1	1.875285	2.141779
DN10243_c0_g1_i1_1	0.8617733	1.723192
DN10244_c0_g1_i1_1	2.542412	3.395391
DN10245_c0_g1_i1_1	1.977351	0.9159417
DN10246_c0_g1_i1_1	2.673494	3.042465
DN10249_c0_g1_i1_1	0.8129709	0.2475407
DN1024_c0_g1_i1_1	1.346582	1.123933
DN10252_c0_g1_i1_1	4.903228	2.096118
DN10253_c0_g1_i1_2	14.53351	13.95905
DN10255_c0_g1_i2_1	2.680379	5.088693
DN10256_c0_g1_i1_1	1.324237	1.373414
DN10262_c0_g1_i1_1	0.9372332	0.4732882
DN10263_c0_g1_i1_1	2.23367	2.667399
DN10267_c0_g1_i1_2	15.04453	17.16483
DN10269_c0_g1_i1_2	2.251465	0.6292664
DN1026_c0_g1_i1_1	0.9874269	2.422455

DN10271_c0_g1_i2_1	0.9786859	1.105298
DN10272_c0_g1_i1_2	0.2189432	0.2549413
DN10273_c0_g1_i1_2	1.31444	1.350493
DN10274_c0_g1_i1_1	1.441804	3.726525
DN10274_c0_g1_i1_2	1.421825	1.163741
DN10275_c0_g1_i1_1	3.900299	2.138513
DN10280_c0_g1_i1_1	3.056408	4.323327
DN10281_c0_g1_i1_1	6.027652	2.239943
DN10285_c0_g1_i2_1	3.220341	4.039556
DN10286_c0_g1_i1_1	1.552014	1.459688
DN10286_c0_g1_i1_2	0	0
DN10289_c0_g1_i1_2	4.033068	0.7870347
DN10296_c0_g1_i1_1	6.350183	3.084396
DN10297_c0_g1_i1_1	0.42254	1.792749
DN10297_c1_g1_i1_1	0.9144544	1.572986
DN1029_c0_g1_i1_1	1.722784	2.211186
DN1029_c0_g1_i1_2	4.213977	2.51932
DN10300_c0_g1_i1_1	1.904421	0.7230975
DN10301_c0_g1_i1_1	2.455897	3.219393
DN10305_c0_g1_i2_1	7.435403	7.386513
DN10306_c0_g1_i1_1	29.58992	11.75497
DN10308_c0_g2_i1_1	0	0.5267913
DN10309_c0_g1_i1_2	3.278867	0.7893208
DN10311_c0_g1_i1_1	13.17864	5.828733

DN10315_c0_g1_i1_1	1.709515	1.682004
DN10317_c0_g1_i1_1	1.558277	0.9987282
DN10319_c0_g1_i1_1	4.159007	4.141518
DN10320_c0_g1_i1_1	0.6006576	0.3892074
DN10321_c0_g1_i1_1	872.0738	330.1617
DN10324_c0_g2_i1_2	2.772049	1.089969
DN10326_c0_g1_i1_1	27.51345	20.00347
DN10328_c0_g1_i1_2	2.45262	0
DN10329_c0_g1_i1_1	2.358768	0.6137879
DN10330_c0_g1_i1_1	0.2236625	1.21E-23
DN10331_c0_g2_i1_1	0.324868	2.275892
DN10332_c0_g1_i1_1	1.127775	0.5368315
DN10333_c0_g1_i1_1	222.2876	405.5378
DN10334_c0_g1_i1_1	2.407926	2.602929
DN10336_c0_g1_i1_1	0.3508541	0
DN10337_c0_g1_i1_1	1.503781	1.457986
DN10339_c0_g1_i2_1	39.04664	155.1361
DN10340_c0_g1_i1_1	1.099841	1.543498
DN10343_c0_g1_i1_1	0.9837539	28.35182
DN10344_c0_g1_i1_2	1.827677	0
DN10344_c0_g2_i1_1	0.9719413	1.709539
DN10345_c0_g1_i1_1	3.543678	9.676422
DN10346_c0_g1_i1_1	0.7179681	1.351397
DN10348_c0_g1_i1_1	0.4490173	1.55596

DN10358_c0_g1_i1_2	0	0
DN10359_c0_g1_i2_1	0.9288581	2.724487
DN10362_c0_g1_i1_1	0.08823565	1.073161
DN10362_c0_g2_i1_1	1.18594	2.889099
DN10365_c0_g1_i1_1	2.861744	2.523417
DN10368_c0_g1_i1_1	1.3343	1.167209
DN10369_c0_g1_i1_1	1.034566	1.226596
DN10371_c0_g1_i1_1	48.17757	40.06095
DN10373_c0_g1_i1_1	0.1600585	0.2462411
DN10378_c0_g1_i1_1	4.77601	9.10588
DN1037_c0_g1_i1_1	0.4052132	2.292608
DN10380_c0_g1_i1_1	0.2362147	2.451435
DN10381_c0_g1_i1_2	1.263245	0.2785661
DN10382_c0_g1_i1_1	0.9387653	1.038558
DN10385_c0_g1_i1_1	1.858594	1.485436
DN10385_c0_g1_i1_2	28.93823	18.86157
DN10386_c0_g1_i1_1	1.000488	1.159327
DN10388_c0_g2_i1_2	3.050949	0
DN10390_c0_g1_i2_1	1.100136	1.946398
DN10390_c0_g2_i1_1	0.4042388	1.138199
DN10391_c0_g1_i1_1	1.633128	0
DN10393_c0_g1_i1_1	0.7301073	1.108205
DN10401_c0_g2_i1_1	0.6094157	0.4233434
DN10402_c0_g1_i1_1	0.908522	8.190774

DN10402_c0_g1_i1_2	3.184671	3.842934
DN10402_c0_g2_i1_2	1.768814	2.127251
DN10402_c0_g3_i1_2	1.595533	2.247781
DN10402_c1_g1_i1_2	1.941933	1.577606
DN10403_c0_g2_i1_1	0.4962977	1.493952
DN10408_c0_g1_i1_2	3.184796	1.126455
DN10417_c0_g1_i1_1	1.103309	1.181506
DN1041_c0_g1_i1_1	5.620701	6.791546
DN10425_c0_g1_i1_1	0.07252054	0.862036
DN10428_c0_g1_i1_1	0.8438362	3.133105
DN10429_c0_g1_i1_1	0.112491	0.433312
DN1042_c0_g1_i1_2	2.669267	1.330875
DN10430_c0_g1_i1_1	0.901355	0
DN10431_c0_g1_i2_1	0.4573442	1.488612
DN10432_c0_g1_i1_1	0.1863754	0.3694499
DN10433_c0_g1_i1_1	2.926757	9.441853
DN10435_c0_g1_i1_2	1.395622	0.6459225
DN10435_c0_g1_i2_1	0.05244363	0.4827778
DN10439_c0_g1_i1_1	1.714917	2.264271
DN10440_c0_g1_i1_2	7.459373	4.940289
DN10445_c0_g2_i1_1	4.289322	4.30049
DN10446_c0_g1_i1_2	0.9587686	0.2551538
DN10446_c0_g2_i1_2	2.428979	1.031537
DN10448_c0_g1_i1_1	0.9842092	1.378668

DN10448_c0_g1_i1_2	3.471132	1.373807
DN10449_c0_g1_i1_1	0.4781431	0.7723999
DN10453_c0_g1_i1_1	0.3451179	0.6528167
DN10455_c0_g1_i1_2	3.133891	0.2613773
DN10456_c0_g1_i1_1	7.483199	5.044567
DN10459_c0_g1_i1_1	0.9640915	0.5291562
DN10462_c0_g1_i1_2	2.444772	0.4525477
DN10463_c0_g1_i1_1	0	4.723924
DN10463_c0_g1_i1_2	5.766691	0
DN10464_c0_g1_i1_1	0.4473275	1.092256
DN10466_c0_g1_i1_2	4.655144	1.093953
DN10466_c0_g1_i3_1	7.256123	10.69484
DN10467_c0_g1_i1_2	3.057791	1.569307
DN10469_c0_g1_i2_1	2.763406	1.363051
DN10472_c0_g1_i1_1	0.8411753	1.341911
DN10472_c0_g2_i1_1	0.2245748	0.9254328
DN10473_c0_g1_i1_1	3.22238	2.311435
DN10474_c0_g1_i1_1	0.5131936	0.9546772
DN10475_c0_g1_i1_2	2.195069	0.4673069
DN10476_c0_g1_i1_2	3.194681	1.045996
DN10478_c0_g1_i1_1	1.687704	1.410904
DN1047_c0_g1_i1_1	1.092405	4.640875
DN10480_c0_g1_i1_1	0.8240877	1.109588
DN10481_c0_g1_i1_2	0.6524499	0.1264409

DN10483_c0_g1_i1_2	0.8716615	0.9385343
DN10488_c0_g1_i1_1	0.4834956	1.964189
DN10489_c0_g1_i1_2	4.439207	2.486587
DN1048_c0_g1_i1_1	0.1075356	1.83488
DN10490_c0_g1_i1_2	0.5031886	0.5711534
DN10490_c0_g2_i1_2	2.582377	0.1878098
DN10492_c0_g1_i1_1	37.87742	21.00398
DN10493_c0_g1_i2_1	4.428599	3.052299
DN10495_c0_g1_i1_1	2.717529	2.64384
DN10496_c0_g1_i1_1	1.874878	1.577668
DN10498_c0_g1_i1_2	1.867611	0.797966
DN10500_c0_g1_i1_2	2.97083	1.688239
DN10502_c0_g1_i1_1	0	0.7718755
DN10502_c0_g1_i1_2	0.763168	0.4761258
DN10504_c0_g1_i1_1	1.485894	1.930794
DN10509_c0_g1_i1_1	14.3908	4.564181
DN10509_c0_g1_i1_2	0.6134562	0.879811
DN1050_c0_g1_i1_2	1.006735	1.679373
DN1050_c0_g2_i1_2	1.539116	2.352986
DN10515_c0_g1_i1_1	2.637632	1.093206
DN10518_c0_g1_i1_1	3.546481	5.005889
DN1051_c0_g1_i1_1	1.536448	1.22365
DN10523_c0_g1_i1_1	2.24573	3.876491
DN10533_c0_g1_i1_2	2.367172	1.913269

DN10537_c0_g2_i1_1	1.866907	2.960323
DN10540_c0_g1_i1_2	1.775652	0.8492145
DN10542_c0_g1_i1_1	2.687028	3.603277
DN10542_c0_g2_i3_1	14.64522	1.635007
DN10546_c0_g2_i1_2	1.446176	0.5998934
DN10547_c0_g1_i1_1	0.5014657	2.174955
DN10548_c0_g1_i1_1	7.648433	5.171168
DN10552_c0_g1_i2_2	14.70231	5.419605
DN10555_c0_g1_i1_1	2.854703	1.07135
DN10556_c0_g1_i1_1	0.3088959	0.5750654
DN1055_c0_g1_i1_1	0.6762882	1.754362
DN10561_c0_g1_i1_2	2.08454	0
DN10565_c0_g1_i2_2	3.324237	0
DN10571_c0_g1_i2_1	0.8161791	0.6795509
DN10571_c0_g3_i1_1	0.6046627	1.318156
DN10572_c0_g1_i1_2	3.650317	0.7713079
DN10574_c0_g1_i1_1	1.274102	1.773515
DN10574_c0_g2_i1_1	1.399713	0.3571705
DN10578_c0_g1_i1_1	1.357896	2.887295
DN10579_c0_g1_i1_1	1.131934	2.07793
DN1057_c0_g1_i1_1	9.758984	7.751286
DN10582_c0_g1_i1_2	0	0
DN10586_c0_g1_i1_1	1.408878	0.3216072
DN10586_c0_g2_i1_1	0	2.682408

DN10588_c0_g1_i1_1	1.121601	0.9824452
DN10593_c0_g1_i1_1	1.53471	1.194589
DN10593_c0_g2_i1_1	0.953627	1.070159
DN10594_c0_g1_i1_2	1.338474	0.2684085
DN10595_c0_g1_i1_1	6.541478	15.09192
DN1059_c0_g1_i1_1	1.522409	0.5298973
DN105_c0_g1_i1_2	2.672511	1.654476
DN10600_c0_g1_i1_1	6094.674	5167.203
DN10601_c0_g3_i1_1	2.16746	6.029781
DN10602_c0_g1_i1_2	3.295637	1.116007
DN10604_c0_g1_i1_1	0.7494594	0.4760915
DN10612_c0_g1_i1_1	0.249888	0.1220069
DN10613_c0_g1_i2_1	3.800412	3.464269
DN10617_c0_g1_i1_2	1.986626	0.1219598
DN10617_c0_g2_i1_2	5.054591	1.676961
DN10618_c0_g1_i1_2	3.08031	2.320444
DN10620_c0_g1_i1_2	1.963358	0
DN10628_c0_g1_i1_1	4.209119	8.620366
DN10630_c0_g1_i1_1	0	0.7715907
DN10630_c0_g1_i1_2	1.22352	0.9411357
DN10631_c0_g1_i2_2	2.611937	1.40708
DN10637_c0_g1_i1_1	0.8336263	1.554937
DN10641_c0_g1_i1_2	0	0
DN10642_c0_g1_i1_2	1.623219	0.8264928

DN10643_c0_g1_i1_1	0.6122593	2.01478
DN10644_c0_g1_i1_2	61.72674	25.28968
DN10645_c0_g1_i1_2	0.9252917	0.1881204
DN10649_c0_g1_i1_2	6.113675	1.665279
DN10650_c0_g1_i1_1	2.633224	2.148991
DN10651_c0_g1_i1_1	1.815625	4.007039
DN10656_c1_g1_i1_2	0.8745342	2.703501
DN10657_c0_g1_i1_1	1.219173	3.521767
DN10658_c0_g1_i1_1	0.6216531	1.213785
DN10659_c0_g1_i1_2	0.7258626	0.2959892
DN1065_c0_g1_i1_1	0	2.964789
DN10660_c0_g1_i1_1	4.136128	3.890622
DN10665_c0_g1_i1_1	0	2.117775
DN10666_c0_g1_i1_2	4.080227	0.3138849
DN10668_c0_g1_i1_2	7.819761	3.206578
DN10669_c0_g1_i1_1	1.364165	2.16354
DN10669_c0_g1_i1_2	5.149222	0.7015582
DN10675_c0_g1_i1_2	0.7587308	0.3719046
DN10681_c0_g1_i1_1	0.753298	1.690456
DN10682_c0_g2_i1_1	0.8147265	1.295994
DN10686_c0_g1_i1_2	4.033839	0.4902506
DN10687_c0_g1_i1_2	1.559409	0
DN10688_c0_g1_i1_1	1.742389	3.140049
DN1068_c0_g1_i1_2	1.170886	1.454589

DN10690_c0_g1_i1_2	103.416	149.4833
DN10690_c0_g1_i2_1	0	5.149548
DN10692_c0_g1_i1_1	3.099921	4.416223
DN10696_c0_g1_i1_1	0.3163833	2.518685
DN1069_c0_g1_i1_1	0.517181	2.4054
DN10701_c0_g1_i1_1	1.166795	1.01548
DN10702_c0_g1_i1_2	5.529182	2.340696
DN10703_c0_g1_i1_2	1.709688	0.3516239
DN10704_c0_g1_i1_1	4.552708	6.621534
DN10707_c0_g1_i1_1	64.25048	153.6133
DN1070_c0_g1_i1_1	9.352372	4.486365
DN1070_c0_g1_i1_2	1.519949	1.973395
DN10714_c0_g1_i1_1	0	0
DN10714_c0_g2_i1_1	0	0.4993288
DN10715_c0_g2_i1_1	5.424122	2.467729
DN10717_c0_g1_i1_2	3.957565	1.083673
DN10721_c0_g1_i2_2	13.15204	19.48044
DN10724_c0_g1_i1_2	0	0
DN10725_c0_g1_i1_1	0.7426187	2.137078
DN10725_c0_g1_i1_2	69.14503	65.09236
DN10726_c0_g1_i2_1	1.258071	3.714177
DN10728_c0_g1_i1_1	267.3157	163.4639
DN10731_c0_g1_i2_1	1.240341	7.894458
DN10734_c0_g1_i1_1	1.107857	1.071615

DN10742_c0_g1_i2_1	33.04519	11.1557
DN10744_c0_g1_i1_1	1.677886	1.925477
DN10746_c0_g1_i2_2	3.240768	2.556351
DN10748_c0_g1_i2_1	1.861953	1.044517
DN10753_c0_g1_i1_1	3.15496	4.570222
DN10757_c0_g1_i1_1	2.756163	5.135161
DN10759_c0_g1_i1_1	2.165349	3.636136
DN10761_c0_g1_i1_2	0.885874	0.3031353
DN10761_c0_g2_i1_2	1.444006	3.42E-79
DN10763_c0_g1_i4_1	0	1.007162
DN10766_c0_g1_i1_1	1.00218	3.175658
DN10767_c0_g1_i1_1	1.594974	4.369207
DN1076_c0_g1_i1_2	1.532424	0.07881699
DN10770_c0_g1_i1_1	1.896032	2.695243
DN10773_c0_g1_i1_1	2.21029	3.714072
DN10774_c0_g1_i1_1	1.400254	2.180517
DN10774_c0_g1_i1_2	1.323595	0.2410213
DN10775_c0_g1_i1_1	1.435083	1.548754
DN10775_c0_g1_i1_2	3.382165	1.370595
DN10775_c0_g2_i1_1	1.286957	0.7582625
DN10776_c0_g1_i2_1	43.25567	29.33797
DN10778_c0_g1_i1_2	6.341203	2.796273
DN10782_c0_g1_i1_2	3.258201	4.040526
DN10786_c0_g1_i1_1	17.62624	11.35756

DN10787_c0_g1_i1_1	0.6999107	1.537931
DN10798_c0_g1_i1_1	0.4959028	1.966492
DN10798_c0_g2_i1_1	1.047863	0.4576115
DN1079_c0_g1_i1_2	1.87149	0
DN1079_c0_g2_i1_2	1.554461	1.33799
DN107_c0_g1_i1_1	1.712072	1.237675
DN10807_c0_g1_i2_1	2.096858	2.941214
DN10807_c0_g3_i1_1	3.317049	5.236559
DN10808_c0_g1_i1_2	1.536216	1.189969
DN10811_c0_g1_i1_1	0.969855	1.415989
DN10812_c0_g1_i1_1	1.096891	1.027997
DN10812_c0_g2_i1_1	3.155452	0.7021719
DN10813_c0_g1_i1_1	14.08316	39.269
DN10814_c0_g1_i1_1	3.074373	2.144942
DN10815_c0_g1_i2_1	9.180442	22.65768
DN10816_c0_g1_i1_2	3.204444	0.6975089
DN10817_c0_g1_i1_1	17.02774	14.5151
DN10819_c0_g1_i1_1	21.99314	11.13313
DN10823_c0_g2_i1_1	0.9576346	1.947541
DN10823_c0_g3_i1_1	1.374344	1.765661
DN10826_c0_g1_i1_1	0	0.7293399
DN10836_c0_g1_i1_2	0.8589715	0.779529
DN10838_c0_g1_i1_1	2.647007	11.18367
DN10838_c0_g1_i1_2	22.20959	9.225429

DN10839_c0_g2_i1_1	0.6134222	2.329988
DN10840_c0_g1_i1_1	0.9299249	0.1840959
DN10842_c0_g1_i1_1	1.511869	44.48283
DN10845_c0_g1_i1_1	22.08933	325.3546
DN10849_c0_g1_i1_1	1.133141	0.7559056
DN10849_c0_g1_i1_2	1.875857	0.8901966
DN10851_c0_g1_i1_1	0.4469568	0.746395
DN10856_c0_g1_i4_1	2.124712	1.24594
DN10862_c0_g1_i1_1	1.817655	1.195744
DN10862_c0_g1_i2_2	76.30214	43.50413
DN10863_c0_g1_i1_1	409.7622	514.4492
DN10864_c0_g1_i1_1	38.16082	17.97811
DN10866_c0_g1_i1_2	1.514079	0.8078183
DN10866_c0_g1_i3_1	11.49713	11.84145
DN10867_c0_g1_i1_2	2.872746	1.145421
DN10868_c0_g2_i1_1	5.548488	37.04721
DN10869_c0_g1_i1_1	0.788361	3.600039
DN10870_c0_g1_i1_1	1.937473	1.539012
DN10874_c0_g1_i2_1	2.443353	3.179375
DN10877_c0_g1_i1_1	0.3381799	0.8093543
DN10877_c0_g1_i1_2	0	0
DN10881_c0_g1_i1_1	5.093786	4.399541
DN10882_c0_g2_i1_1	0.3831386	2.141347
DN10883_c0_g1_i1_1	1.0506	2.153623

DN10883_c0_g2_i1_1	1.078509	0.6502195
DN10884_c0_g1_i1_1	0	1.13006
DN10884_c1_g1_i1_1	3.272341	8.585653
DN10885_c0_g1_i2_1	1.274537	3.957399
DN10886_c0_g1_i1_1	0.4495197	0.6869198
DN10886_c0_g2_i1_1	0.2878075	0.4427544
DN1088_c0_g1_i1_2	0	0
DN10890_c0_g1_i1_1	0.2798191	2.242306
DN10892_c0_g1_i1_1	0.7207695	2.044522
DN10898_c0_g1_i1_1	0.6039386	14.95372
DN108_c0_g1_i2_1	0.6756778	0.6433081
DN10901_c0_g1_i1_1	0.4092802	1.229508
DN10908_c0_g1_i1_1	1.73767	1.809306
DN10908_c0_g1_i1_2	0.4100845	0.1824139
DN10908_c0_g2_i1_1	0.9639807	1.98745
DN10909_c0_g1_i1_1	0.549398	2.531807
DN10910_c0_g1_i1_1	0.3539441	21.02395
DN10912_c0_g1_i1_1	1.728453	4.904192
DN10917_c0_g1_i1_1	1.837098	1.210245
DN10918_c0_g1_i1_1	1.395363	1.285436
DN10923_c0_g1_i1_2	0.4679612	0.7693368
DN10926_c0_g1_i1_1	5.027761	0.3789951
DN10929_c0_g1_i1_1	5.861897	2.01882
DN10930_c0_g1_i2_1	0.2211137	0.5921046

DN10934_c0_g1_i1_2	1.593117	0.6046026
DN10935_c0_g1_i1_1	2.22749	14.5836
DN10936_c0_g2_i1_2	1.290139	0.538289
DN10939_c0_g1_i1_1	111.1444	130.4118
DN1093_c0_g1_i1_1	3.27948	5.709477
DN10943_c0_g1_i1_1	1.823624	3.2225
DN10947_c0_g1_i1_1	0.9743691	2.272803
DN10954_c0_g1_i1_1	0.7874727	1.512546
DN10954_c0_g2_i1_1	0.4866654	1.756989
DN10955_c0_g1_i1_2	0.5366073	1.324024
DN10955_c1_g1_i1_2	0.8764142	0.4908895
DN10956_c0_g1_i1_2	0.9327101	1.919412
DN10958_c0_g1_i1_1	1.859903	4.182631
DN10959_c0_g1_i1_1	110.5535	96.39736
DN10962_c0_g1_i2_1	0.9571206	1.221887
DN10962_c0_g2_i1_1	0.3301173	0.73648
DN10963_c0_g1_i1_1	14.41464	8.300956
DN10967_c0_g1_i1_1	0	0
DN1096_c0_g1_i1_1	2.848311	3.633622
DN1096_c0_g1_i1_2	0.4896571	0
DN10971_c0_g1_i1_1	1.082414	1.125088
DN10973_c0_g1_i1_1	97.66343	130.522
DN10974_c0_g1_i1_2	1.263506	0
DN10978_c0_g1_i1_1	2.261049	0

DN10984_c0_g2_i1_2	0.2275646	0.5419325
DN10987_c0_g1_i2_1	1.76505	1.039572
DN10990_c0_g2_i1_1	1.051035	0.6912349
DN10991_c0_g1_i1_1	1.479413	1.902951
DN10993_c1_g1_i1_1	0.5581267	1.265469
DN10997_c0_g1_i1_2	1.165567	1.308044
DN10998_c0_g1_i1_1	2.810799	2.915538
DN11003_c0_g1_i1_1	74.32751	40.83723
DN11003_c0_g1_i1_2	4.399507	1.226529
DN11005_c0_g1_i1_1	1.89232	2.447591
DN11006_c0_g1_i1_1	1.503606	1.184345
DN11009_c0_g1_i1_1	3.090054	2.771889
DN11010_c0_g1_i1_1	0.3621574	1.181385
DN11010_c0_g1_i1_2	3.804561	0.5692154
DN11010_c0_g2_i1_1	0.4725368	2.790835
DN11013_c0_g1_i2_1	1.01372	3.638803
DN11022_c0_g1_i1_1	1.892362	2.639712
DN11023_c0_g1_i1_2	4.441583	2.354333
DN11029_c0_g1_i1_2	1.236401	0.872707
DN11031_c0_g1_i1_1	19.01088	16.95536
DN11031_c0_g1_i1_2	0.8487051	0.261124
DN11031_c0_g2_i1_2	0.9443815	0.530279
DN11032_c0_g1_i1_1	1.079451	1.2609
DN11032_c0_g1_i1_2	2.696895	1.248456

DN11037_c0_g1_i1_1	1.565959	0.7354825
DN11037_c0_g2_i1_1	1.590669	0.6391297
DN11037_c0_g3_i1_1	1.400922	3.356518
DN11038_c0_g1_i1_1	1.145149	0.6534587
DN11039_c0_g1_i1_1	9.957492	5.292056
DN1103_c0_g1_i1_1	0.486156	0.8526364
DN11044_c0_g2_i1_1	13.76606	9.027386
DN11044_c0_g3_i1_1	2.230947	4.544631
DN11046_c0_g1_i1_1	0	2.817642
DN11047_c0_g1_i1_1	1.386931	0.2203796
DN11047_c0_g1_i1_2	6.280999	2.885113
DN11049_c0_g1_i1_1	1.628944	1.128616
DN1104_c0_g1_i1_1	0	1.53488
DN11054_c0_g1_i1_1	1.787506	1.400782
DN11056_c0_g1_i1_2	0.7457301	0.3526856
DN11058_c0_g1_i1_1	1.047537	0
DN11060_c0_g1_i1_1	0.7829514	1.506291
DN11061_c0_g1_i1_1	4.192814	3.825013
DN11062_c0_g1_i1_2	0.8820686	3.645514
DN11065_c0_g1_i1_1	57.86449	18.43215
DN11066_c0_g1_i1_1	1.431637	2.218906
DN11073_c0_g1_i1_1	0.1636472	2.816772
DN11074_c0_g1_i1_1	2.239417	6.021881
DN11080_c0_g1_i1_1	4.441813	1.654121

DN11081_c0_g1_i1_1	0.6666479	0.6387888
DN11082_c0_g1_i2_1	22.15204	25.76747
DN11084_c0_g1_i1_1	2.13464	2.882311
DN11087_c0_g1_i1_2	4.481634	3.281756
DN1108_c0_g1_i1_1	0.4121632	3.024684
DN11094_c0_g1_i1_1	23.62177	14.38498
DN11095_c0_g2_i1_1	0	2.280791
DN11096_c0_g1_i1_1	0	2.04678
DN11097_c0_g1_i1_1	1.69109	1.178355
DN11105_c0_g1_i2_2	0	0.009777137
DN11107_c0_g1_i1_2	12.67382	17.91292
DN11111_c0_g1_i1_1	10.94539	2.816288
DN11112_c0_g1_i1_1	13.59911	2.889922
DN11116_c0_g1_i1_1	0.5554483	1.657911
DN11116_c0_g2_i1_1	0.2570211	1.058846
DN11118_c0_g1_i1_1	0.6762023	0.1637527
DN11119_c0_g1_i1_2	42.74001	25.54328
DN11120_c0_g2_i1_1	2871.861	2943.359
DN11133_c0_g1_i1_1	12.68361	10.4641
DN11133_c0_g1_i2_2	1.206843	0
DN11134_c0_g3_i1_2	2.867755	2.564091
DN11137_c0_g1_i1_1	92.95407	62.90579
DN11139_c0_g1_i1_1	1.058852	1.673249
DN11140_c0_g1_i1_1	1.835865	3.654734

DN11144_c0_g1_i1_2	2.806774	0.2743575
DN11148_c0_g1_i1_1	1.327749	2.338646
DN11150_c0_g2_i1_1	1.757261	0.4147156
DN11156_c0_g1_i1_1	0.09788112	1.465891
DN11160_c0_g1_i1_2	1.434879	0.8574143
DN11161_c0_g1_i1_2	0	0
DN11164_c0_g1_i1_1	1.538494	3.905372
DN11169_c0_g1_i1_1	0.3262911	1.01501
DN11170_c0_g1_i1_1	82.70737	56.89142
DN11170_c0_g1_i1_2	1.844283	2.516919
DN11173_c0_g1_i1_1	1.213885	0.9447866
DN11175_c0_g2_i1_1	1.724951	1.551851
DN11176_c0_g1_i1_1	2.537335	2.375007
DN11177_c0_g1_i1_1	0.8624122	0.8222374
DN11177_c0_g2_i1_1	1.296676	1.239243
DN11177_c0_g3_i1_1	0.2196208	1.175015
DN11182_c0_g1_i1_1	0.2841539	0.4792924
DN11183_c0_g1_i1_1	1.237075	3.111494
DN11183_c0_g2_i1_1	0.6610874	2.457531
DN11184_c0_g1_i1_1	3.439639	3.365834
DN11184_c0_g1_i1_2	2.258225	2.008267
DN11185_c0_g1_i1_1	1.416905	1.752942
DN11192_c0_g1_i1_1	19.12922	14.88574
DN11196_c0_g1_i2_1	0.7114007	0.8776735

DN11202_c0_g1_i1_2	22.44791	17.59945
DN11203_c0_g1_i1_1	0	1.622028
DN11204_c0_g1_i1_1	0.8137383	0.8462291
DN11204_c0_g2_i1_1	0.2899881	0.4438082
DN11204_c0_g3_i1_1	0.3106943	0.8424247
DN11205_c0_g1_i1_1	1.522431	2.083372
DN11208_c0_g1_i1_2	0.8367636	0.7456366
DN1120_c0_g1_i1_2	0	0.4906702
DN11210_c0_g1_i1_1	0.06727852	1.025599
DN11212_c0_g1_i1_1	0.1648392	1.126324
DN11212_c0_g2_i1_1	0.1260475	1.922812
DN11216_c0_g1_i1_1	5.009088	7.551358
DN11218_c0_g1_i1_1	1.042106	1.043433
DN11219_c0_g1_i2_1	0.2241512	5.616298
DN11223_c0_g1_i1_2	1.739452	0.6047925
DN11223_c0_g1_i2_1	159.8213	56.28951
DN11227_c0_g1_i2_1	0.4802953	2.440385
DN11235_c0_g1_i1_1	1.270601	0.7316837
DN11240_c0_g1_i1_1	26.99451	9.130375
DN11242_c0_g1_i1_1	0.4251443	0.8066742
DN11244_c0_g1_i1_1	5.315416	5.497667
DN11246_c0_g1_i1_1	4.463499	1.817694
DN11246_c0_g1_i1_2	5.193678	2.418753
DN11251_c0_g1_i1_1	1.465875	1.524972

DN11257_c0_g1_i1_1	1.485885	2.704153
DN11258_c0_g1_i1_2	0	0
DN11260_c0_g1_i1_1	1.05235	1.79778
DN11262_c0_g1_i1_1	2.694107	1.558325
DN11263_c0_g1_i1_1	1.051735	0.6021701
DN11264_c0_g1_i1_1	1.67155	3.92627
DN11265_c0_g1_i1_2	0.7599114	0.788628
DN11270_c0_g1_i1_1	0.5102652	0.5120243
DN11279_c0_g1_i1_1	0.9874092	21.69646
DN11285_c0_g1_i1_1	0.8234222	1.012682
DN11287_c0_g1_i1_1	2.059915	3.41998
DN11289_c0_g1_i1_2	2.449005	1.84691
DN11290_c0_g1_i1_2	3.162085	3.89172
DN11291_c0_g1_i1_1	5.089599	2.565558
DN11299_c0_g1_i1_2	1.379028	0.6876531
DN11299_c0_g1_i2_1	19.79093	60.97046
DN11305_c0_g1_i2_1	1.156237	4.488251
DN11308_c0_g1_i3_1	0.7438282	4.562505
DN11309_c0_g1_i1_2	1.194446	0
DN11311_c0_g1_i1_2	0	0
DN11312_c0_g1_i1_1	2.156835	5.268965
DN11313_c0_g1_i1_2	0	0
DN1131_c0_g1_i1_1	0.3844663	0.4549135
DN11321_c0_g1_i1_2	0.7805778	1.643593

DN11323_c0_g1_i1_2	5.806946	0.5417373
DN11324_c0_g1_i1_2	7.209138	5.370777
DN11325_c0_g1_i1_1	0.32961	0.5739076
DN11327_c0_g1_i1_1	3.918025	1.155145
DN11330_c0_g1_i1_1	0.2467344	0.6755573
DN11331_c0_g1_i1_1	1.084872	3.255981
DN11332_c0_g1_i1_1	0.7296847	0.7105858
DN11333_c0_g1_i1_1	3.389866	5.223348
DN11335_c0_g1_i1_2	3.069571	0
DN11336_c0_g1_i1_1	0.9270431	2.023966
DN11337_c0_g1_i1_2	0.5940089	0.4129083
DN11338_c0_g1_i1_2	12.21426	3.252339
DN11339_c0_g1_i1_1	15.26903	20.90394
DN11343_c0_g1_i1_2	2.543057	0
DN11347_c0_g1_i1_2	0.7669379	1.566219
DN1134_c0_g1_i1_2	1.506758	0.8232105
DN1134_c0_g1_i2_1	0.724311	0.1481034
DN11355_c0_g1_i1_1	4.020223	1.185779
DN11356_c0_g1_i1_2	0.5663737	0.7359154
DN11361_c0_g1_i1_1	0.4529734	0.9791644
DN11364_c0_g1_i1_1	0.6510248	0.5639515
DN11371_c0_g1_i1_1	1.92151	2.211303
DN11371_c0_g1_i1_2	1.603348	0.8913566
DN11376_c0_g1_i1_2	14.51463	4.15416

DN11377_c0_g1_i1_1	2.220107	1.698786
DN11380_c0_g1_i1_1	6.624286	4.382295
DN11381_c0_g1_i1_1	0.582916	1.333789
DN11384_c0_g1_i1_1	1.152489	0.3644602
DN11388_c0_g1_i2_2	8.076655	33.5873
DN11404_c0_g1_i1_1	0.5163568	4.31606
DN11404_c0_g1_i1_2	40.49521	28.92699
DN11408_c0_g1_i1_2	1.120291	0.4279493
DN11410_c0_g1_i1_2	0.3076514	0.7012173
DN11413_c0_g1_i1_1	1.854913	12.57335
DN11414_c0_g1_i1_2	78.22424	118.7989
DN11415_c0_g1_i1_1	2.507135	5.110263
DN11418_c0_g1_i1_1	126.3533	90.72173
DN1141_c0_g1_i1_1	1.563516	1.665763
DN11424_c0_g1_i1_2	1.501065	0
DN11433_c0_g1_i1_1	0.9538826	2.151119
DN11439_c0_g1_i1_1	2.048233	1.349325
DN11439_c0_g1_i2_2	1.117702	0.1330986
DN11440_c0_g1_i1_1	22.94092	11.85091
DN11441_c0_g1_i3_1	1.131023	1.81415
DN11442_c0_g2_i1_1	3.757745	4.565805
DN11444_c0_g2_i1_1	7.360972	29.40836
DN11446_c0_g1_i1_1	0.8674394	2.356901
DN11447_c0_g1_i1_2	1.744615	1.196199

DN1144_c0_g1_i1_1	1.196674	3.240097
DN11450_c0_g1_i1_2	1.440507	0.4852298
DN11451_c0_g1_i2_2	2.029621	0.6132793
DN11453_c0_g1_i1_1	0.4476739	1.456833
DN11453_c0_g2_i1_1	1.927205	2.150828
DN11453_c0_g3_i1_1	1.338156	2.023291
DN11455_c0_g1_i1_1	0.930755	1.927872
DN11455_c0_g2_i1_1	1.03909	4.168328
DN11458_c0_g1_i1_1	0.5940963	2.97714
DN11458_c0_g1_i1_2	10.00196	11.40927
DN11462_c0_g1_i2_1	1.531866	3.892541
DN11465_c0_g1_i2_1	2.381461	5.072111
DN11467_c0_g1_i1_1	0.9524491	2.439494
DN11469_c0_g1_i1_1	3.34743	2.648205
DN11469_c0_g1_i1_2	2.119283	1.120827
DN11479_c0_g1_i1_1	1.243677	0.5103034
DN11479_c0_g1_i1_2	1.143127	0.4346285
DN11480_c0_g2_i2_1	1.399909	3.317913
DN11480_c1_g1_i1_1	1.721425	1.081473
DN11482_c0_g1_i1_2	2.56192	0.4977636
DN11485_c0_g1_i1_1	22.58064	16.0428
DN11488_c0_g1_i1_2	2.77408	1.983397
DN11494_c0_g1_i1_1	0.0905289	0.3322757
DN11495_c0_g1_i1_1	0.6353544	0.3041904

DN11497_c0_g1_i1_2	77.93393	65.87496
DN11497_c0_g2_i1_1	0.8969168	11.95009
DN1149_c0_g1_i1_1	1.652753	2.220848
DN1149_c0_g1_i1_2	1.622	0.0993436
DN11500_c0_g1_i1_1	2.724906	1.342804
DN11503_c0_g1_i1_1	0.1978253	3.005815
DN11507_c0_g1_i1_1	160.4967	39.72743
DN11511_c0_g1_i1_1	15.14685	9.624031
DN11512_c0_g1_i1_1	1.736176	1.524031
DN11517_c0_g1_i1_1	1.995269	5.69695
DN11518_c0_g1_i1_1	0.8338265	1.670957
DN11518_c0_g2_i1_1	0.8211251	1.707007
DN11521_c0_g1_i1_1	3.363006	4.350324
DN11522_c0_g1_i1_1	1.856101	0.3046676
DN11528_c0_g1_i1_2	1.767269	0.3409767
DN11533_c0_g2_i1_2	1.212841	0.3196639
DN11536_c0_g1_i2_1	2.874611	3.053492
DN11537_c0_g1_i1_1	1.355558	2.076783
DN11546_c0_g2_i1_1	5.402145	5.079389
DN11549_c0_g1_i1_1	1.132253	8.668805
DN11553_c0_g1_i1_1	1.619457	2.065462
DN11553_c0_g1_i2_2	70.28618	47.04198
DN11553_c0_g2_i1_1	1.618817	1.0111
DN11553_c0_g3_i1_1	2.801772	3.225659

DN11554_c0_g1_i1_2	9.675351	5.10114
DN11558_c0_g1_i1_1	4.032388	2.21893
DN11559_c0_g1_i1_1	12.27319	49.7093
DN11561_c0_g1_i1_1	1.740878	4.828313
DN11561_c0_g1_i1_2	0.4319336	0.2290314
DN11564_c0_g1_i2_2	1.543876	3.141674
DN11565_c0_g1_i1_2	2.274475	1.47678
DN11566_c0_g1_i1_2	1.247985	0
DN11570_c0_g2_i1_1	0.3139897	2.311999
DN11579_c0_g1_i2_1	3.666522	2.62854
DN11583_c0_g2_i1_1	53.81226	51.32487
DN11584_c0_g2_i1_1	1.529505	0.4925705
DN11586_c0_g1_i4_1	209.7586	195.6153
DN11589_c0_g1_i1_1	1.884048	3.312806
DN11591_c0_g1_i2_1	2.481291	1.794595
DN11591_c0_g1_i2_2	0	0
DN11592_c0_g1_i1_2	13.93482	9.16424
DN11594_c0_g1_i1_2	1.969568	1.483305
DN11596_c0_g1_i1_1	1.398086	1.079444
DN11596_c0_g2_i1_1	1.126258	3.583711
DN11597_c0_g1_i1_1	13.53787	13.65613
DN1159_c0_g1_i1_1	15.73624	8.377944
DN11600_c0_g1_i1_1	17.0045	38.62894
DN11603_c0_g1_i2_1	5.350133	3.999849

DN11606_c0_g1_i1_1	0.1947697	0.4602618
DN11607_c0_g1_i1_1	2.938101	5.615713
DN11610_c0_g1_i1_1	0.5738943	5.925957
DN11611_c0_g1_i1_1	1.569427	0.8647906
DN11611_c0_g1_i1_2	0	0
DN11612_c0_g1_i1_1	9.603964	19.72079
DN11614_c0_g1_i1_1	0.4675607	2.202239
DN11615_c0_g1_i1_2	1.68545	0.5475922
DN1161_c0_g1_i1_1	1.292675	0.4757143
DN11622_c0_g1_i1_1	1.077342	0.3166126
DN11625_c0_g1_i1_1	1.086723	0.4060608
DN11626_c0_g1_i1_1	0.9403617	1.157773
DN11628_c0_g1_i1_1	1.048826	1.584862
DN11634_c0_g1_i1_2	1.281333	1.005804
DN11640_c0_g1_i1_1	0	0.1114168
DN11641_c0_g1_i1_1	1.095972	0.5194401
DN11641_c0_g2_i1_1	0.9091076	1.467836
DN11643_c0_g1_i1_1	0.9487261	0.9777391
DN11645_c0_g1_i1_1	82.54406	111.2365
DN11645_c0_g1_i1_2	2.537539	5.88E-05
DN11650_c0_g1_i2_1	26.17107	23.61634
DN11654_c0_g1_i1_2	20.04324	9.460898
DN11656_c0_g1_i1_1	3.776953	6.074985
DN11657_c0_g2_i1_1	0	1.022618

DN11658_c0_g1_i1_1	1.305098	6.747123
DN11658_c0_g1_i1_2	2.148344	0.9614245
DN11659_c0_g1_i1_1	0.348197	9.459165
DN11663_c0_g1_i1_1	0.6655626	1.268022
DN11665_c0_g1_i1_1	3.996556	1.962079
DN11668_c0_g1_i1_1	0.6220229	1.887181
DN11671_c0_g1_i1_1	0.6678926	1.18795
DN11671_c0_g2_i1_1	0.6098559	0.5734829
DN11680_c0_g1_i1_2	3.199107	0.1133649
DN11682_c0_g1_i2_1	4.235897	0.5579924
DN11682_c0_g1_i2_2	3.115328	0.6618249
DN11683_c0_g1_i1_1	2.425309	2.502097
DN11684_c0_g1_i1_1	4.196075	9.949612
DN11687_c0_g2_i1_2	1.648134	2.391843
DN1168_c0_g1_i1_2	0	0
DN11690_c1_g1_i1_2	9.651341	3.654672
DN11699_c0_g1_i1_2	13.10372	7.504802
DN11699_c0_g2_i1_2	5.60587	2.133396
DN11700_c0_g1_i1_2	1.329411	1.290518
DN11702_c0_g1_i1_1	2.068721	2.142187
DN11705_c0_g1_i1_1	1.012299	0.6976078
DN11709_c0_g1_i1_2	2.5082	1.572909
DN11710_c0_g1_i2_2	13.62561	8.681345
DN11711_c0_g1_i1_1	0.7927136	0.5806902

DN11711_c0_g1_i1_2	0.9010597	0.3357518
DN11713_c0_g1_i1_1	3.146184	7.122121
DN11713_c0_g1_i1_2	2.882485	0.5544751
DN11716_c0_g1_i1_1	0.7271345	1.322247
DN11716_c0_g1_i1_2	2.388892	0.7411538
DN11729_c0_g1_i1_1	1.023138	1.683148
DN1172_c0_g1_i1_1	0	2.309347
DN11731_c0_g1_i1_1	2.862019	2.738983
DN11733_c0_g1_i1_1	2.241855	3.050105
DN11733_c0_g2_i1_1	0.2059463	0.1745693
DN11733_c0_g3_i1_1	0.9850014	1.033207
DN11734_c0_g1_i4_1	55.84254	56.89454
DN11737_c0_g1_i1_2	3.978653	1.029816
DN11743_c0_g1_i1_2	3.681514	2.814584
DN11746_c0_g1_i1_1	1.275801	2.73643
DN11749_c0_g1_i1_1	2.037482	2.379936
DN1174_c0_g1_i1_1	0.9939795	2.783251
DN11751_c0_g1_i1_1	0.09387982	3.25237
DN11752_c0_g1_i1_1	0.8861548	22.28273
DN11752_c0_g2_i1_1	4.979369	18.0125
DN11755_c0_g1_i1_2	3.036338	1.851686
DN11756_c0_g1_i1_1	0.6607756	1.17039
DN11757_c0_g1_i1_1	1.091937	7.652652
DN11758_c0_g1_i1_1	0.3250923	0.2943725

DN11761_c0_g1_i2_1	2.976349	1.859906
DN11762_c0_g1_i1_1	0.8504559	0.3096735
DN11765_c0_g1_i1_1	0.1995419	1.81986
DN11765_c0_g1_i1_2	1.330571	0.5340956
DN11769_c0_g2_i1_1	0.4177311	0
DN11771_c0_g1_i1_1	0.68837	3.230444
DN11774_c0_g1_i1_1	1.208358	0.4003362
DN11777_c0_g1_i3_1	0.5000955	3.266423
DN11778_c0_g1_i1_2	0	0
DN11782_c0_g1_i1_1	0.3425703	0.3175993
DN11783_c0_g1_i1_1	0.2902346	3.228239
DN11784_c0_g1_i1_1	1.060549	2.492422
DN11784_c0_g2_i1_1	0.702713	2.198852
DN11786_c0_g1_i2_1	0.9450275	0.9815152
DN11789_c0_g1_i1_2	1.070796	0.68624
DN11790_c0_g1_i1_1	2.214743	2.660609
DN11791_c0_g1_i1_2	1.514015	0
DN11797_c0_g1_i1_1	0.4289036	5.11488
DN117_c0_g1_i1_2	4.253205	8.411535
DN11800_c0_g1_i1_1	48.81398	24.40192
DN11800_c0_g2_i1_2	0.2135048	0.162763
DN11801_c0_g1_i1_1	3.179384	3.236167
DN11801_c0_g1_i1_2	2.417175	3.320598
DN11805_c0_g2_i1_1	0.2676926	2.452031

DN11806_c0_g1_i1_1	1.71E-21	0.3307979
DN11816_c0_g1_i2_1	1.830458	2.636212
DN1181_c0_g1_i1_2	8.705412	4.808321
DN11821_c0_g1_i1_1	0.9374258	2.377111
DN11821_c0_g1_i1_2	4.730057	3.873837
DN11822_c0_g1_i1_1	0.7390601	0.4063142
DN11830_c0_g1_i1_1	9.631659	38.94723
DN11834_c0_g1_i1_1	1.124345	5.846848
DN11836_c0_g1_i1_2	8.424619	11.58515
DN11840_c0_g1_i1_1	2.955748	2.368395
DN11844_c0_g1_i1_1	0.2471288	0.6524136
DN11850_c0_g1_i2_1	18.50464	17.61013
DN11855_c0_g1_i1_1	2.459011	7.780832
DN11860_c0_g1_i1_1	1.32396	1.47862
DN11861_c0_g1_i1_1	3.509371	3.520212
DN11864_c0_g1_i1_1	10.19905	10.32369
DN11865_c0_g1_i1_1	3.64595	4.295296
DN11867_c0_g1_i1_2	2.03571	0.5962146
DN11867_c0_g2_i1_2	1.615271	0.6196588
DN1186_c0_g1_i1_2	2.015001	0.4705286
DN11877_c0_g1_i1_1	2.590763	0.9564108
DN11878_c0_g1_i1_1	28.49232	20.96183
DN11879_c0_g1_i1_2	7.284577	4.735997
DN11880_c0_g1_i1_1	0.9751458	11.8183

DN11887_c0_g1_i1_1	5.635624	4.658443
DN11888_c0_g1_i1_1	0.2448166	2.491455
DN11890_c0_g1_i1_2	1.089493	0.2430834
DN11890_c0_g1_i2_1	1.435988	2.526533
DN11891_c0_g1_i1_2	2.13E-09	0.6158997
DN11895_c0_g1_i1_1	1.849017	1.64271
DN11895_c0_g1_i1_2	2.730237	1.918057
DN11900_c0_g1_i1_2	8.281977	8.450917
DN11903_c0_g1_i1_2	42.0609	19.00891
DN11907_c0_g1_i1_1	0.5385607	5.335519
DN11909_c0_g1_i1_1	3.570891	8.26761
DN11909_c0_g2_i1_1	1.799022	4.403364
DN1190_c0_g1_i1_1	0.2503037	0.3029794
DN11919_c0_g1_i1_1	9.860148	15.22185
DN11920_c0_g1_i1_1	6.378863	9.550665
DN11922_c0_g1_i1_1	1.684782	1.172308
DN11925_c0_g1_i1_1	1.230665	1.609805
DN1192_c0_g1_i1_2	3.446128	0.9939444
DN11931_c0_g1_i1_1	6.533421	1.960503
DN11933_c0_g1_i1_1	2.292848	3.528251
DN11933_c0_g1_i2_2	3.982709	0.6183801
DN11934_c0_g1_i1_1	1.688099	2.234019
DN11934_c0_g2_i1_1	0.6383477	2.47526
DN11936_c0_g2_i1_1	1.839089	1.817442

DN11938_c0_g1_i1_2	1.826848	0.5312935
DN11942_c0_g1_i1_2	2.229772	0.3640195
DN11947_c0_g1_i1_1	0.5772167	1.521697
DN11953_c0_g1_i1_1	0.8310527	2.976581
DN11954_c0_g1_i1_2	1.126616	1.381374
DN11955_c0_g1_i1_1	2.388998	3.079588
DN11958_c0_g1_i1_2	4.648564	3.762775
DN11959_c0_g1_i1_1	1.527293	2.534453
DN11961_c0_g1_i1_2	2.708866	4.823799
DN11963_c0_g1_i1_2	2.265331	2.29946
DN11971_c0_g1_i4_1	1.41738	3.195647
DN11974_c0_g1_i1_2	3.504967	5.860731
DN11978_c0_g1_i1_1	208.184	174.0498
DN1197_c0_g1_i1_2	10.4186	13.27382
DN11981_c0_g1_i1_1	0.7590272	1.976653
DN11981_c0_g2_i1_1	1.03226	5.294794
DN11982_c0_g1_i1_2	1.881183	2.423276
DN11984_c0_g1_i1_1	1.882248	1.944044
DN11984_c0_g1_i1_2	2.111944	0.866149
DN1198_c0_g1_i1_1	3.254243	4.100719
DN11990_c0_g1_i1_1	2.659157	1.421916
DN11991_c0_g1_i1_2	2.58494	1.796705
DN11991_c0_g2_i1_2	8.253053	2.907053
DN11993_c0_g1_i1_1	0.7813638	1.739251

DN11995_c0_g1_i1_1	0.7722773	1.07251
DN11996_c0_g1_i1_1	0.759636	0.6299862
DN11998_c0_g1_i1_1	3.706193	7.740343
DN11998_c0_g2_i1_2	1.762858	1.019254
DN11999_c0_g1_i1_1	0.1568487	0.4465565
DN12000_c0_g1_i1_2	3.420963	1.060023
DN12000_c0_g2_i1_1	1.661833	13.38237
DN12002_c0_g1_i2_1	2.073321	2.408097
DN12002_c0_g2_i1_1	0.7702154	0
DN12007_c0_g1_i2_2	0.2609835	0.7970634
DN12009_c0_g1_i1_1	3.997598	5.193143
DN12009_c0_g1_i1_2	4.383925	0.5453749
DN12011_c0_g1_i1_1	1.198054	2.651476
DN12013_c0_g1_i2_1	1.281235	0.4497407
DN12014_c0_g1_i1_1	10.73845	8.338968
DN12015_c0_g1_i1_1	0.8127003	1.285258
DN12016_c0_g1_i1_1	0.837225	0.7014114
DN12019_c0_g1_i1_1	0.448959	4.348957
DN12021_c0_g1_i1_1	1.352691	1.674574
DN12023_c0_g1_i1_1	1.234452	3.618688
DN12024_c0_g1_i4_1	1.288938	2.838149
DN12028_c0_g1_i1_2	2.352304	1.082258
DN12029_c0_g1_i1_1	1.387658	3.189097
DN1202_c0_g1_i1_2	5.173415	3.351139

DN12030_c0_g2_i1_1	1.272238	1.651343
DN12035_c0_g1_i1_1	0.9013532	1.893703
DN12035_c0_g1_i2_2	0.6780983	1.12E-27
DN12038_c0_g1_i1_1	2.155293	1.758672
DN12042_c0_g1_i1_2	3.736209	1.411332
DN12045_c0_g1_i1_1	0.09251132	0.7528582
DN12045_c1_g1_i1_1	0	0.7225759
DN12046_c0_g1_i1_1	5.178915	10.97519
DN12047_c0_g1_i1_1	0.8946978	3.251788
DN12047_c0_g1_i1_2	1.826923	0.5115186
DN12048_c0_g1_i1_1	2.410944	16.96041
DN12052_c0_g1_i1_2	4.586325	0.8367627
DN12053_c0_g1_i1_1	0.2773147	0.2771034
DN12055_c0_g1_i1_2	8.310345	1.268781
DN12056_c0_g1_i1_1	0.4142916	0.4316429
DN12057_c0_g1_i1_1	1.175562	1.017642
DN12058_c0_g1_i1_2	0	0
DN12060_c0_g1_i1_1	0.6381129	0.6059309
DN12062_c0_g1_i1_1	6.745016	7.083889
DN12063_c0_g1_i1_1	1.869128	2.000086
DN12064_c0_g2_i1_2	1.271524	0.3468769
DN12069_c0_g1_i1_1	1.755423	1.966131
DN1206_c0_g1_i1_2	1.262652	0.1447699
DN12077_c0_g1_i1_2	6.018082	1.921807

DN12079_c0_g1_i1_2	2.317592	2.082272
DN12080_c0_g1_i1_1	49.93661	48.24037
DN12080_c0_g1_i1_2	4.62779	2.976161
DN12082_c0_g1_i1_2	3.632491	1.083993
DN12086_c0_g1_i1_1	0.7967367	0.7065984
DN12086_c0_g1_i1_2	7.052456	5.005551
DN12088_c0_g1_i1_2	2.151055	0.2107305
DN1208_c0_g1_i1_1	3.959762	2.036945
DN1208_c0_g1_i1_2	3.289985	0.2166359
DN12092_c0_g1_i1_1	0	1.075367
DN12094_c0_g1_i1_1	1.065963	6.53645
DN12096_c0_g2_i1_1	2.321602	12.87669
DN12098_c0_g1_i1_1	1.324755	3.542982
DN1209_c0_g1_i1_1	0.9355684	6.074263
DN12101_c0_g1_i1_1	2.017397	1.912309
DN12106_c0_g1_i1_1	1.27865	2.545713
DN12107_c0_g2_i1_1	0.3580294	2.559926
DN12108_c0_g1_i1_1	0.3023673	1.499467
DN12111_c0_g1_i1_1	0.9247144	2.777188
DN12113_c0_g1_i1_1	3.031823	9.277972
DN12114_c0_g1_i2_1	2.868318	2.492086
DN12121_c0_g1_i1_2	6.0913	9.965221
DN12123_c0_g1_i1_1	1.652646	1.812722
DN12124_c0_g1_i1_2	0.561204	2.850807

DN12126_c0_g1_i2_1	2.69644	4.666605
DN12127_c0_g1_i1_1	1.504433	0.654585
DN12127_c0_g1_i1_2	0.3371723	1.22275
DN12127_c0_g2_i1_2	2.699924	0.9521405
DN12127_c0_g3_i1_2	1.77693	0.7971004
DN12128_c0_g1_i1_1	3.977736	4.06961
DN12129_c0_g1_i1_1	1.174905	7.635097
DN12130_c0_g1_i1_1	0.2560478	0.9088881
DN12133_c0_g1_i4_1	0.1635175	1.237555
DN12137_c0_g1_i2_1	1.4754	0.8633332
DN12137_c0_g1_i2_2	2.373597	3.010921
DN12139_c0_g1_i1_1	0.8445343	3.181114
DN1213_c0_g1_i1_1	0.8308248	0.7885439
DN12140_c0_g1_i2_1	4.058026	6.763524
DN12141_c0_g1_i1_1	1.777844	1.697435
DN12151_c0_g1_i1_1	2.050715	1.57196
DN12151_c0_g1_i1_2	0	0
DN12155_c0_g1_i1_1	0.8926425	0.492463
DN12156_c0_g1_i1_1	0.494883	1.405515
DN12157_c0_g1_i1_1	0	0.5508188
DN12158_c0_g1_i1_1	1.446	0.8235564
DN12162_c0_g1_i1_2	55.47208	37.66101
DN12165_c0_g1_i1_2	2.578553	1.678219
DN1216_c0_g1_i1_2	5.214823	5.722376

DN12172_c0_g1_i1_2	2.399296	0.06218685
DN12173_c0_g1_i1_1	0.4219532	2.267142
DN12173_c0_g2_i1_1	0.8553796	1.619043
DN12175_c0_g2_i1_1	1.271772	0.8807304
DN12181_c0_g1_i1_1	1.059806	2.645954
DN12186_c0_g1_i1_1	13.97992	17.90879
DN12189_c0_g1_i1_2	9.998715	1.279171
DN1218_c0_g1_i2_1	33.40133	22.14233
DN12190_c0_g1_i1_1	0.973764	2.150684
DN12191_c0_g1_i1_1	0.9083302	1.704356
DN12191_c0_g2_i1_1	0.4573378	1.905582
DN12192_c0_g1_i1_1	1.413193	1.117714
DN12193_c0_g1_i1_2	1.590552	1.088061
DN12193_c0_g2_i1_2	2.124413	0.7370317
DN12197_c0_g1_i1_2	3.225629	1.39E-13
DN12199_c0_g1_i1_1	41.26604	19.94547
DN12200_c0_g1_i1_2	0.4893943	0.2242835
DN12202_c0_g1_i2_1	0.8579469	2.103019
DN12203_c0_g1_i1_1	5.278122	14.8621
DN12205_c0_g1_i1_2	86.43922	79.31458
DN12206_c0_g2_i1_2	0.4018976	1.478657
DN12207_c0_g1_i1_1	0.8663903	2.33469
DN12208_c0_g1_i1_1	1.301716	2.423251
DN12209_c0_g1_i1_1	1.090125	1.111676

DN1220_c0_g1_i1_1	0.1816899	0.3990139
DN12211_c0_g2_i1_1	1.261196	1.350413
DN12215_c0_g1_i1_1	1.804103	0.9882102
DN12216_c0_g2_i1_1	1.426634	0.9736426
DN12220_c0_g1_i2_1	1.264516	0.7124593
DN12223_c0_g1_i2_1	0.6709278	3.265757
DN12228_c0_g1_i1_1	0.07379089	2.852739
DN12231_c0_g1_i1_1	1.279096	1.579919
DN12233_c0_g1_i1_1	0.8550614	0.839649
DN12238_c0_g1_i1_1	0.7890167	1.075196
DN12238_c0_g1_i1_2	2.049505	0.7204567
DN12238_c0_g2_i1_2	1.522754	2.429467
DN12238_c0_g3_i1_2	2.529201	0.2174001
DN12239_c0_g1_i1_1	0.1696305	1.356464
DN12240_c0_g1_i1_2	1.467115	4.310268
DN12241_c0_g1_i1_1	0.9498393	0.4872091
DN12241_c0_g1_i1_2	2.70876	0.7931771
DN12245_c0_g1_i1_2	2.471124	0.8975349
DN12247_c0_g1_i1_1	0.6399597	4.367694
DN12250_c0_g1_i1_2	1.7106	0
DN12255_c0_g1_i1_1	0.3651166	2.507384
DN12255_c0_g1_i1_2	1.317753	0
DN12257_c0_g1_i1_1	3.660983	7.168216
DN12259_c0_g1_i1_1	32.85734	22.01396

DN12262_c0_g1_i1_1	9.345026	30.44548
DN12263_c0_g1_i1_1	1.038081	1.641621
DN12268_c0_g1_i1_2	1.591483	1.060069
DN12274_c0_g2_i1_1	1.232626	0.6430694
DN12277_c0_g1_i1_2	2.741986	1.529116
DN12277_c0_g2_i1_2	4.820179	2.114784
DN12283_c0_g1_i1_1	2.58887	3.12246
DN12286_c0_g1_i1_2	4.63425	1.273751
DN12287_c0_g2_i1_1	0.6581992	0.7202451
DN12288_c0_g1_i3_1	1.607626	0.7330343
DN12290_c0_g1_i3_2	6.595501	19.60144
DN12295_c0_g1_i1_1	1.935325	0.5109716
DN12298_c0_g1_i1_1	2.125275	3.109403
DN12298_c0_g1_i1_2	1.51008	2.412186
DN12300_c0_g1_i1_1	11.45576	3.670039
DN12303_c0_g1_i1_1	1.469938	2.375448
DN12304_c0_g2_i1_2	3.816366	0.8424167
DN12305_c0_g1_i1_2	3.083681	1.993294
DN12307_c0_g1_i1_1	1.053802	2.144332
DN1230_c0_g1_i1_1	2.588872	3.858913
DN12310_c0_g1_i1_1	0.3903761	1.534374
DN12311_c0_g1_i1_1	0.626788	1.570954
DN12320_c0_g1_i2_1	2.484844	1.065523
DN12321_c0_g1_i1_1	0.6803446	2.429439

DN12321_c0_g2_i1_1	2.176191	2.540961
DN12322_c0_g1_i1_1	4.381304	4.806027
DN12322_c0_g3_i1_1	1.445662	1.405483
DN12324_c0_g1_i1_1	7.550358	7.419217
DN12326_c0_g1_i1_2	29	28.38789
DN12328_c0_g1_i2_2	5.275092	3.651713
DN12337_c0_g1_i1_2	5.162591	28.96302
DN1233_c0_g1_i1_1	0.4408827	1.037265
DN12341_c0_g1_i1_1	1.487919	1.385787
DN12341_c0_g2_i1_1	2.045155	1.472274
DN12343_c1_g1_i1_1	0.2852551	0.3370259
DN12345_c0_g1_i1_2	1.41522	0.2185544
DN12347_c0_g1_i1_1	2.366457	2.531823
DN12347_c0_g1_i1_2	0	0
DN12347_c0_g2_i1_1	1.666526	2.914813
DN12348_c0_g3_i1_1	2.187076	2.107216
DN12352_c0_g2_i1_1	1.596474	0.4700123
DN12352_c0_g3_i1_1	2.097159	1.332886
DN12353_c0_g1_i1_1	0.3091393	2.114727
DN12355_c0_g1_i1_1	0.906477	0.8360131
DN12355_c0_g1_i1_2	2.060547	1.604754
DN12356_c0_g1_i2_1	19.0575	17.26301
DN12369_c0_g1_i1_2	4.514647	1.83219
DN12372_c0_g1_i1_1	0.1855559	1.790774

DN12372_c0_g1_i1_2	1.098841	2.703149
DN12373_c0_g1_i2_1	0.8404235	0.4563949
DN12378_c0_g1_i1_1	1.113911	2.158083
DN12378_c0_g1_i1_2	29.54332	0
DN12378_c0_g2_i1_1	0.8110918	1.564498
DN12379_c0_g1_i1_1	1.223298	1.101151
DN1237_c0_g1_i1_1	0.5669966	2.005804
DN12381_c0_g2_i1_2	4.505926	1.707733
DN12382_c0_g1_i1_1	0.6881189	1.560072
DN12383_c0_g1_i1_1	0.2541746	39.37433
DN12384_c0_g1_i1_2	13.96938	3.566881
DN12386_c0_g1_i1_1	0	2.585327
DN12389_c0_g1_i1_1	1.177973	1.948192
DN12394_c0_g1_i1_1	2.129817	4.40429
DN12397_c0_g1_i1_1	150.1186	104.8981
DN123_c0_g1_i1_1	1.683602	3.012607
DN12406_c0_g1_i1_2	2.208901	0
DN12406_c0_g2_i1_2	2.899305	0.9178281
DN12406_c0_g3_i1_2	1.532228	0.1651687
DN12407_c0_g1_i1_1	0.5393336	1.343074
DN12407_c0_g2_i2_1	1.007228	0.9568621
DN12409_c0_g1_i1_1	2.180178	4.376701
DN12410_c0_g1_i1_1	1.957762	4.228412
DN12414_c0_g1_i1_2	4.361938	5.007597

DN12415_c0_g1_i1_1	5.453331	3.297688
DN12421_c0_g1_i1_1	1.241368	3.866561
DN12422_c0_g2_i1_1	14.52559	7.462367
DN12424_c0_g1_i1_1	1.500784	1.329146
DN12426_c0_g2_i1_2	3.785845	4.564871
DN12428_c0_g1_i1_2	1.891373	38.96136
DN12430_c0_g1_i1_2	2.250197	0.575657
DN12434_c0_g1_i2_1	1.225895	1.265074
DN12436_c0_g2_i1_1	1.088754	1.959995
DN12441_c0_g1_i1_2	12.46879	4.447894
DN12442_c0_g1_i1_2	1.310476	4.496853
DN12444_c0_g1_i1_1	12.46576	3.950241
DN12449_c0_g1_i1_2	1.24662	0.3662068
DN12450_c0_g1_i1_1	0.8665792	0.3023745
DN12450_c0_g2_i1_1	1.458272	1.083452
DN12451_c0_g1_i1_1	1.824913	2.387155
DN12451_c0_g1_i1_2	0.5200112	1.064185
DN12454_c0_g1_i1_1	1.351891	2.410662
DN12455_c0_g1_i1_1	2.306978	2.4347
DN12457_c0_g1_i1_1	2.759729	1.86414
DN12459_c0_g1_i1_1	1.361674	1.405617
DN12462_c0_g1_i1_1	1.880084	0.2429655
DN12462_c0_g1_i1_2	45.23909	9.164472
DN12463_c0_g1_i1_1	1.155713	2.66831

DN12468_c0_g1_i1_2	4.479435	1.886872
DN12471_c0_g1_i1_2	2.307257	1.549452
DN12475_c0_g1_i1_1	3.803897	2.911906
DN12476_c0_g1_i1_1	15.01865	33.5885
DN1247_c0_g1_i1_2	0	0
DN12481_c0_g1_i1_1	1.662731	3.383911
DN12481_c0_g1_i1_2	2.537538	1.848874
DN12484_c0_g1_i1_1	1.512282	1.233266
DN12485_c0_g1_i1_2	2.921547	0.5588631
DN1248_c0_g1_i1_1	0.3622223	1.954306
DN12491_c0_g1_i1_1	2.066772	0.8616239
DN12491_c0_g1_i1_2	3.639679	0.1426935
DN12492_c0_g2_i1_2	10.51949	6.219125
DN12495_c0_g1_i1_2	0.8856421	0.2042824
DN12496_c0_g1_i1_1	1.303553	5.304657
DN12500_c0_g1_i1_1	2.500518	0.7581548
DN12501_c0_g1_i1_1	1.742204	1.654615
DN12502_c0_g1_i1_1	2.554895	9.546817
DN12502_c0_g1_i1_2	19.65035	5.620456
DN12506_c0_g1_i1_2	0	0.2437321
DN12510_c0_g2_i1_1	0.316806	0.5202661
DN12511_c0_g1_i1_1	1.134628	2.913725
DN12514_c0_g1_i1_1	1.828305	1.87306
DN12515_c0_g1_i1_1	2.706908	3.324685

DN12516_c0_g1_i1_2	4.504346	3.804195
DN12519_c0_g1_i1_2	1.560066	42.3718
DN12520_c0_g2_i1_1	2.205676	4.117196
DN12522_c0_g1_i1_1	2.614124	1.883
DN12526_c0_g1_i1_1	3.679682	5.721605
DN12529_c0_g1_i1_1	1.735763	7.170635
DN12535_c0_g1_i2_1	0.2001628	1.390034
DN12536_c0_g1_i1_1	4.350598	2.356659
DN12536_c0_g2_i1_1	0.5840742	1.75878
DN12536_c0_g2_i1_2	0.291666	0.6439441
DN12546_c0_g1_i1_1	0.4400406	0.3496129
DN12546_c0_g1_i2_2	2.193135	1.290563
DN12547_c0_g1_i1_1	4.783696	3.328011
DN12553_c0_g1_i1_2	1.97185	0.8662877
DN12556_c0_g1_i1_1	1.216265	1.434278
DN12560_c0_g1_i1_1	1.696831	3.493806
DN1256_c0_g1_i1_1	0.9132638	0.2486433
DN12574_c0_g1_i1_1	1.006931	3.588901
DN12574_c0_g1_i1_2	7.755237	11.54768
DN12576_c0_g1_i1_1	8.03585	3.169059
DN12580_c0_g1_i1_1	0.4279764	0.3979291
DN12582_c1_g1_i1_1	1.708633	0
DN12585_c0_g1_i2_2	1.394316	1.195205
DN12588_c0_g1_i2_1	23.80309	19.46293

DN12589_c0_g1_i1_1	0.8615654	7.063136
DN12589_c0_g2_i1_1	1.090032	2.875281
DN12596_c0_g2_i1_2	1.514244	0.7942396
DN12600_c0_g2_i1_1	3.020959	2.197845
DN12601_c0_g1_i1_1	0.8569996	0.3289895
DN12602_c0_g1_i1_1	3.053012	4.403562
DN12604_c0_g1_i1_1	2.065797	3.978615
DN12606_c0_g1_i1_1	1.635976	2.023858
DN1260_c0_g1_i1_1	0.4684265	1.01117
DN1260_c0_g1_i1_2	62.06243	17.85559
DN12611_c0_g1_i1_2	16.07636	7.127821
DN12613_c0_g1_i1_2	3.458476	3.828698
DN12619_c0_g1_i2_1	1.049167	3.848277
DN12621_c0_g1_i1_1	0.9629338	0.9611406
DN12621_c0_g1_i1_2	4.408817	4.782168
DN12621_c0_g2_i1_2	4.875497	4.582417
DN12623_c0_g1_i1_1	2.737155	4.612766
DN12625_c0_g1_i1_1	2.915106	10.24424
DN12626_c0_g1_i1_2	2.201351	2.185778
DN12627_c0_g1_i1_1	1.705177	1.819187
DN12629_c0_g1_i1_1	1.002187	0.7810394
DN12629_c0_g2_i1_1	0.9503815	0.9104882
DN12630_c0_g1_i2_1	0.9926951	1.391615
DN12631_c0_g1_i1_1	0.4216055	4.139992

DN12631_c0_g1_i1_2	2.084276	0.7723719
DN12631_c0_g2_i1_2	2.15796	0.7418
DN12631_c0_g2_i2_1	2.568416	2.594955
DN12633_c0_g1_i1_1	4.672341	2.46916
DN12635_c0_g1_i1_1	0.4299786	0.5117762
DN12639_c0_g1_i1_2	1.537826	2.195563
DN1263_c0_g1_i1_2	0.2989467	0
DN12643_c0_g1_i1_1	1.12245	0.6813572
DN12645_c0_g1_i2_1	84.98569	59.6738
DN12648_c0_g1_i1_2	1.428616	1.103369
DN12651_c0_g1_i1_2	1.585614	0.605549
DN12652_c0_g1_i1_1	1.267342	0.4630147
DN12658_c0_g1_i1_2	0	0
DN12661_c0_g1_i1_1	1.828506	87.37429
DN12662_c0_g1_i1_2	8.023184	6.016245
DN12665_c0_g1_i2_1	2.369897	2.970593
DN12666_c0_g1_i2_1	45.34036	44.85146
DN12666_c0_g1_i2_2	6.639356	3.47991
DN12667_c0_g1_i1_1	0.7308526	1.74996
DN12671_c0_g1_i1_1	2.427492	1.150609
DN12673_c0_g1_i1_1	11.41918	14.82971
DN12676_c0_g1_i1_1	38.50923	20.47034
DN12677_c0_g1_i2_1	1.017743	3.832955
DN1267_c0_g1_i1_2	1.504419	0.209507

DN12681_c0_g1_i1_1	3.082961	6.156529
DN12681_c0_g1_i1_2	0.2605617	0.3476319
DN12686_c0_g1_i1_1	0.5613612	3.886374
DN12688_c0_g1_i1_1	7.072278	6.637174
DN12689_c0_g1_i2_1	2.338001	2.521603
DN12698_c0_g1_i1_1	0.5358895	1.468535
DN12698_c0_g1_i1_2	6.069865	1.741867
DN12699_c0_g1_i1_1	4.669206	8.015444
DN126_c0_g1_i1_2	0.6170776	1.277359
DN12703_c0_g1_i1_1	6.00658	8.795549
DN12706_c0_g1_i1_1	3.032521	4.477437
DN12706_c0_g2_i1_1	2.523028	3.244437
DN12708_c0_g1_i1_1	3.447366	5.985657
DN12709_c1_g1_i1_2	1.895351	1.220519
DN12711_c0_g1_i1_1	0.7260763	0.7255502
DN12713_c0_g1_i1_2	2.765152	1.172662
DN12714_c0_g1_i1_1	1.670177	2.300963
DN12722_c0_g1_i2_1	0	11.16447
DN12725_c0_g1_i1_1	0.6000087	3.673653
DN12725_c0_g1_i2_2	0.7317928	4.029036
DN12726_c0_g1_i1_1	0.1669069	1.833098
DN12726_c0_g2_i1_1	0.8804699	0.4756074
DN12728_c0_g1_i1_1	4.05322	3.694972
DN12728_c0_g1_i1_2	1.311346	0.7340102

DN12729_c0_g2_i1_1	1.410597	4.321541
DN1272_c0_g1_i1_1	1.052571	0.6687369
DN1272_c0_g1_i1_2	1.92128	1.43041
DN12734_c0_g1_i1_1	1.007416	1.256327
DN12734_c0_g2_i1_1	1.364716	1.237742
DN12737_c0_g1_i1_1	1.211124	2.914196
DN12738_c0_g1_i1_2	1.401199	1.839537
DN12744_c0_g1_i1_1	1.493864	5.313008
DN12746_c0_g1_i1_1	0.8547416	1.990566
DN12748_c0_g1_i2_1	8.287941	120.0372
DN12749_c0_g1_i1_1	2.419005	7.958015
DN12757_c0_g1_i1_1	0.8380349	0.724191
DN12759_c0_g1_i1_1	0.5465475	2.681976
DN12759_c0_g1_i1_2	3.119929	3.489881
DN12761_c0_g1_i4_1	1.275805	1.043914
DN12762_c0_g1_i1_1	15.18579	15.48538
DN12763_c0_g1_i1_1	0.5520805	1.84422
DN12764_c0_g1_i1_1	3.467224	6.908698
DN12764_c0_g1_i1_2	8.049559	10.92943
DN12769_c0_g1_i1_1	0.7594276	5.71117
DN12773_c0_g1_i1_2	4.574322	0.1985886
DN12774_c0_g1_i1_1	1.081842	5.936416
DN12774_c0_g2_i1_1	5.737264	9.304324
DN12776_c0_g1_i1_1	4.179701	84.53349

DN12781_c0_g1_i1_2	6.185668	3.250219
DN12782_c0_g1_i1_2	1.822898	1.417186
DN12784_c0_g1_i1_1	2.037236	1.92E-68
DN12784_c0_g1_i1_2	2.698859	1.320193
DN12784_c0_g2_i1_1	1.687906	9.20659
DN12787_c0_g1_i1_1	2.556865	1.228761
DN12789_c0_g1_i1_2	1.968252	1.154753
DN12792_c0_g1_i3_1	1.741677	1.104801
DN12795_c0_g1_i1_2	1.155367	5.81E-28
DN12797_c0_g1_i1_1	2.068834	1.924706
DN12797_c0_g1_i1_2	4.968512	0.5306098
DN12799_c0_g1_i1_1	1.811121	2.955891
DN1279_c0_g1_i1_1	7.887093	3.237824
DN12800_c0_g1_i1_2	2.058578	1.06784
DN12803_c0_g1_i1_1	0.3102198	2.451633
DN12805_c0_g1_i1_2	2.307499	0.6587783
DN12813_c0_g1_i1_1	7.604254	2.810076
DN12814_c0_g1_i1_2	0.7265234	1.517522
DN12817_c0_g1_i1_1	0.5170735	0.5984255
DN12819_c0_g1_i1_1	3.352075	2.240719
DN12819_c0_g1_i1_2	2.250221	1.113584
DN12821_c0_g1_i2_2	5.62581	0.3966212
DN12822_c0_g1_i1_2	2.835074	1.275936
DN12825_c0_g1_i1_2	5.44015	2.599761

DN12831_c0_g1_i1_1	0.332451	0.9654886
DN12833_c0_g1_i1_1	1.152993	11.37055
DN12834_c0_g1_i1_1	19.80937	32.64961
DN12838_c0_g1_i1_1	0.6335775	0.8601654
DN12839_c0_g1_i1_1	0.8125223	0.962268
DN12842_c0_g1_i1_2	0.1819981	3.41E-06
DN12846_c0_g1_i1_2	1.564776	1.136755
DN12848_c0_g1_i1_2	1.353857	0.1136328
DN12849_c0_g1_i1_2	6.219861	3.65933
DN12850_c0_g1_i1_1	0.3550103	0.7380421
DN12853_c0_g1_i1_1	0.6317325	2.416882
DN12856_c0_g1_i1_2	1.3112	0.7779079
DN1285_c0_g1_i1_1	0	0.8021489
DN12860_c0_g1_i1_1	0.5986728	0.4164518
DN12865_c0_g1_i1_1	6.303115	8.381034
DN12867_c0_g1_i2_2	10.53613	7.603769
DN12868_c0_g1_i1_1	1.017188	2.803369
DN12868_c0_g1_i1_2	0	0
DN12869_c0_g1_i1_1	1.904309	5.00342
DN12870_c0_g1_i1_1	5.34868	3.287971
DN12872_c1_g1_i1_1	1.40594	1.725926
DN12872_c1_g2_i1_1	4.884312	2.026729
DN12874_c0_g1_i1_1	1.256916	0.3959992
DN12876_c0_g1_i1_1	0.8872257	1.505382

DN12877_c0_g1_i1_1	8.335725	19.58117
DN12883_c0_g1_i1_1	1.937832	2.903346
DN12883_c0_g1_i1_2	2.731496	1.738042
DN12887_c0_g1_i1_1	2.057356	1.852127
DN12887_c0_g1_i1_2	108.0644	286.6254
DN12892_c0_g1_i1_1	2.602079	3.552898
DN12892_c0_g1_i1_2	4.038888	3.616063
DN12893_c0_g1_i1_2	5.937737	2.105755
DN12896_c0_g1_i1_1	2.569873	1.962906
DN12896_c0_g1_i1_2	1.429451	1.001834
DN12896_c0_g2_i1_2	35.93946	34.56855
DN12900_c0_g1_i1_2	61.73473	55.36834
DN12906_c0_g1_i1_1	28.14576	27.905
DN12912_c0_g1_i1_2	0.5143165	0.1403213
DN12914_c0_g1_i1_1	1.27764	1.9427
DN12914_c0_g1_i1_2	0.9327143	1.887077
DN12916_c0_g1_i1_2	1.781017	0
DN12917_c0_g1_i1_1	0.6834226	2.545636
DN12917_c0_g1_i1_2	3.37846	1.406576
DN12917_c0_g2_i1_1	0.8715932	2.806631
DN12917_c0_g3_i1_1	0.284276	0.9975897
DN12917_c0_g4_i1_1	0.9597787	1.614239
DN12918_c0_g1_i1_1	0.826519	1.245452
DN12921_c0_g1_i1_1	0.2556248	0.8070743

DN12922_c0_g1_i1_1	1.742616	2.043918
DN12927_c0_g1_i1_2	5.286072	1.426554
DN12927_c0_g2_i2_1	7.368965	3.19153
DN1292_c0_g1_i1_2	5.612676	2.428245
DN12930_c0_g1_i1_1	0.5408067	1.166681
DN12932_c0_g1_i1_1	7.652104	3.02659
DN12933_c0_g2_i1_1	0.4344758	5.227718
DN12935_c0_g1_i1_1	0.3019399	2.117614
DN12935_c0_g1_i1_2	1.630716	0.936172
DN12936_c0_g1_i1_2	22.06675	17.36424
DN12937_c0_g1_i1_1	2.611167	4.829399
DN1293_c0_g1_i1_1	10.04467	5.996696
DN1293_c0_g1_i1_2	0	0
DN12943_c0_g1_i1_1	8.774306	11.21406
DN12945_c0_g1_i2_2	3.34508	4.685451
DN12946_c0_g1_i2_1	3.995617	11.17798
DN12947_c0_g1_i1_1	7.16739	5.328048
DN12948_c0_g1_i8_1	11.66152	33.30725
DN12949_c0_g2_i1_2	1.60946	2.573391
DN12951_c0_g1_i1_1	1.928106	2.154614
DN12951_c0_g1_i1_2	2.639589	1.153426
DN12952_c0_g1_i1_2	11.36302	11.37025
DN12954_c0_g1_i1_2	0.8505089	0.2936465
DN12954_c0_g2_i1_2	0.8930333	0.8117355

DN12955_c0_g1_i1_1	1.764366	2.158304
DN12957_c0_g1_i1_2	12.11252	19.66697
DN12958_c0_g1_i1_1	0.2301476	1.297688
DN12958_c0_g2_i1_1	0.2652192	0.3602513
DN1295_c0_g1_i1_1	0.1769236	0.4042762
DN12960_c0_g1_i2_2	1.749764	1.03858
DN12961_c0_g1_i1_1	0.2723336	1.633634
DN12965_c0_g1_i1_1	6.548406	12.25009
DN12967_c0_g1_i1_1	1.151058	2.183269
DN1296_c0_g1_i1_1	0.4231408	3.94336
DN12972_c0_g1_i1_1	0.1192473	3.351367
DN12973_c0_g1_i1_1	6.392294	6.018578
DN12973_c0_g2_i2_1	1.471272	3.351825
DN12977_c0_g1_i1_2	1.006912	0.7662559
DN12983_c0_g1_i1_1	0.3865983	2.617878
DN12983_c0_g1_i2_2	2.09264	0.8598123
DN12983_c0_g2_i1_1	0.7197548	5.417308
DN12985_c0_g1_i1_1	0.225176	0.6927216
DN12985_c0_g1_i1_2	2.431486	1.028663
DN1298_c0_g1_i1_1	2.155021	7.940616
DN12991_c0_g1_i1_1	0.1389668	2.955197
DN12991_c0_g1_i2_2	64.67323	41.00332
DN12991_c0_g2_i1_1	0.1888989	1.774927
DN12992_c0_g1_i2_2	3.515328	0.8282016

DN12993_c0_g1_i1_1	1.529804	2.585467
DN12994_c0_g1_i1_1	1.141607	2.716157
DN12994_c0_g1_i1_2	11.16299	4.638533
DN12997_c0_g1_i1_1	3.614859	12.78688
DN12997_c0_g1_i1_2	3.436598	1.243264
DN12998_c0_g1_i1_2	331.1717	404.4041
DN1299_c0_g1_i1_1	0.2535956	0.2740367
DN12_c0_g1_i1_1	1.0586	0.3909625
DN13001_c0_g1_i1_2	8.011912	3.365843
DN13003_c0_g1_i1_1	3.359524	16.26018
DN13005_c0_g1_i1_1	1.059725	1.17909
DN13005_c0_g1_i1_2	0	0
DN13005_c0_g2_i1_1	0.8400128	1.273697
DN13006_c0_g1_i1_2	3.072106	1.155343
DN13008_c0_g1_i1_1	0.9231272	1.143812
DN13009_c0_g1_i1_1	0.725388	3.73311
DN13009_c0_g1_i1_2	1.992085	1.12415
DN13012_c0_g1_i1_1	5.750221	6.771012
DN13013_c0_g1_i2_1	3.904183	4.119039
DN13019_c0_g1_i1_2	5.567815	1.896554
DN13021_c0_g1_i1_1	1.235293	2.275019
DN13021_c0_g2_i1_1	0.8706935	0.808491
DN13022_c0_g1_i1_1	0.7312575	0.7490695
DN13022_c0_g1_i1_2	0.9587531	0

DN13023_c0_g1_i1_2	0.8529471	0.5295643
DN13026_c0_g1_i1_1	1.614122	0
DN13028_c0_g1_i1_2	1.516878	0.2518163
DN13030_c0_g1_i1_2	2.400186	0.6825556
DN13030_c0_g1_i2_1	5.413655	7.879388
DN13034_c0_g1_i1_1	2.920907	2.908797
DN1303_c0_g1_i1_1	0.1283104	1.844103
DN13041_c0_g2_i1_2	0.1961693	2.03E-47
DN13045_c0_g1_i1_1	3.610101	0.5147245
DN13045_c0_g2_i1_1	0.6548489	2.854694
DN13045_c1_g1_i2_1	1.538712	1.098147
DN13047_c0_g1_i1_1	4.320367	7.321179
DN13048_c0_g1_i1_2	0.2252657	0.2700765
DN13049_c0_g1_i1_1	0.2873841	0.3305478
DN13052_c0_g1_i1_1	1.507807	2.806758
DN13057_c0_g2_i1_1	0.3423594	2.466483
DN13058_c0_g1_i1_1	0.4636421	2.096354
DN13059_c0_g1_i1_2	1.024966	0.7171131
DN13064_c0_g1_i1_1	0.3571592	0.7571023
DN13069_c0_g1_i1_1	0.7724599	1.922931
DN13069_c0_g2_i1_1	0.2144954	1.001579
DN13069_c0_g3_i1_1	0.4896351	2.056171
DN13070_c0_g1_i1_1	6.616697	7.407643
DN13073_c0_g2_i1_1	0.951472	1.233808

DN13076_c0_g1_i1_2	1.082334	0.7271446
DN13078_c0_g1_i1_1	2.162388	2.269699
DN1307_c0_g1_i1_1	16.20229	21.23505
DN13083_c0_g1_i1_1	1.341298	1.884368
DN13084_c0_g1_i1_2	1.786064	0
DN13086_c0_g1_i1_2	4.056758	0.2789308
DN13090_c0_g2_i1_2	5.85464	2.974604
DN13091_c0_g1_i1_1	3.228611	4.154298
DN13093_c0_g1_i1_1	0.5611231	1.399394
DN13095_c0_g1_i2_1	5.050555	10.42145
DN13097_c0_g1_i1_1	0.4142321	0.8828122
DN13099_c0_g1_i1_1	0.4027794	0.8912543
DN1309_c0_g1_i1_1	2.214303	0.6862059
DN13100_c0_g1_i1_2	1.725305	0.5321005
DN13102_c0_g1_i1_1	0.9680462	0.5080893
DN13102_c0_g2_i1_1	1.002658	1.46233
DN13103_c0_g1_i1_1	1.569186	1.044311
DN13105_c0_g1_i1_2	0.7732735	1.177636
DN13116_c0_g1_i1_2	8.300214	19.21679
DN13118_c0_g1_i1_1	1.369933	1.771612
DN13122_c0_g1_i1_2	1.134981	1.578603
DN13123_c0_g1_i1_1	0.8177891	1.191036
DN13125_c0_g1_i1_1	2.799406	4.368428
DN13127_c0_g1_i1_1	0.3904932	0.8531621

DN1312_c0_g1_i1_1	5.292605	3.35468
DN1312_c0_g1_i2_2	7.035498	2.045728
DN13131_c0_g1_i1_1	0.5540742	0.878643
DN13133_c0_g1_i1_2	2.123298	0.213244
DN13138_c0_g1_i1_1	0.9283821	2.970975
DN13139_c0_g1_i1_1	1.070803	0.663581
DN13145_c0_g1_i1_1	0.9735297	1.673373
DN13146_c0_g1_i1_2	0.7477131	1.233247
DN13149_c0_g1_i1_1	2.501667	4.264568
DN13150_c0_g1_i1_2	1.513423	1.33E-11
DN13151_c0_g1_i1_1	2.49132	3.68548
DN13152_c0_g1_i1_1	4.296762	3.14223
DN13153_c0_g2_i1_1	0.5982626	1.627849
DN13155_c0_g1_i1_2	1.369458	1.369687
DN13157_c0_g1_i2_1	0.5300834	0.8797673
DN13158_c0_g1_i1_2	3.074301	2.813229
DN13159_c0_g1_i1_2	0.3061039	1.014304
DN1315_c0_g1_i1_1	0.7102559	0.5510374
DN13160_c0_g1_i1_1	1.434351	2.639506
DN13165_c0_g1_i1_2	1.393108	0.1580884
DN13167_c0_g1_i1_2	2.130391	0.736687
DN13170_c1_g2_i1_1	0.2506987	3.41E-12
DN13172_c0_g2_i1_1	1.122432	1.876087
DN13172_c0_g3_i1_1	1.948679	2.748815

DN13174_c0_g1_i1_1	2.042322	0.732409
DN13174_c0_g1_i1_2	5.81659	5.397475
DN13175_c0_g1_i1_1	0.597056	3.452339
DN13175_c0_g1_i1_2	0.9624065	1.53387
DN13175_c0_g2_i1_1	1.030063	1.235073
DN13176_c0_g1_i1_1	6.342014	12.87036
DN13176_c0_g1_i2_2	38.21615	45.72491
DN13182_c0_g1_i1_1	0.9780445	1.132605
DN13182_c0_g1_i1_2	2.906961	0.8638101
DN13186_c0_g1_i2_1	1.843474	2.393681
DN13189_c0_g1_i2_2	0.8861358	0.56413
DN13192_c0_g2_i1_1	1.809577	0.9770838
DN13193_c0_g1_i1_1	2.534927	2.043151
DN13194_c0_g2_i1_2	2.677095	1.043732
DN13195_c0_g1_i1_1	0.6882219	0.6163654
DN13198_c0_g1_i1_1	1.711543	2.351397
DN13201_c0_g1_i1_1	0.789862	1.13228
DN13204_c0_g1_i2_2	2.8193	5.852259
DN13207_c0_g1_i1_1	2.044956	0.4956283
DN13211_c0_g1_i1_1	5.741348	6.406962
DN13211_c0_g1_i1_2	74.21802	137.0515
DN13212_c0_g1_i1_1	1.423449	1.958682
DN13212_c0_g1_i1_2	4.745584	2.455242
DN13213_c0_g1_i1_1	0.3254226	0.4164017

DN13217_c0_g1_i1_1	0.7957138	1.180099
DN13218_c0_g1_i1_2	4.179286	1.583272
DN13219_c0_g1_i1_1	4.232167	1.609765
DN1321_c0_g1_i1_2	0.186799	1.097293
DN13220_c0_g1_i1_2	0.9335447	2.028658
DN13221_c0_g1_i1_2	2.889393	0.6299509
DN13223_c0_g1_i1_2	1.921719	4.062328
DN13226_c0_g1_i1_2	2.099701	0.7432114
DN13235_c0_g1_i1_2	2.165921	0.2783379
DN13235_c0_g1_i2_1	1.274466	3.016347
DN13239_c0_g1_i1_1	0.1752164	1.733343
DN13239_c0_g2_i1_1	0.1198532	1.20659
DN13242_c0_g1_i2_1	2.77307	4.733246
DN13248_c0_g1_i1_1	1.234389	2.517382
DN13248_c0_g2_i1_1	1.528109	1.584186
DN13252_c0_g2_i1_1	0.7676369	0.8913936
DN13253_c0_g1_i1_1	0	1.801636
DN13253_c0_g2_i1_1	0	3.319141
DN13254_c0_g1_i1_2	0	0
DN13257_c0_g1_i2_1	2.182917	2.093674
DN13261_c0_g1_i1_2	0	0
DN13265_c0_g1_i1_2	2.791374	1.731624
DN13269_c0_g1_i1_1	1.788583	1.818759
DN1326_c0_g1_i1_1	0	0.3952943

DN13271_c0_g1_i1_1	5.242976	7.054456
DN13272_c0_g1_i1_1	2.086379	4.996671
DN13273_c0_g1_i1_2	6.896579	1.022848
DN13274_c0_g1_i1_2	6.942255	6.329096
DN13275_c0_g4_i1_1	7.729912	2.524184
DN13277_c0_g1_i1_1	0.8290226	0.7503052
DN13279_c0_g1_i1_2	4.06154	1.653574
DN13281_c0_g1_i1_2	0	0
DN13286_c0_g1_i1_1	1.180964	0.7721344
DN13288_c0_g2_i1_1	5.238837	7.918177
DN1328_c0_g1_i1_1	0.2573867	1.995643
DN13291_c0_g2_i1_1	1.727997	1.962478
DN13296_c0_g1_i6_1	18.50127	33.26494
DN13300_c0_g1_i1_1	0	4.373904
DN13300_c0_g1_i1_2	3.921907	2.329527
DN13303_c0_g1_i1_1	6.248112	37.58166
DN13304_c0_g1_i1_2	5.377601	5.062939
DN13305_c0_g1_i1_1	8.890658	12.18458
DN13305_c0_g1_i1_2	6.430064	9.675509
DN13308_c0_g1_i1_2	198.5459	139.1503
DN13316_c0_g1_i1_1	2.567779	1.564305
DN13323_c0_g1_i2_2	2.106226	2.111641
DN13325_c0_g1_i1_1	1.579487	1.86934
DN13326_c0_g1_i1_2	2.423455	0

DN13328_c0_g2_i1_1	1.267488	1.523544
DN13330_c0_g1_i2_1	1.119329	1.672628
DN13331_c0_g1_i1_1	5.439885	4.512507
DN13332_c0_g1_i1_2	746.294	0.1986779
DN13334_c0_g1_i1_2	0.5847988	0.1919376
DN13334_c0_g2_i1_2	1.212174	0.5246762
DN13336_c0_g1_i2_1	0.3887883	1.702387
DN13337_c0_g1_i1_2	0.5737128	0.9269992
DN13339_c0_g1_i1_1	0.6075255	0.8689962
DN13342_c0_g1_i1_2	15.27683	11.52413
DN13343_c0_g1_i1_1	2.111531	1.746702
DN13344_c0_g1_i1_1	2.8478	5.988931
DN13344_c0_g1_i1_2	1.063966	0.7842085
DN13345_c0_g1_i1_2	3.065335	1.257549
DN13346_c0_g1_i1_1	0.943995	0.5342832
DN13353_c0_g1_i1_1	0.366134	0.7012574
DN13353_c0_g2_i1_1	0.8644134	2.556842
DN13354_c0_g1_i1_2	2.995183	2.121718
DN13355_c0_g1_i1_2	0.742621	0.4522054
DN13355_c0_g2_i1_2	1.25877	0.2181509
DN13367_c0_g1_i1_1	3.15264	2.532631
DN13369_c0_g1_i1_2	3.880279	2.143466
DN1336_c0_g1_i1_2	1.094386	0.2720549
DN13373_c0_g1_i1_1	4.453509	5.854261

DN13374_c0_g1_i1_1	0.6097062	3.231811
DN13374_c0_g1_i1_2	1.688888	0.3543772
DN13375_c0_g1_i1_2	2.462373	0.1492507
DN13377_c0_g1_i1_1	0.9709531	3.023531
DN1337_c0_g1_i1_1	2.743199	3.150842
DN13383_c0_g1_i1_2	25.46822	8.171344
DN13384_c0_g1_i1_1	0.6291279	3.297356
DN13386_c0_g1_i1_1	0.7608315	0.9534765
DN13387_c0_g1_i1_2	17.2943	9.064407
DN13389_c0_g1_i1_1	1.5885	5.826641
DN1338_c0_g1_i1_2	1.349907	0
DN13390_c0_g1_i1_2	0	0
DN13392_c0_g1_i1_2	4.451219	6.407893
DN13393_c0_g1_i1_2	0.3260411	0.1898788
DN13396_c0_g1_i2_1	1.512995	1.333668
DN13398_c0_g1_i3_1	0.7233862	1.184746
DN13399_c0_g1_i2_1	19.24632	36.82536
DN133_c0_g1_i1_2	0.9584178	1.781771
DN13400_c0_g1_i2_1	0.5689868	0.4702987
DN13400_c0_g2_i1_1	1.111114	0.8087092
DN13403_c0_g1_i1_2	0.5162385	1.26769
DN13404_c1_g1_i1_1	0.4288708	1.464065
DN13407_c0_g1_i1_1	0.3471463	3.550821
DN13409_c0_g1_i1_2	6.280926	2.029541

DN13413_c0_g1_i1_2	4.575191	1.584941
DN13415_c0_g1_i1_2	5.78213	0.4920001
DN13417_c0_g1_i1_1	5.424303	7.67932
DN13421_c0_g1_i1_2	0.3650306	0.3692415
DN13424_c0_g1_i1_1	12.56875	14.91391
DN13428_c0_g1_i1_2	1.200278	1.098627
DN13428_c0_g1_i2_1	0.9740972	0.4310252
DN13432_c0_g1_i1_2	1.016047	1.188193
DN13432_c0_g2_i1_2	1.746717	0.4076211
DN13433_c0_g1_i1_2	1.19334	0.05272391
DN13433_c0_g1_i2_1	1.452835	0.914594
DN13433_c0_g2_i2_1	0.7996338	3.525302
DN13435_c0_g1_i1_2	1.663371	1.773708
DN13436_c0_g1_i1_1	7.457336	8.721135
DN13438_c0_g1_i1_1	11.70204	6.41245
DN13441_c0_g1_i1_2	15.66316	8.927067
DN13444_c0_g1_i1_2	1.386689	0.5321756
DN13446_c0_g1_i2_2	1.03637	0.6760543
DN13455_c0_g1_i1_2	2.994013	1.867834
DN13457_c0_g1_i1_1	2.087394	5.899573
DN13460_c0_g1_i1_1	1.139899	2.23854
DN13463_c0_g1_i1_1	1.987456	2.72712
DN13464_c0_g1_i1_2	6.465326	5.228038
DN13465_c0_g1_i1_1	1.089934	1.942382

DN13469_c0_g1_i1_2	2.599885	0.1561179
DN1346_c0_g1_i1_2	1.058579	4.192255
DN13473_c0_g1_i1_1	2.678983	7.31079
DN13474_c0_g1_i1_1	0.8085651	1.143776
DN13474_c0_g1_i1_2	1.172495	1.299247
DN13475_c0_g1_i1_1	1.530343	1.79709
DN13475_c0_g1_i1_2	46.90407	51.86735
DN13479_c0_g1_i3_1	4.16233	2.512615
DN13480_c0_g1_i1_1	1.669185	5.885309
DN13481_c0_g1_i1_1	1.413738	1.390225
DN13481_c0_g2_i1_1	1.22883	1.656435
DN13483_c0_g2_i1_2	2.827087	1.416752
DN13485_c0_g1_i1_2	2.718019	1.51572
DN13488_c0_g1_i1_2	2.56242	0.7068333
DN13492_c0_g1_i1_1	2.049332	3.651075
DN13493_c0_g1_i1_1	1.360518	1.964438
DN13494_c0_g1_i1_1	2.054104	1.977854
DN13497_c0_g1_i1_2	1.285307	0.1279924
DN13510_c0_g1_i1_1	8.68371	5.968011
DN13512_c0_g1_i2_2	29.11728	83.33891
DN13513_c0_g1_i1_1	2.31343	1.080699
DN13515_c0_g1_i2_2	3.951081	0.3000983
DN13517_c0_g1_i1_2	0	0
DN13520_c0_g1_i1_2	1.504309	0.1145751

DN13521_c0_g1_i1_1	3.684576	5.481807
DN13522_c0_g1_i1_1	2.574078	5.266773
DN13524_c0_g1_i1_1	0.5746289	8.958706
DN13525_c0_g1_i1_1	5.355527	7.13368
DN13527_c0_g1_i1_1	2.893263	4.134202
DN13528_c0_g1_i1_2	4.575854	0.4031561
DN13531_c0_g1_i1_2	2.937105	0.7335348
DN13532_c0_g1_i1_2	0.6310366	0.3250357
DN13532_c0_g2_i1_2	1.220448	0.2716852
DN13534_c0_g1_i1_2	0	0
DN13538_c0_g1_i1_1	0.5796476	3.853313
DN13539_c0_g1_i3_1	0.6905044	1.335585
DN13540_c0_g1_i2_2	1.50553	0
DN13543_c0_g1_i1_1	1.535423	3.171055
DN13550_c0_g1_i1_1	1.070978	0.8594879
DN13550_c0_g2_i1_1	1.132189	1.101425
DN13550_c0_g3_i1_1	1.313427	3.206281
DN13551_c0_g1_i1_1	0.9585049	0.5881846
DN13552_c0_g2_i1_2	1.050704	1.455331
DN13553_c0_g1_i1_2	1.645287	1.235839
DN13553_c0_g2_i1_2	0.6362936	1.355575
DN13556_c0_g2_i1_1	3.256485	3.20725
DN13557_c0_g1_i2_1	0.5564839	0.3674779
DN13559_c0_g1_i1_2	10.9999	1.100117

DN13560_c0_g1_i1_1	10.81301	6.927801
DN13564_c0_g1_i1_1	0.8279423	2.20307
DN13566_c0_g1_i1_2	7.044795	2.806331
DN13569_c0_g2_i1_2	2.197556	0.646032
DN13572_c0_g1_i1_1	1.232437	1.035251
DN13573_c0_g1_i1_2	0.2627795	1.377532
DN13575_c0_g1_i1_1	1.863906	1.50797
DN13577_c0_g1_i1_2	1.953315	0.8258188
DN13580_c0_g1_i1_1	1.549564	3.581604
DN13582_c0_g1_i1_2	0.8881786	0.5303334
DN13586_c0_g1_i1_1	20.89676	11.38589
DN13591_c0_g2_i1_1	1.2034	1.864274
DN13595_c0_g1_i3_1	2.976111	11.19264
DN13597_c0_g1_i1_1	4.711018	1.292949
DN135_c0_g1_i1_1	1.256236	2.811305
DN13600_c0_g1_i1_2	49.45142	275.4358
DN13604_c0_g2_i1_2	5.125376	1.114177
DN13606_c0_g1_i1_1	12.96497	15.14888
DN13607_c0_g1_i1_2	0.7904303	0.4086382
DN13608_c0_g2_i1_1	0.7626451	2.049408
DN13609_c0_g1_i1_2	3.10219	1.084798
DN13610_c0_g1_i2_2	3.131227	1.281072
DN13613_c0_g1_i2_1	2.148927	2.128597
DN13614_c0_g1_i1_1	1.21746	1.006446

DN13615_c0_g1_i1_1	4.451488	6.500827
DN13617_c0_g1_i1_2	0	0
DN1361_c0_g1_i1_2	3.684453	0.6125249
DN13620_c0_g1_i1_1	4.873677	2.773229
DN13623_c0_g1_i2_1	3.533668	6.344824
DN13624_c0_g1_i1_1	0.5289821	1.565343
DN13624_c0_g1_i1_2	3.628953	1.275754
DN13625_c0_g1_i1_1	0.6601965	0.9267852
DN13634_c0_g2_i1_2	3.150288	6.006268
DN13637_c0_g1_i1_1	4.180809	7.806075
DN13637_c0_g1_i2_2	8.047047	2.434078
DN13638_c0_g2_i1_1	8.74E-06	0.003426345
DN13639_c0_g1_i1_1	5.985187	92.66415
DN13642_c0_g1_i1_2	5.766826	4.449746
DN13644_c0_g1_i1_1	7.690841	6.812435
DN13645_c0_g1_i2_1	4.041143	16.32912
DN13653_c0_g1_i1_1	14.06665	10.60417
DN13658_c0_g1_i1_1	0.5047468	1.823094
DN13660_c0_g1_i1_2	53.43588	117.4706
DN13661_c0_g1_i1_1	0.35415	2.857714
DN13666_c0_g1_i2_1	3.01763	1.335016
DN13667_c0_g2_i1_1	0.2994634	4.856489
DN13668_c0_g1_i1_2	34.06795	0.340359
DN13670_c0_g1_i1_1	0.1236119	5.262601

DN13670_c0_g1_i1_2	0.5750491	0.8633824
DN13672_c0_g1_i1_1	0.6250982	1.412636
DN13686_c0_g1_i1_1	2.526834	1.224718
DN13686_c0_g1_i1_2	5.493411	2.721766
DN13687_c0_g1_i2_1	0.9886191	1.181658
DN1368_c0_g1_i1_1	27.30352	19.32964
DN1368_c0_g1_i1_2	4.428921	0.9285163
DN13695_c0_g1_i2_2	11.71177	7.866227
DN13696_c0_g1_i1_2	0.9804332	0.2393917
DN13697_c0_g1_i2_2	2.288351	0.9813855
DN13698_c0_g1_i2_1	1.490196	1.33108
DN13703_c0_g1_i1_1	0.7511465	0.8428318
DN13703_c0_g2_i1_1	0.807717	1.133316
DN13707_c0_g1_i1_1	0.769178	7.584874
DN13713_c0_g1_i1_1	0.2988001	3.271247
DN13714_c0_g1_i1_2	2.914954	3.831599
DN13720_c0_g1_i1_1	1.138463	1.636324
DN13723_c1_g1_i1_1	0.2548936	0.2726345
DN13728_c0_g1_i1_1	5.483699	4.082106
DN13730_c0_g1_i1_2	22.5564	29.12695
DN13741_c0_g1_i1_2	1.498979	1.178365
DN13743_c0_g1_i1_1	2.00455	3.383263
DN13744_c0_g1_i2_1	0.7911891	3.924511
DN13747_c0_g1_i1_2	1.493643	0.09423612

DN13750_c0_g1_i1_1	1.612696	1.25792
DN13752_c0_g1_i1_2	7.036117	3.559728
DN13752_c0_g3_i1_2	6.584946	11.6758
DN13755_c0_g1_i1_2	2.66403	1.975633
DN13756_c0_g1_i1_2	3.977055	2.618892
DN13757_c0_g1_i1_1	29.78437	14.05665
DN13760_c0_g1_i2_1	2.522494	1.863363
DN13764_c0_g1_i1_1	7.206803	6.707281
DN13765_c0_g1_i2_2	41.97389	53.87915
DN13767_c0_g1_i1_1	0.5546505	1.382544
DN13769_c0_g1_i1_1	0.05816365	1.69165
DN13771_c0_g1_i1_1	5.213589	4.626225
DN13771_c0_g1_i1_2	1.882663	0.9786053
DN13773_c0_g1_i1_1	2.493022	2.069439
DN13773_c0_g1_i1_2	1.949208	1.221445
DN13776_c0_g1_i1_1	0.8294609	1.992131
DN13776_c0_g2_i1_1	0.4836415	1.714561
DN13778_c0_g1_i3_1	3.091396	3.476839
DN13780_c0_g1_i1_1	1.519102	1.621902
DN13781_c0_g1_i1_2	7.232129	0.6891485
DN13782_c0_g1_i1_2	5.425179	4.713501
DN13783_c0_g1_i1_1	13.99766	10.1079
DN13785_c0_g1_i1_2	39.12523	12.97161
DN13786_c0_g1_i1_1	2.085089	3.938224

DN13787_c0_g1_i1_1	0.06403157	0.5906661
DN13788_c0_g1_i1_1	1.343961	0.7125188
DN13791_c0_g1_i1_1	0.1375994	1.168834
DN13797_c0_g1_i1_1	0.6205922	0.8075312
DN13798_c0_g1_i1_2	12.75928	11.64047
DN1379_c0_g1_i1_1	1.069822	0
DN13803_c0_g1_i1_2	1.103764	0.7845565
DN13812_c0_g1_i1_2	6.374328	2.627258
DN13823_c0_g1_i1_1	1.230214	2.829825
DN13824_c0_g1_i1_1	1.997015	1.399352
DN13828_c0_g1_i1_1	2.390564	6.161594
DN13834_c0_g1_i1_1	1.507494	1.189699
DN13834_c0_g2_i1_2	0.6257132	1.374599
DN13835_c0_g1_i1_2	1.854268	0.8772973
DN13835_c0_g2_i1_1	0.2919378	1.018997
DN13835_c0_g4_i1_1	0.06644134	0.9824199
DN13835_c0_g5_i1_1	7.282164	150.1603
DN13836_c0_g2_i1_2	3.145093	1.917214
DN13840_c0_g1_i3_1	14.0881	2.385988
DN13844_c0_g1_i1_1	1.00909	1.625476
DN13848_c0_g1_i1_1	0.56817	6.506084
DN13850_c0_g1_i1_2	1.047094	0
DN13851_c0_g1_i2_1	0.05286857	1.049391
DN13856_c0_g1_i1_1	0.4306243	8.337106

DN13857_c0_g1_i1_2	4.070376	1.044729
DN13858_c0_g1_i1_1	1.736152	2.228207
DN13858_c0_g1_i1_2	1.368043	0.6309173
DN13861_c0_g1_i1_2	1.319402	0.6580002
DN13862_c0_g1_i1_2	1.321988	1.488945
DN13863_c0_g1_i1_1	6.340873	2.527883
DN13865_c0_g1_i1_1	28.87513	84.29699
DN13869_c0_g1_i1_1	1.094117	2.132873
DN13870_c0_g1_i1_2	0.7636607	0.4753931
DN13871_c0_g1_i1_2	2.538058	1.448433
DN13872_c0_g1_i1_1	4.821457	1.489079
DN13873_c0_g1_i1_2	12.59603	8.018495
DN13873_c0_g2_i1_2	3.306046	0.4179631
DN13874_c0_g1_i2_1	0	2.47311
DN13875_c0_g1_i1_1	1.204786	1.278998
DN13880_c0_g1_i1_2	0.3454336	0.4535271
DN13880_c0_g2_i1_2	3.325999	0.8768273
DN13881_c0_g1_i1_2	6.868624	2.012819
DN13884_c0_g1_i1_1	1.154023	1.54732
DN13884_c0_g1_i1_2	1.566503	1.461732
DN13884_c0_g2_i1_2	0.5721692	0
DN13885_c0_g1_i2_2	3.826494	2.053111
DN13887_c0_g2_i1_1	0.6377641	2.519321
DN13890_c0_g1_i1_1	3.431473	10.78971

DN13891_c0_g1_i1_2	4.135488	2.143357
DN13891_c0_g2_i1_1	0.2380045	1.440746
DN13897_c0_g1_i1_1	23.83099	115.4521
DN13899_c0_g1_i1_1	0	2.149117
DN13899_c0_g2_i1_1	0.9075291	4.102661
DN13899_c0_g3_i1_1	0	1.82381
DN1389_c0_g1_i1_2	1.486532	0.4114696
DN13902_c0_g1_i1_2	1.243339	0.3580237
DN13903_c0_g1_i1_2	5.839539	1.424267
DN13903_c0_g2_i1_2	1.70913	0.7977449
DN13906_c0_g1_i1_2	3.131278	1.471523
DN13907_c0_g1_i1_2	2.108349	1.624653
DN13910_c0_g1_i1_1	2.509249	7.753845
DN13912_c0_g1_i1_2	1.669249	0.515471
DN13912_c0_g2_i1_2	3.62E-11	0
DN13913_c0_g1_i2_1	2.322511	0.8931259
DN13913_c0_g2_i1_1	1.549096	1.136736
DN13913_c0_g3_i2_1	1.066732	0.9953729
DN13919_c0_g1_i1_2	0.653148	0.9711964
DN13921_c0_g1_i1_1	3.53461	3.485894
DN13923_c0_g1_i1_1	15.47843	12.26844
DN13925_c0_g1_i1_1	32.16313	45.5349
DN1392_c0_g1_i1_2	1.353894	0
DN13930_c0_g1_i1_1	1.02924	1.159165

DN13933_c0_g1_i1_1	1.384276	2.405185
DN13934_c0_g1_i1_1	16.62257	34.88222
DN13936_c0_g1_i1_1	0.8821036	2.864805
DN13936_c0_g1_i1_2	0.3222406	0.03492132
DN13941_c0_g1_i1_2	1.894122	1.258555
DN13942_c0_g1_i1_2	3.092949	1.414557
DN13943_c0_g1_i1_1	1.577708	2.590444
DN13944_c0_g1_i1_1	3.683909	4.653955
DN13945_c0_g1_i2_1	0.8222193	1.310482
DN13947_c0_g1_i1_2	10.76066	16.36326
DN1394_c0_g1_i1_1	1.70E-25	0.3956819
DN13953_c0_g1_i1_1	0.1183433	2.416458
DN13954_c0_g1_i1_1	1.418307	0.5239148
DN13954_c0_g2_i1_1	0.3393528	1.550795
DN13958_c0_g1_i1_1	2.104755	0.7268044
DN13960_c0_g1_i1_2	7.627015	19.95897
DN13961_c0_g1_i1_1	0.9225211	1.570264
DN13961_c0_g1_i1_2	1.924442	1.482322
DN13961_c0_g2_i1_1	0.452114	1.224203
DN13965_c0_g1_i1_1	43.54145	48.23464
DN13965_c0_g2_i1_1	3.136106	3.746308
DN13969_c0_g1_i2_1	1.416947	2.274679
DN1396_c0_g1_i1_2	5.932465	2.876747
DN13976_c0_g1_i1_2	2.68011	0.2140173

DN1397_c0_g1_i2_2	0.7364838	0.5594307
DN13981_c0_g1_i1_1	1.939139	2.299446
DN13986_c0_g1_i1_1	0.3022461	1.248778
DN13986_c0_g2_i2_1	1.177785	2.583657
DN13986_c0_g3_i2_1	1.156084	1.622576
DN13986_c0_g4_i1_1	0.3683734	1.985907
DN13989_c0_g1_i1_1	0.06568578	6.551337
DN13992_c0_g1_i1_1	3.07621	2.569013
DN13995_c0_g1_i1_1	4.644933	5.967624
DN13996_c0_g1_i1_2	5.458297	0.9973259
DN13997_c0_g1_i1_1	0.9996153	1.728513
DN13997_c0_g2_i1_1	0.6788479	1.063347
DN13998_c0_g1_i1_1	2.312614	3.434674
DN13_c0_g1_i1_2	0	0
DN14001_c0_g1_i1_1	0.5340254	1.273079
DN14003_c0_g1_i1_2	2.06127	3.390682
DN14011_c0_g1_i1_1	1.084793	5.74785
DN14013_c0_g1_i1_2	3.617668	1.931592
DN14013_c0_g2_i1_2	1.905988	0.6090131
DN14017_c0_g1_i1_2	10.10101	3.926782
DN14018_c0_g1_i1_1	0.563065	1.045693
DN1401_c0_g1_i1_1	2.047104	0.6312677
DN14020_c0_g1_i2_1	6.499881	1.786879
DN14026_c0_g1_i1_2	2.781801	1.264586

DN14026_c0_g3_i1_2	8.395646	1.532093
DN14027_c0_g1_i1_1	0.3694492	1.469135
DN14029_c0_g1_i1_1	15.72686	18.64649
DN14030_c0_g1_i1_2	1.93488	1.235861
DN14031_c0_g1_i1_1	0.8929905	1.291389
DN14031_c0_g1_i1_2	2.302572	0.3673023
DN14035_c0_g2_i1_1	1.129919	0.9140065
DN14037_c0_g1_i1_2	1.874271	0.2845721
DN14039_c0_g1_i1_2	66.10572	21.06402
DN14042_c0_g1_i1_1	20.02895	31.50579
DN14045_c0_g1_i1_2	2.189374	0.3295571
DN14046_c0_g1_i1_2	1.79745	0.514236
DN14047_c0_g1_i1_1	0.3888263	1.561281
DN14047_c0_g2_i1_1	1.280733	2.792119
DN14047_c0_g2_i1_2	2.979367	1.742197
DN14049_c0_g1_i1_1	1.187748	1.734054
DN14049_c0_g2_i1_1	0	1.407746
DN14062_c0_g1_i1_1	13.75297	24.24005
DN14065_c0_g1_i1_1	0.6042961	2.654879
DN14065_c0_g1_i1_2	3.250114	2.77029
DN14070_c0_g1_i2_1	32.00213	19.75375
DN14070_c0_g1_i2_2	9.933414	5.964461
DN14072_c0_g1_i1_1	1.197372	1.470662
DN14073_c0_g1_i1_1	4.091953	3.425887

DN14075_c0_g1_i1_1	0	0.904816
DN14075_c0_g1_i2_2	6.17185	1.343326
DN14075_c0_g2_i2_1	0.2556987	4.117671
DN14078_c0_g1_i1_1	1.383542	0.7052782
DN14078_c0_g2_i1_1	2.139552	1.763256
DN14080_c0_g1_i1_2	3.345252	3.14513
DN14084_c0_g1_i2_1	7.989354	3.100139
DN14085_c0_g1_i1_1	6.212373	1.64452
DN14086_c0_g1_i1_2	2.176645	1.358861
DN14089_c0_g1_i1_1	1.31328	2.156632
DN14092_c0_g1_i1_1	1.232902	2.26179
DN14095_c0_g1_i1_2	2.947723	0.2957477
DN14098_c0_g2_i1_1	17.51306	613.4311
DN14103_c0_g1_i2_2	6.384233	8.996388
DN14104_c0_g1_i5_1	9.124421	4.534151
DN14109_c0_g1_i1_1	0.3591265	10.84392
DN1410_c0_g1_i1_1	0.5825877	2.452371
DN14110_c0_g3_i1_1	1.424208	2.053815
DN14113_c0_g1_i2_2	2.804746	9.146443
DN14119_c0_g1_i1_2	1.858189	1.242327
DN14121_c0_g2_i1_1	1.834874	1.54088
DN14122_c0_g1_i2_2	14.74359	8.275771
DN14128_c0_g1_i1_1	1.131487	1.084917
DN14128_c0_g2_i1_2	1.615439	1.963558

DN14131_c0_g1_i1_1	1.382629	2.472209
DN14132_c0_g1_i1_1	0.2027233	2.952462
DN14132_c0_g1_i1_2	8.806145	0.9316457
DN14143_c0_g1_i1_1	3.541905	3.999392
DN14149_c0_g1_i1_1	1.114964	2.666322
DN14152_c0_g1_i1_2	4.61656	42.67468
DN14155_c0_g1_i1_2	3.689544	0.826792
DN14159_c0_g1_i1_1	5.738885	2.688702
DN14163_c0_g1_i1_1	0.9537585	2.970349
DN14164_c0_g1_i4_1	133.3585	53.79029
DN14174_c0_g1_i1_1	5.420192	24.60744
DN14174_c0_g1_i1_2	5.345592	0.9706239
DN14180_c0_g1_i1_1	0.9957314	2.861089
DN14181_c0_g1_i1_2	883.0467	944.4127
DN14186_c0_g1_i1_2	1.89005	1.20911
DN14190_c0_g1_i1_2	16.7794	12.5891
DN14193_c0_g1_i2_2	3.954484	2.900896
DN1419_c0_g1_i1_1	3.034433	2.73899
DN14200_c0_g1_i1_2	1.668504	0.8502577
DN14200_c0_g2_i1_2	1.627242	0.6273824
DN14202_c0_g1_i1_1	6.170347	15.18789
DN14203_c0_g1_i1_2	6.154771	2.095997
DN14203_c0_g2_i1_2	7.569116	1.534592
DN14205_c0_g1_i1_2	16.68983	10.70137

DN14206_c0_g1_i1_2	1.223708	0.5007037
DN14209_c0_g1_i1_2	1.612787	0.2612452
DN14209_c0_g2_i1_2	2.160553	0.2008508
DN14214_c0_g1_i1_1	5.58855	5.173109
DN14220_c0_g1_i1_2	2.976855	0.895074
DN14221_c0_g1_i1_1	0.3688481	0.6238788
DN14222_c0_g1_i1_2	1.202495	0.4545135
DN14223_c0_g2_i1_2	2.202267	1.77E-34
DN14228_c0_g1_i1_2	2.320314	3.531405
DN14229_c0_g1_i1_2	2.256674	1.691419
DN14230_c0_g1_i1_1	2.807214	3.630223
DN14230_c0_g2_i1_1	1.910036	1.644291
DN14232_c0_g2_i1_1	1.334048	1.574366
DN14233_c0_g1_i1_1	15.64533	19.50607
DN14243_c0_g1_i1_2	6.048894	6.016017
DN14247_c0_g1_i1_1	0.8536903	1.691241
DN14254_c0_g1_i1_2	3.684544	2.012266
DN14256_c0_g1_i1_1	3.257392	4.679521
DN14257_c0_g1_i1_2	3.391422	3.906952
DN14260_c0_g1_i2_1	4.199367	5.96677
DN14262_c0_g1_i2_1	1.103096	3.652322
DN14263_c0_g1_i2_2	4.330947	0.7004751
DN14265_c0_g2_i1_1	2.04899	1.559964
DN14268_c0_g1_i1_2	20.80018	16.97024

DN14268_c0_g1_i2_1	1.153194	0.9189975
DN1426_c0_g1_i1_1	0.9662109	1.049166
DN1426_c0_g1_i1_2	2.372326	1.751667
DN14274_c0_g1_i1_1	1.327874	22.85264
DN14275_c0_g1_i2_1	1.795213	14.74914
DN14278_c0_g1_i1_2	1.634266	1.916396
DN14284_c0_g1_i1_2	5.320896	3.264257
DN14288_c0_g1_i1_2	2.656022	0.1737134
DN14289_c0_g1_i2_1	129.7548	108.5127
DN1428_c0_g1_i1_1	0.06628249	0.3310913
DN14291_c0_g1_i1_2	31.64762	11.32095
DN14294_c0_g1_i2_1	1.316368	1.039328
DN14296_c0_g1_i1_1	20.33739	16.89146
DN14297_c0_g2_i1_2	5.206106	3.382779
DN14300_c0_g1_i1_1	2.71E-08	1.669199
DN14300_c0_g2_i1_1	2.843126	1.316216
DN14302_c0_g1_i1_1	30.14999	21.56962
DN14303_c0_g1_i1_2	10.00464	3.724544
DN14303_c0_g1_i2_1	6.050878	5.387561
DN14303_c0_g2_i1_1	0.870688	1.055225
DN14305_c0_g1_i1_1	1.115359	1.308567
DN1430_c0_g1_i1_2	0.5328995	0.2995568
DN14310_c0_g1_i1_2	1.513219	0.8664408
DN14311_c0_g1_i1_2	1.578591	1.796783

DN14313_c0_g1_i1_1	2.317672	6.706904
DN14317_c0_g1_i3_1	21.40517	9.29885
DN14319_c0_g1_i1_2	1.069738	0.174478
DN14319_c1_g1_i1_1	0.8075102	2.602319
DN14320_c0_g1_i1_2	3.569896	1.050103
DN14321_c0_g1_i3_1	0	0.3834578
DN14323_c0_g1_i1_1	1.756935	3.54585
DN14329_c0_g1_i1_1	1.42075	3.316642
DN14330_c0_g1_i1_1	1.775527	1.181261
DN14336_c0_g1_i1_2	2.615666	1.014997
DN14337_c0_g1_i1_1	1.276982	1.57518
DN14356_c0_g1_i1_2	1.131463	0.200567
DN14361_c0_g2_i1_1	0.820895	0.6109013
DN14369_c0_g1_i1_1	1.195259	2.402384
DN14369_c0_g2_i1_1	2.83656	5.489808
DN14371_c0_g1_i1_1	18.27834	16.17664
DN14376_c0_g1_i2_1	5.41509	6.583489
DN14378_c0_g1_i3_1	1.374143	2.587874
DN14382_c0_g1_i1_1	3.802119	4.938579
DN14382_c0_g1_i1_2	5.890177	3.691783
DN14388_c0_g2_i1_2	1.525484	1.752957
DN14388_c0_g5_i1_2	1.012046	0.1075389
DN14389_c0_g1_i1_1	9.321106	2.172972
DN1438_c0_g1_i1_2	0.5522112	0.3945012

DN1438_c0_g1_i2_1	20.66988	18.96921
DN14391_c0_g1_i1_2	1.028369	0.8185829
DN14394_c0_g1_i1_2	1.118144	2.507059
DN14395_c0_g1_i1_1	1.998205	2.644698
DN14396_c0_g3_i1_1	1.607764	0.7050741
DN14396_c0_g4_i1_1	7.312109	2.075849
DN14396_c0_g5_i1_1	1.931984	1.910166
DN14404_c0_g1_i1_2	4.044228	3.285588
DN14405_c0_g2_i1_2	2.78241	3.971509
DN14406_c0_g1_i1_2	0.7191457	0.7748569
DN14410_c0_g1_i1_1	0.8979283	4.706056
DN14410_c0_g2_i1_1	0.368435	0.2475086
DN14410_c0_g3_i1_1	1.471542	0.7527148
DN14413_c0_g1_i1_2	1.163397	1.045273
DN14413_c0_g2_i1_2	2.323496	4.68E-26
DN14421_c0_g1_i1_1	1.92426	4.487081
DN14424_c0_g1_i1_1	3.45319	3.813599
DN14425_c0_g1_i1_1	1.976756	7.243333
DN14427_c0_g1_i1_2	1.944534	0.4394607
DN14429_c0_g1_i1_1	0.5979535	1.597957
DN14429_c0_g2_i1_1	0.490169	2.069243
DN14431_c0_g1_i1_2	0.2418389	0.1819098
DN14433_c0_g2_i1_1	0.6054497	0.3984746
DN14434_c0_g1_i1_2	1.35165	0.6379761

DN14435_c0_g1_i1_2	30.1481	43.46782
DN14439_c0_g1_i1_2	0.974126	1.007102
DN14440_c0_g1_i1_2	0	0
DN14446_c0_g1_i1_1	2.934487	6.057746
DN14452_c0_g2_i1_1	0.3322541	3.257776
DN14456_c0_g1_i1_2	1.861925	1.947642
DN14460_c0_g1_i1_1	0.342488	1.100952
DN14460_c0_g2_i1_1	0.4832353	4.997798
DN14463_c0_g1_i1_2	1.374524	1.470932
DN14464_c0_g1_i1_2	5.160942	0.9321
DN14464_c0_g2_i1_2	1.95497	0.4556517
DN1446_c0_g1_i1_1	1.134575	2.666385
DN14473_c0_g1_i1_1	18.0095	47.80176
DN14479_c0_g1_i1_1	1.542188	1.59628
DN14482_c0_g1_i2_2	3.380718	1.434056
DN14483_c0_g1_i1_2	9.521799	2.934959
DN14485_c0_g2_i1_1	1.365515	2.845891
DN14486_c0_g1_i1_1	0.6325245	2.666895
DN14496_c0_g1_i1_1	0.1735313	0.7680223
DN14498_c0_g1_i3_2	1.304296	0.6345589
DN14499_c0_g1_i1_2	1.05185	0.740896
DN14500_c0_g1_i1_1	1.439857	4.117312
DN14502_c0_g2_i1_1	2.240953	5.371255
DN14503_c0_g1_i1_2	0.9202422	0.3643285

DN14504_c0_g1_i1_2	3.024777	1.351245
DN14507_c0_g1_i1_2	4.086057	0.2985207
DN14508_c0_g2_i1_1	0.2151468	0.6672281
DN14517_c0_g1_i1_2	2.328988	2.037856
DN14520_c0_g1_i1_2	0.3766182	0.08880252
DN14524_c0_g1_i1_1	13.92658	17.36153
DN14526_c0_g1_i1_1	0.8289646	2.055778
DN14526_c0_g2_i1_1	3.994862	1.903928
DN14528_c0_g2_i2_1	8.700063	5.859984
DN14528_c0_g3_i1_1	5.386534	4.297205
DN1452_c0_g1_i2_2	10.2305	6.030965
DN14531_c0_g1_i1_1	0.5514443	2.409243
DN14531_c0_g2_i1_1	0.3776956	2.957133
DN14535_c0_g1_i1_1	4.928186	11.28399
DN14537_c0_g2_i2_2	0	0
DN14547_c0_g1_i1_1	0.8729436	2.778612
DN14548_c0_g1_i1_2	2.555574	0
DN14550_c0_g1_i1_1	1.681525	2.996954
DN14557_c1_g1_i1_2	68.50714	0
DN14558_c0_g1_i1_1	11.21234	9.626322
DN14560_c0_g1_i2_1	0.73094	1.310999
DN14562_c0_g1_i1_2	3.502521	0.3030468
DN14565_c0_g1_i1_1	0.7816348	1.495703
DN14565_c0_g2_i2_1	1.864695	0.837647

DN14566_c0_g1_i2_1	1.127436	0.482746
DN14568_c0_g1_i2_1	18.21516	40.62811
DN14569_c0_g1_i1_1	3.468504	2.622494
DN14571_c0_g1_i1_2	3.350124	1.599183
DN14572_c0_g2_i1_1	0.7397177	1.715086
DN14572_c0_g3_i1_1	0.5289226	2.43031
DN14573_c0_g1_i1_1	3.275319	4.680558
DN14576_c0_g1_i1_1	3.565543	5.842285
DN14577_c0_g1_i1_1	1.90657	5.881265
DN14578_c0_g1_i2_2	4.014661	0.8083719
DN14578_c0_g2_i1_2	6.611142	2.014261
DN14579_c0_g1_i1_1	2.011083	1.856409
DN14581_c0_g1_i1_1	2.131626	4.13626
DN14583_c0_g1_i1_2	8.667369	6.342681
DN14587_c0_g1_i1_2	3.573267	2.937079
DN14594_c0_g1_i1_1	1.908452	2.341955
DN14611_c0_g1_i1_1	1.514615	1.373617
DN14612_c0_g1_i1_2	1.015195	0.6193878
DN14619_c0_g1_i1_2	43.06653	25.10101
DN14622_c0_g1_i1_1	0.5284434	1.346887
DN14623_c0_g2_i1_2	1.364409	0.2008388
DN14624_c0_g1_i1_1	68.47152	42.40514
DN14625_c0_g1_i1_1	22.61888	20.00729
DN14626_c0_g1_i1_2	2.411765	1.127248

DN14627_c0_g1_i1_2	5.612215	8.484031
DN14628_c0_g1_i1_2	1.083019	2.532798
DN14629_c0_g2_i1_1	0.2429375	1.922117
DN14630_c0_g1_i2_1	1.866458	2.654421
DN14631_c0_g1_i1_1	3.160008	4.544453
DN14634_c0_g1_i1_1	100.9036	258.0671
DN14634_c0_g1_i1_2	5.930015	5.895847
DN14634_c0_g4_i1_1	5.582672	76.64168
DN14636_c0_g1_i1_2	17.44277	12.89774
DN14637_c0_g1_i1_1	1.605964	1.45703
DN14637_c0_g2_i1_1	0.2850102	2.613172
DN14638_c0_g1_i1_2	0.9307742	0.568174
DN14640_c0_g1_i1_1	0.832559	2.109293
DN14642_c0_g1_i1_1	4.874759	5.836338
DN14642_c0_g1_i1_2	1.369451	0.5523246
DN14643_c0_g2_i1_1	1.205453	0.639773
DN14645_c0_g1_i1_1	1.516023	3.372605
DN14646_c0_g1_i1_1	16.00083	12.13308
DN14646_c0_g1_i1_2	3.054933	1.425197
DN14649_c0_g1_i1_2	2.71739	1.917018
DN1464_c0_g1_i1_1	0.4425574	1.455054
DN14651_c0_g1_i1_1	1.313363	3.440948
DN14656_c0_g1_i1_2	1.334809	1.464611
DN14666_c0_g1_i1_2	5.958199	3.828814

DN14669_c0_g1_i1_2	2.950191	0.5018585
DN14672_c0_g1_i1_1	6.961935	3.146905
DN14674_c0_g1_i1_1	1.19E-91	0.3413565
DN14674_c0_g2_i1_1	0.588276	0.3405422
DN14674_c0_g3_i1_1	1.671837	1.827779
DN14678_c0_g1_i1_2	3.213862	1.091537
DN14681_c0_g1_i1_1	0.5389433	6.843416
DN14685_c0_g1_i1_2	13.61079	9.023127
DN14686_c0_g1_i2_1	4.951674	7.798335
DN14688_c0_g2_i1_1	5.401462	3.566018
DN14689_c0_g1_i1_1	4.705679	3.483301
DN14694_c0_g1_i1_1	2.947301	2.001327
DN14695_c0_g1_i1_1	0.3155473	0.8005868
DN14695_c0_g2_i1_1	0.4427885	0.4927469
DN14696_c0_g1_i1_1	0.104539	7.830078
DN14704_c0_g1_i1_1	0.7995437	1.946861
DN14704_c0_g2_i1_1	0.2761757	1.00971
DN14707_c0_g1_i1_1	64.47521	290.0077
DN14712_c0_g1_i1_2	19.01299	7.919432
DN14714_c0_g1_i1_1	2.943405	1.880916
DN14714_c0_g1_i1_2	1.950585	2.382335
DN14715_c0_g1_i1_1	1.400033	1.96475
DN14720_c0_g1_i1_2	2.731251	1.896819
DN14726_c0_g1_i1_1	18.30525	394.7095

DN1472_c0_g1_i1_1	8.564676	4.176412
DN14732_c0_g1_i1_2	2.680367	1.76597
DN14733_c0_g1_i1_1	1.978265	4.382874
DN14740_c0_g1_i2_1	2.838816	8.153136
DN14741_c0_g1_i1_2	8.02496	6.706831
DN14743_c0_g1_i1_2	22.9115	25.86496
DN14744_c0_g1_i1_1	5.044354	2.565536
DN14744_c0_g1_i1_2	1.210289	1.151381
DN14746_c0_g1_i1_1	0.8428124	1.619637
DN14749_c0_g1_i1_1	1.601827	2.765241
DN14750_c0_g1_i1_1	0.8847387	1.321094
DN14758_c0_g3_i1_1	1.821511	2.838937
DN14760_c0_g1_i1_2	4.47411	1.270267
DN14761_c0_g1_i1_2	12.16976	5.882239
DN14766_c0_g1_i1_2	39.30271	9.545198
DN14769_c0_g1_i2_1	4.681327	16.55545
DN1476_c0_g1_i1_1	0.2192424	0.4749444
DN14770_c0_g2_i1_1	0.3922595	0.629086
DN14770_c0_g4_i4_1	1.062755	1.568417
DN14773_c0_g1_i1_1	1.568574	1.132739
DN14774_c0_g1_i1_1	2.037294	1.336241
DN14774_c0_g1_i1_2	0.7237604	0.5908344
DN14775_c0_g1_i1_1	0.1766275	0.5320421
DN14778_c0_g1_i2_1	74.50327	73.10217

DN14779_c0_g1_i1_1	3.531526	4.682498
DN14788_c0_g1_i1_2	54.6143	35.37659
DN14789_c0_g1_i2_1	246.2399	59.86271
DN14793_c0_g1_i1_1	1.342504	1.607389
DN14793_c0_g2_i1_2	4.782262	1.608119
DN14795_c0_g1_i1_2	0	0
DN14797_c0_g1_i1_2	2.10048	1.224692
DN14802_c0_g1_i1_1	3.764847	5.834107
DN14804_c0_g1_i1_1	0.5879973	2.117515
DN14806_c0_g1_i1_2	2.537182	1.098726
DN14811_c0_g1_i1_1	1.11245	14.45772
DN14813_c0_g1_i1_2	1.272747	0.6727706
DN14814_c0_g1_i1_1	1.196396	1.749519
DN14818_c0_g1_i1_1	1.028276	1.768664
DN14818_c0_g1_i1_2	4.468125	1.205352
DN14819_c0_g1_i1_2	1.071767	0.9117436
DN14821_c0_g1_i1_1	1.442461	5.15144
DN14825_c0_g1_i1_2	7.412302	3.375935
DN14828_c0_g1_i3_1	12.32624	24.41498
DN14834_c0_g1_i1_2	2.349817	1.202933
DN14838_c0_g1_i1_1	1.268997	1.579058
DN14838_c0_g1_i1_2	1.013543	0.9606074
DN14838_c0_g2_i1_2	3.133185	4.34E-60
DN14843_c0_g1_i1_1	1.49606	1.685321

DN14847_c0_g1_i1_1	1.0001	1.607604
DN14847_c1_g2_i1_1	0.5824213	2.0126
DN14847_c1_g3_i1_1	1.520311	2.75631
DN14848_c0_g1_i1_1	10.49687	4.426386
DN14848_c0_g1_i1_2	1.489603	2.711942
DN14853_c0_g1_i1_2	2.809251	0.9759064
DN14853_c0_g2_i1_2	11.2832	2.66537
DN14853_c0_g3_i1_2	3.272443	1.929043
DN14854_c0_g1_i2_1	3.693463	5.450587
DN14855_c0_g1_i1_1	3.87342	16.81186
DN14856_c0_g1_i1_1	1.478853	1.749658
DN14864_c0_g1_i1_1	1.998748	2.431232
DN14865_c0_g1_i1_2	1.42878	0.9066755
DN14869_c0_g1_i1_2	2.686146	1.224649
DN1486_c0_g1_i1_1	0.8930258	0.7526362
DN1486_c0_g1_i1_2	6.81246	1.307426
DN14872_c0_g1_i1_1	0.2068352	7.885084
DN14873_c0_g1_i1_2	8.712975	15.26007
DN14874_c0_g1_i1_1	11.92892	13.31681
DN14874_c0_g2_i1_1	2.105825	0.8713278
DN14878_c0_g1_i2_2	1.715347	0.02727278
DN1487_c0_g1_i1_1	1.235349	1.640937
DN14882_c0_g1_i1_1	6.162716	15.21798
DN14886_c0_g1_i1_1	8.774548	4.788946

DN14887_c0_g1_i1_2	1.313291	1.268942
DN14888_c0_g1_i1_2	1.922469	2.787988
DN14888_c0_g2_i1_2	0.3860392	0
DN14888_c0_g3_i2_2	3.258344	2.471438
DN14890_c0_g1_i1_2	46.70867	23.42224
DN14893_c0_g1_i1_1	2.814942	3.116909
DN14897_c0_g1_i1_1	2.791378	3.159838
DN14897_c0_g1_i1_2	5.127678	4.737481
DN14899_c0_g1_i1_2	0.7190191	0.6733779
DN148_c0_g1_i1_1	155.8038	471.9117
DN14902_c0_g1_i1_2	433.9486	1.258569
DN14907_c0_g3_i1_2	0.8615823	0.5912492
DN14909_c0_g1_i1_2	1.604317	0.5620828
DN1490_c0_g1_i1_1	2.097877	2.977272
DN14910_c0_g1_i1_1	1.597273	1.273233
DN14910_c0_g1_i1_2	2.220897	0.759414
DN14910_c0_g2_i1_2	1.574726	0
DN14916_c0_g1_i1_2	100.4949	1.753283
DN14919_c0_g1_i2_1	7.188529	9.098635
DN14920_c0_g1_i1_1	0.7638489	6.232043
DN14920_c0_g3_i1_2	1.603629	0.194204
DN14928_c0_g1_i2_1	1.287313	7.957512
DN14931_c0_g1_i1_1	0.6235332	0.9004879
DN14936_c0_g1_i2_1	1.402732	0.5141827

DN14937_c0_g3_i1_2	1.405791	0.701704
DN14940_c0_g1_i1_1	1.555052	2.76685
DN14945_c0_g1_i1_2	39.88725	353.5782
DN14945_c0_g2_i1_2	0	0
DN14948_c0_g2_i1_1	0.1419553	2.067191
DN14949_c0_g1_i1_1	14.26531	8.769562
DN14950_c0_g1_i1_2	3.578152	1.422184
DN14954_c0_g1_i1_1	1.799737	1.970443
DN14956_c0_g1_i1_2	28.57726	23.1743
DN14959_c0_g1_i1_2	2.065867	3.222347
DN1495_c0_g1_i1_1	1.451442	1.347952
DN14960_c0_g2_i1_2	29.86695	33.29944
DN14963_c0_g1_i2_1	3.23875	3.402851
DN14964_c0_g2_i1_2	2.003703	1.468322
DN14967_c0_g2_i1_2	0.5603955	0.691895
DN14970_c0_g2_i2_2	1.523296	0.5555759
DN14971_c0_g1_i1_1	0.7599389	2.283479
DN14971_c0_g1_i2_2	1.882122	0.6028909
DN14971_c0_g2_i1_1	0.2373492	3.522903
DN14972_c0_g1_i1_2	18.63233	19.05177
DN14979_c1_g1_i1_1	1.989054	0.6213177
DN14985_c0_g2_i1_1	3.82581	6.065606
DN14990_c0_g1_i1_2	6.710691	3.372471
DN14991_c0_g1_i1_1	7.977878	10.05069

DN14996_c0_g1_i1_2	22.88028	4.735548
DN14997_c0_g1_i1_1	0.3875223	0.3444343
DN14998_c0_g1_i1_2	6.603627	4.472459
DN15004_c0_g1_i1_2	0.4467243	2.656148
DN15006_c0_g1_i2_1	310.4996	318.3335
DN15008_c0_g1_i1_1	1.319477	1.642552
DN15011_c0_g1_i2_1	3.48123	2.027028
DN15011_c0_g2_i2_1	2.89364	2.202276
DN15018_c0_g1_i1_2	7.022468	4.883077
DN15020_c0_g1_i1_2	3.328331	3.122874
DN15022_c0_g1_i1_2	4.660055	1.861429
DN1502_c0_g1_i1_1	7.363971	18.72887
DN15032_c0_g1_i2_1	4.67381	5.902893
DN15035_c0_g1_i1_1	341.1427	343.3028
DN15038_c0_g1_i1_2	24.16334	12.7859
DN1503_c0_g1_i1_1	0.6696571	2.47037
DN15043_c0_g1_i1_2	1.728943	0.3200566
DN15044_c0_g1_i1_2	4.630703	2.053707
DN15044_c0_g3_i1_1	1.927762	4.518732
DN15047_c0_g2_i1_2	4.537539	1.619136
DN15050_c0_g1_i1_1	0.5465763	1.867269
DN15052_c0_g1_i1_1	78.77908	74.1606
DN15059_c0_g1_i1_1	13.06416	5.293505
DN1505_c0_g1_i1_1	4.235713	24.28933

DN15062_c0_g1_i1_1	8.525519	5.093511
DN15063_c0_g2_i1_1	8.802551	4.656103
DN15064_c0_g1_i1_2	30.08636	57.96068
DN15066_c0_g1_i1_1	3.944384	4.693276
DN15070_c0_g1_i1_2	1.620239	0.3519306
DN15070_c0_g2_i1_2	5.791361	1.753927
DN15071_c0_g1_i1_2	1.654132	0.767671
DN15073_c0_g1_i1_1	0.6025494	1.627402
DN15074_c0_g1_i1_2	19.07119	5.069326
DN15076_c0_g1_i1_2	4.766555	2.053993
DN15078_c0_g1_i1_2	7.845325	8.694334
DN15079_c0_g1_i4_1	4.528877	3.189127
DN1507_c0_g1_i1_1	12.78846	7.493556
DN1507_c0_g1_i1_2	2.486272	1.172136
DN15080_c0_g1_i1_2	0.3289307	0.4903076
DN15087_c0_g1_i1_1	0.7628696	0.6606992
DN15087_c0_g1_i1_2	3.219147	2.458739
DN15088_c0_g1_i1_1	26.21674	858.2457
DN15099_c0_g1_i1_2	45.37186	56.06293
DN15101_c0_g2_i1_2	6819.704	2660.91
DN15104_c0_g1_i1_1	2.127733	3.131488
DN15104_c0_g2_i1_1	2.404011	3.368873
DN15108_c0_g1_i1_1	1.216914	2.845953
DN15111_c0_g1_i1_1	1.563964	1.523218

DN15111_c0_g2_i1_1	1.711863	0.7959071
DN15113_c0_g1_i1_1	3.336934	1.840258
DN15117_c0_g1_i1_2	1.737819	1.567246
DN15118_c0_g1_i1_1	1.539915	2.359646
DN15118_c0_g2_i1_1	0.4675678	1.667505
DN15118_c0_g3_i1_1	0.9127285	0.9912979
DN15122_c0_g1_i1_1	0.9912869	1.50762
DN15128_c0_g1_i1_1	0	3.849142
DN15131_c0_g1_i1_1	2.342217	3.219049
DN15131_c0_g1_i1_2	4.590409	53.66875
DN15133_c0_g1_i1_1	0	0.3024359
DN15133_c0_g3_i1_1	0.9094067	1.040564
DN15133_c0_g5_i1_1	0.3538778	0.9602941
DN15133_c0_g6_i1_1	0.9034485	1.719111
DN15137_c0_g1_i1_2	3.218183	2.27564
DN15145_c0_g1_i1_2	1.842413	59.13271
DN15147_c0_g2_i2_1	1.947752	1.493702
DN15149_c0_g1_i1_1	36.45189	14.44895
DN15150_c0_g1_i1_1	1.112684	0.8693468
DN15150_c0_g2_i1_1	1.15193	1.224126
DN15152_c0_g1_i1_1	1.453042	1.678731
DN15153_c0_g1_i1_2	0.2752157	0.2628007
DN15153_c0_g1_i2_1	2.386619	2.576273
DN15153_c0_g2_i1_2	2.146123	0.1981966

DN15154_c0_g1_i1_2	2.542123	7.245375
DN15162_c0_g2_i1_1	27.06792	11.65165
DN15165_c0_g1_i1_2	2.607419	2.053876
DN15165_c1_g1_i1_1	1.028131	1.610152
DN15166_c0_g1_i3_2	1.999534	0.9803718
DN15168_c0_g1_i1_1	1.746442	1.87053
DN1516_c0_g1_i1_1	0.5616067	1.880517
DN1516_c0_g2_i1_1	1.537141	1.94151
DN15170_c0_g1_i1_1	0	2.19263
DN15172_c0_g1_i2_2	0	0
DN15179_c0_g1_i1_2	11.3598	6.218778
DN15181_c0_g1_i1_2	1.565877	1.366929
DN15188_c0_g1_i4_1	1.469299	1.6958
DN15192_c0_g1_i1_2	1.188958	0.5439999
DN15199_c0_g2_i1_2	0.3244447	1.485636
DN15201_c0_g1_i2_2	2.112705	1.333602
DN15201_c0_g2_i1_2	2.002572	0.5928545
DN15202_c0_g1_i1_2	8.494403	2.769766
DN15203_c0_g1_i1_1	1.006941	8.498069
DN15207_c0_g1_i2_1	3.260462	1.178228
DN1520_c0_g1_i1_1	0.6029096	2.757311
DN15215_c0_g1_i1_1	1.40594	0.8705298
DN15215_c0_g1_i1_2	1.327171	1.457114
DN15216_c0_g1_i1_2	3.694841	4.708633

DN15216_c0_g2_i1_2	3.989683	4.63819
DN15219_c0_g1_i1_1	3.447958	9.101103
DN15224_c0_g1_i1_1	5.224429	3.058054
DN15225_c0_g1_i1_2	3.269621	1.60782
DN15226_c0_g1_i2_2	203.3648	2463.008
DN15228_c0_g1_i1_2	0.2358992	0.6136399
DN15229_c0_g1_i1_2	5.91017	5.123802
DN15231_c0_g1_i1_2	1.150795	2.469931
DN15236_c0_g1_i1_2	14.19722	18.90735
DN15239_c0_g1_i2_2	4.197262	0.6309175
DN15239_c1_g2_i1_1	0	0
DN15239_c3_g2_i1_1	4.795157	3.417457
DN15239_c3_g3_i1_1	12.04028	13.63897
DN15239_c4_g4_i1_1	129.7549	91.96535
DN15242_c0_g1_i1_2	2.395109	14.15426
DN15244_c0_g1_i1_1	10.03369	6.989165
DN15245_c0_g1_i1_1	3.667751	2.412013
DN15248_c0_g1_i1_2	0.7148647	0.6810076
DN15250_c0_g1_i1_1	0.3596645	4.47453
DN15250_c0_g1_i1_2	1.517923	0.5155185
DN15251_c0_g1_i1_2	15.15923	59.36446
DN15256_c0_g1_i1_1	12.58186	7.457052
DN15257_c0_g1_i1_2	222.6275	140.0161
DN15257_c0_g1_i3_1	1.54293	1.324309

DN15259_c0_g1_i1_2	2.031473	1.539419
DN15260_c0_g1_i1_2	3.724308	2.27429
DN15267_c0_g1_i1_2	84.31315	440.7128
DN15270_c0_g1_i1_1	12.49099	5.570075
DN15271_c0_g2_i1_2	0.8588077	1.156262
DN15273_c0_g1_i2_1	3.366066	43.68472
DN15274_c0_g1_i1_2	1.723077	0.911931
DN1527_c0_g1_i1_1	1.210247	0.7585654
DN15283_c0_g1_i1_1	4.583478	2.591102
DN15283_c0_g1_i1_2	1.427581	1.347696
DN15286_c0_g1_i1_2	0	0
DN15288_c0_g1_i1_1	4.4986	6.097593
DN15291_c0_g1_i1_2	20.0868	10.49557
DN15295_c0_g1_i1_1	12.48989	13.2103
DN15299_c0_g1_i1_1	1.759352	5.096497
DN1529_c0_g1_i1_2	0.8106264	2.259921
DN152_c0_g1_i1_1	2.533444	2.692719
DN15302_c0_g1_i1_2	1.184433	0.6299797
DN15304_c0_g1_i1_1	1.354832	3.391461
DN15306_c0_g1_i1_2	3.915354	2.631289
DN15308_c0_g1_i1_1	4.239699	1.924139
DN15310_c0_g1_i1_1	1.01743	2.382875
DN15310_c0_g2_i2_1	0.8723974	1.198037
DN15318_c0_g1_i1_1	2.206976	2.573202

DN15319_c0_g1_i1_2	5.612508	2.563244
DN15320_c0_g1_i1_1	0.6884505	3.812316
DN15320_c0_g1_i1_2	1.717136	0.6557378
DN15320_c0_g2_i1_1	1.572756	3.552008
DN15320_c0_g3_i1_1	1.231649	3.320379
DN15320_c0_g4_i1_1	1.480126	1.440774
DN15324_c0_g1_i1_2	21.63175	40.71924
DN15326_c0_g1_i2_1	9.722122	14.94884
DN15337_c0_g1_i1_1	1.071991	2.668736
DN15339_c0_g1_i1_2	4.682449	2.695844
DN15340_c0_g2_i1_2	1.313628	0.7459377
DN15344_c0_g1_i2_2	6.794752	4.081667
DN15352_c0_g1_i1_2	6.075565	5.284725
DN15353_c0_g1_i1_1	0.8049952	2.172406
DN15359_c0_g1_i1_2	5.771911	2.475861
DN15362_c0_g2_i1_2	2.968635	2.686789
DN15364_c0_g1_i1_2	2.756024	5.577087
DN15369_c0_g1_i1_1	1.506554	4.140927
DN15375_c0_g2_i1_2	2.424275	1.082192
DN15376_c0_g1_i1_1	0.4144661	10.8546
DN15377_c0_g2_i1_1	1.340296	1.294977
DN15377_c0_g3_i1_1	0.5906847	0.896248
DN15377_c0_g4_i2_1	0.2360564	1.980552
DN15377_c0_g5_i1_1	0.5491171	2.663825

DN15381_c0_g1_i1_1	4.938789	20.12893
DN15381_c0_g1_i1_2	6.117683	4.5391
DN15382_c0_g1_i1_1	27.383	16.36151
DN1538_c0_g1_i1_1	3.097922	1.479776
DN15393_c0_g1_i1_2	1.232355	0.8884346
DN15398_c0_g4_i1_2	4.059246	1.078485
DN1539_c0_g1_i1_1	0.9287635	2.083886
DN15404_c0_g1_i2_2	2.089564	2.253226
DN15405_c0_g1_i1_2	2.645832	1.692401
DN15407_c0_g1_i1_1	1.597464	3.259602
DN15409_c0_g1_i1_2	0	0
DN15413_c0_g1_i1_2	1.382487	0.2648256
DN15414_c0_g1_i2_1	30.43273	9.782964
DN15419_c0_g1_i1_1	9.774422	14.4269
DN1541_c0_g1_i1_2	1.777048	1.392126
DN15422_c0_g1_i1_2	22.6727	8.998584
DN15429_c0_g1_i1_2	2.108896	3.96156
DN15429_c0_g1_i2_1	1.00079	1.653574
DN1542_c0_g1_i1_2	2.280982	0.7983994
DN15431_c0_g1_i1_2	114.8303	58.43054
DN15432_c0_g2_i1_1	7.54E-107	0.7254547
DN15434_c0_g1_i1_2	5.013533	1.370428
DN15441_c0_g1_i1_2	1.445747	0
DN15442_c0_g1_i1_1	1.692455	1.789454

DN15447_c0_g1_i2_1	3.31758	18.69955
DN15451_c0_g1_i1_2	0.1887408	0.5219462
DN15454_c0_g1_i1_2	4.476575	3.924569
DN15456_c0_g1_i1_2	4.216315	8.597395
DN15457_c0_g1_i1_1	13.5508	11.4796
DN15467_c0_g1_i1_1	12.71602	8.371928
DN15468_c0_g1_i2_1	4.245146	6.725191
DN15469_c0_g1_i1_1	1.603374	2.477627
DN15473_c0_g1_i1_1	2.044162	7.541746
DN15477_c0_g4_i1_1	0.6514766	0.9302893
DN15477_c0_g5_i1_1	1.208019	3.739627
DN15478_c0_g1_i1_2	0.7141372	0.8326128
DN15478_c0_g3_i1_2	0.853865	0.7245872
DN1547_c0_g1_i1_1	0.3427003	2.387112
DN15482_c0_g1_i1_1	2.312383	1.800691
DN15482_c0_g1_i1_2	26.41417	3.739632
DN15482_c0_g3_i1_2	108.3755	12.86862
DN15482_c0_g4_i1_1	1.350483	1.554959
DN15483_c0_g1_i2_2	6.971791	9.852134
DN15491_c0_g2_i1_2	5.612584	0.6084988
DN15492_c0_g1_i1_2	3.897228	1.764583
DN15493_c0_g1_i1_2	72.12016	51.31495
DN15494_c0_g1_i1_2	1.582422	0.3036764
DN15496_c0_g2_i1_1	0.8985254	1.892236

DN15496_c0_g3_i1_1	2.745261	5.283056
DN15499_c0_g1_i1_2	15.01201	13.98734
DN154_c0_g1_i1_1	10.93136	11.7384
DN15501_c0_g1_i1_2	5.050373	0.9563901
DN15504_c0_g1_i1_2	4.722508	3.183286
DN15507_c0_g1_i1_2	4.771196	2.530473
DN15509_c0_g1_i1_1	0.9179746	4.841269
DN1550_c0_g1_i1_2	3.264558	1.620571
DN15512_c0_g1_i1_1	6.726141	6.04221
DN15515_c0_g1_i2_2	0.6598006	0.2036375
DN15517_c0_g1_i1_2	4.415682	1.828095
DN15519_c0_g1_i1_1	17.01477	12.76759
DN15522_c0_g1_i1_1	0.0708516	1.504465
DN15523_c0_g1_i1_2	3.172419	45.45117
DN15525_c0_g1_i1_2	3.19047	0.3246507
DN15526_c0_g1_i1_2	1.646177	0.421503
DN15527_c0_g1_i1_2	3.225385	1.085384
DN15532_c0_g2_i1_1	0.9631501	0.7627418
DN15533_c0_g1_i2_2	6.583772	2.512618
DN15539_c0_g1_i1_1	1.010106	0.7178239
DN15540_c0_g1_i1_1	1.236374	3.497099
DN15541_c0_g1_i1_1	0.2245748	2.718135
DN15546_c0_g1_i1_2	2.544929	1.450965
DN15549_c0_g1_i1_1	5.730061	7.367478

DN15551_c0_g1_i2_2	4.133368	1.665091
DN15551_c0_g2_i1_1	2.109773	1.360244
DN15551_c0_g2_i1_2	5.65942	3.000563
DN15552_c0_g1_i1_1	0.6245706	2.173343
DN15552_c0_g1_i1_2	2.321854	3.060969
DN15553_c0_g1_i2_1	3.445192	2.092313
DN15554_c0_g1_i1_1	1.725409	3.175393
DN15555_c0_g1_i1_2	1.150802	1.313739
DN15555_c0_g1_i2_1	3.461385	27.41339
DN15556_c0_g1_i1_1	0.1794849	1.874068
DN15556_c0_g2_i1_1	0.06530966	4.012868
DN15565_c0_g1_i1_1	0.7536641	3.028806
DN15566_c0_g1_i1_1	14.27641	37.8688
DN15566_c0_g1_i1_2	3.52171	1.298236
DN15566_c0_g2_i1_2	23.98832	10.4904
DN15568_c0_g1_i1_1	1.497693	1.483955
DN15568_c0_g1_i1_2	2.097433	0.3320455
DN15570_c0_g1_i1_2	0	0
DN15575_c0_g1_i1_2	6.491178	16.61545
DN15577_c0_g1_i1_1	2.467324	1.869792
DN15578_c0_g1_i2_2	3.088219	0.1023264
DN15580_c0_g1_i1_2	2.268256	1.966867
DN15587_c0_g1_i1_2	3.61555	6.474101
DN15588_c0_g1_i1_2	3.744943	2.059837

DN1558_c0_g1_i1_1	1.670587	0
DN15596_c0_g1_i1_1	2.348201	5.943138
DN15596_c0_g2_i1_2	3.563692	0.8521835
DN15598_c0_g1_i1_2	3.20179	3.459729
DN15599_c0_g1_i1_1	2.480806	6.297929
DN15600_c0_g1_i1_2	4.780733	2.321293
DN15609_c0_g1_i1_2	0	0
DN15612_c0_g1_i1_2	7.414241	3.244549
DN15613_c0_g3_i1_2	0.5408165	2.49E-14
DN15620_c0_g1_i1_2	0.7567747	1.75E-24
DN15620_c0_g4_i1_2	2.157308	1.551592
DN15622_c0_g1_i1_1	1.900013	1.988381
DN15625_c0_g1_i1_2	4.335701	0.7858617
DN15625_c0_g2_i1_1	0.8428881	0
DN15630_c0_g1_i2_1	6.648209	5.39387
DN15631_c0_g1_i2_1	2.750785	1.661821
DN15635_c0_g1_i2_1	1.926882	2.06849
DN15636_c0_g1_i2_1	1.383663	1.646875
DN1563_c0_g1_i1_1	0.0873567	0.5664434
DN15640_c0_g1_i1_1	11.87294	4.690553
DN15640_c0_g1_i1_2	0	0
DN15642_c0_g1_i1_1	1.887432	2.213197
DN15644_c0_g1_i1_2	7.856539	4.862389
DN15651_c0_g1_i1_2	2.272662	2.878392

DN15653_c0_g1_i1_1	2.302534	1.760934
DN15654_c0_g1_i1_2	3.573835	3.572104
DN15658_c0_g1_i1_2	2.533225	2.031223
DN1565_c0_g1_i1_1	2.51666	2.407181
DN15661_c0_g1_i1_1	0.5328763	10.53133
DN15667_c0_g1_i1_1	2.210311	2.354071
DN1566_c0_g1_i1_1	1.950818	3.852194
DN15670_c0_g1_i1_1	0.8898662	2.728986
DN15676_c0_g3_i1_2	2.790275	1.177308
DN15678_c0_g1_i1_2	1.041615	0.1509974
DN15678_c0_g1_i2_1	0.2562814	5.136594
DN15678_c0_g2_i1_2	1.701961	0.7423004
DN1567_c0_g1_i1_1	1.614812	3.628873
DN15685_c0_g1_i1_2	0.5768673	0.09447269
DN15686_c0_g1_i1_2	3.686114	4.816752
DN15689_c0_g1_i1_1	2.415812	5.449243
DN15692_c0_g1_i1_1	14.02417	24.5498
DN15696_c0_g1_i1_1	2.113705	1.942447
DN1569_c0_g1_i1_1	4.398045	24.1407
DN15701_c0_g1_i2_2	2.591245	1.043898
DN15707_c0_g1_i1_1	2.070638	2.613719
DN15709_c0_g1_i1_2	5.257746	0.8173351
DN15710_c0_g2_i1_1	1.570709	2.306713
DN15711_c0_g2_i1_1	0.9404527	1.353109

DN15712_c0_g1_i1_1	15.91959	96.38332
DN15712_c0_g1_i1_2	9.604455	8.912957
DN15713_c0_g1_i1_2	2.657653	1.849282
DN15717_c0_g1_i1_2	2.905842	1.864449
DN15718_c0_g2_i2_2	6.131697	0.1335008
DN15720_c0_g1_i1_1	1.386628	1.973861
DN15726_c0_g1_i1_2	4.666972	2.284805
DN1572_c0_g1_i1_1	27.89724	34.01736
DN15730_c0_g1_i1_1	3.241024	20.13115
DN15731_c0_g1_i2_1	0.03922285	3.438344
DN15734_c0_g1_i1_1	297.7871	117.2904
DN15739_c0_g1_i2_1	6.101235	17.13735
DN15739_c0_g1_i3_2	4.754635	1.705841
DN15743_c0_g1_i1_1	0.2114168	2.317274
DN15743_c0_g1_i1_2	1.757395	1.158774
DN15744_c0_g1_i1_1	3.189477	7.32024
DN1574_c0_g1_i1_1	3.81447	7.251595
DN15761_c0_g1_i1_1	7.273015	7.679573
DN15777_c0_g1_i2_2	4.991231	4.690973
DN15782_c0_g1_i1_2	1.469546	0.4289055
DN15784_c0_g1_i1_1	0.03165808	5.62947
DN15785_c0_g1_i1_2	1.301872	1.454961
DN15786_c0_g1_i1_2	3.760294	3.388681
DN15796_c0_g1_i1_1	3.104077	12.77752

DN15797_c0_g1_i6_2	2.341301	0
DN15808_c0_g1_i1_2	4.166731	2.924201
DN15809_c0_g1_i2_1	2.150331	1.549234
DN15809_c0_g2_i1_1	0.7631495	2.667912
DN15810_c0_g1_i1_1	2.712748	2.155565
DN15815_c0_g1_i1_1	0.2483622	2.523679
DN15824_c0_g1_i1_2	1.284901	1.493995
DN15824_c0_g2_i1_2	3.619542	2.620501
DN15824_c1_g1_i1_2	1.237141	1.069753
DN15825_c0_g1_i2_2	26.30736	17.99261
DN15826_c0_g1_i1_2	1.565393	1.524055
DN15827_c0_g1_i2_2	0.6828256	0.385227
DN15827_c0_g2_i1_2	0.2573775	1.875096
DN15828_c0_g1_i1_1	8.315661	31.92076
DN15828_c0_g1_i1_2	3.585704	4.377368
DN15829_c0_g1_i1_2	1.219505	7.8562
DN15830_c0_g1_i1_1	1.039374	3.092118
DN15831_c0_g6_i1_1	1.846912	1.148823
DN15833_c0_g1_i2_1	23.18432	48.22211
DN15833_c0_g2_i1_2	7.700055	1.288407
DN15841_c0_g1_i1_2	5.783565	2.080435
DN15847_c0_g1_i1_1	22.35312	9.350798
DN15848_c0_g1_i1_1	1.203032	0.8236506
DN15849_c0_g1_i1_2	3.944262	4.982524

DN1584_c0_g1_i1_1	2.916932	2.25659
DN15851_c0_g2_i1_2	1.848288	2.309401
DN15861_c0_g1_i1_1	3.383934	5.583687
DN15864_c0_g1_i2_1	4.725949	54.66507
DN1586_c0_g1_i1_1	1.29937	0.8733355
DN15870_c0_g1_i1_1	1.464987	1.020552
DN1587_c0_g1_i1_2	3.250081	2.054176
DN15885_c0_g1_i1_1	1.25295	2.494235
DN15885_c0_g1_i1_2	3.166528	0.3351669
DN15898_c0_g1_i1_1	3.285484	9.597589
DN15899_c0_g1_i1_1	0.6987885	5.789008
DN15902_c0_g1_i1_2	5.106546	1.475456
DN15907_c0_g1_i1_2	13.27598	20.93659
DN1590_c0_g1_i1_2	1.778688	0.3661921
DN15910_c0_g1_i2_1	1.577998	1.822044
DN15911_c0_g1_i4_1	0.8177982	1.561713
DN15916_c0_g1_i2_1	4.620277	3.58249
DN15927_c0_g1_i1_1	6.154843	4.179782
DN1592_c0_g1_i1_1	1.805704	5.546925
DN15931_c0_g1_i1_1	2.981962	2.715375
DN15935_c0_g1_i1_1	0.7243582	3.12359
DN15935_c0_g1_i1_2	2.118116	0.4555784
DN15935_c0_g2_i1_1	1.139201	1.885002
DN15936_c0_g1_i1_2	0.7706952	0.6786986

DN15937_c0_g1_i1_2	43.78039	28.71388
DN15938_c0_g1_i1_1	1.589988	1.512077
DN15939_c0_g1_i1_2	22.55767	4.101204
DN15940_c0_g2_i1_2	5.035925	1.655934
DN15949_c0_g1_i1_1	1.803764	4.030621
DN15955_c0_g1_i1_1	2.986367	6.141422
DN15956_c0_g1_i1_2	2.484917	0
DN15957_c0_g1_i1_1	1.122208	2.889633
DN15957_c0_g1_i1_2	2.91523	11.52386
DN15959_c0_g1_i1_1	1.852195	1.55132
DN15961_c0_g1_i1_2	2.209281	1.138329
DN15963_c0_g1_i1_2	5.310333	1.415318
DN15967_c0_g1_i1_1	0.613829	0.8604719
DN15969_c0_g1_i1_1	2.246778	7.179402
DN1596_c0_g1_i1_2	0.1483529	0.6380772
DN15972_c0_g1_i1_1	1.162317	2.389344
DN15987_c0_g1_i1_2	4.612424	0.7275846
DN15991_c0_g1_i1_2	0.7366382	0.8035163
DN15993_c0_g1_i1_2	4.931112	5.359266
DN15994_c0_g1_i1_1	1.559323	3.310848
DN15995_c0_g1_i1_1	1.793788	3.439819
DN15997_c0_g1_i1_1	0.9881375	2.054824
DN16003_c0_g2_i1_2	33.36963	16.15089
DN16005_c0_g1_i2_1	3.447147	2.946721

DN16006_c0_g1_i40_1	40.82496	29.46118
DN16010_c0_g1_i2_1	17.08928	58.8751
DN16016_c0_g1_i1_1	0.706479	6.091851
DN16018_c0_g1_i1_1	4.674671	6.09871
DN16020_c0_g1_i2_1	2.073483	3.346482
DN16023_c0_g1_i2_1	2.711028	4.589503
DN16024_c0_g2_i1_2	2.530279	0.9750358
DN16028_c0_g1_i1_1	6.76292	11.62886
DN16030_c0_g3_i1_1	1.480054	1.522485
DN16030_c0_g6_i1_1	0.491493	1.04733
DN16031_c1_g4_i1_1	1.173914	2.522955
DN16032_c0_g1_i1_1	3.13765	2.282219
DN16033_c0_g1_i1_1	2.106844	4.786459
DN16037_c0_g1_i2_2	3.544334	2.227422
DN16038_c0_g2_i2_2	5.571376	2.499265
DN16041_c0_g1_i1_1	1.067787	1.815265
DN16042_c0_g1_i1_1	1.410154	3.440633
DN16043_c0_g2_i1_1	0.591223	2.293497
DN16046_c0_g1_i2_2	2.230781	0.4398212
DN16056_c0_g1_i1_1	12.90446	17.48544
DN16056_c0_g1_i1_2	13.16196	10.07232
DN16058_c0_g1_i1_1	0.09989196	4.244342
DN16063_c0_g1_i1_1	0.9040609	4.56825
DN16066_c0_g1_i1_2	17.85736	6.43857

DN16068_c0_g1_i2_1	2.809828	3.137298
DN16081_c0_g1_i1_2	1.558259	3.30528
DN16087_c0_g1_i1_1	0.1805121	1.364441
DN16088_c0_g2_i1_1	1.958066	1.994513
DN1608_c0_g1_i1_2	7.402162	3.283237
DN16093_c0_g1_i1_2	1.049441	1.619318
DN16095_c0_g1_i1_1	28.4361	26.27964
DN16098_c0_g1_i1_1	0.6992353	5.83679
DN16099_c0_g1_i1_2	2.277231	0.7116404
DN1609_c0_g1_i1_1	5.225052	3.603336
DN160_c0_g1_i1_1	3.701312	3.603411
DN160_c0_g1_i1_2	1.974341	1.101026
DN16103_c0_g1_i1_2	2.224355	2.576225
DN16105_c0_g1_i1_1	1.928947	3.589805
DN16106_c0_g1_i1_2	3.208033	0.9589047
DN16109_c0_g1_i1_1	8.304498	30.94383
DN16109_c0_g1_i2_2	5.679228	0.9163618
DN16110_c0_g1_i1_1	1.425224	4.487984
DN16113_c0_g1_i2_1	1.650121	1.504001
DN16114_c0_g1_i1_1	0.9661567	1.236563
DN16118_c0_g1_i7_1	10.82438	11.25958
DN16120_c0_g1_i1_2	1.853763	0.8158178
DN16121_c0_g2_i1_2	1.769502	2.397852
DN16121_c0_g3_i1_2	0.8536996	2.429081

DN16125_c0_g1_i1_2	0.9791246	0.586859
DN16127_c0_g1_i1_1	1.00557	1.988384
DN16129_c0_g1_i1_1	16.97241	4.708878
DN16131_c0_g1_i1_1	0.199863	3.912297
DN16135_c0_g1_i1_1	20.87695	8.342983
DN16140_c0_g1_i1_1	1.572093	3.05081
DN16141_c0_g1_i1_1	0.04020136	3.822948
DN16146_c0_g1_i1_1	1.094131	1.259126
DN16148_c0_g1_i1_2	1.558244	0.9349438
DN16149_c0_g1_i1_1	3.580818	4.329477
DN16155_c0_g1_i1_2	5.596545	2.966188
DN16160_c0_g1_i2_2	2.337025	3.419611
DN16164_c0_g1_i1_2	0.575285	0.3703802
DN16166_c0_g4_i2_1	3.908516	2.289432
DN1616_c0_g1_i2_1	1.810219	4.428661
DN16171_c0_g1_i1_1	0.9630672	2.405918
DN16179_c0_g1_i1_2	10.68501	0.8277223
DN16181_c0_g1_i1_1	6.0658	3.34408
DN16185_c0_g1_i1_1	1.74564	5.499549
DN16190_c0_g1_i1_1	15.88729	9.036535
DN16192_c0_g1_i2_2	5.26291	8.188536
DN16197_c0_g1_i2_2	6.990298	2.639456
DN16197_c0_g2_i1_2	0.2561557	0.411934
DN16200_c0_g1_i2_1	2.064862	3.601597

DN16203_c0_g1_i1_1	2.485497	2.379119
DN16212_c0_g2_i1_1	4.721473	3.238616
DN16217_c0_g1_i1_2	4992.983	1.639849
DN16222_c0_g1_i1_1	1.256442	10.83965
DN16229_c0_g1_i1_1	41.24297	16.02309
DN16236_c0_g1_i1_1	7.709925	18.93366
DN16240_c0_g2_i1_2	2.680257	4.981198
DN16242_c0_g1_i1_1	3.618076	4.423997
DN16246_c0_g1_i1_2	3.170644	1.031847
DN16259_c0_g1_i1_2	32.9398	9.272959
DN16260_c0_g1_i1_1	3.33051	6.110166
DN16264_c0_g1_i1_1	5.596709	80.04646
DN16264_c1_g1_i1_2	4.296945	1.123489
DN16268_c0_g1_i1_1	0.5621704	5.940277
DN16268_c0_g2_i1_1	1.583098	3.364476
DN16269_c0_g1_i1_1	0.9482654	2.402802
DN16269_c0_g2_i1_1	2.250212	3.280576
DN16269_c0_g3_i1_1	1.295223	2.18041
DN16270_c0_g1_i1_2	7.034922	40.26745
DN16271_c0_g2_i1_1	2.844903	3.970747
DN16277_c0_g1_i2_1	1.084693	2.065095
DN16277_c0_g2_i1_1	1.303603	0.7869151
DN16280_c0_g1_i1_2	2.102148	1.496949
DN16285_c0_g1_i1_1	1.08432	1.188576

DN16285_c0_g1_i1_2	1.388463	0.554506
DN16285_c0_g2_i2_2	1.767877	0.8022027
DN16285_c0_g3_i1_1	0.1417498	1.028235
DN16287_c0_g1_i1_2	0.7722075	0.3431722
DN1628_c0_g1_i1_1	1.374398	2.755559
DN16290_c0_g1_i2_2	1.444162	0.04047545
DN16297_c0_g1_i1_2	0.1603323	0.8576497
DN16297_c0_g2_i1_1	2.97928	3.550474
DN16302_c0_g1_i1_2	21.76679	13.06181
DN16305_c0_g1_i1_1	4.345774	3.298684
DN16306_c0_g1_i1_2	7.076731	7.524588
DN16307_c0_g1_i1_2	0.7936224	1.91109
DN16308_c0_g1_i1_1	2.001095	4.270962
DN16309_c0_g1_i1_1	18.75235	13.87615
DN16311_c0_g1_i1_1	5.435502	17.90061
DN16314_c0_g1_i1_1	1.637048	2.250213
DN16319_c0_g1_i1_2	10.23644	14.5923
DN1631_c0_g2_i1_1	4.658885	29.39942
DN16323_c0_g1_i2_1	0.4812504	0.1853263
DN16324_c0_g1_i1_2	1.961824	1.773611
DN16326_c0_g1_i1_1	1.74546	5.52095
DN1632_c0_g1_i1_1	3.635578	4.754373
DN16334_c0_g1_i1_1	5.949771	7.771262
DN16339_c0_g1_i2_1	9.429046	5.435679

DN16343_c0_g1_i1_2	4.205003	1.717064
DN16344_c0_g1_i1_1	0.3884431	1.241511
DN16345_c0_g1_i1_2	5.234161	0.9020317
DN16346_c0_g1_i1_1	0.7224103	1.968993
DN16346_c0_g1_i2_2	254.574	0
DN1634_c0_g1_i2_1	1.469653	3.334155
DN16353_c0_g1_i1_1	1.310103	2.860502
DN16355_c0_g1_i1_2	13.2044	10.56678
DN16358_c0_g1_i1_1	2.849823	5.464189
DN1635_c0_g1_i1_1	0.9983516	1.89243
DN16360_c0_g1_i1_1	0.4252947	1.832379
DN16363_c0_g1_i1_2	1.441622	0.9998883
DN16364_c0_g1_i1_1	0.8700677	1.568538
DN16364_c0_g3_i2_1	1.311844	1.044677
DN16365_c0_g1_i1_1	1.212608	1.924335
DN16368_c0_g2_i2_2	5.103602	0.8619298
DN16373_c0_g1_i1_1	5.678598	4.633524
DN16373_c0_g1_i1_2	62.05389	31.43042
DN16374_c0_g1_i1_1	2.765156	2.018548
DN16375_c0_g1_i1_1	0.9011226	1.469074
DN16377_c1_g1_i2_2	3.841805	0.1992383
DN16377_c1_g2_i1_2	5.596908	1.258794
DN16377_c1_g3_i1_2	45.51803	21.92336
DN16378_c0_g1_i2_1	1.933196	2.839181

DN16379_c0_g1_i1_1	1.522585	3.534725
DN16380_c0_g1_i1_1	4.640223	3.751026
DN16384_c0_g1_i1_1	11.75391	4.491048
DN16386_c0_g2_i2_2	2.406194	2.409584
DN16386_c0_g4_i1_2	2.25724	1.449092
DN16387_c0_g1_i1_2	0.8653345	0.4933116
DN16388_c0_g1_i1_2	3.980412	2.522707
DN1638_c0_g1_i1_2	4.308501	6.364101
DN16398_c0_g1_i2_2	7.877474	4.206636
DN16401_c0_g1_i1_1	1.193718	0.7459576
DN16403_c0_g1_i1_2	12.46954	5.394219
DN16409_c0_g1_i1_2	0.7346895	0.1611509
DN16413_c0_g1_i1_2	4.224445	6.85856
DN16432_c0_g1_i5_1	1.339373	1.22552
DN16432_c0_g2_i3_1	1.734074	1.743742
DN16437_c0_g1_i2_1	1.695798	1.13766
DN16437_c0_g1_i2_2	2.59045	1.330221
DN16437_c0_g2_i3_2	2.485371	0.6282251
DN16438_c0_g1_i1_2	1.165996	1.705576
DN16440_c0_g1_i1_1	0.2039521	12.95589
DN16443_c0_g1_i1_2	4.422467	0.6653917
DN16449_c0_g1_i1_1	1.407685	2.335324
DN16449_c0_g1_i2_2	8.767122	6.970696
DN16452_c0_g1_i1_1	2.734933	2.993997

DN16461_c0_g1_i1_1	2.192279	7.272558
DN16466_c0_g1_i1_2	17.77337	16.47599
DN16466_c0_g1_i4_1	7.639663	7.792982
DN16468_c0_g1_i1_1	1.114347	0.7713924
DN1646_c0_g1_i2_2	1.443144	1.172734
DN16470_c0_g1_i1_1	1.257479	2.89883
DN16472_c0_g1_i1_1	4.172279	15.85349
DN16472_c0_g1_i1_2	2.559074	0.5830002
DN16477_c0_g1_i1_1	1.50717	2.308478
DN16481_c0_g1_i1_1	0.286251	4.726093
DN16483_c0_g1_i1_1	1.854428	1.158122
DN16483_c0_g2_i1_1	1.720625	1.482662
DN16486_c0_g2_i1_1	1.34369	0.5433421
DN16487_c0_g1_i1_2	1.093959	4.30E-46
DN16487_c0_g2_i1_2	1.436135	1.100273
DN16488_c0_g1_i1_1	2.400061	4.286907
DN1648_c0_g1_i1_1	14.74362	15.46513
DN16492_c0_g3_i1_2	1.95411	0.4731081
DN16493_c0_g1_i1_2	2.518251	0.7398677
DN16497_c0_g1_i1_1	1.043746	1.46129
DN16497_c0_g2_i1_1	1.067317	2.528559
DN16497_c0_g2_i1_2	4.249843	3.54122
DN16499_c0_g1_i1_2	0.4598057	0.06445418
DN16500_c0_g1_i1_2	0.7364467	0.3902948

DN16501_c0_g1_i1_2	4.516386	0.2020391
DN16502_c0_g1_i1_1	0.6354739	4.140713
DN16509_c0_g1_i1_2	8.327288	3.058775
DN16509_c0_g1_i2_1	0.2412008	1.545206
DN1650_c0_g1_i1_1	1.095107	1.840803
DN16518_c0_g1_i1_1	0.184817	7.06894
DN16520_c0_g1_i1_1	11.9344	4.192126
DN16523_c0_g1_i1_2	1.706871	0.841689
DN16528_c0_g1_i2_2	30.52659	47.08568
DN1652_c0_g1_i1_1	62.65331	34.80851
DN16533_c0_g2_i1_2	0.7668095	0.7009892
DN16534_c0_g1_i1_2	29.92474	43.45194
DN16535_c0_g1_i1_2	6.154308	4.712976
DN16537_c0_g2_i1_1	0.4425328	0.4210931
DN16538_c0_g1_i1_1	4.567741	2.90107
DN16545_c0_g1_i1_2	3.84419	1.301361
DN16546_c0_g2_i1_1	1.339588	1.806109
DN16558_c0_g1_i1_1	0.3467686	7.91752
DN16567_c0_g1_i1_1	1.001994	1.594791
DN16570_c0_g1_i1_2	4.392345	4.270143
DN16574_c0_g1_i1_2	1.798446	0.3896567
DN16576_c0_g1_i4_1	2.5696	2.417027
DN16577_c0_g1_i1_1	0.4429603	15.26683
DN16577_c0_g2_i3_1	0.6237707	30.166

DN16578_c0_g1_i1_2	6.815857	7.382286
DN16581_c0_g1_i1_2	2.030998	1.716903
DN16587_c0_g1_i1_2	3.649261	2.807387
DN16589_c0_g1_i1_1	4.18402	2.955322
DN16590_c0_g1_i2_2	10.25733	3.763585
DN16590_c0_g3_i1_1	4.22E-09	1.285265
DN16595_c0_g1_i1_1	6.522701	9.075596
DN16596_c0_g1_i1_1	0.4851336	0.6003864
DN16596_c1_g1_i1_1	1.68188	2.303615
DN16599_c0_g1_i1_1	0.1203816	4.55E-24
DN16599_c0_g2_i1_1	1.559449	1.239307
DN16603_c0_g1_i2_2	0.9158785	1.726065
DN16604_c0_g1_i2_2	2.386601	0.7826224
DN16609_c0_g1_i1_1	1.264946	2.052115
DN16610_c0_g3_i1_1	1.910436	1.312182
DN16612_c0_g1_i1_1	2.391774	0.7264295
DN16614_c0_g1_i3_2	4.109374	1.907459
DN16615_c0_g1_i1_1	0.6765098	2.27127
DN16617_c0_g1_i1_1	0.7631702	6.254413
DN16618_c0_g1_i1_1	1.133068	1.364386
DN16620_c0_g1_i1_2	2.472604	1.208885
DN16622_c0_g2_i1_2	1.211216	0.9635217
DN16622_c0_g3_i1_2	0.8982174	0.2942067
DN16623_c0_g1_i1_2	4.75826	5.142901

DN16629_c0_g1_i2_2	2.790502	3.923933
DN16630_c0_g1_i1_1	0.5314776	3.296579
DN16630_c1_g1_i1_2	1.294858	0.3178274
DN16633_c0_g1_i1_1	1.761309	2.658911
DN16638_c0_g1_i1_1	4.086923	16.05681
DN16640_c0_g1_i2_1	3.095292	1.885702
DN16641_c0_g1_i1_2	3.304738	1.416748
DN16642_c0_g1_i1_1	6.702194	2.005861
DN16644_c0_g1_i2_1	3.931451	2.567817
DN16657_c0_g1_i1_1	0.3803478	0.8258563
DN16661_c0_g1_i1_1	0.09051022	6.692873
DN16665_c0_g1_i2_2	2.098066	6.112004
DN16675_c0_g1_i1_1	1.533092	2.246134
DN16681_c0_g1_i1_2	3.155461	2.176067
DN16687_c0_g1_i1_2	15.88839	59.88917
DN16688_c0_g1_i1_2	3.216509	2.452818
DN16699_c0_g1_i1_1	0.2133343	2.993617
DN16699_c0_g1_i3_2	5.903479	11.41678
DN166_c0_g1_i1_2	3.622998	0.99941
DN16703_c0_g1_i1_1	2.412373	2.703283
DN16703_c0_g2_i1_1	2.669451	3.808411
DN16718_c0_g1_i2_1	1.665519	3.206951
DN16719_c0_g1_i1_1	0.8045548	1.203552
DN16720_c0_g1_i1_1	1.443714	3.808057

DN16728_c0_g1_i1_2	8.94016	17.84878
DN1672_c0_g1_i1_1	0.8688075	2.378972
DN16735_c0_g1_i1_2	1.975787	2.781402
DN16735_c0_g3_i1_2	3.375942	2.56028
DN1673_c0_g1_i1_2	0	0
DN16742_c0_g1_i2_1	1.127188	1.398743
DN16744_c0_g1_i1_1	0.2409697	3.773842
DN16747_c1_g4_i1_1	0.1811359	0.7447059
DN1674_c0_g1_i1_2	3.374648	0.2928585
DN16752_c0_g1_i1_2	3.586946	5.676627
DN16753_c0_g1_i1_2	6.331216	7.01835
DN16755_c0_g1_i1_1	0.9545237	1.101468
DN16755_c0_g2_i1_1	3.294187	4.765651
DN16756_c0_g1_i1_1	2.385755	8.231146
DN16767_c0_g1_i1_2	2.461239	3.713104
DN1676_c0_g1_i1_1	4.114235	2.532166
DN16771_c0_g1_i1_2	5.970279	9.274634
DN16778_c0_g2_i1_1	0.8468559	1.527566
DN16781_c0_g1_i1_1	5.435886	2.689247
DN16781_c0_g1_i1_2	38.9379	61.06873
DN16788_c0_g1_i1_1	2.563172	1.730964
DN16792_c0_g1_i1_1	1.000512	0.6040295
DN16792_c0_g2_i1_1	1.407801	0.8465756
DN16797_c0_g1_i2_1	5.071927	7.286728

DN167_c0_g1_i1_1	1.581466	1.578021
DN16802_c0_g1_i1_2	8.436796	1.548245
DN16808_c0_g2_i1_1	1.517897	3.799602
DN16813_c0_g1_i1_2	2.310201	1.026277
DN16813_c0_g2_i1_2	3.645744	1.548019
DN16816_c0_g1_i1_2	1.478626	0.9691337
DN16822_c0_g3_i1_1	16.03014	8.130406
DN16822_c0_g6_i1_1	0.1381265	0.3914608
DN16822_c1_g6_i1_1	11.03679	7.349171
DN16828_c0_g1_i2_1	1.073773	5.844289
DN16830_c0_g1_i1_1	1.859619	2.262724
DN16833_c3_g1_i1_2	0	0
DN16837_c0_g1_i1_1	9.828385	10.44406
DN16840_c0_g1_i2_2	15.26635	7.608013
DN16844_c0_g1_i1_1	1.792196	1.155549
DN16846_c0_g1_i1_2	2.40204	1.845981
DN16848_c0_g1_i3_1	5.838526	17.07277
DN1684_c0_g1_i1_1	0.5652351	1.281014
DN16850_c0_g1_i1_1	1.850307	11.92651
DN16851_c0_g1_i1_1	0.08377652	3.203996
DN16852_c0_g1_i7_1	2.163329	1.904747
DN16859_c0_g1_i1_1	13.02012	50.4997
DN16859_c0_g1_i1_2	3.733371	1.147557
DN16859_c0_g2_i1_2	2.256906	1.285617

DN16862_c0_g2_i1_1	3.349864	1.336347
DN16863_c0_g1_i1_1	29.99557	8.785363
DN16863_c0_g1_i1_2	1.733309	3.515258
DN16872_c0_g1_i1_2	6.126601	6.994821
DN16876_c0_g2_i1_2	4.126903	1.772945
DN16879_c0_g2_i1_2	2.862001	0.8499539
DN16879_c0_g3_i1_2	1.321458	0.4892676
DN16882_c0_g1_i1_1	2.584134	3.369184
DN16884_c0_g1_i1_1	1.532168	4.050383
DN16884_c0_g1_i1_2	1.119436	0.5376536
DN16889_c0_g1_i1_1	15.9877	47.68355
DN16892_c0_g1_i1_2	2.737868	1.97754
DN16898_c0_g2_i1_1	1.731388	2.274537
DN16901_c0_g2_i1_1	13.57535	8.974729
DN16903_c0_g1_i1_1	24.60369	28.76243
DN16914_c0_g1_i1_2	3.263682	3.325056
DN16915_c0_g1_i1_2	3.259647	0
DN16924_c0_g1_i1_2	19.66498	106.6091
DN16926_c0_g1_i1_2	2.870754	0.8397232
DN16926_c0_g2_i1_2	2.068302	1.176953
DN16931_c0_g1_i1_1	0.9689889	2.82171
DN16931_c0_g2_i1_1	1.030898	1.773733
DN16938_c0_g1_i1_1	1.536852	1.516398
DN16939_c0_g1_i1_1	0.1031356	6.174361

DN16942_c0_g1_i1_1	3.46404	2.280391
DN16944_c0_g1_i1_1	3.558771	3.331218
DN16946_c0_g1_i1_2	5.460315	3.692202
DN16952_c0_g1_i1_1	8.49483	14.29349
DN16955_c0_g1_i2_2	1.565252	1.20553
DN16957_c0_g2_i1_1	0.9874871	2.73089
DN16960_c0_g1_i1_1	2.379807	5.364724
DN16967_c0_g1_i1_1	0.6805962	15.14845
DN16971_c0_g1_i1_2	3.733809	0.6392202
DN16971_c0_g2_i1_2	3.390135	0
DN16982_c0_g1_i1_2	0	0
DN16984_c0_g1_i1_1	113.5148	56.85005
DN16986_c0_g1_i1_1	1.858927	1.798642
DN16987_c0_g1_i1_1	3.512089	5.003099
DN16988_c0_g2_i1_1	2.229198	1.565331
DN16989_c0_g1_i1_1	2.963234	5.808315
DN16999_c0_g1_i1_2	8.420138	6.707815
DN1699_c0_g1_i1_1	0.3014572	1.098879
DN169_c0_g1_i1_2	2.139595	1.497645
DN17000_c0_g1_i1_2	2.575406	1.009868
DN17001_c0_g1_i1_1	2.099736	11.31661
DN17005_c0_g1_i1_1	3.051588	0.8108567
DN17008_c0_g1_i1_2	4.854167	1.280022
DN17009_c0_g1_i1_1	1.195711	3.349209

DN17010_c0_g1_i1_1	2.722829	1.995218
DN17012_c0_g1_i3_1	271.4223	74.72252
DN17012_c0_g2_i1_2	3.788745	2.256349
DN17017_c0_g1_i1_2	1.526429	1.995318
DN17022_c0_g1_i1_1	1.890215	2.046978
DN17025_c0_g1_i1_2	7.04046	7.225877
DN17027_c0_g1_i1_2	0.8576944	0.4039887
DN17027_c1_g1_i1_2	0.9486935	0.813032
DN17027_c1_g2_i1_2	13959.33	14555.92
DN17029_c0_g1_i1_1	4.878502	10.40529
DN17033_c0_g1_i1_1	0.9075835	1.521312
DN17033_c0_g3_i1_1	0.846566	1.900614
DN17033_c0_g4_i1_1	1.760244	1.482633
DN17033_c0_g5_i1_1	1.731533	0.602538
DN17037_c0_g1_i1_1	1.475689	1.712293
DN17037_c0_g2_i1_1	1.831392	2.602038
DN17038_c0_g1_i1_1	0.8297738	3.121631
DN17038_c0_g2_i1_1	0	2.573778
DN17039_c0_g1_i1_1	10.65856	5.971837
DN1703_c0_g1_i1_1	0.9351334	7.116294
DN17040_c0_g1_i2_1	2.832612	4.617118
DN17064_c0_g1_i1_1	5.99161	1.90896
DN17064_c0_g2_i1_2	0.7801278	1.576862
DN17069_c0_g1_i1_1	4.026315	4.507978

DN17076_c0_g2_i1_1	0.4845753	3.950086
DN17080_c0_g1_i1_1	3.80032	2.491876
DN17084_c0_g1_i1_2	3.622824	0.6918274
DN17091_c0_g3_i1_2	1.83927	0.4719674
DN17091_c1_g3_i1_2	2.34419	1.432672
DN17091_c2_g1_i1_2	2.756175	0.2973359
DN17095_c0_g1_i1_1	1.863066	3.316359
DN17095_c0_g1_i1_2	33.00593	8.028597
DN17097_c0_g1_i2_1	1.281237	6.221244
DN170_c0_g1_i1_1	6.509434	8.518732
DN17100_c0_g1_i1_2	1.803844	1.193017
DN17101_c0_g1_i1_2	1.48357	0.7825345
DN17101_c0_g2_i1_2	3.779008	0.6755531
DN17101_c0_g3_i1_1	0.9353944	0.5530583
DN17102_c0_g1_i1_2	4.35233	3.157862
DN17109_c0_g1_i1_1	4.652947	7.68458
DN17112_c0_g2_i2_1	0.6661301	5.738854
DN17113_c0_g3_i1_2	3.411873	0.4363033
DN17116_c0_g1_i1_2	6.079739	6.536172
DN17118_c0_g1_i3_1	1.323531	7.125614
DN17122_c0_g1_i1_1	1.939741	2.73918
DN17127_c0_g2_i1_1	2.140537	1.911157
DN17128_c0_g1_i1_2	4.420113	2.896001
DN17129_c0_g3_i1_1	0.3694577	1.905947

DN17130_c0_g1_i1_2	2.503192	2.433098
DN17135_c0_g1_i3_1	0.1886063	5.569529
DN17137_c0_g1_i1_2	2.826009	2.456053
DN17139_c0_g2_i1_2	3.296042	1.239056
DN17141_c0_g1_i1_2	9.111752	4.619497
DN17147_c0_g1_i2_1	4.043363	19.94984
DN17148_c0_g1_i4_2	1.901251	2.153344
DN17153_c0_g1_i1_2	1.321575	1.156578
DN17154_c0_g1_i1_2	3.003767	0.8077239
DN17156_c0_g1_i1_2	4.418217	1.581159
DN17156_c0_g2_i1_2	0.7244316	0
DN17157_c0_g1_i1_1	10.59321	5.105922
DN17163_c0_g1_i1_1	1.116235	0.8929098
DN17163_c1_g1_i1_1	1.439122	4.017138
DN17166_c0_g1_i1_1	0.4728442	0.9993715
DN17166_c0_g1_i2_2	4.724561	1.87852
DN17168_c0_g1_i1_1	2.371687	6.97216
DN17171_c0_g1_i1_2	23.49436	25.72493
DN17174_c0_g1_i2_1	1.568389	17.04769
DN17175_c0_g1_i1_1	1.046989	8.289405
DN17175_c0_g4_i1_1	0.5398238	2.161639
DN17175_c0_g5_i2_1	14.58678	14.44812
DN1717_c0_g1_i1_2	0.5950407	0.3342896
DN17186_c0_g1_i1_2	2.208554	0.7375106

DN17187_c0_g1_i1_1	3.859104	1.679378
DN17188_c0_g1_i1_1	1.79238	1.180573
DN17190_c0_g1_i1_2	2.630216	1.05655
DN17197_c0_g1_i1_1	2.275719	2.962211
DN17199_c0_g2_i1_1	1.639101	2.274874
DN171_c0_g1_i1_2	1.656662	0
DN17200_c0_g1_i1_2	4.445652	1.512598
DN17202_c0_g1_i1_1	2.481818	2.074035
DN17204_c0_g1_i1_2	5.206327	2.787677
DN17210_c1_g1_i1_1	15.19027	6.022444
DN17215_c0_g1_i1_2	2.184667	1.924627
DN17216_c0_g1_i1_2	1.846403	1.397312
DN17221_c0_g1_i1_1	1.20011	2.2019
DN17222_c0_g1_i2_1	0	1.428881
DN17222_c1_g1_i1_1	0.9331329	1.134382
DN17226_c0_g2_i1_1	2.297757	3.51969
DN17227_c0_g1_i2_2	0.788059	0.5305663
DN17236_c0_g2_i1_2	1.021605	0.7642658
DN17238_c0_g1_i2_1	2.462833	6.580455
DN17239_c0_g1_i1_1	0.4368433	1.431849
DN17253_c0_g1_i2_1	2.339211	6.603557
DN17255_c0_g1_i2_2	9.963551	2.281316
DN17257_c0_g2_i1_1	0.6726919	2.253978
DN17258_c0_g1_i1_1	0.4295333	3.398974

DN17258_c0_g2_i1_1	0.3651016	1.827432
DN17266_c0_g1_i1_2	2.856343	1.394767
DN17266_c0_g1_i2_1	5.337551	4.590709
DN1726_c0_g1_i1_2	0	0
DN17270_c0_g1_i1_1	0.6684121	3.238769
DN17275_c0_g1_i3_2	2.019451	6.856175
DN17275_c0_g3_i1_2	2.181623	6.144978
DN17279_c0_g1_i3_1	1.987772	2.340812
DN17280_c0_g1_i1_1	1.48596	3.511757
DN17282_c0_g1_i2_2	9.299414	9.969399
DN17285_c0_g1_i1_2	189.6353	3.462422
DN17286_c0_g1_i1_2	7.503721	5.861618
DN17287_c1_g3_i1_1	1.285721	6.895086
DN17296_c0_g1_i1_2	2.79512	0.2514155
DN17298_c0_g1_i2_2	2.070633	0.7350091
DN1729_c0_g1_i1_1	0	0.7647809
DN17300_c0_g1_i1_2	0.8643018	0.8726865
DN17304_c0_g1_i1_1	0.9821872	3.844438
DN17307_c0_g1_i2_1	0.9633577	2.775559
DN17310_c0_g1_i1_2	4.76526	4.618794
DN17312_c0_g1_i1_2	1.134046	1.508256
DN17314_c0_g1_i1_1	1.374422	3.463348
DN17320_c0_g1_i1_1	8.247964	4.469517
DN17324_c0_g1_i2_2	10.29702	3.548959

DN17324_c2_g2_i2_1	2.035055	0.8663049
DN17325_c0_g1_i1_2	2.06685	1.084295
DN17336_c0_g1_i1_2	18.31316	5.100962
DN17337_c1_g2_i1_1	2.289004	4.32774
DN17340_c0_g1_i1_1	1.160901	2.196069
DN17341_c0_g1_i1_1	3.778025	6.677133
DN17343_c0_g1_i2_2	86.35202	14.49477
DN17345_c0_g1_i1_1	3.9059	5.671419
DN17346_c0_g1_i2_1	1.458163	1.406248
DN17349_c0_g1_i1_2	11.4199	6.445553
DN1734_c0_g1_i1_2	6.005111	0
DN17353_c0_g1_i1_1	1.478885	4.403114
DN17358_c0_g1_i1_1	3.132718	2.219207
DN17364_c0_g1_i1_2	2.560636	3.282325
DN17366_c0_g1_i1_2	18.25801	3.339906
DN17366_c0_g2_i1_2	6.104852	2.080471
DN17374_c0_g1_i1_1	0.4013854	3.068058
DN17380_c0_g3_i4_1	5.017491	2.651427
DN17383_c0_g1_i2_2	1.282895	0.2799362
DN1738_c0_g1_i1_1	1.587117	1.269739
DN17392_c0_g1_i1_2	5.519839	3.964471
DN17393_c0_g2_i1_1	10.59887	55.78856
DN17396_c0_g1_i1_2	31.82157	22.29568
DN17400_c0_g2_i1_2	6.00E-18	0.323005

DN17402_c0_g2_i3_2	6.11787	1.767465
DN17411_c1_g1_i1_1	184.5392	55.86481
DN17419_c0_g1_i1_1	1.308706	2.766184
DN1741_c0_g1_i2_1	15.90755	23.20931
DN17422_c0_g2_i1_1	12.95546	8.273473
DN17424_c0_g1_i2_2	0	0
DN17425_c0_g1_i1_1	1.615613	1.796908
DN17428_c0_g1_i1_1	21.42194	18.05997
DN1742_c0_g1_i1_1	1.64099	1.9734
DN17430_c0_g1_i2_2	5.532083	2.763755
DN17431_c0_g1_i1_1	2.576366	7.514572
DN17434_c1_g2_i1_1	0.5212758	4.543678
DN17434_c1_g3_i1_1	0.5755238	1.201481
DN17434_c1_g4_i1_1	0.3752574	1.206971
DN17440_c0_g3_i1_1	22.76953	21.84332
DN17443_c0_g1_i1_1	1.894929	3.732656
DN17444_c0_g1_i1_1	1.795475	4.373561
DN1744_c0_g1_i1_1	33.62558	69.01559
DN17459_c0_g2_i1_1	2.418986	4.283071
DN1745_c0_g1_i1_1	1.413333	1.268615
DN17465_c0_g1_i1_1	0.5878307	1.23567
DN17470_c0_g1_i2_2	2.40338	2.00447
DN17472_c0_g2_i1_2	6500.97	4.111269
DN17473_c0_g1_i1_1	5.041782	4.905578

DN17475_c0_g1_i1_1	1.801438	3.919492
DN17476_c0_g1_i1_1	1.065428	4.395481
DN17480_c0_g1_i2_1	12.84092	7.15708
DN17485_c0_g1_i1_2	3.75646	1.199124
DN17490_c0_g1_i2_1	6.105657	6.056516
DN17494_c0_g1_i1_2	17.32766	10.91095
DN17494_c0_g1_i3_1	1.135407	11.85431
DN17502_c0_g1_i1_1	4.783452	23.82175
DN17503_c0_g1_i1_2	29.13794	14.52242
DN17503_c0_g2_i2_2	68.58257	0
DN17506_c0_g1_i1_2	1.487739	0.447103
DN17506_c2_g5_i1_1	1.174887	0.9923762
DN17509_c0_g1_i1_1	1.378102	4.079991
DN17510_c1_g3_i2_1	8.219504	5.764206
DN17511_c0_g1_i1_2	2.379159	1.323532
DN17511_c0_g1_i3_1	2.557048	3.046468
DN17516_c0_g1_i1_2	3.399071	5.709585
DN17519_c0_g1_i1_2	15.3788	166.1668
DN1751_c0_g1_i1_1	0.8371792	5.033861
DN1751_c0_g1_i1_2	3.07135	0.3912735
DN1751_c0_g2_i1_1	1.412216	1.933484
DN17521_c0_g1_i1_2	1.911694	1.105672
DN17522_c0_g1_i1_1	0.5723953	5.224136
DN17531_c0_g1_i2_2	6.973812	7.927441

DN1753_c0_g1_i1_1	0	1.240584
DN17543_c0_g1_i2_1	1.542483	2.468874
DN17544_c0_g1_i1_2	2.21821	0.243873
DN1754_c0_g1_i1_2	5.301876	2.735754
DN17550_c0_g1_i1_1	1.505756	0.5414272
DN17557_c0_g2_i1_2	6.320618	12.94907
DN17558_c0_g1_i1_1	1.070919	2.688321
DN17560_c0_g2_i1_1	1.508177	1.477768
DN17568_c0_g1_i1_1	1.433734	4.349794
DN17574_c0_g1_i2_2	15.41278	12.14254
DN17585_c0_g1_i1_1	5.363375	1.585711
DN17591_c0_g2_i1_2	0	0
DN17593_c1_g1_i6_1	1.256986	1.476515
DN17595_c0_g1_i2_2	1.002614	0.6855734
DN1759_c0_g1_i1_1	0.5381119	0.3870414
DN17601_c0_g2_i1_1	3.565165	0
DN17601_c1_g2_i3_1	6.158371	6.502011
DN17601_c1_g4_i1_1	0.4446969	1.279785
DN17609_c0_g1_i1_1	3.933783	3.868623
DN17616_c0_g1_i1_1	0.8208827	1.703765
DN17616_c0_g2_i1_1	1.142168	3.719302
DN17617_c0_g1_i2_2	8.163822	9.937877
DN17619_c0_g1_i1_2	1.341918	0.6700407
DN17627_c0_g1_i3_2	6.580251	2.958867

DN17627_c0_g1_i7_1	1.44542	2.831357
DN17627_c1_g1_i1_1	10.44423	25.19241
DN1762_c0_g1_i1_1	1.933203	1.102438
DN17632_c0_g1_i2_1	2.767754	9.722763
DN17642_c0_g1_i1_1	2.282521	5.291876
DN17649_c0_g1_i1_2	8.674212	3.059056
DN1764_c0_g1_i1_2	2.059446	1.485377
DN17651_c0_g1_i1_1	0.1006082	1.984297
DN17652_c0_g1_i1_1	2.315615	6.802489
DN17653_c0_g1_i2_1	0.4283292	0.2885048
DN17653_c0_g2_i1_1	1.759572	2.204383
DN17656_c0_g1_i3_1	0.3657678	3.34715
DN17659_c0_g1_i1_2	2.751409	3.842389
DN17660_c0_g1_i1_2	3.958547	1.805821
DN17664_c0_g1_i1_1	0.747964	9.107476
DN17667_c0_g1_i1_2	34.32571	8.288475
DN17672_c0_g1_i1_1	0.3699433	4.314097
DN17674_c0_g1_i1_2	3.498971	2.39571
DN17679_c0_g1_i1_2	7.594137	4.041965
DN17681_c0_g1_i1_1	1.269977	1.829818
DN17693_c0_g1_i1_2	1.459009	0.3191053
DN17693_c0_g2_i1_2	2.155212	0.4959508
DN17697_c0_g1_i1_2	2.779715	4.740444
DN17702_c0_g1_i1_1	10.26813	15.15548

DN17708_c0_g3_i1_1	2.286431	1.451064
DN17709_c0_g1_i1_2	4.281742	1.3578
DN1770_c0_g1_i1_1	0.8488395	0.7035858
DN17712_c0_g3_i2_1	1.479222	0.8666811
DN17714_c0_g1_i1_1	2.437941	5.430824
DN17715_c0_g1_i3_2	4.963517	4.71743
DN17716_c0_g1_i2_1	37.90075	65.27221
DN17721_c0_g1_i1_2	2.272309	0
DN17721_c0_g1_i5_1	1.785875	2.596768
DN17723_c0_g1_i1_1	12.97442	8.158341
DN17723_c0_g1_i1_2	2.092209	0.3375191
DN17731_c0_g1_i1_1	1.647885	11.38628
DN17736_c0_g1_i1_2	2.429654	1.90874
DN17736_c0_g1_i2_1	3.160565	2.959638
DN17736_c0_g2_i1_2	3.295688	0.4892175
DN17747_c0_g1_i1_2	0.9391875	1.444391
DN17757_c0_g3_i1_1	2.141821	3.62461
DN1775_c0_g1_i1_1	0.955429	0.9794681
DN17769_c0_g1_i1_1	0	10.25193
DN17769_c1_g1_i7_1	0.6249027	2.990153
DN17769_c1_g2_i1_1	0.2093408	7.053831
DN17770_c0_g1_i1_2	3.067436	1.290276
DN17773_c0_g1_i1_2	2.679691	1.319401
DN17774_c0_g1_i1_2	3.699264	2.657146

DN17783_c0_g1_i1_1	1.605647	4.417472
DN17784_c0_g1_i1_1	1.81884	1.676013
DN17784_c0_g2_i2_1	1.876207	1.25998
DN17786_c0_g1_i1_1	2.070605	4.535805
DN17789_c0_g1_i2_2	1.854151	1.813172
DN17790_c0_g1_i1_1	4.409911	3.962497
DN17793_c0_g1_i1_2	1.880989	0.679161
DN17798_c0_g1_i3_2	5.476314	2.850808
DN17802_c0_g1_i1_2	9.13964	6.715378
DN17802_c0_g1_i2_1	5.344132	6.913736
DN17803_c0_g1_i1_1	1.573264	2.056407
DN17804_c0_g2_i1_2	3.130916	2.09548
DN17805_c0_g1_i1_2	6.759698	10.60274
DN17808_c0_g1_i1_2	3.233249	1.331595
DN17808_c0_g2_i1_2	1.781331	1.151622
DN17809_c0_g1_i1_2	8.262761	3.534693
DN17813_c0_g1_i1_1	1.301371	2.476455
DN17816_c0_g1_i1_1	0.9316186	2.906564
DN17819_c0_g1_i1_2	2.438871	0.1807295
DN1781_c0_g1_i1_1	0.7341231	1.271698
DN1781_c0_g2_i1_1	0.3010649	1.617771
DN17835_c0_g1_i1_2	6.67144	5.399346
DN17835_c1_g3_i1_1	0.8619824	1.127838
DN17840_c0_g1_i2_2	23.7659	29.6858

DN17856_c0_g1_i1_1	2.497399	6.138618
DN17862_c0_g1_i1_2	9.619014	12.46526
DN17865_c0_g1_i2_1	4.050844	4.356176
DN17869_c0_g2_i1_2	1.914916	2.920284
DN17869_c0_g3_i1_2	8.96376	4.826496
DN1786_c0_g1_i1_1	3.365622	5.961841
DN17870_c0_g1_i2_1	1.478903	8.907129
DN17874_c0_g1_i2_2	4.914624	2.273033
DN17876_c0_g1_i1_2	10.83421	6.905281
DN17878_c0_g2_i3_1	25.00947	6.979939
DN1787_c0_g2_i1_2	2.895443	0.2184095
DN17893_c0_g1_i1_1	0.4533338	7.25748
DN17902_c0_g1_i1_2	1.615012	0
DN17902_c0_g2_i1_2	1.693519	0.9192344
DN17902_c0_g3_i1_2	2.131587	0.9530845
DN17903_c0_g1_i1_1	1.50675	3.949595
DN17909_c0_g1_i1_1	2.688017	4.149969
DN1790_c0_g1_i1_2	0.5193825	0.6249662
DN17910_c1_g2_i2_1	1.83438	4.29E-44
DN1791_c0_g1_i1_1	1.138337	0.8126248
DN1791_c0_g2_i1_1	1.776644	3.033321
DN17926_c0_g1_i1_2	2.05875	0.7873254
DN1792_c0_g1_i1_1	0.5375713	1.161587
DN17930_c0_g1_i1_1	6.621943	4.676724

DN17932_c0_g1_i1_2	3.216606	3.818742
DN1793_c0_g1_i1_1	1.046669	4.581315
DN17940_c0_g1_i4_2	36.88573	1.42799
DN17942_c0_g1_i1_1	2.883819	4.937238
DN17952_c0_g1_i1_1	0.2170744	0.229505
DN17958_c0_g1_i1_2	50.42098	0.09655729
DN1795_c0_g1_i1_1	0.4097662	0.3825751
DN17961_c0_g1_i1_1	0.6315757	0.8616366
DN17962_c0_g1_i1_1	1.802685	16.33632
DN17964_c0_g1_i2_1	3.669406	2.290436
DN17968_c0_g1_i1_2	15.75998	3.923757
DN17969_c0_g2_i1_2	4.878146	1.639606
DN17969_c0_g3_i1_2	5.348131	2.085147
DN17969_c1_g1_i1_2	4.038503	1.083831
DN1796_c0_g1_i1_1	13.28814	16.35193
DN17970_c1_g3_i1_2	2.673751	1.109381
DN17977_c0_g3_i1_1	0.5198417	0.2409622
DN17981_c0_g1_i1_2	2.223945	0.8229599
DN17985_c0_g1_i2_1	0.9156142	3.462859
DN17986_c0_g1_i1_1	0.9548741	0.8760719
DN17987_c0_g2_i1_2	31.29895	11.15646
DN17988_c0_g1_i2_1	0.4391485	1.62798
DN1798_c0_g1_i1_1	2.334465	5.85597
DN17994_c0_g1_i3_1	8.170515	4.871598

DN179_c0_g1_i1_1	1.861961	1.832988
DN18003_c0_g1_i1_1	1.101149	4.341396
DN18005_c0_g1_i1_1	35.43494	125.5275
DN1800_c0_g1_i1_1	1.554941	5.019972
DN18011_c0_g1_i1_1	2.465716	5.936451
DN18018_c0_g1_i1_1	1.830811	6.397196
DN18025_c0_g1_i1_2	10.74726	2.915439
DN18026_c0_g1_i1_1	1.346081	9.698739
DN18026_c0_g1_i4_2	4.517988	5.677156
DN18026_c0_g2_i1_2	6.436677	2.89175
DN18028_c0_g1_i1_2	7.72244	1.838239
DN18035_c0_g1_i1_1	0.4021109	2.222505
DN18042_c0_g1_i1_2	2.983399	1.838208
DN18043_c0_g1_i1_2	11.05379	3.658167
DN18057_c0_g1_i2_1	1.617238	1.799504
DN18062_c0_g1_i1_2	6.04096	1.663734
DN18063_c0_g1_i1_1	1.282948	5.463518
DN18069_c0_g1_i1_1	0.5619847	3.386816
DN18069_c0_g1_i1_2	2.702675	0.4329824
DN18071_c0_g1_i1_2	2.39387	1.680848
DN18071_c2_g1_i2_1	1.721151	1.805288
DN18071_c2_g2_i1_1	1.819326	0.3239923
DN18071_c3_g1_i3_1	2.407335	2.019449
DN18072_c0_g1_i1_2	3.694371	1.812867

DN18078_c0_g1_i1_1	1.299475	3.504381
DN18080_c0_g1_i1_1	9.811383	10.81934
DN18081_c0_g1_i2_1	28.81192	76.32273
DN18089_c0_g1_i1_1	1.187423	3.608897
DN18090_c0_g2_i1_2	3.502539	0.7282192
DN18093_c0_g1_i1_2	1.792919	1.093169
DN18093_c0_g1_i2_1	6.654237	12.29908
DN18095_c0_g1_i1_1	19.36735	8.551268
DN18105_c0_g1_i2_1	1.003276	2.494157
DN18111_c0_g1_i1_1	5.167012	5.238353
DN18112_c0_g1_i1_1	1.915705	4.436302
DN18119_c1_g1_i1_1	14.87323	6.329609
DN18120_c0_g1_i1_1	0.7523376	1.407299
DN18120_c0_g1_i2_2	10.15961	4.382966
DN18124_c0_g1_i3_2	2.026709	2.653797
DN18136_c0_g2_i1_2	3.989381	1.770894
DN1813_c0_g1_i1_1	0.2199605	1.401346
DN1814_c0_g1_i1_1	0.2967856	3.461963
DN18153_c0_g1_i2_2	7.425726	3.647954
DN18154_c0_g1_i1_1	2.734177	4.925984
DN18156_c0_g2_i1_1	1.223317	3.511033
DN18157_c0_g1_i1_2	2.541568	1.297732
DN18157_c0_g2_i1_2	4.540297	1.466886
DN18158_c0_g1_i1_1	1.407172	5.618368

DN1815_c0_g1_i1_1	1.34341	1.556819
DN18160_c0_g2_i1_2	2.785769	2.049853
DN18163_c0_g1_i31_1	8.274447	115.9907
DN18164_c0_g2_i1_1	1.843094	1.799852
DN18174_c0_g1_i1_2	33.52496	6.163805
DN18174_c1_g1_i1_2	69.78356	37.45746
DN18186_c0_g1_i1_1	0.3660987	2.077469
DN18187_c1_g4_i1_1	2.662299	4.900827
DN18192_c0_g2_i1_1	2.523073	4.523455
DN18195_c0_g1_i1_2	1.907099	1.339009
DN18200_c0_g1_i1_1	0.3809015	0.5547319
DN18204_c0_g1_i1_1	1.973276	15.2255
DN18209_c0_g1_i1_2	34.3682	15.56958
DN1820_c0_g1_i1_2	3.160056	2.101921
DN18212_c0_g1_i1_2	24.93282	5.952344
DN18214_c0_g2_i1_1	18.0987	8.423554
DN18216_c0_g1_i1_1	1.717674	1.052838
DN18218_c0_g10_i1_1	0.912823	0.4547671
DN18221_c0_g2_i2_1	2.10367	1.340262
DN18222_c0_g1_i1_1	1.918653	2.935812
DN1822_c0_g1_i1_1	1.7214	0.8334143
DN18235_c0_g1_i1_1	0.6413598	2.32598
DN18238_c0_g1_i1_2	9.56242	3.341556
DN18241_c0_g1_i1_1	0.4844617	2.178032

DN18243_c0_g1_i2_2	21.23852	10.81232
DN18246_c0_g1_i1_1	4.377771	3.680562
DN18250_c0_g3_i1_1	2.067242	0.106889
DN18257_c0_g1_i1_2	7.6236	3.181347
DN18260_c1_g3_i1_1	1.08214	0.109106
DN18260_c1_g6_i1_1	0.345118	2.10206
DN18262_c0_g1_i1_2	4.907143	5.435387
DN18262_c0_g5_i1_1	3.133079	6.93008
DN18267_c0_g1_i3_1	1.009489	2.589577
DN18268_c0_g1_i8_1	2.161478	0.8369405
DN1826_c0_g1_i1_1	0.9696297	1.369884
DN18274_c0_g1_i1_1	2.827192	3.256057
DN18277_c0_g1_i1_1	0.5296583	6.322696
DN18278_c0_g1_i1_1	12.72106	3.260084
DN1828_c0_g1_i1_2	0.5329381	0
DN18291_c0_g5_i1_1	0.8292548	3.356586
DN18291_c0_g7_i1_1	1.277034	1.435634
DN18292_c0_g2_i1_1	2.012849	2.123703
DN18293_c0_g1_i1_1	13.99711	17.66751
DN18299_c0_g1_i1_2	3.794148	1.837975
DN1829_c0_g1_i1_1	0.7494014	0.9035177
DN18309_c0_g2_i1_1	0.546145	2.053773
DN1830_c0_g1_i1_2	0.130519	0.3016347
DN18312_c0_g1_i1_1	9.599988	6.23724

DN18312_c0_g1_i1_2	5.975526	2.273246
DN18318_c0_g1_i1_2	7.575035	1.770081
DN18322_c0_g1_i1_1	3.588422	7.505054
DN18330_c0_g1_i6_2	8.858964	2.309999
DN18333_c0_g1_i1_2	7.797195	4.072256
DN18335_c0_g1_i1_1	1.164529	5.353577
DN18336_c0_g1_i1_1	0.6671253	11.68759
DN18337_c0_g1_i1_2	2.686561	3.752995
DN18338_c0_g1_i1_2	1.571813	0.8706645
DN18338_c0_g2_i1_1	0.4060129	0.4889529
DN18339_c0_g1_i2_2	3.020729	1.200957
DN1833_c0_g1_i1_1	5.25127	11.02323
DN18341_c0_g1_i1_2	15.26295	9.592659
DN18343_c0_g1_i2_1	3.142184	6.357036
DN18348_c0_g1_i3_2	6.73991	5.505456
DN1834_c0_g2_i1_1	2.009975	2.287714
DN18353_c0_g1_i2_1	2.223555	4.313323
DN18359_c0_g1_i1_1	0.8519528	7.109012
DN18360_c0_g1_i1_1	0.4528681	5.527612
DN18364_c0_g1_i1_1	0.1087133	4.665677
DN18373_c0_g1_i1_1	0.9873125	52.46817
DN18377_c0_g1_i1_1	2.272255	3.531065
DN18379_c0_g2_i1_1	0.6015567	1.058086
DN18383_c0_g2_i1_2	1.419081	1.390405

DN1838_c0_g1_i1_2	1.315739	0.5662189
DN18392_c0_g1_i1_2	3.963251	3.009808
DN18392_c0_g4_i1_1	0.6415731	1.228592
DN18393_c0_g1_i2_1	2.899113	33.94531
DN18400_c0_g1_i1_2	20.05318	11.29245
DN18401_c0_g1_i1_1	13.88668	6.647023
DN18402_c0_g1_i1_1	14.77313	16.68936
DN18404_c0_g1_i3_2	7.817346	10.54596
DN18420_c0_g1_i2_2	14.30165	5.641064
DN18423_c0_g1_i1_2	1.943351	0.2934087
DN18433_c0_g1_i1_2	0.8741129	0.04395285
DN18433_c0_g2_i1_2	1.792379	0.7171994
DN18445_c1_g3_i1_1	0.8745454	0.6851995
DN18446_c0_g1_i1_1	0.1795828	6.729523
DN18447_c0_g2_i1_1	0.6651974	3.746883
DN18449_c0_g1_i1_2	5.546516	1.557428
DN18450_c0_g1_i1_1	0.2920532	3.675076
DN18454_c0_g2_i1_2	0.207387	1.29E-110
DN18455_c0_g4_i1_2	3.207984	72.95525
DN18456_c0_g1_i3_2	4.064303	1.970546
DN18459_c0_g1_i1_1	7.963047	6.972484
DN18460_c0_g1_i1_2	63.05432	17.76922
DN18469_c0_g1_i3_1	101.142	45.00748
DN18482_c0_g1_i1_2	4.748147	2.849122

DN18486_c0_g3_i1_1	6.132664	1.388823
DN18493_c0_g2_i1_1	1.627227	1.842266
DN18497_c0_g1_i2_2	61.96768	35.16156
DN18498_c0_g1_i1_1	7.41439	5.98838
DN18504_c0_g1_i4_1	10.70272	9.901199
DN18507_c0_g1_i1_1	16.88208	93.72544
DN18512_c0_g1_i1_2	2.299004	1.730733
DN18512_c0_g2_i1_2	8.887612	4.932568
DN18512_c0_g3_i1_2	2.399324	2.959215
DN18515_c0_g1_i1_2	4.326618	2.266441
DN18516_c0_g2_i1_2	2.911995	1.806651
DN18516_c0_g3_i1_2	2.475766	1.866886
DN18520_c0_g1_i3_1	2.507473	2.268521
DN18520_c0_g2_i1_1	1.883405	0.6692423
DN18521_c0_g1_i1_1	1.060104	41.13698
DN1852_c0_g1_i1_1	0.347726	0.9225011
DN1852_c0_g1_i2_2	3.624016	3.430024
DN1853_c0_g1_i1_2	1.326877	1.252238
DN18542_c0_g1_i1_1	1.800927	4.730206
DN18545_c0_g1_i1_1	100.2758	104.8271
DN18553_c0_g1_i1_1	0.2749921	5.826395
DN18553_c0_g1_i1_2	0.7297723	0.2552637
DN18561_c0_g1_i2_2	2.748474	1.950324
DN18564_c0_g1_i14_1	0.7758764	20.91243

DN18564_c0_g4_i1_1	1.163389	5.345492
DN18564_c0_g5_i1_1	0.3359684	3.005056
DN1857_c0_g1_i2_1	2.583183	5.788998
DN18583_c0_g1_i1_2	4.962318	11.74983
DN18586_c0_g1_i1_2	4.221907	2.30251
DN18587_c0_g1_i1_1	3.874028	5.628995
DN18589_c0_g1_i1_1	1.411075	3.130084
DN18589_c0_g2_i2_1	1.72809	8.191912
DN18593_c0_g1_i4_2	20.69166	10.22603
DN18598_c0_g1_i1_1	0.2362115	7.609094
DN1859_c0_g1_i1_2	1.292982	0.7858548
DN18601_c0_g1_i1_1	0.7922766	1.593542
DN18605_c0_g1_i1_2	2.113533	1.711791
DN18613_c0_g1_i4_1	2.964988	36.9498
DN18614_c0_g1_i1_2	2.305509	0.7622814
DN18621_c1_g1_i3_1	2.048043	1.362584
DN1862_c0_g1_i1_2	1.096098	3.534276
DN18630_c0_g1_i1_2	2.264261	0.9602856
DN18631_c0_g1_i1_2	1.358645	1.088138
DN18635_c0_g1_i1_2	5.854643	4.225141
DN18641_c0_g1_i2_1	2.673133	2.861028
DN18644_c1_g4_i1_2	0.6591424	0.5708276
DN18645_c0_g1_i2_2	2.745433	0.8822544
DN18645_c0_g2_i1_2	2.860717	0.710289

DN18645_c1_g1_i1_2	2.167527	0.2481748
DN18651_c2_g1_i1_1	10.43958	7.083024
DN18651_c2_g4_i1_1	12.06331	2.999572
DN18652_c0_g1_i1_2	8.17408	4.078105
DN18663_c0_g1_i2_2	4.383592	3.715106
DN18675_c0_g1_i2_2	5.851198	3.683703
DN18679_c0_g1_i3_1	1.913192	1.097105
DN18682_c0_g1_i1_2	17.65365	48.71905
DN18687_c0_g1_i1_2	4.435098	1.614266
DN18687_c0_g3_i2_2	6.42621	5.074845
DN1868_c0_g1_i1_1	0.7273698	0.9846451
DN18693_c0_g1_i1_1	1.880299	2.376277
DN18697_c0_g1_i1_2	1.822558	0.9045208
DN18697_c0_g2_i5_1	5.467589	7.776719
DN186_c0_g1_i1_2	0.9435493	0.6545681
DN18701_c0_g1_i2_2	2.719606	2.938711
DN18703_c0_g1_i1_1	5.319017	5.118991
DN18706_c0_g1_i1_2	5.625708	2.613283
DN18707_c0_g1_i2_2	8.812699	12.73623
DN18710_c0_g1_i1_2	1.512645	1.999998
DN18715_c0_g1_i1_1	11.76441	13.36609
DN18720_c0_g1_i2_1	0.9979257	13.90999
DN18720_c0_g1_i2_2	4.291855	1.353938
DN18725_c0_g1_i1_1	3.087091	14.49027

DN18726_c0_g1_i1_2	27.31317	5.878566
DN18727_c0_g1_i1_2	1.243351	0.5192707
DN18727_c1_g2_i1_1	1.486513	3.699713
DN18727_c1_g3_i1_1	1.859241	3.925543
DN18727_c1_g4_i1_1	0.1125792	0.1976014
DN18727_c1_g5_i1_1	2.271854	3.123743
DN1872_c0_g1_i1_2	4.938266	1.587662
DN18738_c0_g1_i1_2	2.458568	6.235944
DN18748_c0_g1_i1_1	1.741713	4.525109
DN18748_c0_g1_i1_2	4.439625	4.159466
DN18749_c0_g1_i1_1	0.8056929	3.472781
DN18751_c0_g3_i1_1	2.542376	1.430806
DN18751_c0_g9_i1_1	1.453688	5.13097
DN18755_c0_g1_i1_1	5.095548	11.49962
DN18758_c0_g2_i1_2	3.014712	1.691443
DN18759_c0_g1_i1_2	5.962524	4.572158
DN18759_c0_g2_i1_2	6.084408	6.124357
DN18768_c0_g1_i1_2	4.25302	2.908206
DN1876_c0_g1_i1_2	2.233387	1.927362
DN18771_c0_g1_i1_1	0.5471852	1.626908
DN18772_c0_g1_i1_1	4.598867	11.19053
DN18774_c0_g1_i1_2	2.367563	1.605645
DN18784_c0_g1_i1_1	2.295417	7.948933
DN18789_c0_g3_i1_1	0.44887	2.041199

DN18790_c0_g1_i1_2	12.66119	6.135464
DN18791_c0_g1_i1_2	1.32416	0.7398039
DN18793_c0_g1_i1_1	46.07744	28.19046
DN18801_c0_g3_i1_2	1.720603	1.879799
DN18811_c0_g2_i1_2	6.746075	4.045764
DN18814_c0_g1_i1_1	0.7608636	2.756244
DN18817_c0_g1_i1_2	15.19906	14.46801
DN18819_c0_g4_i1_1	2.094014	0.9309254
DN1881_c0_g1_i1_1	10.73499	3.448234
DN18821_c0_g1_i1_2	0	0.1175664
DN1882_c0_g1_i2_2	3.655013	1.845284
DN18830_c0_g2_i1_2	9.352793	1.933945
DN18834_c0_g3_i1_1	1.40046	2.783634
DN18843_c0_g1_i1_2	4.005991	2.401319
DN18843_c1_g3_i1_1	1.976669	3.251678
DN18856_c0_g1_i1_1	3.416222	5.892519
DN18860_c0_g1_i1_2	10.42766	5.477506
DN18870_c0_g1_i1_2	10.52494	4.200083
DN18871_c0_g1_i1_1	1.320046	1.334824
DN18871_c0_g4_i2_1	1.434648	2.682101
DN1887_c0_g1_i1_2	1.681574	0.3668994
DN18882_c0_g1_i1_1	5.990417	4.63891
DN18885_c0_g3_i1_1	2.628529	12.31703
DN18889_c0_g1_i9_1	5.825123	3.40579

DN18898_c0_g1_i1_1	1.668999	14.75106
DN18902_c0_g1_i1_2	2.808819	2.519269
DN18905_c0_g1_i1_2	85.24952	0
DN18907_c0_g1_i1_2	4.097821	2.32247
DN18909_c0_g1_i2_1	5.639876	6.713033
DN18911_c0_g1_i1_2	4.231626	9.559981
DN18912_c0_g1_i1_2	5.852491	3.739632
DN18912_c0_g2_i1_2	8.066283	3.890157
DN18913_c0_g1_i1_2	7.834997	5.869457
DN18915_c1_g2_i1_1	1.988856	2.02695
DN18916_c0_g1_i1_1	1.300142	0.9469543
DN18919_c0_g1_i1_1	7.371037	8.062177
DN1892_c0_g1_i1_1	1.338647	0.7635077
DN18937_c0_g1_i1_2	2.550127	2.119067
DN18943_c0_g1_i1_1	0.08567163	1.645455
DN18943_c0_g1_i1_2	0.8695136	0.5108892
DN18947_c0_g1_i1_1	4.167324	9.738188
DN18948_c0_g1_i1_1	1.267454	20.62699
DN18961_c0_g2_i1_2	6.691082	7.95419
DN18964_c0_g1_i1_1	2.132498	9.991721
DN18968_c0_g3_i1_1	1.041148	1.625345
DN18968_c0_g4_i2_1	1.101558	1.159825
DN1896_c0_g1_i1_1	1.504018	2.619924
DN18978_c0_g1_i1_2	3.328014	2.489273

DN18983_c0_g1_i3_2	2.907311	1.516072
DN18990_c0_g2_i1_1	1.7845	2.646941
DN18990_c0_g3_i1_1	6.854378	5.750732
DN18990_c0_g4_i1_1	1.234668	0.9342883
DN18991_c0_g1_i2_1	1.829393	4.355076
DN18994_c0_g1_i1_1	0.7547171	18.89638
DN18996_c0_g2_i1_1	2.350458	1.59187
DN1900_c0_g1_i2_1	39.39508	6.380525
DN19016_c0_g1_i1_2	3.642997	2.039813
DN19024_c0_g1_i1_1	0.9110369	1.806402
DN19027_c0_g1_i2_2	8.350149	6.730187
DN1902_c0_g1_i1_2	5.784725	1.646929
DN19032_c0_g1_i2_2	1.92094	4.023748
DN19033_c0_g1_i5_1	0.190736	9.15941
DN19034_c0_g1_i1_1	2.223895	3.473563
DN19037_c0_g2_i1_1	4.221244	1.833728
DN19046_c0_g1_i2_2	4.624028	3.771915
DN1904_c0_g1_i1_2	5.87627	1.837424
DN19053_c0_g1_i1_2	4.032597	5.54724
DN19060_c0_g1_i1_1	2.562983	3.458265
DN19067_c0_g1_i1_1	2.07179	3.560007
DN19067_c1_g2_i1_1	0	1.375463
DN19067_c1_g3_i1_1	11.33995	10.84629
DN19067_c2_g1_i1_1	5.067507	3.623017

DN19069_c0_g1_i1_2	4.879961	2.619082
DN19071_c0_g1_i1_2	10.24385	7.182522
DN19072_c0_g1_i1_2	10.56372	3.814458
DN19078_c0_g1_i1_2	2.914177	1.993764
DN19079_c0_g2_i1_2	71.29851	122.8205
DN19084_c0_g1_i1_2	6.755296	13.52599
DN19086_c0_g2_i1_2	3.14815	3.051032
DN19096_c0_g1_i1_2	3.685447	3.67342
DN190_c0_g1_i2_2	2.453264	0.7564934
DN19111_c0_g4_i1_2	0.3971231	0.4313795
DN19113_c0_g1_i1_2	29.90305	22.33512
DN19114_c0_g1_i1_2	6.056563	4.315203
DN19117_c0_g4_i1_2	4.06525	18.54432
DN1911_c0_g1_i1_2	2.826382	1.462139
DN19125_c0_g1_i2_1	0.4676788	0.2819396
DN19127_c0_g1_i2_2	6.507692	2.782245
DN19127_c0_g2_i1_1	1.815317	18.7891
DN19128_c0_g1_i1_2	74.30892	0
DN1912_c0_g1_i1_1	0.6773861	5.26098
DN19136_c0_g3_i1_1	1.888307	1.778865
DN19136_c2_g13_i1_1	0.4977745	1.672047
DN19136_c2_g5_i1_1	0.8975461	1.620357
DN19136_c2_g6_i1_1	0	0
DN19136_c2_g8_i1_1	2.199242	1.752923

DN19138_c0_g1_i1_1	0.4564106	7.098734
DN1913_c0_g1_i1_1	0	3.940619
DN19141_c0_g1_i1_2	7.53742	8.431202
DN19144_c0_g1_i7_1	6.440444	18.44179
DN19147_c0_g1_i1_1	0.1889498	4.289692
DN1914_c0_g1_i1_1	0.4395267	0.49662
DN19158_c0_g1_i1_2	5.042638	1.358419
DN1916_c0_g1_i1_1	1.36279	1.777595
DN19170_c0_g3_i1_2	0	0
DN19181_c0_g1_i1_2	3.20047	0.80119
DN19181_c0_g2_i1_2	1.063247	0.6136958
DN19183_c0_g1_i1_2	7.329634	2.953189
DN19189_c0_g1_i1_1	1.961304	0.8603934
DN1918_c0_g1_i1_1	0.07653355	0.4200911
DN19206_c0_g2_i1_2	1.985044	1.490422
DN19220_c0_g1_i2_1	2.210037	2.911598
DN19221_c0_g1_i1_2	9.342561	4.0042
DN19221_c0_g3_i1_2	10.59134	3.010174
DN19241_c0_g1_i2_2	5.487108	6.020721
DN1924_c0_g1_i1_1	0.3092543	0.5740774
DN19251_c0_g1_i1_2	9.942677	4.190214
DN19258_c0_g1_i1_2	1.908989	2.332871
DN19261_c0_g4_i1_2	2.140269	2.370985
DN19261_c0_g6_i1_2	6.392013	4.575555

DN19261_c0_g8_i1_2	5.203674	1.885637
DN19266_c1_g1_i2_1	0.7004503	4.756244
DN19285_c0_g2_i1_1	38.53184	34.02461
DN19289_c0_g1_i1_1	0.06030946	3.354913
DN19289_c0_g3_i1_1	3.45E-47	3.385883
DN19294_c0_g1_i1_2	2.354306	2.945332
DN19297_c0_g1_i5_2	2.491376	0.6774129
DN192_c0_g1_i1_1	0.68166	1.237386
DN19300_c0_g3_i1_1	1.616384	1.716658
DN19300_c0_g4_i1_1	1.059776	1.626478
DN1930_c0_g1_i1_1	0.3373778	15.74788
DN1930_c0_g1_i1_2	3.055278	0.28026
DN1930_c0_g2_i1_2	2.56165	0.2602701
DN19316_c0_g2_i1_2	3.783455	8.499909
DN19319_c0_g1_i1_2	2.456576	1.145114
DN19319_c0_g2_i1_2	2.166901	0.8310901
DN19331_c0_g2_i1_1	0.9670269	3.232976
DN19339_c0_g1_i2_2	6.796938	3.416222
DN1933_c0_g1_i1_1	0	0.667849
DN1933_c0_g1_i1_2	0.8530722	0.1199513
DN19341_c0_g1_i1_2	1.446548	0.2600651
DN19341_c2_g1_i1_1	2.554165	4.837813
DN19351_c1_g5_i1_2	4.154889	0.6950261
DN19355_c0_g1_i1_2	7.277126	2.814056

DN19359_c0_g1_i2_1	2.327328	12.75058
DN1935_c0_g1_i1_2	3.034618	0.313609
DN19360_c0_g1_i2_1	5.479617	3.933413
DN19365_c0_g1_i1_1	0.3769726	12.21189
DN19369_c0_g3_i1_2	2.411631	0.4930818
DN19370_c0_g1_i3_2	1.952453	1.854798
DN19376_c0_g1_i1_1	7.306535	3.502534
DN19378_c0_g1_i1_2	5.629545	5.02023
DN19380_c1_g1_i1_2	1.333444	0.3003384
DN19390_c0_g1_i1_2	4.201609	6.248372
DN19392_c0_g2_i1_1	1.07455	2.226082
DN19395_c0_g2_i1_1	1.35905	2.374174
DN19396_c0_g1_i2_1	1.751742	2.009844
DN193_c0_g1_i1_2	1.04259	0.6013767
DN19402_c0_g1_i2_2	10.04139	3.836921
DN19415_c0_g1_i1_2	6.265962	24.388
DN19417_c1_g1_i2_2	5.234511	9.985279
DN19426_c0_g3_i1_1	0	0.7914689
DN19442_c0_g1_i1_1	5.183978	2.563902
DN19452_c2_g5_i1_2	0.7960286	3.896183
DN19459_c0_g1_i1_2	0.806799	0.6305598
DN1945_c0_g1_i1_1	71.76056	44.09265
DN19460_c0_g1_i1_1	0.8346887	2.005294
DN19460_c0_g3_i10_1	8.320734	5.912998

DN19468_c1_g1_i7_2	7.467305	3.359551
DN19468_c1_g2_i1_2	1.401655	0.4450422
DN19477_c0_g3_i4_1	2.321683	4.043035
DN19485_c0_g1_i1_2	19.53519	29.73449
DN19491_c0_g1_i1_1	0.8096479	39.57516
DN19495_c0_g2_i1_1	4.7323	3.86221
DN19497_c0_g5_i1_2	0.6525148	0.4045373
DN19497_c1_g1_i1_2	0.9570608	1.534626
DN1949_c0_g1_i1_1	1.041389	0.4279509
DN19504_c0_g1_i6_2	153.5625	99.82595
DN19505_c0_g2_i1_2	4.335819	1.125952
DN19510_c0_g2_i1_1	0.554815	1.494486
DN19521_c0_g2_i5_2	0.8356217	0.8194141
DN19523_c0_g2_i1_1	1.208968	2.390193
DN19523_c0_g3_i1_1	0.1990878	0.7003619
DN19523_c0_g4_i1_1	5.103983	6.498125
DN19524_c4_g1_i2_2	1.498915	1.799168
DN19524_c6_g1_i1_2	0.6999244	3.232253
DN19529_c0_g1_i2_2	3.605114	0.05675356
DN19529_c1_g4_i1_1	4.906982	2.250155
DN19529_c1_g5_i1_1	2.240911	0.7871739
DN19529_c1_g6_i1_1	1.871933	1.377204
DN19530_c0_g1_i1_2	5.446745	1.361507
DN19530_c0_g2_i1_2	3.216665	0.8487942

DN19534_c0_g2_i1_2	25.67898	140.776
DN19536_c0_g1_i1_2	9.645682	9.254774
DN19537_c0_g1_i2_2	28.82361	0.2037718
DN19539_c0_g2_i1_1	0.4861843	0.3747312
DN19541_c0_g1_i1_2	10.24389	9.271737
DN19542_c3_g1_i1_2	35.51175	1.913105
DN19553_c0_g2_i1_2	1.053835	0.7566166
DN19555_c0_g1_i2_1	6.427626	9.05773
DN19564_c0_g1_i1_1	0.372457	3.163277
DN19566_c0_g1_i3_2	16.69647	47.10458
DN19566_c1_g1_i1_2	7.554502	1.911479
DN19568_c0_g1_i1_1	2.035146	2.388232
DN19569_c0_g1_i1_2	3.383698	0.5446444
DN19580_c0_g7_i1_1	2.006734	1.671105
DN19580_c0_g8_i1_1	0.9482283	4.922498
DN19580_c0_g9_i1_1	5.017218	4.315474
DN19582_c0_g2_i1_2	11.18166	13.58211
DN19585_c0_g4_i1_1	8.278062	2.85392
DN19591_c0_g1_i1_1	6.04464	3.714001
DN1959_c0_g1_i1_2	2.786864	0.6336092
DN195_c0_g1_i1_2	2.599226	17.50571
DN195_c0_g1_i2_1	0	0.7489706
DN19601_c1_g1_i1_1	2.818379	1.580289
DN19601_c1_g2_i3_1	3.721381	2.543576

DN19601_c1_g4_i1_1	18.58405	7.057836
DN19602_c0_g1_i2_2	2.888189	1.393201
DN19602_c0_g2_i1_2	2.791896	1.993655
DN19607_c2_g2_i1_1	0	0.5630578
DN19607_c2_g4_i1_1	0.3279137	0.3999021
DN19608_c0_g1_i11_1	3.566987	2.319417
DN19613_c0_g3_i1_1	1.022535	1.799595
DN19613_c1_g4_i1_1	1.128447	0.6875161
DN19621_c2_g3_i1_1	0.7082838	0.637783
DN19622_c2_g3_i1_1	1.420782	1.970006
DN19625_c0_g2_i1_2	2.714293	4.611267
DN19635_c0_g3_i1_2	15.41549	5.129909
DN19638_c0_g1_i1_2	2.833814	5.010192
DN19639_c0_g1_i1_1	0.3005975	0.1641158
DN19642_c0_g1_i1_2	7.090155	7.316423
DN19658_c1_g2_i2_1	1.478087	1.23E-06
DN19658_c1_g4_i1_1	3.820612	2.532984
DN19669_c0_g3_i2_1	0.9617123	1.541081
DN1966_c0_g1_i1_2	21.3944	39.9367
DN19675_c0_g1_i1_2	9.770227	4.213938
DN1967_c0_g1_i1_1	28.19942	25.55675
DN19681_c0_g1_i1_2	2.763624	1.299195
DN19681_c0_g1_i2_1	20.28135	21.23325
DN19685_c0_g1_i1_1	1.420198	2.603654

DN19685_c1_g2_i1_1	1.314677	0.745449
DN19685_c1_g5_i1_1	1.311125	1.934383
DN19686_c0_g1_i1_1	0.9198685	44.06758
DN19694_c0_g1_i1_2	16.21018	4.105269
DN19704_c1_g4_i1_2	14.31503	7.927184
DN19706_c0_g1_i1_2	4.602862	1.618602
DN19708_c0_g1_i1_2	4.815917	2.383997
DN19713_c0_g3_i1_2	1.026817	1.662203
DN1972_c0_g1_i1_2	3.252863	2.135204
DN19731_c1_g7_i1_1	0.2561155	12.17115
DN19736_c1_g1_i1_1	0.9900633	1.981075
DN19748_c0_g1_i1_1	3.736448	5.520282
DN19751_c0_g1_i1_1	1.977412	2.749844
DN19757_c0_g4_i1_2	1.639056	1.641067
DN19761_c0_g1_i1_1	0.9363194	1.251518
DN19761_c1_g8_i1_1	1.206405	1.96335
DN19768_c0_g1_i2_2	7.065095	6.651595
DN19771_c0_g1_i2_1	3.541367	8.076762
DN19773_c0_g1_i1_1	1.057389	1.384515
DN19778_c0_g1_i1_2	2.01931	0.4458753
DN19778_c1_g2_i1_1	10.23231	0.323228
DN19794_c0_g3_i1_2	1.24459	0.4434132
DN19797_c0_g1_i3_2	3.293873	0.9134998
DN19798_c1_g2_i2_1	2.489656	1.847324

DN19800_c1_g11_i1_1	1.024207	2.077668
DN19800_c1_g14_i1_1	0.7908177	1.415081
DN19800_c1_g4_i1_1	0.4745357	1.054331
DN19800_c1_g9_i1_1	1.053214	1.193166
DN19807_c2_g4_i1_1	3.260082	1.927184
DN19811_c0_g1_i5_2	5.82166	4.292314
DN19811_c0_g2_i1_2	1.580246	0.8835518
DN19818_c0_g1_i1_1	4.126015	2.098875
DN19818_c1_g3_i1_1	1.971664	3.717652
DN1981_c0_g1_i1_1	1.12078	1.585485
DN19821_c0_g1_i4_2	15.55406	4.59251
DN19828_c0_g1_i1_2	39.52234	34.58531
DN19832_c1_g1_i1_1	3.607697	1.961785
DN19832_c2_g1_i19_1	12.41299	7.097658
DN19832_c2_g4_i1_1	1.391888	1.770523
DN19833_c3_g1_i2_1	1.280368	4.502365
DN19833_c5_g4_i1_1	1.701678	0.4772096
DN19833_c5_g7_i1_1	1.479076	3.3545
DN19835_c0_g1_i1_1	0.5638622	1.189984
DN19835_c0_g2_i1_1	2.439371	1.555838
DN19839_c3_g1_i3_1	3.371424	4.242611
DN19841_c2_g1_i2_1	12.81087	12.67288
DN19842_c4_g1_i1_1	0	1.18606
DN19845_c1_g2_i1_1	2.15538	0.962187

DN19848_c0_g1_i1_1	1.225866	1.470887
DN19849_c0_g1_i1_1	0.2167285	0.5091691
DN19850_c0_g1_i1_1	0.3707061	11.97115
DN19878_c0_g1_i1_1	2.449571	1.727476
DN19885_c0_g1_i1_1	4.041407	4.501129
DN19886_c0_g1_i1_1	1.981687	2.525602
DN19890_c0_g1_i1_1	1.789713	1.373955
DN1989_c0_g1_i1_2	1.920326	0.84546
DN19901_c0_g1_i1_1	0.4590346	0.6930004
DN19902_c0_g1_i1_1	4.423436	15.20841
DN19905_c0_g1_i1_1	9.119116	16.87505
DN19909_c0_g1_i1_1	3.936527	6.615032
DN1990_c0_g1_i1_1	0.3636741	2.608636
DN19914_c0_g1_i1_1	0.9863514	1.546231
DN19915_c0_g1_i1_1	5.583758	10.84182
DN19919_c0_g1_i1_1	0.1245064	5.252285
DN1991_c0_g1_i1_1	0.159616	0.9392974
DN19923_c0_g1_i2_2	1.275598	0.3939235
DN19925_c0_g1_i1_1	0.4293369	0.7298757
DN19926_c0_g2_i1_2	2.51402	1.225691
DN19927_c0_g1_i1_1	6.284804	20.31583
DN19928_c0_g1_i1_2	3.589018	2.067285
DN19929_c0_g1_i1_1	0.3726235	1.917023
DN19933_c0_g1_i1_1	7.377815	3.130495

DN19934_c0_g1_i1_1	6.888696	8.093102
DN1993_c0_g1_i2_1	5.070562	9.415624
DN19942_c0_g1_i1_1	1.574959	2.959242
DN19947_c0_g1_i1_2	12.45301	8.392497
DN19952_c0_g1_i1_1	1.677035	1.412843
DN19953_c0_g1_i1_1	0.2370922	0.3243805
DN19954_c0_g1_i1_1	0.6751091	18.60052
DN19954_c0_g1_i1_2	8.463269	4.421378
DN19958_c0_g1_i1_1	0.5109406	2.913961
DN19959_c0_g1_i1_1	0.4355374	1.217585
DN19970_c0_g1_i1_2	409.3001	146.9471
DN19971_c0_g1_i1_1	1.230294	2.650959
DN19977_c0_g1_i1_2	0	0
DN19978_c0_g1_i1_1	3.632141	3.739635
DN19979_c1_g8_i1_2	2.13E-73	1.01E-83
DN19981_c0_g1_i1_1	2.568122	2.026454
DN19982_c0_g1_i1_1	2.786162	2.553256
DN19984_c0_g1_i1_1	0.514357	1.480194
DN19988_c0_g1_i1_1	1.749893	0.7973885
DN19988_c0_g1_i2_2	3.121735	5.855712
DN19989_c0_g1_i1_1	0.2823999	0.5118515
DN1998_c0_g1_i1_1	0.2469297	4.691445
DN19993_c0_g1_i1_1	1.826841	4.099262
DN19996_c0_g1_i1_1	2.53243	1.804456

DN1999_c0_g1_i1_1	0.3922141	0
DN20003_c0_g1_i1_1	1.007128	1.148039
DN20008_c0_g1_i1_1	1.110136	1.51247
DN20010_c0_g1_i1_1	0.5853182	0.4310877
DN20012_c0_g1_i1_2	2.623716	1.401842
DN20013_c0_g1_i1_1	2.521946	4.084112
DN20015_c0_g1_i1_1	1.332758	7.755428
DN20025_c0_g2_i1_2	4.776147	2.821157
DN20027_c0_g1_i1_1	1.175317	1.865104
DN20027_c2_g1_i2_2	4.062272	2.371846
DN20031_c0_g1_i1_1	36.77561	21.87243
DN20031_c0_g1_i2_2	9.805652	3.748845
DN20034_c0_g1_i1_1	1.163055	0.3109218
DN20046_c0_g1_i1_2	2.292471	0.9635536
DN20050_c0_g1_i3_2	3.371085	1.744625
DN20051_c0_g1_i1_1	1.856396	1.496117
DN20052_c0_g1_i1_1	0.6378003	1.558124
DN20055_c0_g1_i1_1	17.1699	26.43224
DN20059_c0_g1_i1_1	3.034389	1.662706
DN20065_c0_g1_i1_1	1.329507	0.5212637
DN20069_c0_g2_i1_2	1.836112	0.6895731
DN20073_c0_g1_i1_1	0.2524113	1.716815
DN20074_c1_g1_i5_2	8.836219	1.02226
DN20074_c1_g3_i1_2	5.957481	2.978754

DN20074_c1_g4_i3_2	11.7164	5.581262
DN20074_c1_g5_i1_2	3.829145	0.8488004
DN20074_c1_g6_i1_2	2.919538	0.7441945
DN2007_c0_g1_i1_1	1.400054	1.894774
DN2007_c0_g1_i1_2	1.649967	0.4371075
DN20095_c0_g1_i1_1	1.947425	2.257597
DN20101_c0_g1_i8_2	0	0
DN20102_c0_g1_i1_2	4.799526	8.978225
DN20104_c0_g1_i1_2	34.47547	13.72113
DN20106_c0_g1_i1_2	4.669406	2.477133
DN20107_c0_g1_i1_1	1.120173	3.5318
DN20114_c0_g1_i1_1	0.9985479	1.011711
DN2011_c0_g1_i1_1	2.818795	1.689701
DN20120_c0_g1_i1_1	11.48351	4.281348
DN20137_c2_g2_i2_2	3.270485	1.152435
DN20137_c2_g3_i2_2	5.097451	3.599143
DN20137_c2_g5_i1_2	2.448565	0.1862918
DN20137_c2_g6_i1_2	5.570288	1.433844
DN20137_c2_g9_i1_2	1.252154	0.5804494
DN20138_c0_g1_i1_1	2.993537	3.837687
DN2013_c0_g2_i1_1	1.90993	1.117422
DN20148_c0_g1_i1_2	8.955793	13.46567
DN20149_c0_g1_i1_1	2.637956	1.260999
DN2014_c0_g1_i1_2	0.7321249	0.5453927

DN2014_c0_g2_i1_1	2.005617	0.8374045
DN2014_c0_g2_i1_2	0.5118659	0.4830132
DN20150_c0_g1_i1_1	5.142244	5.455787
DN20150_c2_g1_i2_2	12.09518	0.5201701
DN20153_c0_g1_i2_2	2.216049	2.123624
DN20157_c0_g1_i1_1	0.7915995	0.9257809
DN20165_c0_g1_i1_1	33.97111	16.26844
DN20168_c0_g1_i1_1	5.896514	3.222926
DN2016_c0_g1_i1_1	2.47594	1.6799
DN20170_c0_g1_i1_1	1.797606	5.021511
DN20179_c0_g1_i1_1	2.214639	8.308009
DN2017_c0_g1_i1_1	0	0
DN2017_c0_g1_i1_2	1.331734	1.872682
DN20182_c0_g1_i1_1	2.016632	2.59949
DN20184_c0_g2_i6_2	23.78343	15.89603
DN20184_c0_g4_i1_2	1.298941	0.2057133
DN20185_c0_g1_i1_2	5.178453	2.575678
DN20186_c0_g1_i1_1	0.9028991	0.9732757
DN20191_c0_g1_i1_1	5.56378	2.60052
DN20199_c0_g1_i1_1	13.18773	4.62337
DN20199_c0_g1_i5_2	37.49341	0.4112067
DN20202_c0_g13_i1_2	157.0368	4.688797
DN20202_c0_g1_i1_1	1.790893	3.756997
DN20202_c0_g7_i4_2	2.89777	0.1315095

DN20208_c0_g1_i1_1	2.860873	6.232236
DN20210_c0_g1_i1_1	12.11746	10.35304
DN20210_c0_g2_i1_2	2.649845	0.8573091
DN20211_c0_g1_i1_1	0	1.790067
DN20216_c0_g1_i1_1	2.652553	3.580548
DN20222_c0_g1_i1_1	4.413287	4.137386
DN20228_c0_g1_i1_1	2.436877	11.55308
DN20229_c0_g1_i1_1	34.20519	18.82286
DN20234_c0_g1_i1_1	0.505102	3.356403
DN20238_c0_g1_i3_2	33.7057	11.74817
DN2023_c0_g1_i1_2	0.6953593	0.2403784
DN20245_c0_g1_i1_1	5.534547	5.26747
DN20249_c0_g1_i1_1	2.13423	7.391369
DN2024_c0_g1_i1_1	4.605345	1.831076
DN2024_c0_g1_i1_2	3.471952	1.234286
DN20253_c0_g1_i1_1	1.902878	4.38472
DN20254_c0_g1_i1_2	1.968382	0.6497673
DN20256_c0_g1_i1_1	5.189161	2.835847
DN20260_c0_g1_i1_2	24.28675	10.66535
DN20264_c0_g1_i1_1	1.040673	1.944634
DN20269_c0_g1_i1_1	12.35979	30.79729
DN20273_c0_g1_i1_1	0	0
DN20277_c0_g1_i1_1	1.051585	0.8987685
DN20285_c0_g1_i2_2	6.761306	15.43997

DN20286_c0_g1_i1_1	14.67532	32.20629
DN20290_c0_g1_i1_1	1.731078	1.488521
DN20293_c0_g1_i1_1	0.8069701	0.8141038
DN20295_c0_g1_i1_1	8.039579	30.88192
DN20305_c0_g1_i1_1	5.250374	4.273203
DN20308_c0_g1_i1_1	6.900034	3.141283
DN20309_c0_g1_i1_1	0.320529	0.730824
DN2030_c0_g2_i1_2	3.965746	0.5880298
DN20313_c0_g1_i1_1	19.70103	71.1207
DN20315_c0_g1_i1_1	51.76665	59.83799
DN20316_c0_g1_i1_1	2.099109	2.529196
DN20320_c0_g1_i1_1	0	0
DN20322_c0_g1_i1_1	4.995302	19.92271
DN20330_c0_g1_i1_1	1.036626	1.704781
DN20331_c0_g1_i1_1	2.162244	6.80554
DN20332_c0_g1_i1_1	2.388076	3.934127
DN20333_c0_g1_i1_1	0.659894	0.6224051
DN20333_c0_g1_i1_2	10.51158	6.289304
DN20337_c0_g1_i1_1	1.29454	1.454845
DN20339_c0_g1_i1_1	0.1915087	0.9782059
DN20344_c0_g1_i1_1	0.7605426	11.82715
DN20350_c0_g1_i1_1	0.2709966	2.151827
DN20351_c0_g1_i1_1	2.767228	1.882827
DN20352_c0_g1_i1_1	0.3282681	0.3427739

DN20357_c0_g1_i1_2	8.678491	5.894829
DN20360_c0_g1_i1_1	1.586113	2.941668
DN20362_c0_g4_i2_2	4.058369	0.7453504
DN20368_c0_g1_i1_1	4.55655	4.15864
DN20368_c0_g1_i2_2	10.13781	1.723608
DN20370_c0_g1_i1_1	1.410652	1.871318
DN20371_c1_g1_i7_2	7.933286	3.059677
DN20371_c1_g3_i1_2	6.621967	2.837962
DN20374_c0_g1_i1_1	3.666302	3.02598
DN20378_c0_g1_i1_1	3.494	3.554755
DN20379_c0_g1_i1_2	3.364134	1.73498
DN20382_c0_g1_i1_1	1.56737	1.664015
DN20384_c0_g1_i1_1	0.1263462	0.6848545
DN20387_c0_g1_i1_2	4.840934	3.53585
DN2038_c0_g1_i1_2	11.73687	9.729391
DN20390_c0_g1_i1_1	0.7687104	0.8864209
DN20393_c0_g1_i1_1	1.492893	1.513189
DN20398_c0_g1_i1_1	45.30748	40.91075
DN20399_c0_g1_i1_2	4.874322	2.64158
DN20401_c0_g1_i1_2	7.453472	3.190286
DN20403_c0_g1_i1_1	0.3362348	14.71878
DN20403_c1_g3_i1_2	0	0
DN20407_c0_g1_i1_1	0	0
DN20408_c0_g1_i1_1	5.717976	4.029447

DN2040_c0_g1_i1_1	2.707116	2.166424
DN20410_c0_g1_i1_1	1.35994	1.1078
DN20414_c0_g1_i1_2	15.30791	6.414977
DN20420_c0_g1_i1_1	17.57528	16.06554
DN20425_c0_g1_i1_1	0.277944	3.361919
DN2042_c0_g1_i1_2	2.676811	1.024743
DN20430_c0_g1_i1_1	0.563909	0.5402339
DN20438_c0_g1_i1_1	1.767061	5.725078
DN20439_c0_g1_i1_1	3.4179	5.488405
DN20451_c0_g1_i1_1	0.192245	1.703584
DN2045_c0_g2_i1_2	17.07443	11.53762
DN20464_c0_g1_i1_2	3.951305	3.73869
DN20466_c0_g1_i2_2	4.621666	2.544907
DN20471_c0_g1_i1_1	0.2750697	1.360531
DN20475_c0_g1_i1_1	5.409374	4.446264
DN20484_c0_g1_i1_1	1.41379	2.06315
DN20487_c0_g1_i1_1	0.2215463	0.4780395
DN20488_c0_g1_i1_1	2.778884	2.363372
DN20498_c0_g1_i1_1	3.344896	4.171038
DN20500_c0_g1_i4_2	9.940463	4.758285
DN20500_c0_g2_i1_2	4.55434	2.4096
DN20500_c0_g4_i1_2	3.551813	2.975123
DN20500_c0_g6_i2_2	2.190095	3.480711
DN20502_c0_g1_i1_1	3.682317	4.898385

DN20503_c0_g1_i1_1	1.127844	4.784264
DN20504_c0_g1_i1_1	10.97112	43.00256
DN20505_c0_g1_i1_2	7.983036	15.8303
DN20509_c0_g1_i1_1	305.5101	253.3402
DN20509_c0_g2_i1_2	8.408823	0
DN20511_c0_g1_i1_1	3.312195	1.039526
DN20515_c0_g1_i1_1	0.6003463	1.734975
DN20522_c0_g1_i1_1	227.6112	11.1922
DN20523_c0_g1_i1_1	2.376874	5.328267
DN20527_c0_g1_i1_1	7.553227	15.13647
DN20528_c0_g1_i1_1	1.880391	3.243858
DN20532_c0_g1_i1_1	1.30401	1.011851
DN20539_c0_g1_i1_1	0.2006555	2.040322
DN20542_c0_g1_i1_1	1.044619	1.340215
DN20550_c0_g1_i1_1	0.5571064	1.865644
DN20552_c0_g1_i1_1	1.59793	1.243469
DN20557_c0_g1_i1_1	1.95E-27	5.00E-23
DN20558_c0_g2_i1_2	47.11345	68.93556
DN20563_c0_g1_i1_2	3.816664	1.646531
DN20567_c0_g1_i1_1	0.890474	1.483562
DN2056_c0_g1_i1_2	2.680567	2.599911
DN20576_c0_g4_i1_2	2.802649	0.8251768
DN20577_c0_g1_i1_1	5.15857	9.305823
DN20582_c0_g1_i1_1	0.9788287	1.087421

DN20590_c0_g1_i1_1	5.890259	13.76347
DN20591_c0_g1_i1_1	2.20211	5.956359
DN20594_c0_g1_i1_1	1.048759	0.6104339
DN20599_c0_g1_i1_1	2.69E-17	0.6785895
DN2059_c0_g2_i1_1	1.120681	0.9962647
DN20603_c0_g1_i1_1	0.1924815	4.791828
DN20605_c0_g1_i1_1	1.394289	1.36696
DN20606_c0_g1_i1_1	2.042166	1.650508
DN20607_c0_g1_i1_1	1.44393	17.10672
DN20609_c0_g1_i1_1	0.4684723	0.301753
DN2060_c0_g1_i1_2	10.59687	3.729095
DN20612_c0_g1_i1_1	2.645009	1.142376
DN20613_c0_g1_i1_2	4.038686	0.6303458
DN20615_c0_g1_i1_1	1.494199	2.827988
DN20617_c0_g1_i1_1	1.320885	1.528273
DN2061_c0_g1_i1_1	0.9092958	1.445092
DN20623_c0_g1_i1_1	0.465591	1.405163
DN20629_c0_g1_i1_1	0	2.19314
DN2062_c0_g1_i1_1	2.108196	2.037177
DN20631_c0_g2_i1_2	4.475678	4.793917
DN20632_c0_g1_i1_1	0	0.4773936
DN20635_c0_g1_i1_1	2.98392	4.132677
DN20643_c0_g1_i1_1	1.231815	2.519144
DN20644_c1_g1_i1_2	4.629183	9.506489

DN20644_c1_g6_i1_2	6.779169	9.942471
DN20645_c0_g1_i1_1	0	0.7852288
DN20647_c0_g1_i1_1	119.7769	174.9039
DN20649_c0_g1_i1_1	0.673154	1.033574
DN20651_c0_g1_i1_1	2.159449	3.226038
DN20654_c0_g1_i1_1	0.8973076	4.609209
DN20655_c0_g1_i1_1	2.413058	1.202836
DN20656_c0_g1_i1_1	0.7932738	1.055188
DN20658_c0_g1_i1_1	0.9354414	0.989909
DN20660_c0_g1_i1_1	0	3.974501
DN20665_c0_g1_i1_1	0	0.7337612
DN20670_c0_g1_i1_1	1.650809	1.631898
DN20672_c0_g1_i1_2	5.359556	1.523622
DN20677_c0_g1_i24_2	7.52847	3.594229
DN2067_c0_g1_i1_1	1.904336	3.401558
DN20683_c0_g1_i1_1	0.5681933	1.326744
DN20683_c0_g1_i1_2	8.174535	5.979937
DN20690_c0_g1_i1_2	10.99298	2.254685
DN20690_c0_g2_i1_2	5.734037	0.6402178
DN20691_c0_g1_i1_1	27.37298	29.31837
DN20695_c0_g1_i1_1	0.7293841	0.5786576
DN20697_c0_g1_i1_1	0.1618415	0
DN20699_c0_g1_i1_1	0.2644989	1.022974
DN20708_c0_g1_i1_1	0.3565094	0.09942245

DN20708_c1_g1_i2_2	5.223177	7.009535
DN2070_c0_g1_i1_1	0.6298541	0.4529172
DN20710_c0_g1_i1_1	32.34563	18.66216
DN20717_c0_g1_i1_1	1.144357	0.3981419
DN20717_c0_g1_i3_2	2.51636	1.732931
DN2071_c0_g1_i1_1	11.00305	1.798124
DN20727_c0_g1_i1_1	0.7111798	1.762702
DN20728_c0_g1_i1_1	148.85	143.8784
DN2072_c0_g1_i1_1	1.466314	2.195288
DN20730_c0_g1_i1_1	1.11423	4.505467
DN20732_c0_g1_i1_1	1.379439	3.096601
DN20738_c0_g1_i1_1	0.5071607	0.3585403
DN20739_c0_g1_i1_1	3.763068	1.42198
DN20742_c0_g1_i1_1	1.232431	0.6074648
DN20743_c0_g1_i1_1	0	1.425154
DN20744_c0_g1_i5_2	4.328919	4.429791
DN20750_c0_g1_i1_1	1.53051	0.8895105
DN20751_c0_g1_i1_1	0.457485	1.568212
DN20755_c0_g1_i1_1	20.48525	49.26227
DN20757_c0_g1_i1_1	0.8851391	1.004416
DN20764_c0_g1_i1_1	1.050813	2.510792
DN20765_c0_g1_i1_1	5.719408	11.44672
DN20768_c0_g1_i1_1	0.3959649	1.679187
DN20769_c0_g1_i1_1	0.9481367	0.6694362

DN20769_c0_g1_i1_2	11.5833	2.658122
DN20771_c0_g4_i2_2	2.193952	0
DN20771_c0_g6_i1_2	0.712948	2.149612
DN20779_c0_g1_i1_1	1.142766	0.6962723
DN2077_c0_g1_i1_1	1.296765	1.621209
DN20780_c0_g1_i1_1	0.4551436	1.191719
DN20782_c0_g1_i1_1	0.7468423	1.025482
DN20783_c0_g2_i1_2	2.317835	1.019959
DN20789_c0_g1_i1_1	14.39012	7.412563
DN2078_c0_g1_i1_1	0.1119098	0.3987393
DN20790_c0_g4_i1_2	2.193916	1.636789
DN20794_c0_g1_i1_1	27.95871	28.26051
DN20795_c0_g1_i1_1	0.2419977	1.435308
DN20798_c0_g1_i1_1	0	4.557022
DN20799_c0_g1_i1_1	3.68225	2.007796
DN20800_c0_g1_i1_1	1.833424	0.8297918
DN20800_c0_g1_i1_2	2.063753	2.530544
DN20807_c0_g1_i1_1	2.33643	3.889546
DN20809_c0_g1_i1_1	0.6923378	1.536139
DN2080_c0_g2_i1_1	0	0
DN20811_c0_g1_i1_1	0.6021604	1.22821
DN20815_c0_g1_i1_1	1.249983	0.9987657
DN20816_c1_g1_i1_2	4.121132	0.1590675
DN20816_c1_g3_i1_2	1.087343	0.09333867

DN20816_c1_g4_i4_2	5.900304	1.471796
DN20818_c0_g1_i3_2	7.926564	5.070776
DN20820_c0_g1_i1_1	1.494032	2.143531
DN20821_c2_g1_i2_2	3.506722	0.6591937
DN20821_c2_g2_i2_2	1.746946	0.7205565
DN20822_c0_g1_i1_1	4.73336	0.6818013
DN20825_c0_g1_i1_1	3.02727	1.166533
DN20825_c0_g2_i2_2	3.201281	1.137386
DN20825_c0_g4_i1_2	0.5589226	0.05989821
DN20828_c0_g1_i1_1	4.997977	3.137732
DN2082_c0_g1_i1_2	7.047048	1.267837
DN20830_c0_g1_i1_1	2.166993	1.167831
DN20833_c0_g1_i1_1	17.8927	5.00668
DN20837_c0_g1_i1_1	0.9389552	0.6649592
DN2083_c0_g1_i1_2	0.8957298	0.6259508
DN20843_c0_g1_i1_1	15.40024	34.36648
DN20850_c0_g1_i1_1	1.685652	3.136225
DN20869_c0_g1_i1_2	4.55578	1.147248
DN2086_c0_g1_i1_1	2.168372	2.809844
DN20871_c0_g1_i1_1	33.97369	26.96543
DN20874_c0_g1_i1_1	0.3439953	0.5037748
DN20877_c0_g1_i1_1	0.7693368	2.060913
DN20881_c0_g1_i1_1	1.139384	1.743075
DN20883_c0_g1_i1_1	0.9317363	1.633771

DN20889_c0_g1_i1_1	0.5951452	0.6134608
DN2088_c0_g1_i2_1	24.2494	22.39837
DN20895_c0_g1_i1_2	3.678455	5.763446
DN20897_c0_g1_i1_1	2.690957	4.778295
DN20898_c0_g1_i1_1	1.095954	1.945738
DN20900_c0_g1_i1_1	1.768204	2.126497
DN20915_c0_g1_i1_1	1.09574	10.15434
DN20916_c0_g2_i2_2	15.68063	5.567366
DN20916_c0_g3_i1_2	10.59386	1.926154
DN20917_c0_g1_i1_1	4.520018	14.0945
DN20918_c0_g1_i1_1	1.017654	3.719816
DN20921_c0_g1_i1_1	3.079724	2.214127
DN20922_c0_g1_i1_1	37.06166	54.51376
DN20923_c0_g1_i1_1	3.635666	5.16993
DN20924_c0_g1_i2_2	170.7504	1.058259
DN20927_c0_g1_i1_1	1.892321	18.55031
DN20930_c0_g1_i1_1	0	0.3751082
DN20934_c0_g1_i1_1	1.42241	3.295687
DN20937_c0_g1_i1_1	1.172634	2.464973
DN20938_c0_g1_i1_1	5.980119	42.27805
DN20938_c0_g1_i6_2	11.95733	11.34137
DN20944_c0_g1_i1_1	2.795827	5.785231
DN20946_c0_g1_i1_1	20.75185	2.868607
DN20951_c0_g1_i1_1	6.910817	6.617735

DN20954_c0_g1_i1_1	0.7554021	1.941676
DN20959_c0_g1_i1_1	0.3422102	0.7596439
DN2095_c0_g1_i1_1	4.008224	4.684452
DN2095_c0_g1_i1_2	3.346655	3.34338
DN20960_c1_g1_i1_2	1.145059	0.5853496
DN20965_c0_g1_i1_1	0	1.068585
DN20966_c0_g1_i1_2	23.85585	4.086001
DN20968_c0_g1_i1_1	3.580595	4.934262
DN20969_c0_g1_i1_1	3.480487	1.984377
DN20969_c0_g1_i2_2	8.43E-16	1.33567
DN2096_c0_g1_i1_1	0.2072706	0.5588693
DN20976_c0_g1_i1_1	41.83638	24.91723
DN20982_c0_g1_i1_2	5.11217	3.733263
DN20983_c0_g1_i1_1	4.660117	11.97468
DN20985_c0_g1_i1_1	23.35764	30.90949
DN20987_c0_g1_i1_1	0.9890929	3.623559
DN20990_c0_g1_i1_1	0.4782635	1.574456
DN20994_c0_g1_i1_1	1.804563	1.430891
DN20998_c0_g1_i1_1	0.438227	0.7576679
DN21008_c0_g1_i1_1	0.4926901	0.853897
DN2100_c0_g1_i1_1	1.310011	1.040732
DN2100_c0_g1_i1_2	10.67151	8.079289
DN21012_c0_g1_i1_1	0.6990013	1.740411
DN21017_c2_g3_i1_2	0.05646757	0.1908096

DN21017_c3_g1_i1_2	0.08978424	0.05208503
DN21019_c0_g1_i1_1	5.144809	4.14803
DN2101_c0_g1_i1_1	0.9957169	0.3773435
DN21023_c0_g1_i1_1	2.412246	2.885065
DN21025_c0_g1_i1_1	1.488333	2.265016
DN21030_c0_g1_i1_1	1.299431	0.8564548
DN21033_c0_g1_i4_2	4.487215	1.727488
DN21038_c0_g1_i1_1	3.270889	7.708334
DN21040_c0_g1_i1_1	0.6115884	1.136095
DN21041_c0_g1_i1_1	0	0
DN21052_c0_g1_i1_1	1.015656	1.304607
DN21054_c0_g1_i1_1	0.6817324	0.4092887
DN21058_c0_g1_i1_1	4.670288	3.978146
DN21060_c0_g1_i1_1	0.5845566	1.152384
DN21062_c0_g1_i1_1	1.480648	2.326185
DN21067_c0_g1_i1_1	0.5638407	0.3049914
DN21069_c0_g1_i1_1	2.563517	2.360914
DN21070_c0_g1_i1_1	4.036803	18.13597
DN21071_c0_g1_i1_1	9.49821	3.094564
DN21074_c0_g1_i1_1	1.395721	1.894576
DN21075_c0_g1_i1_1	2.163946	0.4106801
DN21077_c0_g1_i1_1	0.8053436	2.74843
DN21087_c0_g1_i1_1	0.6762485	3.374071
DN21088_c0_g1_i1_2	3.281895	11.27552

DN21089_c0_g1_i1_1	0.1382131	2.054125
DN21091_c0_g1_i1_1	0.3296796	2.421928
DN21092_c0_g1_i1_1	0.8337163	0.8324446
DN21097_c0_g1_i1_1	0.5008074	0.6488262
DN21097_c0_g1_i2_2	6.715352	2.936121
DN21113_c0_g1_i1_1	1.336237	0.2360091
DN21114_c0_g1_i1_1	37.97026	69.45605
DN21125_c0_g1_i1_1	1.687106	1.641076
DN2112_c0_g1_i1_1	1.647844	0.8195796
DN21132_c0_g1_i1_1	1.650557	1.604905
DN21134_c0_g1_i1_1	2.974501	6.340156
DN21137_c0_g1_i1_1	0	0.5155971
DN21137_c0_g1_i1_2	5.575324	4.844553
DN21143_c0_g1_i1_1	0.6491473	1.977413
DN21143_c0_g1_i3_2	23.86443	27.38294
DN21143_c0_g2_i1_2	16.45377	18.93714
DN21144_c0_g1_i1_1	13.31264	6.438252
DN21148_c0_g1_i1_1	0.9197988	1.175992
DN21155_c0_g1_i1_1	0.3111617	0.834145
DN2115_c0_g1_i1_2	2.759885	0.3785976
DN21162_c0_g1_i1_1	1.248784	5.877772
DN21164_c0_g1_i1_2	0.6842632	0.3746847
DN21167_c0_g1_i1_1	0	1.146574
DN21167_c0_g2_i2_2	1.695182	0.4769785

DN21174_c0_g1_i1_1	2.663478	9.859521
DN21176_c0_g3_i1_2	3.509688	1.858433
DN21176_c0_g4_i1_2	5.660918	1.557695
DN21179_c0_g1_i1_1	1.260509	1.303976
DN21182_c0_g1_i1_1	0.1294685	4.049612
DN21185_c0_g1_i1_1	1.439377	2.877441
DN21188_c0_g2_i2_2	11.00926	2.930031
DN21189_c0_g1_i1_1	2.971244	3.669366
DN21191_c0_g1_i1_1	1.149047	2.609744
DN21192_c0_g1_i8_2	40.51109	34.10969
DN21192_c0_g2_i6_2	14.22461	14.19534
DN21196_c0_g1_i1_2	3.033604	2.247551
DN21198_c0_g1_i1_1	24.07903	49.60877
DN21199_c0_g1_i1_1	1.937512	2.307303
DN211_c0_g1_i1_1	1.197005	1.790664
DN211_c0_g1_i1_2	8.423877	17.99284
DN21204_c0_g1_i1_1	0.4372337	0.7713738
DN21206_c0_g1_i1_1	0.4599172	1.672039
DN21207_c0_g1_i1_1	2.27E-28	0.002828707
DN21208_c0_g1_i1_1	0.8889603	0.3741476
DN21215_c0_g6_i1_2	2.571201	2.381263
DN21218_c0_g1_i1_1	3.024441	3.128371
DN2121_c0_g1_i1_1	32.63912	31.29084
DN21223_c0_g1_i1_1	0.8957097	1.628473

DN21226_c0_g1_i1_1	0.5987722	0.7432004
DN21227_c0_g3_i2_2	76.03309	96.93636
DN21231_c0_g1_i1_1	0	0
DN2123_c0_g1_i1_1	1.672719	1.838128
DN21247_c0_g1_i1_1	1.156083	1.211287
DN21250_c0_g1_i1_1	1.853849	1.621782
DN21251_c0_g1_i1_1	32.62832	19.33517
DN21254_c0_g1_i1_1	1.010062	0.2447875
DN21256_c0_g1_i1_1	0.5431452	0.5493997
DN21259_c0_g1_i1_1	1.123855	0.4463776
DN21265_c0_g1_i1_1	1.113547	0.1841203
DN21266_c0_g1_i1_1	0.303447	0
DN21267_c0_g1_i1_1	0.06788711	2.208233
DN21269_c0_g1_i1_1	0	1.43487
DN21269_c2_g1_i1_2	2.395018	0.6447112
DN21269_c3_g1_i1_2	2.404081	1.425806
DN21269_c4_g5_i1_2	3.250804	0.7932611
DN21274_c0_g1_i1_1	18.53216	14.20544
DN21277_c0_g1_i1_1	8.111181	4.209908
DN21277_c0_g2_i1_2	2.455046	0.9869209
DN21279_c0_g1_i1_1	0.8769827	0.6905605
DN2127_c0_g1_i1_1	0.9263696	1.00686
DN21284_c0_g1_i1_1	0.3426443	0.8542498
DN21289_c0_g1_i1_1	0.2400315	0.5913215

DN21296_c0_g1_i1_1	0.9661646	0.6972549
DN21298_c0_g1_i1_1	0.5793724	1.902187
DN21299_c0_g1_i1_1	0.8402188	2.309185
DN21302_c0_g1_i7_2	2.735144	2.741352
DN21302_c0_g2_i1_2	4.04407	4.012543
DN21302_c0_g3_i1_2	2.15099	1.585644
DN21303_c0_g1_i1_1	2.316465	0.7175072
DN21309_c0_g1_i1_1	0	0.5827355
DN2130_c0_g1_i1_1	0	1.52933
DN21312_c0_g1_i1_2	2.544732	0.7932356
DN21313_c0_g1_i1_1	0	0
DN21314_c0_g1_i1_1	0.9062917	0.4176222
DN21316_c0_g1_i1_1	0.2938247	1.010035
DN21320_c0_g5_i1_2	5.299755	1.383126
DN21320_c0_g8_i1_2	4.719798	1.292432
DN21321_c0_g1_i3_2	5.508472	7.368623
DN21323_c0_g1_i1_2	4.400086	24.44596
DN21329_c0_g1_i1_1	0.70759	2.427631
DN21331_c0_g1_i1_1	0.7805843	1.145934
DN21331_c0_g1_i9_2	8.104872	3.444598
DN21333_c0_g1_i1_1	1.10328	1.72419
DN21335_c0_g1_i1_1	0	1.032891
DN21339_c0_g1_i1_1	6.985131	3.963532
DN21344_c0_g2_i1_2	4.015326	0.5094441

DN21346_c0_g1_i1_1	1.197107	1.156057
DN2134_c0_g2_i1_1	0.1849331	0.8193441
DN21351_c0_g1_i1_1	0	0.8325923
DN21352_c0_g1_i1_1	1.18172	0.5299736
DN21353_c2_g1_i1_2	1.316336	0.642071
DN21356_c1_g1_i1_2	31.04866	6.174474
DN21356_c1_g2_i2_2	9.34374	4.708563
DN21358_c0_g1_i1_1	1.722239	2.875118
DN21361_c0_g1_i1_1	6.339364	7.609521
DN21362_c0_g1_i1_1	2.481586	1.249533
DN21363_c0_g1_i1_1	2.213798	6.226095
DN21365_c0_g3_i1_2	0.9044254	0.5116717
DN21367_c0_g1_i1_1	0.2040613	0.645577
DN21367_c2_g11_i2_2	20.05456	8.813306
DN21367_c2_g1_i1_2	1.850243	0.5773215
DN21367_c2_g3_i1_2	1.451917	0.5466141
DN21367_c2_g4_i1_2	2.807807	0.6520859
DN21369_c1_g7_i1_2	0.9671192	0.154264
DN21372_c0_g1_i5_2	72.20308	152.1557
DN21376_c0_g1_i2_2	32.61572	0.6214244
DN21377_c0_g1_i1_1	0.9230169	0.7300708
DN21379_c0_g1_i1_1	2.563353	5.832173
DN21380_c0_g1_i1_1	0.7550059	1.232882
DN21383_c0_g1_i1_1	0.5761766	3.052501

DN21384_c1_g5_i1_2	6.220297	0.8025201
DN2138_c0_g1_i1_2	20.83072	33.81492
DN21394_c0_g1_i1_1	1.0329	1.008485
DN21396_c0_g1_i1_1	1.656231	3.803769
DN21399_c0_g1_i1_1	0.8878878	0.5341397
DN21399_c0_g1_i3_2	1.162704	1.078705
DN21399_c0_g2_i3_2	1.443145	1.233357
DN21401_c0_g1_i1_1	10.92987	6.263131
DN21402_c0_g1_i1_1	9.371605	10.18022
DN21403_c0_g1_i1_1	0.7345028	0.4708043
DN21404_c0_g1_i1_1	0.9148431	2.117332
DN21406_c0_g1_i1_1	1.233398	1.336185
DN21407_c0_g1_i1_1	8.039304	2.267956
DN2140_c0_g1_i1_1	1.134835	3.30861
DN21413_c0_g1_i1_1	12.19279	5.882638
DN21414_c0_g1_i1_1	0	1.170155
DN21416_c0_g1_i1_1	3.204776	0.9865343
DN21418_c0_g1_i1_1	0.8479207	0.8376517
DN2141_c0_g1_i2_1	13.77006	5.71606
DN2141_c0_g1_i2_2	2.67883	2.286042
DN21422_c0_g1_i1_2	1.874098	0.8178929
DN21422_c0_g2_i1_2	1.858593	1.175575
DN21422_c1_g1_i1_2	0.507817	0.3376773
DN21422_c1_g2_i1_2	0.9242663	0.3887579

DN21422_c2_g6_i1_2	5.316633	0.9716465
DN21425_c0_g1_i1_1	1.45882	3.523557
DN21427_c0_g1_i1_1	0.5261293	1.772045
DN21430_c0_g1_i1_1	1.231238	1.845813
DN21431_c0_g1_i1_1	0.4322502	0.9545349
DN21432_c0_g1_i1_1	3.791282	1.53279
DN21433_c0_g1_i1_1	0.4186325	0.5748857
DN21436_c0_g1_i1_1	2.055829	1.029304
DN21443_c0_g1_i1_1	0.6645975	0.6156243
DN21446_c1_g1_i2_2	19.31021	13.25751
DN21448_c0_g1_i1_1	4.119477	3.744264
DN21448_c0_g1_i2_2	28.97155	42.34632
DN21450_c0_g1_i1_1	1.112168	4.298317
DN21451_c0_g1_i1_1	0.431279	0.3930559
DN21458_c0_g1_i1_1	0.4521512	1.313488
DN21459_c0_g1_i1_1	0.6032915	1.942783
DN2145_c0_g1_i1_1	2.334886	2.429831
DN2145_c0_g1_i1_2	1.29623	0.1775957
DN21460_c0_g1_i1_1	2.803253	0.3708731
DN21461_c0_g1_i1_1	0	0.2794944
DN21499_c0_g1_i1_1	0.8599505	0.525273
DN2149_c0_g1_i1_1	1.28088	1.409
DN21502_c0_g2_i1_2	2.641842	1.456199
DN21506_c0_g1_i1_1	0.305473	2.076291

DN21507_c0_g1_i1_1	0.3328003	2.031412
DN2150_c0_g1_i1_2	0	0
DN21510_c0_g1_i1_1	45.19246	88.36916
DN21511_c0_g1_i1_1	0.8146363	1.688028
DN21515_c0_g1_i1_1	0	0.586372
DN21516_c0_g1_i1_1	1.939175	0.313842
DN2151_c0_g1_i1_1	1.3834	0.1635053
DN21521_c0_g1_i1_1	0.5832367	2.242825
DN21523_c0_g1_i1_1	2.744156	4.671431
DN21529_c0_g1_i1_1	1.384198	1.304756
DN21531_c0_g1_i1_1	0.564017	2.733443
DN21534_c0_g1_i1_1	3.299304	8.029385
DN21538_c0_g2_i1_2	0.9676637	0.7220751
DN21544_c0_g1_i1_2	3.241518	0.7350639
DN21544_c1_g1_i14_2	11.88258	5.475805
DN21545_c1_g2_i1_2	3.405972	0.4766431
DN21547_c0_g1_i1_1	0.2173067	1.046204
DN21547_c2_g2_i2_2	3.257536	1.949073
DN21551_c0_g1_i1_1	3.126657	1.153329
DN21552_c0_g1_i1_1	1.57159	1.162999
DN21553_c3_g2_i1_2	10.99527	3.894653
DN21554_c0_g1_i1_1	1.704964	2.373868
DN21554_c2_g1_i1_2	1.817739	0.3944695
DN21559_c0_g1_i1_1	1.681972	1.282313

DN21567_c0_g1_i1_1	1.538054	2.040471
DN21568_c0_g1_i1_1	1.442463	1.332628
DN21571_c0_g1_i1_1	0.2023643	0.9403801
DN21572_c0_g1_i1_1	1.396291	1.037976
DN21573_c0_g1_i1_1	1.079555	1.092109
DN21575_c0_g1_i1_1	3.867369	2.250656
DN21578_c0_g1_i1_1	2.359617	1.219406
DN21585_c0_g1_i1_1	1.492578	3.234101
DN21588_c0_g1_i1_1	2.55899	4.430738
DN21591_c0_g1_i1_1	12.19322	6.122261
DN21597_c0_g1_i1_1	0.07561161	0.7696801
DN21598_c0_g1_i1_1	0.3838496	1.110633
DN21599_c2_g1_i1_2	0.8920722	0.4308104
DN2159_c0_g1_i1_1	0.3106686	0.1583506
DN21600_c0_g1_i1_1	0	3.986643
DN21603_c0_g1_i1_1	1.583353	0.7438316
DN21606_c0_g1_i1_1	0.6055141	1.117805
DN2160_c0_g1_i1_1	1.11711	0.9849365
DN21613_c0_g1_i1_1	23.15164	7.940567
DN21614_c0_g1_i1_1	0.9849617	0.39546
DN21616_c0_g1_i1_1	1.406528	8.984665
DN21617_c0_g1_i1_1	0.7161681	1.128377
DN21618_c0_g1_i1_1	0	1.29738
DN21619_c0_g1_i1_1	0.3411813	0.4719954

DN21621_c0_g1_i1_1	1.175692	0.2562637
DN21622_c0_g1_i1_2	2.245102	1.968564
DN21622_c1_g1_i8_2	14.44208	3.310541
DN21624_c0_g1_i1_1	0.2929142	0.9029171
DN21626_c0_g2_i1_2	5.241845	3.236679
DN21627_c1_g5_i1_2	1.865509	1.22869
DN21630_c2_g7_i1_2	8.04514	4.196331
DN21631_c0_g1_i1_1	1.663101	1.170193
DN21638_c0_g1_i1_1	4.596556	13.65006
DN2163_c0_g1_i1_1	2.599054	10.45407
DN21640_c0_g1_i1_1	1.248114	1.200181
DN21642_c0_g1_i1_1	24.27249	35.20774
DN21643_c0_g1_i1_1	1.238437	1.995308
DN21650_c0_g2_i3_2	9.485817	4.495177
DN21653_c0_g1_i1_1	0.9659011	1.48214
DN21658_c0_g1_i1_1	9.776362	12.45957
DN21659_c0_g1_i1_1	1.315049	0.7391033
DN21660_c0_g1_i1_1	0	0
DN21664_c0_g1_i1_1	0.3868447	2.212213
DN21664_c0_g1_i1_2	2.919252	1.738585
DN21666_c0_g1_i1_2	26.97836	17.68991
DN21669_c0_g1_i1_1	17.0569	46.70799
DN21676_c0_g1_i1_1	1.024807	1.823708
DN2167_c0_g1_i1_1	2.545546	6.902965

DN21681_c0_g1_i1_1	1.378023	1.682795
DN21687_c0_g1_i1_1	8.568735	4.637742
DN21688_c0_g1_i1_1	306.7719	87.94177
DN21690_c0_g1_i1_1	3.432688	2.464982
DN21692_c0_g1_i1_2	14.02035	1.475292
DN21694_c0_g1_i1_1	0.6962505	1.405004
DN21695_c0_g1_i1_1	1.513736	3.339005
DN21696_c0_g1_i1_1	8.548983	33.82643
DN21696_c0_g1_i1_2	7.237568	8.48751
DN21697_c0_g1_i1_1	2.883496	6.26583
DN2169_c0_g1_i1_1	0	0.6705554
DN216_c0_g1_i1_1	2.448823	2.528146
DN21702_c0_g1_i1_1	3.117707	3.028851
DN21707_c0_g1_i1_1	3.176897	1.374758
DN21710_c0_g1_i1_1	1.665526	6.761711
DN21711_c0_g1_i1_1	1.193452	0.5464356
DN21714_c0_g1_i1_1	17.19062	11.64258
DN21718_c0_g1_i1_1	0	0.3948869
DN2171_c0_g1_i1_1	1.329852	4.22645
DN21723_c0_g1_i1_1	1.695357	0.3774074
DN21726_c0_g1_i1_1	58.09346	32.87952
DN21735_c0_g1_i1_1	28.09359	11.91277
DN21742_c0_g1_i1_1	0.2171208	0
DN21744_c0_g1_i1_1	2.299397	9.04E-07

DN21745_c0_g1_i1_1	2.23E-17	1.13721
DN21748_c0_g1_i1_1	2.8327	3.775406
DN21748_c0_g1_i1_2	4.626154	1.020325
DN21752_c0_g1_i1_2	0	0
DN21757_c0_g1_i1_1	0.1637845	0.4463985
DN21758_c0_g1_i1_1	1.360015	2.12212
DN21761_c0_g1_i1_1	0	0
DN21766_c0_g1_i1_1	0	1.232446
DN21769_c0_g1_i1_1	1.170153	1.404374
DN2176_c0_g1_i1_1	2.403781	2.235359
DN21775_c0_g1_i1_1	0.5062809	0.7031412
DN21775_c0_g1_i1_2	14.66122	30.471
DN21777_c0_g1_i1_1	0.5081608	0.8909123
DN21778_c0_g1_i1_1	0	0.4559627
DN21781_c0_g1_i1_2	41.11168	30.12741
DN21785_c0_g1_i1_1	0.6919204	0.6706294
DN21786_c0_g1_i1_1	0.9216537	0
DN21788_c0_g1_i1_1	1.720212	1.159331
DN2178_c0_g1_i1_1	1.98261	2.65626
DN2178_c0_g1_i1_2	1.638994	0.5391263
DN21790_c0_g1_i1_1	1.272839	1.876619
DN21791_c0_g1_i1_1	139.278	289.6841
DN21793_c0_g1_i1_1	2.068757	4.660616
DN21794_c0_g1_i1_1	0.5407427	2.192207

DN21797_c0_g1_i1_1	1.873638	1.75928
DN21798_c0_g1_i1_1	1.228709	3.840963
DN21802_c0_g1_i1_2	0.6060612	0.4229278
DN21804_c0_g1_i1_1	0.2497494	0
DN21810_c0_g1_i1_1	0.2384452	0.2989015
DN21811_c0_g1_i1_1	0.7228863	3.927921
DN21813_c0_g1_i1_1	0	2.502488
DN21814_c0_g1_i1_1	0	0.6014867
DN21815_c0_g1_i1_1	0	1.726099
DN21828_c0_g1_i1_1	7.758231	7.871658
DN21830_c0_g1_i1_1	0.7048655	1.447512
DN21833_c0_g1_i1_1	0.9399634	1.625419
DN21836_c0_g1_i1_1	18.50361	13.10274
DN21842_c0_g1_i1_2	0.5408476	1.07444
DN21847_c0_g1_i1_1	1.135278	1.458804
DN2184_c0_g1_i1_1	0.1815021	0.84035
DN21852_c0_g1_i1_1	0	1.244905
DN21855_c0_g1_i1_1	6.94114	5.854273
DN21855_c0_g1_i1_2	1.657545	0
DN21856_c0_g1_i1_1	0.830647	0.8378767
DN21857_c0_g1_i1_2	1.954067	0.1747048
DN21859_c0_g1_i1_2	5.747615	0.6006221
DN21861_c0_g1_i1_1	4.741932	16.13552
DN21861_c0_g1_i1_2	4.034278	3.849119

DN21862_c0_g1_i1_2	0.7154986	0.723186
DN21864_c0_g1_i1_1	2.058294	2.791905
DN2186_c0_g1_i1_1	1.065904	6.010437
DN21871_c0_g1_i1_2	2.460701	2.892763
DN21872_c0_g1_i1_2	18.01292	32.43473
DN21873_c0_g1_i1_1	1.089094	3.041621
DN2188_c0_g1_i1_1	1.692879	0.4680627
DN21890_c0_g1_i1_2	4.949933	0.9158321
DN21898_c0_g1_i1_1	1.12007	2.64255
DN2189_c0_g1_i1_1	3.264664	10.04236
DN21904_c0_g1_i1_1	0.6823039	26.31236
DN21905_c0_g1_i1_2	2.007963	1.54489
DN21906_c0_g1_i1_2	3.936824	0.6238951
DN2190_c0_g1_i1_1	4.861658	1.312616
DN21911_c0_g1_i1_2	1.714554	2.236456
DN21914_c0_g1_i1_1	0.3616201	1.321137
DN21922_c0_g1_i1_1	0	0.7068476
DN21923_c0_g1_i1_1	7.210649	5.952167
DN21924_c0_g1_i1_1	0.6680564	1.647988
DN21926_c0_g1_i1_2	10.07263	9.282397
DN21930_c0_g1_i1_1	0.5663699	1.001915
DN21939_c0_g1_i1_1	0	1.659076
DN2193_c0_g1_i1_2	6.104019	4.384969
DN21940_c0_g1_i1_1	12.35218	7.359576

DN21944_c0_g1_i1_2	1.89957	0.5433352
DN21953_c0_g1_i1_1	13.41896	28.32467
DN21954_c0_g1_i1_1	7.19735	33.94743
DN21954_c0_g1_i1_2	30.94528	2.982337
DN21958_c0_g1_i1_2	2.246251	1.857487
DN21968_c0_g1_i1_1	1.096718	1.587157
DN21970_c0_g1_i1_1	1.87712	2.34318
DN21975_c0_g1_i1_2	0.5700874	0.2694062
DN21978_c0_g1_i1_1	2.758606	1.711013
DN21979_c0_g1_i1_1	2.204064	18.84278
DN21982_c0_g1_i1_1	0.1869223	1.576322
DN21990_c0_g1_i1_1	0.2766192	0.4177954
DN21991_c0_g1_i1_2	3.806256	0.9089003
DN21996_c0_g1_i1_1	0	1.508135
DN21998_c0_g1_i1_1	0.707546	1.22736
DN21999_c0_g1_i1_1	0.9671637	0
DN22000_c0_g1_i1_2	2.783538	1.548313
DN22002_c0_g1_i1_1	1.281226	1.319705
DN22006_c0_g1_i1_1	0.3506479	0.9258211
DN22007_c0_g1_i1_1	2.592221	5.284987
DN22009_c0_g1_i1_1	1.032031	2.13698
DN2200_c0_g1_i1_1	0.4688897	1.585526
DN22010_c0_g1_i1_2	1.220086	1.40773
DN22013_c0_g1_i1_1	2.552089	2.011376

DN22017_c0_g1_i1_1	0.2031345	1.430746
DN22017_c0_g1_i1_2	3.399586	1.295434
DN22018_c0_g1_i1_2	4.205018	1.938245
DN22019_c0_g1_i1_2	14.56249	12.46928
DN22022_c0_g1_i1_1	1.274732	0
DN22023_c0_g1_i1_2	12.88804	10.7611
DN2202_c0_g1_i1_1	4.909568	8.729054
DN22030_c0_g1_i1_1	2.313979	1.094655
DN22030_c0_g1_i1_2	2.577459	2.100532
DN22033_c0_g1_i1_1	0	1.857626
DN22037_c0_g1_i1_2	2.646469	0.2076866
DN2203_c0_g1_i1_1	1.437029	2.183176
DN22044_c0_g1_i1_2	3.554634	1.000806
DN22058_c0_g1_i1_1	5.287155	28.23313
DN22059_c0_g1_i1_1	0.7038138	1.331845
DN22060_c0_g1_i1_2	5.123529	6.458099
DN22061_c0_g1_i1_1	5.083795	5.607743
DN22062_c0_g1_i1_1	2.702861	5.419075
DN22064_c0_g1_i1_1	3.39873	2.848164
DN22071_c0_g1_i1_1	10.62799	11.09861
DN22073_c0_g1_i1_2	2.225471	1.899866
DN22075_c0_g1_i1_2	1.862106	3.94E-31
DN2207_c0_g1_i1_1	2.997753	3.994846
DN22084_c0_g1_i1_1	1.550218	0.5905815

DN22086_c0_g1_i1_1	0.7884389	1.032962
DN22087_c0_g1_i1_1	6.877083	12.87399
DN2208_c0_g1_i1_1	1.030809	3.704347
DN22090_c0_g1_i1_1	0.4413091	0.9797665
DN22093_c0_g1_i1_2	2.485319	0.7024683
DN22094_c0_g1_i1_2	95.23176	355.6475
DN22099_c0_g1_i1_1	0.6060508	0.03695201
DN22099_c0_g1_i1_2	1.521101	0.5312381
DN2209_c0_g1_i1_1	2.526763	2.818561
DN22109_c0_g1_i1_1	0.8314271	1.307234
DN2210_c0_g1_i1_2	2.348868	0.8796687
DN22113_c0_g1_i1_2	0.7869965	1.276082
DN22118_c0_g1_i1_1	1.267567	0.8363238
DN22123_c0_g1_i1_1	1.368162	1.311328
DN22123_c0_g1_i1_2	3.52752	0.1716609
DN22131_c0_g1_i1_2	1.721017	3.413132
DN22132_c0_g1_i1_1	2.575736	3.047166
DN22135_c0_g1_i1_1	5.457385	4.578056
DN22135_c0_g1_i1_2	1.283243	1.955576
DN22136_c0_g1_i1_1	15.05077	14.21915
DN22142_c0_g1_i1_2	30.42912	91.30648
DN22145_c0_g1_i1_2	0.07269731	0.4351561
DN22147_c0_g1_i1_1	13.69804	27.32045
DN22150_c0_g1_i1_2	2.959171	1.827849

DN22151_c0_g1_i1_1	6.890584	5.866401
DN22153_c0_g1_i1_1	0	5.617844
DN22153_c0_g1_i1_2	3.782234	2.493338
DN22159_c0_g1_i1_1	14.78509	17.28448
DN2215_c0_g1_i1_1	0.7492157	1.282369
DN22160_c0_g1_i1_1	0	1.514939
DN22167_c0_g1_i1_1	2.144285	1.603179
DN22168_c0_g1_i1_2	5.44751	2.423881
DN2216_c0_g1_i1_1	0.9393322	1.109379
DN22173_c0_g1_i1_1	3.669302	2.462022
DN22175_c0_g1_i1_1	5.197974	5.60979
DN22175_c0_g1_i1_2	4.294289	0.7021806
DN22178_c0_g1_i1_2	4.733496	1.756324
DN22183_c0_g1_i1_1	10.99235	50.7972
DN22186_c0_g1_i1_1	1.103008	1.054606
DN22195_c0_g1_i1_1	1.259342	4.238676
DN22196_c0_g1_i1_2	0.6539287	1.354412
DN22199_c0_g1_i1_1	1.241557	1.753163
DN2219_c0_g1_i1_1	1.467135	1.921135
DN221_c0_g1_i1_2	5.074259	3.882721
DN22200_c0_g1_i1_1	1.088153	3.043583
DN22200_c0_g1_i1_2	0.4905491	0.662092
DN22202_c0_g1_i1_1	0	0.2564077
DN22206_c0_g1_i1_2	3.817853	0.8146173

DN22210_c0_g1_i1_1	6.680442	5.443799
DN22215_c0_g1_i1_2	7.743785	1.001624
DN22224_c0_g1_i1_2	17.87563	8.367933
DN22225_c0_g1_i1_1	6.186054	12.19825
DN22230_c0_g1_i1_2	1.991164	0.3706295
DN22238_c0_g1_i1_2	0.1578277	0.6551546
DN22239_c0_g1_i1_1	0.5547358	0.8991376
DN2223_c0_g1_i1_1	7.327306	4.556774
DN22240_c0_g1_i1_1	1.248854	3.340892
DN22244_c0_g1_i1_1	1.434049	1.007275
DN22244_c0_g1_i1_2	179.9965	92.28118
DN22252_c0_g1_i1_2	0	0
DN22253_c0_g1_i1_1	4.259042	4.597179
DN22257_c0_g1_i1_1	1.306582	1.960933
DN22258_c0_g1_i1_1	0.3978984	2.816213
DN22261_c0_g1_i1_1	4.193594	3.315492
DN22261_c0_g1_i1_2	1.325434	1.031523
DN22262_c0_g1_i1_2	3.837234	2.803958
DN22263_c0_g1_i1_1	1.912847	1.304641
DN22264_c0_g1_i1_2	2.548304	1.523557
DN22265_c0_g1_i1_2	18.05365	37.34877
DN22266_c0_g1_i1_1	3.828078	5.71229
DN22270_c0_g1_i1_1	53.76413	61.91713
DN22273_c0_g1_i1_2	4.802198	3.617503

DN22274_c0_g1_i1_1	0.3594711	0.7946791
DN22275_c0_g1_i1_1	0.7251585	1.277515
DN22276_c0_g1_i1_2	29.12573	16.09253
DN22277_c0_g1_i1_2	3.662793	3.163102
DN22282_c0_g1_i1_1	2.874825	3.367258
DN22287_c0_g1_i1_2	1.419468	0
DN22290_c0_g1_i1_1	0.3841583	0.8449656
DN22298_c0_g1_i1_2	65.7735	7.346153
DN22299_c0_g1_i1_1	25.70473	13.95572
DN2229_c0_g1_i1_1	0.2049664	1.149665
DN222_c0_g1_i1_2	2.429526	0
DN22301_c0_g1_i1_1	0.6503715	0.7210337
DN22303_c0_g1_i1_1	28.3235	37.23694
DN22308_c0_g1_i1_1	2.187726	1.712634
DN22314_c0_g1_i1_2	13.26617	4.203432
DN22315_c0_g1_i1_1	0.255127	0.6431192
DN22324_c0_g1_i1_1	10.53822	17.03592
DN22331_c0_g1_i1_2	14.27926	15.76506
DN22332_c0_g1_i1_1	0.259665	1.639419
DN22332_c0_g1_i1_2	3.845626	0
DN22337_c0_g1_i1_1	2.256814	0.4838823
DN22339_c0_g1_i1_1	6.171411	3.944302
DN22345_c0_g1_i1_2	0.4274394	0.7064115
DN22347_c0_g1_i1_1	1.05509	1.561105

DN22347_c0_g1_i1_2	4.458993	1.007686
DN22352_c0_g1_i1_1	1.283965	1.332338
DN22355_c0_g1_i1_1	2.071047	4.800581
DN22356_c0_g1_i1_2	5.448092	0.9360325
DN22357_c0_g1_i1_2	2.982956	2.512898
DN2235_c0_g1_i1_1	0.0804004	0.9995083
DN22361_c0_g1_i1_2	70.7035	109.9027
DN22362_c0_g1_i1_1	2.693387	1.779882
DN22364_c0_g1_i1_1	1.109234	0.8005101
DN22367_c0_g1_i1_1	2.138509	1.57575
DN22380_c0_g1_i1_1	0.3374236	0.6135752
DN22388_c0_g1_i1_1	3.56614	4.482471
DN22392_c0_g1_i1_1	4.129829	7.659172
DN22399_c0_g1_i1_1	0.6813364	5.849055
DN22401_c0_g1_i1_1	2.004858	1.907101
DN22403_c0_g1_i1_1	2.218755	1.217606
DN22403_c0_g1_i1_2	1.186975	1.668761
DN22407_c0_g1_i1_1	6.803638	8.728648
DN2240_c0_g1_i1_1	0.2732763	2.319483
DN22410_c0_g1_i1_2	3.093279	0.9491027
DN22412_c0_g1_i1_1	0.8146495	0.911629
DN22412_c0_g1_i1_2	2.934016	7.07342
DN22416_c0_g1_i1_2	0	0
DN22417_c0_g1_i1_1	1.44299	6.655954

DN22420_c0_g1_i1_1	0.7554852	1.080732
DN22421_c0_g1_i1_2	2.573985	0.9351484
DN22424_c0_g1_i1_1	0.5868372	1.865332
DN22425_c0_g1_i1_1	3.03953	1.538396
DN22425_c0_g1_i1_2	4.987172	1.539424
DN2242_c0_g1_i1_2	9.850569	3.556324
DN22432_c0_g1_i1_1	4.755415	6.305735
DN22436_c0_g1_i1_2	0.803109	0.3217236
DN22439_c0_g1_i1_2	2604.255	1396.529
DN22442_c0_g1_i1_1	1.220623	4.555668
DN22442_c0_g1_i1_2	0.9640998	0.1082556
DN22443_c0_g1_i1_1	2.929247	7.088337
DN22445_c0_g1_i1_2	7.689726	2.850217
DN22450_c0_g1_i1_1	2.948718	2.093695
DN22455_c0_g1_i1_2	2.130007	2.339092
DN22456_c0_g1_i1_2	3.294268	2.884442
DN22458_c0_g1_i1_1	24.71976	12.75018
DN22464_c0_g1_i1_2	0.4149745	0.5346971
DN22468_c0_g1_i1_2	1.308937	1.797427
DN22470_c0_g1_i1_1	3.11193	11.83174
DN22475_c0_g1_i1_1	14.08614	16.40742
DN22480_c0_g1_i1_2	1.246073	2.442844
DN22481_c0_g1_i1_1	0.7101588	0
DN22483_c0_g1_i1_1	41.097	20.29582

DN22484_c0_g1_i1_1	0.9302584	2.256522
DN22486_c0_g1_i1_1	1.740414	10.67618
DN2248_c0_g2_i1_1	1.879746	1.93481
DN22490_c0_g1_i1_1	0.2449443	2.480277
DN22492_c0_g1_i1_2	8.096556	4.125115
DN22495_c0_g1_i1_1	0.1672054	1.40639
DN22496_c0_g1_i1_1	6.922894	2.250407
DN22498_c0_g1_i1_1	0.2280131	1.61225
DN22498_c0_g1_i1_2	0	0
DN2249_c0_g1_i1_2	5.228134	3.405397
DN22502_c0_g1_i1_1	0.5969464	1.237902
DN22502_c0_g1_i1_2	2.245337	0.2773104
DN22505_c0_g1_i1_2	0	0
DN22507_c0_g1_i1_1	0.494597	1.39077
DN22509_c0_g1_i1_2	2.850208	0.4517471
DN22512_c0_g1_i1_2	1.841392	1.333269
DN22513_c0_g1_i1_2	256.6387	11.16005
DN22514_c0_g1_i1_1	1.499261	2.676474
DN22515_c0_g1_i1_2	0	0
DN22516_c0_g1_i1_1	19.20096	8.552472
DN22517_c0_g1_i1_2	0.7626735	0.5589903
DN22518_c0_g1_i1_1	4.251927	10.2679
DN22521_c0_g1_i1_1	1.301811	1.378683
DN22524_c0_g1_i1_1	42.34081	149.7828

DN22529_c0_g1_i1_2	7.686939	2.322962
DN22531_c0_g1_i1_1	11.3736	22.22501
DN22532_c0_g1_i1_2	4.215323	1.371851
DN22534_c0_g1_i1_1	7.881131	38.38035
DN22536_c0_g1_i1_1	13.06448	11.27525
DN2253_c0_g1_i1_1	1.181395	1.794769
DN2253_c0_g2_i1_1	2.188754	3.415917
DN22542_c0_g1_i1_2	2.612917	0.9571895
DN22548_c0_g1_i1_2	1.958689	2.703944
DN2254_c0_g1_i1_2	5.081176	1.17987
DN22550_c0_g1_i1_2	0	0
DN22553_c0_g1_i1_1	28.73871	91.77417
DN22554_c0_g1_i1_1	0.8548352	1.610682
DN22555_c0_g1_i1_2	1.221414	1.629875
DN2255_c0_g1_i1_1	2.642126	3.706101
DN22565_c0_g1_i1_2	1.035757	0.5532284
DN22566_c0_g1_i1_1	1.456551	1.849482
DN22570_c0_g1_i1_1	21.00864	7.75358
DN22585_c0_g1_i1_1	0.7623334	1.26
DN22586_c0_g1_i1_2	3.882121	2.231806
DN22588_c0_g1_i1_1	0.3917372	0.4850348
DN22588_c0_g1_i1_2	1.665878	0.7436244
DN22591_c0_g1_i1_2	2.600783	0.8636766
DN22592_c0_g1_i1_1	12.86003	57.15961

DN22603_c0_g1_i1_1	1.510217	1.039068
DN22606_c0_g1_i1_1	7.341079	11.04085
DN22612_c0_g1_i1_2	3.123517	0.6922182
DN22614_c0_g1_i1_1	0.08816727	0.4812349
DN22616_c0_g1_i1_1	17.21948	24.58892
DN22618_c0_g1_i1_1	0.3709154	1.076837
DN2261_c0_g1_i1_2	2.330321	1.603918
DN22620_c0_g1_i1_2	7.7163	8.366884
DN22622_c0_g1_i1_2	0.8008789	1.36025
DN22628_c0_g1_i1_2	8.035282	2.404893
DN22629_c0_g1_i1_2	1.133797	0.3774271
DN22633_c0_g1_i1_1	0.8504327	1.316831
DN22633_c0_g1_i1_2	9.678586	12.39207
DN22634_c0_g1_i1_1	1.736964	1.149769
DN22636_c0_g1_i1_1	1.333097	1.527054
DN22642_c0_g1_i1_1	6.131254	14.17374
DN22644_c0_g1_i1_1	0	0
DN22647_c0_g1_i1_1	0.2302158	3.07615
DN22648_c0_g1_i1_1	2.006208	3.346174
DN22653_c0_g1_i1_1	0.4539571	1.437407
DN22656_c0_g1_i1_2	0	0
DN22659_c0_g1_i1_1	2.62	3.534714
DN22663_c0_g1_i1_2	2.242502	0.9226814
DN22664_c0_g1_i1_1	2.855047	1.243328

DN22665_c0_g1_i1_1	0.2592216	0.3781952
DN22666_c0_g1_i1_2	0.7881084	0.5122502
DN2266_c0_g1_i1_1	2.325862	0.9366034
DN22672_c0_g1_i1_2	4.153298	11.74346
DN22673_c0_g1_i1_1	2.360517	2.704437
DN22675_c0_g1_i1_1	2.237958	0.4611137
DN22675_c0_g1_i1_2	2.057099	0.2701475
DN22676_c0_g1_i1_1	0	0.5456574
DN22677_c0_g1_i1_2	6.130644	3.629431
DN22678_c0_g1_i1_1	0.6274407	0.8292379
DN22685_c0_g1_i1_1	1.145973	1.878193
DN22686_c0_g1_i1_1	1.800651	2.442716
DN22691_c0_g1_i1_1	2.473765	2.81358
DN22692_c0_g1_i1_1	4.295064	0.9748721
DN22693_c0_g1_i1_1	3.177961	6.599411
DN22693_c0_g1_i1_2	2.770589	2.367539
DN22697_c0_g1_i1_1	1.231079	2.403361
DN22701_c0_g1_i1_2	58.70289	112.4539
DN22705_c0_g1_i1_1	1.100469	0.7358333
DN22706_c0_g1_i1_1	4.881543	3.010542
DN22713_c0_g1_i1_2	0.1379382	0.1886013
DN22715_c0_g1_i1_1	0.4927177	4.878771
DN22715_c0_g1_i1_2	2.677105	1.953201
DN22717_c0_g1_i1_2	1.420781	0.3966653

DN2271_c0_g1_i1_1	0.736341	0.9676134
DN22730_c0_g1_i1_1	3.698975	1.601429
DN22730_c0_g1_i1_2	0.910644	0.435252
DN22734_c0_g1_i1_2	2.29786	0.1809056
DN22735_c0_g1_i1_1	4.844253	0.7748065
DN22736_c0_g1_i1_2	3.572158	1.420264
DN22737_c0_g1_i1_2	27.53529	27.26326
DN22739_c0_g1_i1_2	3.229554	1.677475
DN22743_c0_g1_i1_1	40.46977	15.74367
DN22746_c0_g1_i1_1	2.404155	3.067363
DN22746_c0_g1_i1_2	2.43245	0.8412489
DN22753_c0_g1_i1_2	0.554676	0.2743405
DN22760_c0_g1_i1_2	1.614733	0.693833
DN22763_c0_g1_i1_1	3.989692	6.200909
DN22764_c0_g1_i1_1	4.95249	2.023364
DN22768_c0_g1_i1_1	0.3235539	0.3132933
DN2276_c0_g1_i1_1	9579.81	2824.454
DN22777_c0_g1_i1_1	2.403421	1.858121
DN22784_c0_g1_i1_1	1.301847	4.662708
DN22784_c0_g1_i1_2	1.053091	0.4195611
DN22787_c0_g1_i1_1	1.369316	1.776383
DN22789_c0_g1_i1_2	1412.012	1635.069
DN22792_c0_g1_i1_2	3.364709	1.296507
DN22800_c0_g1_i1_2	6.746298	8.300871

DN22802_c0_g1_i1_1	2.957105	2.670028
DN22807_c0_g1_i1_1	0.9952543	24.8306
DN22808_c0_g1_i1_2	4.48723	1.247273
DN2280_c0_g1_i1_1	15.53846	62.44822
DN22810_c0_g1_i1_1	25.86391	41.96531
DN22811_c0_g1_i1_1	3.505753	3.118171
DN22812_c0_g1_i1_1	8.856308	2.620056
DN22815_c0_g1_i1_1	0.3099083	0.06000423
DN22816_c0_g1_i1_1	3.414252	16.56004
DN22817_c0_g1_i1_1	1.148062	13.32823
DN22826_c0_g1_i1_2	1.776275	2.285478
DN22827_c0_g1_i1_1	3.483136	3.251547
DN22831_c0_g1_i1_1	3.335404	6.748819
DN22834_c0_g1_i1_1	12.30522	3.439906
DN22835_c0_g1_i1_2	3.597176	0
DN22839_c0_g1_i1_2	1.603934	1.46352
DN2283_c0_g2_i1_1	0.6723862	0.5917896
DN22845_c0_g1_i1_1	0	0.6414205
DN22847_c0_g1_i1_2	4.294387	3.631633
DN22852_c0_g1_i1_1	1.75764	2.878379
DN22856_c0_g1_i1_1	1.831918	2.962632
DN22858_c0_g1_i1_1	1.334343	2.512936
DN22861_c0_g1_i1_2	0.7365634	0.7933494
DN22865_c0_g1_i1_2	2.855613	0.9119863

DN22870_c0_g1_i1_1	5.648011	4.401416
DN22878_c0_g1_i1_1	42.72772	23.31014
DN22880_c0_g1_i1_1	0.9178215	3.667978
DN22881_c0_g1_i1_1	0.5828983	2.095447
DN22881_c0_g1_i1_2	3.155971	0
DN22887_c0_g1_i1_2	1.979613	1.290106
DN22889_c0_g1_i1_1	1.49804	2.441608
DN2288_c0_g1_i1_1	1.793024	1.793045
DN22891_c0_g1_i1_1	0.3024345	1.432025
DN22893_c0_g1_i1_1	1.634771	1.216628
DN22897_c0_g1_i1_1	0.3392555	0.4800253
DN22898_c0_g1_i1_1	0.8584772	2.46136
DN22898_c0_g1_i1_2	23.46597	10.74203
DN22904_c0_g1_i1_1	1.33322	6.34622
DN22911_c0_g1_i1_1	1.216821	2.410732
DN22914_c0_g1_i1_1	0	1.84765
DN22916_c0_g1_i1_2	4.454142	5.15967
DN22917_c0_g1_i1_2	2.143633	0.6463565
DN22919_c0_g1_i1_2	63.08708	28.28089
DN22921_c0_g1_i1_2	3.482742	2.33759
DN22923_c0_g1_i1_1	0.4909851	2.697512
DN22928_c0_g1_i1_2	1.266398	0.1241855
DN22929_c0_g1_i1_1	0.7689269	1.485012
DN22932_c0_g1_i1_1	1.542633	2.163343

DN22934_c0_g1_i1_1	1.350382	1.142584
DN22940_c0_g1_i1_1	0.7924168	0.8069941
DN22942_c0_g1_i1_1	1.240224	6.956134
DN22944_c0_g1_i1_2	11.63184	1.408037
DN22946_c0_g1_i1_1	2.297206	3.315015
DN2294_c0_g1_i1_1	0.3423128	2.622627
DN2294_c0_g2_i1_1	0	2.50559
DN22950_c0_g1_i1_1	1.145376	0.9625215
DN22952_c0_g1_i1_2	0.7110973	0
DN22954_c0_g1_i1_2	34.92608	14.04265
DN22957_c0_g1_i1_1	30.35943	17.68499
DN22958_c0_g1_i1_1	5.696684	11.8613
DN22959_c0_g1_i1_1	4.672055	2.426133
DN22962_c0_g1_i1_1	1.271365	1.086866
DN22965_c0_g1_i1_2	1.788632	1.339242
DN22966_c0_g1_i1_2	1.370618	1.877814
DN22968_c0_g1_i1_1	0	0
DN22977_c0_g1_i1_2	5.199474	6.879161
DN22978_c0_g1_i1_1	0.6321218	1.926926
DN2297_c0_g1_i1_1	0.575624	2.178809
DN22981_c0_g1_i1_1	0.7575821	1.307069
DN22982_c0_g1_i1_1	0.1818243	1.197187
DN22983_c0_g1_i1_1	1.804682	2.251424
DN22984_c0_g1_i1_1	0.77061	2.470186

DN22990_c0_g1_i1_1	4.223569	2.383255
DN22991_c0_g1_i1_1	7.821008	8.065029
DN22993_c0_g1_i1_1	0.2960226	4.53299
DN22995_c0_g1_i1_1	1.922419	1.764187
DN22997_c0_g1_i1_1	0	1.317159
DN22998_c0_g1_i1_1	5.530557	10.46469
DN22999_c0_g1_i1_1	0.7895708	1.751664
DN2299_c0_g1_i1_1	1.271766	2.320193
DN23001_c0_g1_i1_1	0.4578364	2.056646
DN23001_c0_g1_i1_2	2.028255	1.683287
DN23003_c0_g1_i1_2	3.505426	2.404289
DN23005_c0_g1_i1_1	0.7709273	1.612404
DN23008_c0_g1_i1_1	0.1331897	1.590378
DN23009_c0_g1_i1_1	0.575656	1.769196
DN2300_c0_g1_i1_2	0.6572962	1.090745
DN23012_c0_g1_i1_1	0.4253197	0.9216009
DN23013_c0_g1_i1_2	0.6866258	1.252839
DN23014_c0_g1_i1_2	0.2866128	0.6063711
DN23017_c0_g1_i1_1	0.707356	0.3699873
DN23018_c0_g1_i1_1	1.377487	2.040499
DN2301_c0_g1_i2_1	0.8365142	3.425452
DN23024_c0_g1_i1_1	0.547582	0.9070617
DN23025_c0_g1_i1_1	0.6223648	0.7433185
DN2302_c0_g1_i1_1	0.801435	1.189221

DN23038_c0_g1_i1_2	0	0
DN23040_c0_g1_i1_1	1.281767	3.736382
DN23043_c0_g1_i1_2	16.0548	21.99152
DN23045_c0_g1_i1_1	0.3963445	1.106401
DN23046_c0_g1_i1_1	0	0.478531
DN2304_c0_g1_i1_1	0.8176365	2.169734
DN23052_c0_g1_i1_1	2.117122	0.2324485
DN23056_c0_g1_i1_1	0.8705624	1.255089
DN23057_c0_g1_i1_1	2.172464	2.949641
DN23058_c0_g1_i1_1	0.4256705	0.536944
DN23061_c0_g1_i1_2	1.379013	1.079119
DN23063_c0_g1_i1_2	2.732049	4.294716
DN23065_c0_g1_i1_1	2.233529	1.350356
DN23066_c0_g1_i1_1	0.5260928	3.25862
DN23066_c0_g1_i1_2	1.039762	0.3298197
DN23067_c0_g1_i1_1	0.2510722	7.36698
DN23068_c0_g1_i1_2	1.750516	0.6804791
DN23075_c0_g1_i1_2	4.67134	6.247419
DN23077_c0_g1_i1_1	1.206857	0
DN23077_c0_g1_i1_2	1.570154	1.097786
DN2307_c0_g1_i1_1	2.72894	1.875581
DN2307_c0_g1_i1_2	1.018055	0.7194372
DN23083_c0_g1_i1_1	1.495447	2.231439
DN23088_c0_g1_i1_2	1.638195	0.4800631

DN23092_c0_g1_i1_1	2.929642	3.947251
DN23092_c0_g1_i1_2	0.6499931	0.1081876
DN23094_c0_g1_i1_1	0	0.5520073
DN23099_c0_g1_i1_2	51.91782	49.88166
DN2309_c0_g1_i2_1	5.26781	3.930465
DN23100_c0_g1_i1_1	1.175888	1.269916
DN23104_c0_g1_i1_2	1.465654	2.137987
DN23106_c0_g1_i1_1	0.5095079	0.9304607
DN23108_c0_g1_i1_2	5.720614	1.701275
DN23109_c0_g1_i1_1	0.7362764	8.154118
DN2310_c0_g1_i1_2	169.7873	0.1024928
DN23117_c0_g1_i1_1	0.5167866	3.84484
DN2311_c0_g1_i1_2	2.060215	0
DN23122_c0_g1_i1_2	0.6906962	0.3041184
DN23126_c0_g1_i1_1	0	1.447622
DN2312_c0_g1_i1_2	1.753215	1.791043
DN23131_c0_g1_i1_1	0.3414321	0.4219889
DN23131_c0_g1_i1_2	2.195338	0
DN23137_c0_g1_i1_1	0.7811754	1.142686
DN23140_c0_g1_i1_1	6.580423	2.85726
DN23141_c0_g1_i1_2	2.003377	0.3659314
DN23143_c0_g1_i1_1	3.290121	8.038642
DN23143_c0_g1_i1_2	5.096721	3.096108
DN23148_c0_g1_i1_1	0.9750461	1.008294

DN23149_c0_g1_i1_1	1.111575	1.005514
DN23150_c0_g1_i1_2	9.923294	3.655912
DN23155_c0_g1_i1_1	5.068665	1.486677
DN23155_c0_g1_i1_2	15.06543	16.33959
DN23157_c0_g1_i1_1	0.1908985	1.197864
DN23157_c0_g1_i1_2	0.1801441	0
DN23158_c0_g1_i1_1	0.2097566	2.750721
DN23159_c0_g1_i1_1	0.4647795	2.158268
DN23159_c0_g1_i1_2	379.8312	615.9543
DN23162_c0_g1_i1_1	0.9358384	2.38805
DN23164_c0_g1_i1_2	2.525591	0.9231212
DN23165_c0_g1_i1_2	9.701487	6.660077
DN23166_c0_g1_i1_2	0	0.7562354
DN23167_c0_g1_i1_1	1.47753	1.052571
DN23173_c0_g1_i1_2	1.0315	0.7172586
DN23183_c0_g1_i1_1	0.6740738	0.7221664
DN23186_c0_g1_i1_1	4.388987	4.62483
DN23186_c0_g1_i1_2	3.955069	0.6898944
DN23187_c0_g1_i1_1	1.000922	1.353689
DN23187_c0_g1_i1_2	1.073889	0.4818027
DN23188_c0_g1_i1_1	1.045071	3.77029
DN2318_c0_g1_i1_1	1.486503	1.661582
DN23191_c0_g1_i1_1	0.04014513	1.726398
DN23191_c0_g1_i1_2	8.242009	3.800433

DN23194_c0_g1_i1_2	0.8713324	1.038005
DN23196_c0_g1_i1_1	3.93589	13.00524
DN23196_c0_g1_i1_2	0.1786582	0
DN23197_c0_g1_i1_1	1.034297	0.7067482
DN2319_c0_g1_i1_1	0.9344536	0.9131223
DN23200_c0_g1_i1_2	1.137267	0.4257022
DN23201_c0_g1_i1_1	76.54098	40.54882
DN23202_c0_g1_i1_2	0.03319031	0.06921806
DN23205_c0_g1_i1_2	59.95081	44.4456
DN23206_c0_g1_i1_1	0.3071041	4.198786
DN2320_c0_g1_i1_1	2.064856	1.380182
DN23214_c0_g1_i1_1	0.6767454	0.2948334
DN23217_c0_g1_i1_1	1.382249	0.7848787
DN23222_c0_g1_i1_1	5.01342	1.316889
DN23224_c0_g1_i1_1	0.5927539	0.4996456
DN23226_c0_g1_i1_1	0.420343	0.2715013
DN2322_c0_g1_i1_1	0.3674142	0.6724526
DN23234_c0_g1_i1_2	2.873428	0.5109381
DN23236_c0_g1_i1_2	1.101389	1.358294
DN23237_c0_g1_i1_2	0	0
DN2323_c0_g1_i1_2	9.884103	5.656391
DN23240_c0_g1_i1_1	0.8894651	2.965247
DN23240_c0_g1_i1_2	0	0
DN23241_c0_g1_i1_1	0.8203948	2.431004

DN23253_c0_g1_i1_1	1.9998	3.031636
DN23256_c0_g1_i1_1	2.682471	1.532582
DN23257_c0_g1_i1_1	0.03590457	0
DN23262_c0_g1_i1_1	2.672229	2.746456
DN23267_c0_g1_i1_1	0.3660918	0.2132305
DN23268_c0_g1_i1_2	7.77714	11.10071
DN2326_c0_g2_i1_1	0.3437797	1.241216
DN23270_c0_g1_i1_2	19.03988	8.832198
DN23274_c0_g1_i1_1	0.9334257	1.956069
DN23276_c0_g1_i1_1	1.167396	2.994987
DN23285_c0_g1_i1_2	3.056998	2.702693
DN23286_c0_g1_i1_1	214.3564	289.2461
DN23290_c0_g1_i1_1	0	0.647858
DN23290_c0_g1_i1_2	0	0.8715497
DN23291_c0_g1_i1_1	0.414134	1.133286
DN23294_c0_g1_i1_1	0.08685363	0.9834717
DN23296_c0_g1_i1_1	0.3323821	0.3215301
DN2329_c0_g1_i1_2	5.462794	1.021034
DN23302_c0_g1_i1_2	3.546002	1.754652
DN23303_c0_g1_i1_1	1.633693	2.521797
DN23304_c0_g1_i1_1	3.296965	7.747191
DN23304_c0_g1_i1_2	4.540964	2.714871
DN23305_c0_g1_i1_1	1.682181	1.533736
DN23308_c0_g1_i1_1	0.1548347	4.107772

DN23318_c0_g1_i1_1	0.1873866	1.580905
DN23321_c0_g1_i1_1	0.09894744	0.5131992
DN23323_c0_g1_i1_1	1.247184	2.434697
DN23325_c0_g1_i1_1	1.42788	4.441652
DN2332_c0_g1_i1_1	5.206424	2.750508
DN23331_c0_g1_i1_1	0.4859188	0.3166669
DN23334_c0_g1_i1_1	10.90968	22.00201
DN23342_c0_g1_i1_1	0.845501	0.4070093
DN23344_c0_g1_i1_1	1.559457	2.046379
DN23344_c0_g1_i1_2	1.046668	0.6248942
DN23345_c0_g1_i1_1	0.4659305	1.814502
DN23349_c0_g1_i1_1	0.3692437	3.837504
DN23354_c0_g1_i1_1	0.2606796	0.3539843
DN23355_c0_g1_i1_2	218.6878	124.4807
DN23356_c0_g1_i1_1	60.8898	47.96483
DN23360_c0_g1_i1_2	2.426835	2.380295
DN23361_c0_g1_i1_1	7.785436	5.331583
DN23362_c0_g1_i1_1	0	2.39501
DN23362_c0_g1_i1_2	4.946376	1.602577
DN23365_c0_g1_i1_1	4.8583	1.7641
DN23368_c0_g1_i1_2	3.050582	4.485759
DN23372_c0_g1_i1_1	1.139232	0
DN23375_c0_g1_i1_1	0.8936298	1.64698
DN23377_c0_g1_i1_1	0.2912937	1.934558

DN23381_c0_g1_i1_1	2.157204	1.746194
DN23382_c0_g1_i1_2	0.6254647	0.4992129
DN23384_c0_g1_i1_1	0.3859644	1.360381
DN23385_c0_g1_i1_1	1.266533	1.518749
DN23387_c0_g1_i1_1	2.776435	2.401628
DN23387_c0_g1_i1_2	2.20013	0
DN23389_c0_g1_i1_1	1.511539	0
DN23389_c0_g1_i1_2	2.385999	2.482316
DN2338_c0_g1_i1_1	0	0.3317708
DN23391_c0_g1_i1_2	3.358279	0
DN23394_c0_g1_i1_1	0.2774038	0.2378664
DN23395_c0_g1_i1_1	1.698316	2.193264
DN2339_c0_g1_i1_1	0.2818462	10.38416
DN23400_c0_g1_i1_2	3.767728	1.408331
DN23401_c0_g1_i1_2	1.215472	0.6214925
DN23402_c0_g1_i1_1	0.1257695	0.3279041
DN23405_c0_g1_i1_1	4.107783	2.986242
DN23406_c0_g1_i1_2	2.078994	0
DN23408_c0_g1_i1_1	1.590698	2.676556
DN23409_c0_g1_i1_1	0.5717986	1.682795
DN2340_c0_g1_i1_1	1.510983	2.284277
DN23411_c0_g1_i1_2	1.89093	2.12839
DN23414_c0_g1_i1_1	1.589559	1.025846
DN23416_c0_g1_i1_1	8.111352	10.06745

DN23419_c0_g1_i1_2	0	0
DN23420_c0_g1_i1_1	1.965376	2.018486
DN23422_c0_g1_i1_1	0.9752462	0.5349216
DN23423_c0_g1_i1_2	3.757493	0.3395864
DN23425_c0_g1_i1_1	18.70102	16.11604
DN23426_c0_g1_i1_2	1.259948	0
DN23427_c0_g1_i1_2	0	0
DN23429_c0_g1_i1_2	8.079346	7.118251
DN23433_c0_g1_i1_1	0.2348379	0.9486949
DN23435_c0_g1_i1_2	7.786358	0.7943763
DN23437_c0_g1_i1_1	76.40145	30.49713
DN23438_c0_g1_i1_1	12.86957	10.59763
DN23439_c0_g1_i1_1	0.7883411	10.45041
DN23440_c0_g1_i1_1	0	0.6760101
DN23440_c0_g1_i1_2	2.436152	0.2265885
DN23441_c0_g1_i1_1	12.17972	16.31345
DN23442_c0_g1_i1_2	1.267067	0.4063256
DN23443_c0_g1_i1_1	7.431088	8.730973
DN23444_c0_g1_i1_1	0	3.62265
DN23447_c0_g1_i1_2	2.239241	0
DN23452_c0_g1_i1_2	20.07442	18.4058
DN23458_c0_g1_i1_1	0	0.5598296
DN23459_c0_g1_i1_1	0.5404055	5.172012
DN23464_c0_g1_i1_1	0.4606666	1.965486

DN23465_c0_g1_i1_2	15.23434	23.33023
DN23467_c0_g1_i1_2	1.273469	0.5119427
DN23470_c0_g1_i1_1	1.283523	1.848772
DN23471_c0_g1_i1_1	6.102891	6.350292
DN23472_c0_g1_i1_1	0	1.448692
DN23474_c0_g1_i1_1	21.41289	19.69524
DN23474_c0_g1_i1_2	1.545223	0.4692572
DN23477_c0_g1_i1_2	0.4892621	1.372495
DN23478_c0_g1_i1_2	2.520311	2.896692
DN23480_c0_g1_i1_1	6.314678	9.163572
DN23482_c0_g1_i1_1	1.764989	7.792077
DN23482_c0_g1_i1_2	0.5512755	0.1456225
DN23484_c0_g1_i1_2	1.712889	1.462961
DN23488_c0_g1_i1_1	0.5115553	1.246639
DN23489_c0_g1_i1_1	1.991748	5.774559
DN23489_c0_g1_i1_2	0.8942371	0.3088716
DN2348_c0_g1_i1_1	0	0
DN23492_c0_g1_i1_1	0.4953199	0.9157027
DN23493_c0_g1_i1_2	0.6057562	0.7913771
DN23494_c0_g1_i1_1	1.126832	1.134013
DN23497_c0_g1_i1_1	6.327576	2.445369
DN23500_c0_g1_i1_1	1.238832	2.787432
DN23501_c0_g1_i1_1	4.855796	5.696941
DN23502_c0_g1_i1_2	2.559875	2.549827

DN23504_c0_g1_i1_2	2.532313	0
DN23506_c0_g1_i1_1	0	1.735582
DN23509_c0_g1_i1_2	0.9746467	0.8465668
DN2350_c0_g1_i1_1	1.002718	2.680527
DN23510_c0_g1_i1_1	1.96878	0.3800719
DN23512_c0_g1_i1_1	2.124209	2.842253
DN23513_c0_g1_i1_1	6.333372	6.913351
DN23514_c0_g1_i1_1	0.9442482	0.4430023
DN23515_c0_g1_i1_1	1.077565	1.454956
DN23515_c0_g1_i1_2	3.722015	1.818779
DN23519_c0_g1_i1_1	1.250956	1.42E-30
DN23521_c0_g1_i1_1	3.269545	2.428374
DN23521_c0_g1_i1_2	3.929109	2.666239
DN23522_c0_g1_i1_1	3.376132	0.9184617
DN23526_c0_g1_i1_1	0.04542316	2.268089
DN2352_c0_g1_i1_1	0.1386405	5.25E-24
DN23530_c0_g1_i1_1	9.115278	3.909895
DN23531_c0_g1_i1_1	82.73048	80.94057
DN23537_c0_g1_i1_1	0.105401	1.137804
DN23542_c0_g1_i1_2	2.769663	1.451032
DN23543_c0_g1_i1_1	1.445082	1.905876
DN23545_c0_g1_i1_1	3.313481	1.445708
DN23546_c0_g1_i1_1	26.22339	39.76927
DN23547_c0_g1_i1_1	0.2012585	1.897732

DN23547_c0_g1_i1_2	3.052809	0.7514448
DN23549_c0_g1_i1_2	1.014532	0.9752552
DN2354_c0_g1_i1_2	0.5557444	0.1873848
DN23551_c0_g1_i1_1	0.2794653	1.014431
DN23554_c0_g1_i1_1	0.7203218	0.4797487
DN23557_c0_g1_i1_1	0.2777531	3.609401
DN23558_c0_g1_i1_2	3.020801	2.613023
DN23560_c0_g1_i1_1	1.126375	7.798953
DN23563_c0_g1_i1_2	1.412637	0.4450689
DN23564_c0_g1_i1_2	7.073647	1.793021
DN23565_c0_g1_i1_1	1.870757	1.176067
DN23565_c0_g1_i1_2	7.845174	5.0979
DN23566_c0_g1_i1_2	0.1064765	0.06147751
DN23567_c0_g1_i1_1	0	0
DN23568_c0_g1_i1_1	17.92595	87.46295
DN23569_c0_g1_i1_1	0.8685128	1.806909
DN2356_c0_g2_i1_1	1.367962	53.3789
DN23572_c0_g1_i1_1	0	0.224843
DN23573_c0_g1_i1_1	0.248859	2.690534
DN23576_c0_g1_i1_2	1.425789	0.2373321
DN23579_c0_g1_i1_2	3.891419	2.751884
DN23580_c0_g1_i1_1	2.041423	1.340253
DN23580_c0_g1_i1_2	42.2659	33.41392
DN23581_c0_g1_i1_1	3.495976	4.067191

DN23582_c0_g1_i1_1	3.591577	3.245416
DN23584_c0_g1_i1_1	1.435747	1.669459
DN23585_c0_g1_i1_1	2.041153	1.993258
DN23585_c0_g1_i1_2	11.33449	4.398927
DN23590_c0_g1_i1_1	1.865554	0.2469768
DN23590_c0_g1_i1_2	57.10877	14.76511
DN23598_c0_g1_i1_1	1.335429	1.158387
DN23598_c0_g1_i1_2	3.983411	0.6520097
DN23599_c0_g1_i1_1	1.334707	0.2925057
DN2359_c0_g1_i1_1	0.9893992	0.6904802
DN235_c0_g1_i1_1	1.694023	1.595536
DN23600_c0_g1_i1_1	1.538012	0.5527807
DN23605_c0_g1_i1_1	1.19946	1.589495
DN23605_c0_g1_i1_2	1.629695	0.4899645
DN23607_c0_g1_i1_1	0.4347319	0.695163
DN23607_c0_g1_i1_2	0	0
DN23610_c0_g1_i1_1	0.5509423	4.575613
DN23613_c0_g1_i1_2	0.2270023	0
DN23614_c0_g1_i1_1	3.883748	9.744914
DN23614_c0_g1_i1_2	10.29719	5.078281
DN23615_c0_g1_i1_1	27.63606	24.17731
DN23624_c0_g1_i1_1	1.402249	2.70819
DN23625_c0_g1_i1_1	3.682044	2.636351
DN23628_c0_g1_i1_2	0.6184546	0.07033708

DN23630_c0_g1_i1_2	2.470808	4.362267
DN23634_c0_g1_i1_2	0.4130151	1.140662
DN23637_c0_g1_i1_1	1.216384	3.695112
DN23639_c0_g1_i1_1	40.64558	16.06933
DN23639_c0_g1_i1_2	1.600051	0.707912
DN23641_c0_g1_i1_2	0.9132039	0.4052195
DN23642_c0_g1_i1_1	0.6691587	3.257808
DN23644_c0_g1_i1_1	7.624796	9.196586
DN23645_c0_g1_i1_2	3.450875	1.49576
DN23651_c0_g1_i1_1	13.33289	6.30374
DN23651_c0_g1_i1_2	2.072041	0.4000068
DN23655_c0_g1_i1_2	0.2626227	0
DN23656_c0_g1_i1_2	0.6275394	0
DN23658_c0_g1_i1_2	2.834547	0.2890668
DN23659_c0_g1_i1_1	0	0.935584
DN23660_c0_g1_i1_1	0.5338717	1.941071
DN23661_c0_g1_i1_1	1.210032	0
DN23662_c0_g1_i1_1	14.46291	6.035134
DN23664_c0_g1_i1_1	0.3826909	1.996126
DN23667_c0_g1_i1_1	1.058365	1.487891
DN23669_c0_g1_i1_1	2.8954	2.135559
DN23669_c0_g1_i1_2	2.363009	0.1646906
DN23675_c0_g1_i1_1	1.65234	0.6365275
DN23679_c0_g1_i1_1	0.6357883	2.236902

DN23679_c0_g1_i1_2	7.128645	1.284825
DN23682_c0_g1_i1_1	0.5811739	0
DN23683_c0_g1_i1_1	4.661664	5.38034
DN23688_c0_g1_i1_2	28.68416	24.08097
DN23689_c0_g1_i1_1	0.9761252	1.23063
DN23689_c0_g1_i1_2	0.3353851	0
DN2368_c0_g1_i1_1	1.088192	1.364804
DN23690_c0_g1_i1_2	3.729416	2.309445
DN23694_c0_g1_i1_1	0.4168279	1.946777
DN23694_c0_g1_i1_2	1.700217	0
DN23697_c0_g1_i1_1	0.1288937	0.5547579
DN23697_c0_g1_i1_2	2.088933	1.03E-28
DN23698_c0_g1_i1_1	1.460385	0.8140771
DN23699_c0_g1_i1_2	22.48138	15.63682
DN23701_c0_g1_i1_2	0.4277482	0.4258436
DN23703_c0_g1_i1_1	0.7055907	0.4431374
DN23704_c0_g1_i1_1	0.4039818	1.085333
DN23708_c0_g1_i1_1	0.4750194	0.5703618
DN23709_c0_g1_i1_2	1.987958	0
DN23712_c0_g1_i1_1	0	1.143177
DN23718_c0_g1_i1_1	1.583448	1.195912
DN23720_c0_g1_i1_1	0	1.645279
DN23723_c0_g1_i1_1	0.7753935	2.656492
DN23723_c0_g1_i1_2	0.4500794	0.5410432

DN23726_c0_g1_i1_1	0.5744654	0.5736892
DN23728_c0_g1_i1_1	36.89153	20.56433
DN23729_c0_g1_i1_1	0.2996618	0.7420799
DN23730_c0_g1_i1_1	0	4.127158
DN23730_c0_g1_i1_2	1.729188	0.7818814
DN23731_c0_g1_i1_2	4.014409	1.207403
DN23733_c0_g1_i1_2	1.382149	0.2693613
DN23736_c0_g1_i1_1	0.5902369	0.2748147
DN23738_c0_g1_i1_1	0.5577279	0
DN23738_c0_g1_i1_2	1.707221	3.22289
DN23740_c0_g1_i1_2	0	0
DN23741_c0_g1_i1_1	0.4860348	1.924585
DN23742_c0_g1_i1_2	1.45721	0.5785451
DN23746_c0_g1_i1_1	0.1328119	1.280842
DN23750_c0_g1_i1_1	4.540972	8.438849
DN23754_c0_g1_i1_1	3.338127	1.872869
DN23756_c0_g1_i1_1	11.98551	31.1582
DN23756_c0_g1_i1_2	0.700033	0.4108488
DN23757_c0_g1_i1_1	0.8891305	0
DN23760_c0_g1_i1_2	2.880138	0
DN23762_c0_g1_i1_2	2.839094	0.3795732
DN23764_c0_g1_i1_2	1.002289	0.8648931
DN23765_c0_g1_i1_1	2.004562	1.130106
DN23766_c0_g1_i1_1	6.764663	7.993052

DN23767_c0_g1_i1_1	2.210577	1.082436
DN23768_c0_g1_i1_2	6.503984	3.530962
DN23769_c0_g1_i1_2	2.514161	0.8589357
DN23772_c0_g1_i1_1	1.745087	3.21907
DN23773_c0_g1_i1_1	0.7707881	2.809505
DN23774_c0_g1_i1_2	2.22178	1.344664
DN23775_c0_g1_i1_1	0	2.054236
DN23776_c0_g1_i1_1	0	4.318812
DN23777_c0_g1_i1_2	42.04382	27.21767
DN23780_c0_g1_i1_1	0.4642734	0.5125952
DN23781_c0_g1_i1_1	0.7329054	1.715508
DN23781_c0_g1_i1_2	1.103187	0
DN23782_c0_g1_i1_1	0	1.35538
DN23784_c0_g1_i1_2	2.294057	0.4186191
DN23785_c0_g1_i1_2	2.249414	0.6797222
DN23788_c0_g1_i1_1	0.5909687	2.834999
DN23789_c0_g1_i1_1	7.98373	13.49834
DN23794_c0_g1_i1_1	0	0
DN23795_c0_g1_i1_1	1.60556	0.3352139
DN23796_c0_g1_i1_1	1.554055	2.997357
DN23797_c0_g1_i1_1	2.142215	3.546869
DN23802_c0_g1_i1_1	0.2786163	0.2982634
DN23802_c0_g1_i1_2	1.088729	0.5362159
DN23804_c0_g1_i1_2	1.850034	0.9622969

DN23805_c0_g1_i1_2	0	0
DN23813_c0_g1_i1_2	86.63203	74.56569
DN23816_c0_g1_i1_1	3.704218	6.919022
DN23820_c0_g1_i1_1	5.644932	6.19802
DN23822_c0_g1_i1_2	1.912575	0.2769772
DN23826_c0_g1_i1_1	3.408506	2.745813
DN23827_c0_g1_i1_1	0.4644152	1.443601
DN23827_c0_g1_i1_2	2.005373	0.6188682
DN23828_c0_g1_i1_2	1.605405	0.2984585
DN2382_c0_g1_i1_1	0.5211176	1.6897
DN23830_c0_g1_i1_1	0.9724218	1.249752
DN23837_c0_g1_i1_1	3.442439	4.064342
DN23839_c0_g1_i1_1	0	1.219991
DN23840_c0_g1_i1_2	1.939128	0
DN23843_c0_g1_i1_1	1.429568	2.63726
DN23843_c0_g1_i1_2	19.49691	14.81505
DN23846_c0_g1_i1_2	2.713997	0.5141018
DN23848_c0_g1_i1_1	2.108079	1.718868
DN2384_c0_g1_i2_1	3.489397	2.296796
DN23851_c0_g1_i1_2	0.3957798	0
DN23852_c0_g1_i1_2	2.048599	1.289988
DN23853_c0_g1_i1_1	4.328768	6.920645
DN23855_c0_g1_i1_1	0.3840555	0
DN23858_c0_g1_i1_1	4.199159	5.343642

DN23862_c0_g1_i1_1	0.2897647	0.8678295
DN23866_c0_g1_i1_2	2.739546	0
DN23867_c0_g1_i1_1	1.181902	0.968907
DN23868_c0_g1_i1_1	0.7651182	1.302653
DN23871_c0_g1_i1_1	1.648057	4.735519
DN23873_c0_g1_i1_1	0.2215285	1.112644
DN23874_c0_g1_i1_1	0	0
DN23875_c0_g1_i1_1	0	0
DN23876_c0_g1_i1_2	1.683895	1.152693
DN23879_c0_g1_i1_2	3.850818	0.6021983
DN23880_c0_g1_i1_1	1.357179	4.07E-15
DN23881_c0_g1_i1_1	162.2168	66.00094
DN23888_c0_g1_i1_2	0	0.1702954
DN23891_c0_g1_i1_2	6.3719	2.738487
DN23892_c0_g1_i1_1	3.028573	3.47182
DN23896_c0_g1_i1_2	0.7002765	0
DN23897_c0_g1_i1_1	0	0.6554212
DN23897_c0_g1_i1_2	5.81219	9.785433
DN23899_c0_g1_i1_1	0	1.713691
DN23900_c0_g1_i1_2	1.394004	1.041732
DN23902_c0_g1_i1_1	0	0.7865555
DN23906_c0_g1_i1_1	0.1253103	0.376741
DN23906_c0_g1_i1_2	2.275452	0.4788522
DN23908_c0_g1_i1_2	1.818114	2.18731

DN23909_c0_g1_i1_1	1.856995	1.986591
DN23910_c0_g1_i1_1	0.5498343	0
DN23910_c0_g1_i1_2	1.777319	0.3336169
DN23911_c0_g1_i1_1	0	1.950278
DN23911_c0_g1_i1_2	2.800115	0.6839841
DN23912_c0_g1_i1_1	0.652577	0
DN23912_c0_g1_i1_2	0.1977807	0.07227106
DN23913_c0_g1_i1_2	2.980471	2.152398
DN23917_c0_g1_i1_1	1.162413	0.8292408
DN23918_c0_g1_i1_1	0	1.78243
DN23922_c0_g1_i1_1	0.2979985	0
DN23924_c0_g1_i1_1	0.2929094	0.2896276
DN23925_c0_g1_i1_2	2.702297	1.915304
DN23927_c0_g1_i1_1	4.303009	6.168913
DN23931_c0_g1_i1_1	0.7916604	4.816895
DN23932_c0_g1_i1_2	2.726571	0.2464965
DN23945_c0_g1_i1_2	6.816232	3.357805
DN23948_c0_g1_i1_1	0.7684496	0.9775761
DN23948_c0_g1_i1_2	1.639649	2.059542
DN23965_c0_g1_i1_1	0.3260541	4.032707
DN23969_c0_g1_i1_2	2.773269	1.779399
DN23973_c0_g1_i1_1	0.7102476	1.176692
DN23977_c0_g1_i1_2	3.266308	1.462658
DN23980_c0_g1_i1_1	26.22993	8.22785

DN23982_c0_g1_i1_1	5.510108	12.64859
DN23982_c0_g1_i1_2	72.33953	55.01703
DN23988_c0_g1_i1_2	3.899887	2.830631
DN23990_c0_g1_i1_2	30.89618	10.03068
DN23991_c0_g1_i1_1	0.2943989	1.229296
DN23992_c0_g1_i1_2	1.232778	0.9578366
DN23994_c0_g1_i1_1	0.4268501	16.70191
DN23996_c0_g1_i1_1	0.3969533	0.4855592
DN23999_c0_g1_i1_1	21.75005	14.10788
DN239_c0_g1_i1_1	0	1.449025
DN24010_c0_g1_i1_1	0.4705489	1.257001
DN24013_c0_g1_i1_2	1.299689	0
DN24014_c0_g1_i1_1	0.3488219	3.581049
DN24017_c0_g1_i1_2	0.8165623	0.3074463
DN24018_c0_g1_i1_1	1.166949	0.9467412
DN24022_c0_g1_i1_1	0.4797475	0.8771213
DN24023_c0_g1_i1_1	0.7991989	0.7942155
DN24035_c0_g1_i1_2	4.220423	10.42497
DN24043_c0_g1_i1_1	1.103946	3.65322
DN24046_c0_g1_i1_1	36.74992	15.92008
DN24055_c0_g1_i1_1	3.364118	3.528624
DN24055_c0_g1_i1_2	1.644161	3.31E-28
DN24056_c0_g1_i1_2	0.5196198	0.1968619
DN24057_c0_g1_i1_2	2.533992	1.151492

DN24058_c0_g1_i1_1	0	7.574322
DN24060_c0_g1_i1_1	2.140634	3.913079
DN24062_c0_g1_i1_1	1.189652	0.8033917
DN24066_c0_g1_i1_1	54.36737	34.38816
DN24072_c0_g1_i1_2	0.4450909	0.1929552
DN24076_c0_g1_i1_1	2.55961	1.19813
DN24078_c0_g1_i1_1	1.722864	2.510989
DN24078_c0_g1_i1_2	1.230692	0.4328289
DN24085_c0_g1_i1_1	0.7398861	0.9002849
DN24087_c0_g1_i1_2	21.52023	18.74462
DN24091_c0_g1_i1_1	2.23517	4.858494
DN24094_c0_g1_i1_2	4.499679	1.437282
DN24099_c0_g1_i1_2	248.7948	0.166243
DN24115_c0_g1_i1_1	2.035379	8.071865
DN24121_c0_g1_i1_1	4.534218	2.589861
DN24122_c0_g1_i1_1	4.979481	7.241208
DN24123_c0_g1_i1_1	0.3959985	0
DN24123_c0_g1_i1_2	5.841972	2.462114
DN24124_c0_g1_i1_2	1.921783	0.4734379
DN24128_c0_g1_i1_1	0.6750501	1.410171
DN24129_c0_g1_i1_2	0.290123	1.237774
DN24136_c0_g1_i1_1	0.5922623	1.169814
DN24143_c0_g1_i1_1	12.72403	6.378729
DN24144_c0_g1_i1_1	0.2835876	1.683577

DN24145_c0_g1_i1_1	0.6404998	0.2500147
DN24146_c0_g1_i1_2	9.222314	15.67187
DN24147_c0_g1_i1_1	1.269019	2.787989
DN24149_c0_g1_i1_1	8.562356	21.19299
DN24156_c0_g1_i1_1	0.07411551	0.7495216
DN24157_c0_g1_i1_1	1.371006	1.959087
DN24158_c0_g1_i1_1	2.466976	3.752917
DN24167_c0_g1_i1_2	0.7490703	1.140913
DN24173_c0_g1_i1_1	7.984951	7.747688
DN24179_c0_g1_i1_2	1.825619	1.806999
DN24180_c0_g1_i1_2	0.9553982	0.7153418
DN24188_c0_g1_i1_2	75.69667	13.88342
DN24192_c0_g1_i1_1	4.574852	4.140922
DN24200_c0_g1_i1_1	2.268737	23.6931
DN24201_c0_g1_i1_2	15.60042	80.10131
DN24202_c0_g1_i1_1	19.20938	18.65977
DN24204_c0_g1_i1_1	0.2975214	5.294085
DN24208_c0_g1_i1_2	36.44981	23.81442
DN24210_c0_g1_i1_2	0.7340172	1.690154
DN24215_c0_g1_i1_2	6.220698	3.41933
DN24218_c0_g1_i1_2	1.806069	4.07227
DN24231_c0_g1_i1_1	3.542751	1.496784
DN24232_c0_g1_i1_1	1.184333	2.585074
DN24240_c0_g1_i1_1	2.491565	2.696229

DN24240_c0_g1_i1_2	0.8571278	0
DN24243_c0_g1_i1_1	2.151646	6.619535
DN24247_c0_g1_i1_1	1.152216	7.685108
DN24254_c0_g1_i1_2	4.984033	4.063243
DN2425_c0_g1_i1_1	15.50574	23.87028
DN24260_c0_g1_i1_2	1.395883	0.2401246
DN24261_c0_g1_i1_1	4.271934	5.32548
DN24263_c0_g1_i1_2	17.578	14.04907
DN2426_c0_g1_i1_1	0.8437675	1.800554
DN24276_c0_g1_i1_1	5.695407	2.408164
DN24277_c0_g1_i1_1	0.2400446	2.222383
DN24279_c0_g1_i1_2	0.7586618	0.2261068
DN2427_c0_g1_i1_1	2.529655	0.5372787
DN24280_c0_g1_i1_1	0	0.2362805
DN24281_c0_g1_i1_1	0.9157035	1.327721
DN24286_c0_g1_i1_1	1.639896	0.7996114
DN2428_c0_g1_i1_2	4.718893	5.548112
DN24293_c0_g1_i1_2	6.403304	3.144357
DN24296_c0_g1_i1_1	5.15753	5.772923
DN24296_c0_g1_i1_2	2.566677	1.582607
DN24299_c0_g1_i1_2	2.571782	1.478481
DN242_c0_g1_i1_2	2.122468	2.905029
DN24302_c0_g1_i1_1	0	0
DN24303_c0_g1_i1_1	0.1349544	1.682788

DN24312_c0_g1_i1_1	1.797488	1.304203
DN24326_c0_g1_i1_1	1.786289	4.7155
DN24327_c0_g1_i1_2	18.87779	13.58297
DN24328_c0_g1_i1_2	10.52137	14.12154
DN24329_c0_g1_i1_1	30.01071	27.26315
DN24332_c0_g1_i1_1	37.12641	19.60522
DN24333_c0_g1_i1_1	3.968279	8.332995
DN24335_c0_g1_i1_2	2.635112	0.6608037
DN24338_c0_g1_i1_1	4.653453	23.20204
DN24340_c0_g1_i1_1	2.381187	14.98438
DN24342_c0_g1_i1_1	1.281769	4.920954
DN24342_c0_g1_i1_2	27.74759	15.77862
DN24345_c0_g1_i1_2	1.78022	0.6832344
DN24346_c0_g1_i1_1	2.829232	3.973414
DN24347_c0_g1_i1_1	1.589941	2.02892
DN24354_c0_g1_i1_1	3.367557	1.595523
DN24356_c0_g1_i1_1	0	1.308262
DN24359_c0_g1_i1_1	0.4578244	1.812937
DN24363_c0_g1_i1_1	1.523114	1.42123
DN24363_c0_g1_i1_2	17.60339	34.18558
DN24364_c0_g1_i1_1	4.07895	4.272754
DN24364_c0_g1_i1_2	2.639212	0.7301385
DN24369_c0_g1_i1_1	0.4016346	0.6575519
DN24373_c0_g1_i1_1	1.906307	5.965194

DN24376_c0_g1_i1_1	0.5609983	1.038237
DN24376_c0_g1_i1_2	2.439128	1.620892
DN24379_c0_g1_i1_1	0.5978111	0.1573429
DN24381_c0_g1_i1_2	0.5564727	0.2683758
DN24388_c0_g1_i1_2	0.5626915	0
DN2438_c0_g1_i2_2	5.148135	2.834237
DN2439_c0_g1_i1_1	0.1721835	0.9676877
DN243_c0_g1_i1_1	3.846371	3.694276
DN243_c0_g1_i1_2	1.141465	0.4267453
DN24402_c0_g1_i1_2	1.650164	0.3251032
DN24406_c0_g1_i1_1	15.44858	5.341356
DN2440_c0_g1_i1_1	0.5580143	0.9214222
DN24410_c0_g1_i1_1	2.916502	3.859231
DN24411_c0_g1_i1_2	0.7656748	0.4250996
DN24416_c0_g1_i1_1	0.06225142	0.1493605
DN24420_c0_g1_i1_1	1.410219	1.238795
DN24421_c0_g1_i1_1	1.773217	2.425033
DN24424_c0_g1_i1_1	1.189968	10.40488
DN24427_c0_g1_i1_1	1.474633	1.358218
DN24429_c0_g1_i1_1	0.4011004	0.5160742
DN2442_c0_g1_i1_1	0.2143786	0.4963325
DN24430_c0_g1_i1_1	0.3148826	2.958263
DN24431_c0_g1_i1_1	70.28222	43.58616
DN24434_c0_g1_i1_1	2.519553	2.341303

DN2443_c0_g1_i1_1	2.954301	2.618046
DN24447_c0_g1_i1_1	0.6101465	1.131681
DN24450_c0_g1_i1_2	0.9602309	0.9355104
DN24455_c0_g1_i1_1	3.24991	6.883812
DN24457_c0_g1_i1_1	0.8611208	0.9367424
DN24460_c0_g1_i1_1	0.5439388	4.744925
DN2446_c0_g1_i1_1	6.799148	2.39952
DN24474_c0_g1_i1_1	0.3581714	1.572498
DN24474_c0_g1_i1_2	2.750492	0.7927052
DN24476_c0_g1_i1_2	1.484412	0
DN2447_c0_g1_i1_1	0.9266033	0.846755
DN24481_c0_g1_i1_1	1.256058	2.293076
DN24483_c0_g1_i1_1	6.957602	2.681759
DN24486_c0_g1_i1_1	0.3104446	29.27521
DN24495_c0_g1_i1_1	0.965322	1.98785
DN24496_c0_g1_i1_2	2.165725	1.431449
DN24499_c0_g1_i1_2	3.635813	0.6640545
DN24503_c0_g1_i1_1	1.481501	0.6130716
DN24509_c0_g1_i1_2	1.574691	1.32953
DN2450_c0_g1_i1_2	7.650659	10.33513
DN24513_c0_g1_i1_1	1.056856	1.37182
DN24514_c0_g1_i1_1	0	0
DN24515_c0_g1_i1_1	0.24711	0.8940129
DN24521_c0_g1_i1_2	1.852654	2.063192

DN24522_c0_g1_i1_1	13.54214	13.30091
DN24523_c0_g1_i1_2	4.061759	2.786123
DN24527_c0_g1_i1_2	5.305999	0.4654726
DN24529_c0_g1_i1_1	0.8881988	14.39039
DN24531_c0_g1_i1_2	8.884166	2.740342
DN24537_c0_g1_i1_1	1.128473	0.7613925
DN24539_c0_g1_i1_1	0.02094975	0.9760507
DN24541_c0_g1_i1_1	1.451663	3.90957
DN24544_c0_g1_i1_1	0.8942422	0.8209657
DN24545_c0_g1_i1_1	1.00592	1.273509
DN24547_c0_g1_i1_1	1.626789	3.32084
DN24550_c0_g1_i1_1	0.6749742	1.84E-20
DN24551_c0_g1_i1_1	44.45058	42.30644
DN24553_c0_g1_i1_1	0.6330245	0.5674067
DN24554_c0_g1_i1_1	0	1.080552
DN24558_c0_g1_i1_2	3.576745	0.5569757
DN24560_c0_g1_i1_2	3.404928	7.466667
DN24561_c0_g1_i1_2	0.5463716	0.1269182
DN24562_c0_g1_i1_1	1.772503	8.123724
DN24564_c0_g1_i1_1	1.192132	1.225339
DN24567_c0_g1_i1_1	8.440751	4.832453
DN24572_c0_g1_i1_1	3.87208	4.401116
DN24573_c0_g1_i1_2	61.51292	25.91825
DN24580_c0_g1_i1_1	0.7338123	2.786578

DN24581_c0_g1_i1_1	6.449398	3.74532
DN24586_c0_g1_i1_1	8.910983	9.996083
DN24590_c0_g1_i1_1	0.8403936	0.6085682
DN24591_c0_g1_i1_2	1.042273	0.6888735
DN24592_c0_g1_i1_1	0	0.7208355
DN24595_c0_g1_i1_1	0.4092749	3.69899
DN24599_c0_g1_i1_1	1.608233	1.104754
DN24603_c0_g1_i1_1	0.2207645	1.590407
DN24604_c0_g1_i1_1	4.253031	4.322864
DN24610_c0_g1_i1_2	4.089101	2.496293
DN24613_c0_g1_i1_1	1.801145	1.134699
DN24618_c0_g1_i1_1	0.2628584	4.380761
DN24621_c0_g1_i1_1	1.37963	1.224842
DN24622_c0_g1_i1_2	2.381579	1.834776
DN24624_c0_g1_i1_1	0	0.4226556
DN24631_c0_g1_i1_1	2.211465	5.823716
DN24633_c0_g1_i1_1	0	0
DN24634_c0_g1_i1_2	2.939627	0.7552915
DN24637_c0_g1_i1_1	4.133595	3.339748
DN24638_c0_g1_i1_2	1.204917	0.5529767
DN24640_c0_g1_i1_1	0.1869553	1.624909
DN24641_c0_g1_i1_2	6.834314	0.8700416
DN24642_c0_g1_i1_2	14.02219	1.093883
DN24644_c0_g1_i1_1	2.385548	1.029583

DN24646_c0_g1_i1_2	23.413	5.879326
DN24652_c0_g1_i1_1	0.8577428	1.903192
DN24653_c0_g1_i1_2	0.3240026	0.2367719
DN24666_c0_g1_i1_1	3.119987	1.361733
DN24666_c0_g1_i1_2	13.35507	7.251994
DN24669_c0_g1_i1_1	0.3524658	0.3267672
DN24669_c0_g1_i1_2	5.032076	5.907219
DN24670_c0_g1_i1_1	0.6745143	2.235045
DN24671_c0_g1_i1_2	0	0
DN24679_c0_g1_i1_2	3.354965	1.354688
DN2467_c0_g1_i1_2	36.25316	16.32518
DN24681_c0_g1_i1_1	3.525754	10.46566
DN24683_c0_g1_i1_1	8.950033	12.60714
DN24684_c0_g1_i1_1	0.8562608	1.705824
DN24684_c0_g1_i1_2	0.7999271	0.6207493
DN24685_c0_g1_i1_1	2.847947	3.945393
DN24686_c0_g1_i1_1	2.164294	6.935404
DN24687_c0_g1_i1_1	2.056783	2.323359
DN24688_c0_g1_i1_1	0.8539034	2.130366
DN24692_c0_g1_i1_1	0.8283041	1.546772
DN24698_c0_g1_i1_1	0.6737979	0.3027198
DN24704_c0_g1_i1_1	0.5198178	3.591595
DN24705_c0_g1_i1_1	1.46015	3.691949
DN24708_c0_g1_i1_1	161.573	386.5744

DN2470_c0_g1_i1_2	3.025878	8.065937
DN24711_c0_g1_i1_2	22.58463	6.82973
DN24714_c0_g1_i1_1	0.6442323	0.08999605
DN24714_c0_g1_i1_2	0	0
DN24717_c0_g1_i1_2	2.713859	1.182814
DN24720_c0_g1_i1_1	32.34192	28.69855
DN24720_c0_g1_i1_2	2.37997	3.135787
DN24722_c0_g1_i1_1	0.523446	1.399112
DN24725_c0_g1_i1_1	0.2799157	1.209617
DN24725_c0_g1_i1_2	1.781163	0.2751413
DN24726_c0_g1_i1_1	5.256491	2.145329
DN24729_c0_g1_i1_2	0.1365078	0.1188775
DN24730_c0_g1_i1_1	1.477323	5.869302
DN24732_c0_g1_i1_2	8.541082	24.2417
DN24738_c0_g1_i1_1	1.969917	3.134383
DN24743_c0_g1_i1_1	0.3690673	3.026645
DN24743_c0_g1_i1_2	1.310073	2.230361
DN24744_c0_g1_i1_2	0.9536104	0.3026353
DN24745_c0_g1_i1_1	0.9189495	1.507705
DN24752_c0_g1_i1_1	0.2586486	1.090435
DN24753_c0_g1_i1_1	10.37036	18.36043
DN24756_c0_g1_i1_2	1.48126	1.540316
DN24758_c0_g1_i1_2	0.7304357	0.1337066
DN24759_c0_g1_i1_1	0.8997254	3.438331

DN24763_c0_g1_i1_1	1.269554	14.21463
DN24764_c0_g1_i1_1	0.5686797	0.6200298
DN24767_c0_g1_i1_2	2.62877	1.007631
DN24768_c0_g1_i1_1	0.2540337	0.6059406
DN24771_c0_g1_i1_1	1.696827	1.552856
DN24772_c0_g1_i1_1	0.9616779	2.887568
DN24773_c0_g1_i1_1	0.4508806	0.6538532
DN24775_c0_g1_i1_2	1.250839	0.4157547
DN24782_c0_g1_i1_1	2.923568	3.132617
DN24788_c0_g1_i1_2	12.90451	21.91929
DN24789_c0_g1_i1_1	1.360483	1.765369
DN24793_c0_g1_i1_1	2.758398	6.519627
DN24794_c0_g1_i1_2	0.681261	1.234054
DN247_c0_g1_i1_1	0.1290656	1.197694
DN24801_c0_g1_i1_2	8.95513	3.05834
DN24803_c0_g1_i1_1	2.349087	5.76273
DN24804_c0_g1_i1_2	2.096855	1.216251
DN24806_c0_g1_i1_1	0.08757038	9.11E-26
DN24806_c0_g1_i1_2	0	0
DN24807_c0_g1_i1_1	1.236089	16.68806
DN24808_c0_g1_i1_2	2.619821	1.662276
DN24810_c0_g1_i1_1	57.57256	43.6243
DN24812_c0_g1_i1_1	2.927588	0.9919265
DN24816_c0_g1_i1_1	1.13174	1.449283

DN24817_c0_g1_i1_1	7.432367	5.231634
DN24817_c0_g1_i1_2	7.142085	3.920904
DN24819_c0_g1_i1_2	1.204276	3.84E-29
DN24825_c0_g1_i1_1	1.423169	1.273394
DN24831_c0_g1_i1_2	22.38537	20.53159
DN24836_c0_g1_i1_2	17.55661	27.56821
DN24837_c0_g1_i1_1	2.218036	2.0437
DN24837_c0_g1_i1_2	2.34077	0.6516896
DN24838_c0_g1_i1_1	0.7332101	0.4920004
DN24843_c0_g1_i1_2	4.220861	11.1775
DN24844_c0_g1_i1_1	7.474093	2.700885
DN24845_c0_g1_i1_2	0.9128694	0.6092805
DN24848_c0_g1_i1_1	29.4217	25.91929
DN2484_c0_g1_i1_1	0.184715	0.6001864
DN24850_c0_g1_i1_1	0.5862948	1.698385
DN24851_c0_g1_i1_1	0.8155593	0.2012431
DN24854_c0_g1_i1_2	0.425394	0.7975331
DN24856_c0_g1_i1_2	7.615785	35.03582
DN24858_c0_g1_i1_1	2.28291	2.01908
DN24859_c0_g1_i1_1	0	0
DN24859_c0_g1_i1_2	26.41575	13.33162
DN24860_c0_g1_i1_2	1.68578	0.1731223
DN24862_c0_g1_i1_1	2.098059	2.191749
DN24865_c0_g1_i1_1	0.4928057	0.588977

DN24868_c0_g1_i1_1	2.868192	3.526472
DN24871_c0_g1_i1_1	1.032139	1.939169
DN24873_c0_g1_i1_1	1.120375	3.32647
DN24875_c0_g1_i1_2	3.619629	1.685586
DN24878_c0_g1_i1_2	1.9899	0.3430478
DN24879_c0_g1_i1_2	1.883147	0.9785486
DN24880_c0_g1_i1_2	9.814774	1.927201
DN24883_c0_g1_i1_1	56.32962	13.42835
DN24884_c0_g1_i1_2	9.987639	7.317393
DN24885_c0_g1_i1_1	0.5173304	1.819958
DN24887_c0_g1_i1_2	1.84778	0.1601234
DN24890_c0_g1_i1_1	20.94072	13.42103
DN24895_c0_g1_i1_2	0.5441196	0.5515585
DN24896_c0_g1_i1_2	4.831014	5.704666
DN2489_c0_g1_i1_2	3.786865	0
DN248_c0_g1_i1_1	0	3.388602
DN24900_c0_g1_i1_2	1.132433	1.380198
DN24905_c0_g1_i1_1	1.864186	3.12179
DN24905_c0_g1_i1_2	0.7682442	1.517627
DN24917_c0_g1_i1_2	1.931885	0.05676837
DN24918_c0_g1_i1_2	2.848881	3.019472
DN24923_c0_g1_i1_1	0.5906834	0.89233
DN24928_c0_g1_i1_2	3.65562	4.50812
DN24931_c0_g1_i1_1	17.49263	6.807726

DN24935_c0_g1_i1_1	0.9228845	0.6956252
DN24935_c0_g1_i1_2	1.805343	0.3346901
DN24937_c0_g1_i1_1	0.219052	0.2240024
DN24939_c0_g1_i1_2	3.070815	0.4655798
DN24941_c0_g1_i1_1	0.5243099	1.134189
DN24943_c0_g1_i1_2	1.64867	0.7619909
DN24947_c0_g1_i1_1	0.7944846	0.5688539
DN24950_c0_g1_i1_1	0.2800557	2.081222
DN24950_c0_g1_i1_2	2.776782	1.574802
DN24952_c0_g1_i1_2	1.875328	0
DN24957_c0_g1_i1_2	1.759359	1.445437
DN24958_c0_g1_i1_1	0.3135722	0.8167195
DN2495_c0_g1_i1_1	1.374582	2.618636
DN24960_c0_g1_i1_1	0.9491137	0.3670021
DN24962_c0_g1_i1_1	0.3611777	0.9035461
DN24964_c0_g1_i1_2	0	0
DN24965_c0_g1_i1_1	1.39358	2.230685
DN24967_c0_g1_i1_1	3.912901	8.804785
DN24971_c0_g1_i1_2	2.821408	0.7664777
DN24975_c0_g1_i1_1	0.2067224	1.02026
DN24982_c0_g1_i1_1	0.3490346	1.554562
DN24984_c0_g1_i1_2	0.8460421	0.3221156
DN24991_c0_g1_i1_1	6.303618	4.633813
DN24993_c0_g1_i1_1	2.878726	2.666521

DN24997_c0_g1_i1_2	1.541716	1.533256
DN24998_c0_g1_i1_1	0.9579175	2.893866
DN25000_c0_g1_i1_2	0	0
DN25003_c0_g1_i1_2	4.320139	2.882952
DN25005_c0_g1_i1_1	0.1249302	0.5197312
DN25006_c0_g1_i1_1	0.5321324	0.827253
DN25006_c0_g1_i1_2	1.626186	0.8142021
DN25009_c0_g1_i1_1	1.114945	3.011863
DN25012_c0_g1_i1_1	0.8396755	3.311639
DN25014_c0_g1_i1_2	2.165416	1.663794
DN25016_c0_g1_i1_1	0.7772932	6.425016
DN25017_c0_g1_i1_1	0.116316	0.2245854
DN25018_c0_g1_i1_1	0.6416962	9.221037
DN25018_c0_g1_i1_2	7.170436	1.478238
DN25019_c0_g1_i1_1	2.950908	3.125678
DN2501_c0_g1_i1_2	1.960455	4.958629
DN25020_c0_g1_i1_2	1.386086	0.9475064
DN25028_c0_g1_i1_1	0.6460013	0.8361905
DN25030_c0_g1_i1_1	40.40069	23.11724
DN25031_c0_g1_i1_1	0.8736946	18.87677
DN25035_c0_g1_i1_1	1.267705	24.10283
DN25036_c0_g1_i1_1	1.555251	2.176539
DN25037_c0_g1_i1_2	0.8884692	0.780941
DN2503_c0_g1_i1_2	1.101372	0

DN25043_c0_g1_i1_1	0.872068	0.9728343
DN25044_c0_g1_i1_2	1.859689	0.8783408
DN25046_c0_g1_i1_1	0.8931172	1.76483
DN25047_c0_g1_i1_1	1.886179	3.492547
DN25048_c0_g1_i1_1	1.600636	1.506686
DN25050_c0_g1_i1_1	2.204441	4.859161
DN25054_c0_g1_i1_1	0.7000485	2.20253
DN25058_c0_g1_i1_1	0.7027289	0.4101815
DN25060_c0_g1_i1_1	1.355734	1.326168
DN25062_c0_g1_i1_2	29.01954	9.498934
DN25063_c0_g1_i1_2	0	0
DN25069_c0_g1_i1_1	1.628599	5.641647
DN25070_c0_g1_i1_2	5.280802	0.8170411
DN25071_c0_g1_i1_2	7.129818	1.552002
DN25072_c0_g1_i1_1	0.7213595	1.70426
DN25073_c0_g1_i1_1	15.54581	10.34439
DN25073_c0_g1_i1_2	1.656904	0.9721561
DN25075_c0_g1_i1_1	0.4326033	3.261157
DN25075_c0_g1_i1_2	2.814904	3.469005
DN25077_c0_g1_i1_1	1.175303	2.762985
DN25078_c0_g1_i1_1	0	2.870778
DN25078_c0_g1_i1_2	2.550942	1.506153
DN25079_c0_g1_i1_2	9.931103	11.10361
DN25083_c0_g1_i1_2	0	2.7558

DN25086_c0_g1_i1_1	1.860975	0.8287033
DN2508_c0_g1_i1_1	5.836751	5.697045
DN25090_c0_g1_i1_2	73.25197	213.9093
DN25099_c0_g1_i1_2	2.790801	0
DN25101_c0_g1_i1_1	1.14634	0.9607938
DN25105_c0_g1_i1_1	2.11019	1.554532
DN25108_c0_g1_i1_2	15.16024	18.22171
DN25112_c0_g1_i1_1	0.4159806	0.4490756
DN25112_c0_g1_i1_2	35.84142	8.282847
DN25115_c0_g1_i1_1	0.2566882	1.157734
DN25118_c0_g1_i1_1	1.329112	2.424637
DN25120_c0_g1_i1_1	0.2539743	0.4262786
DN25121_c0_g1_i1_1	0	0.3901657
DN25123_c0_g1_i1_2	0	1.242876
DN25124_c0_g1_i1_2	5.581289	3.30659
DN25126_c0_g1_i1_1	0.2190317	0.2329414
DN25131_c0_g1_i1_1	0	0.4850348
DN25133_c0_g1_i1_2	1.116242	0
DN25135_c0_g1_i1_1	5.179819	2.859068
DN25137_c0_g1_i1_1	3.420013	0.3057728
DN25137_c0_g1_i1_2	1.230026	0.1318704
DN25140_c0_g1_i1_2	3.485012	0.7193866
DN25141_c0_g1_i1_1	22.32325	33.04924
DN25142_c0_g1_i1_2	16.72384	19.69368

DN25143_c0_g1_i1_1	1.761337	1.570877
DN25148_c0_g1_i1_2	2.288411	1.396321
DN25150_c0_g1_i1_1	0.9382015	1.070732
DN25151_c0_g1_i1_1	0.6590087	0.5703529
DN25152_c0_g1_i1_1	1.712226	3.015185
DN25153_c0_g1_i1_1	0.228978	0.6526007
DN25153_c0_g1_i1_2	1.327319	1.645412
DN25154_c0_g1_i1_2	1.513691	0.5512041
DN25156_c0_g1_i1_1	3.97729	2.041376
DN25159_c0_g1_i1_1	0.9202653	2.827793
DN25160_c0_g1_i1_1	21.71585	13.89967
DN25161_c0_g1_i1_2	61.64687	8.37452
DN25164_c0_g1_i1_1	4.917934	0
DN25164_c0_g1_i1_2	1.77752	1.584812
DN25165_c0_g1_i1_1	1.092975	1.526956
DN25167_c0_g1_i1_1	0.8066532	0.3029147
DN25170_c0_g1_i1_2	11.58495	6.492999
DN25171_c0_g1_i1_2	0.2322769	0.7800985
DN25173_c0_g1_i1_1	0.1787161	0.7356893
DN25173_c0_g1_i1_2	2.259764	0.3219769
DN25174_c0_g1_i1_2	7.471121	4.184142
DN25176_c0_g1_i1_1	1.391164	2.690811
DN25182_c0_g1_i1_2	0.3233258	3.403479
DN25184_c0_g1_i1_1	1.321773	0.9730801

DN25186_c0_g1_i1_2	2.461118	1.741731
DN2518_c0_g1_i1_1	0	1.234238
DN25190_c0_g1_i1_2	181.0828	78.66092
DN25195_c0_g1_i1_1	2.800507	7.73E-31
DN25195_c0_g1_i1_2	3.575697	1.648532
DN25196_c0_g1_i1_2	4.050685	3.483762
DN25197_c0_g1_i1_1	0.8373774	0.3357701
DN25197_c0_g1_i1_2	2.668819	0.7161188
DN25200_c0_g1_i1_2	1.413933	2.206164
DN25208_c0_g1_i1_2	1.312455	0.8004878
DN25209_c0_g1_i1_1	1.57397	1.104423
DN25210_c0_g1_i1_1	1.907682	3.005272
DN25211_c0_g1_i1_1	2.646683	1.120074
DN25215_c0_g1_i1_2	2.410398	0.2343548
DN25216_c0_g1_i1_1	18.7189	16.57393
DN25216_c0_g1_i1_2	1.240481	1.654092
DN25224_c0_g1_i1_1	0.1681639	0.3392389
DN25225_c0_g1_i1_1	1.092255	0.6653165
DN25229_c0_g1_i1_2	4.036544	1.642448
DN25232_c0_g1_i1_1	1.993643	1.528145
DN25235_c0_g1_i1_1	3.436238	3.348034
DN25235_c0_g1_i1_2	1.672465	0.6705206
DN25236_c0_g1_i1_1	1.644438	2.704567
DN25246_c0_g1_i1_1	0.3160008	0.8502419

DN25248_c0_g1_i1_2	2.19912	1.109135
DN2524_c0_g1_i1_1	1.231684	1.555595
DN25251_c0_g1_i1_1	0.898517	10.05219
DN25253_c0_g1_i1_1	2.905391	6.690785
DN25253_c0_g1_i1_2	0.4401371	0
DN25254_c0_g1_i1_2	0.6891722	0.4252893
DN25257_c0_g1_i1_2	0.7150591	0.2464547
DN25258_c0_g1_i1_1	0.7088237	1.797687
DN25258_c0_g1_i1_2	0.6709683	0.302463
DN25259_c0_g1_i1_1	0	5.772698
DN25259_c0_g1_i1_2	1.07884	0.2316382
DN25261_c0_g1_i1_1	18.13585	9.481938
DN25262_c0_g1_i1_1	1.209586	0.9528419
DN25262_c0_g1_i1_2	1.775993	1.342854
DN25263_c0_g1_i1_1	2.140267	3.223102
DN25264_c0_g1_i1_1	0.4407986	1.456717
DN25265_c0_g1_i1_1	0.8676713	1.413152
DN25266_c0_g1_i1_1	12.4555	9.270188
DN25266_c0_g1_i1_2	0.7083145	0.1600491
DN25270_c0_g1_i1_1	1.706076	1.842453
DN25271_c0_g1_i1_1	0.8962982	2.154737
DN25272_c0_g1_i1_1	4.581713	11.75951
DN25275_c0_g1_i1_1	8.816226	3.049462
DN25275_c0_g1_i1_2	0	0

DN25280_c0_g1_i1_1	39.22899	24.91747
DN25282_c0_g1_i1_1	0.7326704	1.190021
DN25282_c0_g1_i1_2	4.770992	1.592413
DN25285_c0_g1_i1_1	0.3603751	1.79483
DN25287_c0_g1_i1_1	0.9323958	1.334247
DN2528_c0_g1_i1_2	0.6504775	0.3652698
DN2528_c0_g2_i1_2	1.184209	0
DN25290_c0_g1_i1_2	0.9300296	0.979838
DN25291_c0_g1_i1_1	3.405703	1.017014
DN25295_c0_g1_i1_1	4.590419	1.618418
DN25296_c0_g1_i1_1	1.019507	4.29646
DN252_c0_g1_i1_2	11.60199	14.92168
DN25300_c0_g1_i1_1	0.6115395	1.25595
DN25308_c0_g1_i1_2	0.3623179	3.070134
DN25309_c0_g1_i1_2	0.7822696	0.4664729
DN25310_c0_g1_i1_1	2.384583	2.133695
DN25314_c0_g1_i1_1	0.7405964	0.4218531
DN25315_c0_g1_i1_1	0.2722931	0
DN25317_c0_g1_i1_1	0.6124016	1.592061
DN25321_c0_g1_i1_1	0.8610625	0
DN25321_c0_g1_i1_2	32.54101	2.467214
DN25322_c0_g1_i1_1	0.2257452	1.456315
DN25327_c0_g1_i1_1	1.448557	1.632125
DN25328_c0_g1_i1_1	0.8754495	1.904944

DN25329_c0_g1_i1_1	1.154236	0.9721738
DN2532_c0_g1_i1_1	0.0716329	0.4001517
DN25330_c0_g1_i1_1	2.23181	2.770814
DN25335_c0_g1_i1_1	3.556255	5.78078
DN25336_c0_g1_i1_2	3.143111	0
DN25339_c0_g1_i1_2	2.030443	0.6608949
DN2533_c0_g2_i1_2	5.471735	1.853178
DN25342_c0_g1_i1_2	2.567807	0.4707998
DN25343_c0_g1_i1_1	1.107	1.530779
DN25349_c0_g1_i1_1	7.894999	4.947942
DN25356_c0_g1_i1_1	31.31743	40.68575
DN25357_c0_g1_i1_1	1.937622	1.070002
DN25358_c0_g1_i1_1	3.017788	1.826085
DN25360_c0_g1_i1_1	4.739751	2.529355
DN25361_c0_g1_i1_1	1.172274	0.5932273
DN25366_c0_g1_i1_1	0.4638576	0.1815622
DN25367_c0_g1_i1_1	0.9808567	1.302286
DN2536_c0_g1_i1_1	1.028279	0
DN25371_c0_g1_i1_1	1.395428	1.650188
DN25372_c0_g1_i1_1	0.8742717	0.6922541
DN25374_c0_g1_i1_1	0.5182709	0.5651422
DN25380_c0_g1_i1_1	0.5327106	1.037223
DN25381_c0_g1_i1_1	2.785278	2.233921
DN25382_c0_g1_i1_1	1.515257	1.141789

DN25383_c0_g1_i1_1	0	1.43876
DN25383_c0_g1_i1_2	8.850375	1.249268
DN25384_c0_g1_i1_1	0.3822197	1.583046
DN25386_c0_g1_i1_1	1.908711	0.5928241
DN25387_c0_g1_i1_1	2.92548	1.839257
DN25387_c0_g1_i1_2	4.511105	1.776298
DN25390_c0_g1_i1_1	1.052246	0.9064335
DN25391_c0_g1_i1_1	0	1.453751
DN25393_c0_g1_i1_2	0.3708773	0
DN25395_c0_g1_i1_1	0.7449441	0.7527489
DN25396_c0_g1_i1_2	1.485206	0.9242966
DN25399_c0_g1_i1_1	0	0
DN25403_c0_g1_i1_1	0	1.170473
DN25409_c0_g1_i1_1	2.394519	7.872278
DN25410_c0_g1_i1_1	0.514342	1.167593
DN25411_c0_g1_i1_2	3.031553	1.874643
DN25412_c0_g1_i1_1	0.9257098	1.196457
DN25412_c0_g1_i1_2	1.027454	0.3527241
DN25414_c0_g1_i1_1	2.07633	0.7491104
DN25415_c0_g1_i1_1	0.1266004	1.475377
DN25420_c0_g1_i1_1	2.87744	5.467356
DN25421_c0_g1_i1_1	1.71752	5.289466
DN25422_c0_g1_i1_1	1.486539	2.212905
DN25424_c0_g1_i1_1	1.314978	1.140083

DN25424_c0_g1_i1_2	4.188462	2.423976
DN25428_c0_g1_i1_1	0.8907507	2.677497
DN25429_c0_g1_i1_1	4.041484	2.801822
DN25436_c0_g1_i1_1	0.3119877	1.142608
DN25440_c0_g1_i1_1	6.888172	2.714409
DN25446_c0_g1_i1_2	0.8681226	0.212722
DN25449_c0_g1_i1_1	2.359379	3.504733
DN25450_c0_g1_i1_1	51.05044	72.41083
DN25451_c0_g1_i1_2	1.091225	0.7462508
DN25452_c0_g1_i1_1	137.2028	250.2375
DN25453_c0_g1_i1_2	1.821716	0.2873983
DN25454_c0_g1_i1_1	4.309955	4.865341
DN25456_c0_g1_i1_2	2.795066	0.7404745
DN25457_c0_g1_i1_1	0.4026179	1.294584
DN25457_c0_g1_i1_2	7.278063	1.038832
DN25459_c0_g1_i1_1	3.865967	3.56953
DN2545_c0_g1_i1_1	2.158035	2.791035
DN25461_c0_g1_i1_2	1.257392	0.3648586
DN25462_c0_g1_i1_2	5.403319	1.381019
DN25464_c0_g1_i1_1	1.521888	1.537329
DN25469_c0_g1_i1_1	1.075417	3.10351
DN25469_c0_g1_i1_2	0.5573737	0
DN2546_c0_g1_i1_1	0.2446746	0
DN25470_c0_g1_i1_2	0.2001473	0.4578516

DN25475_c0_g1_i1_2	0.8281869	0.6981182
DN25476_c0_g1_i1_1	1.358259	3.920673
DN25478_c0_g1_i1_2	0	0
DN25479_c0_g1_i1_2	1.810421	0.1673668
DN25480_c0_g1_i1_1	2.468605	3.192144
DN25483_c0_g1_i1_2	0.320623	0.7121088
DN25484_c0_g1_i1_1	73.92614	1.51269
DN25484_c0_g1_i1_2	3.958182	3.288216
DN25486_c0_g1_i1_1	1.036878	8.865337
DN25487_c0_g1_i1_1	2.52381	1.575091
DN25489_c0_g1_i1_1	13.56759	5.042208
DN2548_c0_g2_i1_1	0.465028	1.071038
DN25491_c0_g1_i1_2	2.003083	3.415733
DN25492_c0_g1_i1_1	0.4590123	2.18007
DN25495_c0_g1_i1_1	9.755798	16.15103
DN25498_c0_g1_i1_1	0.532197	1.77433
DN25498_c0_g1_i1_2	1.773252	2.288312
DN25499_c0_g1_i1_1	0	0
DN25500_c0_g1_i1_1	0.9594682	0.1772709
DN25503_c0_g1_i1_1	0.8057513	1.829059
DN25506_c0_g1_i1_1	2.173153	5.892302
DN25506_c0_g1_i1_2	4.591657	0.4656865
DN25507_c0_g1_i1_1	1.536499	1.828665
DN25508_c0_g1_i1_1	21.97324	14.70274

DN2550_c0_g1_i1_1	0.1319807	0.1952014
DN25510_c0_g1_i1_1	1.348947	0.3832979
DN25514_c0_g1_i1_1	3.506233	3.034605
DN25515_c0_g1_i1_1	3.901683	1.03324
DN25516_c0_g1_i1_1	0.5988827	0
DN25517_c0_g1_i1_1	3.732058	5.391808
DN25518_c0_g1_i1_1	18.00932	6.10861
DN2551_c0_g1_i1_1	0.5882354	0.2575952
DN25520_c0_g1_i1_1	0.09551457	2.354796
DN25522_c0_g1_i1_1	0.1342391	0.3365293
DN25523_c0_g1_i1_2	1.164848	1.51003
DN25524_c0_g1_i1_1	0.1902567	1.350253
DN25526_c0_g1_i1_1	5.549368	7.459742
DN25528_c0_g1_i1_1	1.00527	2.762502
DN25531_c0_g1_i1_1	0.8163147	0.5975002
DN25531_c0_g1_i1_2	0	0
DN25532_c0_g1_i1_1	0.6794474	3.304827
DN25533_c0_g1_i1_1	1.446924	2.184578
DN25533_c0_g1_i1_2	0.3281534	0.3349689
DN25534_c0_g1_i1_2	2.240367	1.416662
DN25537_c0_g1_i1_2	2.145213	0.6202216
DN25538_c0_g1_i1_1	0.6280078	1.506527
DN25539_c0_g1_i1_2	29.08729	22.11705
DN2553_c0_g2_i1_1	0.9608653	0.8460387

DN25542_c0_g1_i1_1	2.312001	3.365433
DN25544_c0_g1_i1_2	6.789239	5.562315
DN25551_c0_g1_i1_2	0.928903	0.5660217
DN25552_c0_g1_i1_1	0.8733805	0.166015
DN25552_c0_g1_i1_2	1.657917	2.067684
DN25554_c0_g1_i1_1	0.3326347	0
DN25559_c0_g1_i1_1	1.70221	1.886056
DN25560_c0_g1_i1_1	10.21806	7.028071
DN25562_c0_g1_i1_1	0.6479234	0.9360088
DN25563_c0_g1_i1_1	0.7548673	0.6660036
DN25566_c0_g1_i1_1	0	6.231353
DN25567_c0_g1_i1_1	0	0
DN2556_c0_g1_i1_1	0.2387616	0.945281
DN25570_c0_g1_i1_2	2.243057	6.33288
DN25571_c0_g1_i1_1	0.7645078	1.847633
DN25574_c0_g1_i1_1	0.3025951	4.881093
DN25575_c0_g1_i1_1	2.04201	1.765376
DN25579_c0_g1_i1_2	3.66157	0.7209075
DN2557_c0_g1_i1_2	1.053009	1.175794
DN25580_c0_g1_i1_1	4.499754	6.690289
DN25581_c0_g1_i1_2	0.739718	2.497043
DN25584_c0_g1_i1_2	2.07993	0.1255677
DN25588_c0_g1_i1_1	1.026723	2.927437
DN25589_c0_g1_i1_2	1.093288	0.1114187

DN2558_c0_g1_i1_1	0	0
DN25590_c0_g1_i1_1	0.8404527	0.9188892
DN25590_c0_g1_i1_2	0.5319753	0.3768452
DN25591_c0_g1_i1_1	0	1.053268
DN25592_c0_g1_i1_1	1.367226	2.028275
DN25594_c0_g1_i1_2	0.8324875	0.4462143
DN25595_c0_g1_i1_2	2.681836	2.800476
DN25597_c0_g1_i1_1	74.58383	59.61301
DN2559_c0_g1_i2_2	3.607231	2.924525
DN25601_c0_g1_i1_1	2.629414	2.845322
DN25603_c0_g1_i1_1	0	1.5023
DN25603_c0_g1_i1_2	5.656055	1.89798
DN25604_c0_g1_i1_1	1.117844	1.50894
DN25607_c0_g1_i1_1	2.523169	5.040878
DN25609_c0_g1_i1_1	0.5505293	0.5243046
DN2560_c0_g1_i1_1	1.24292	1.773696
DN25612_c0_g1_i1_2	0.5646133	1.942164
DN25613_c0_g1_i1_1	0.155183	0.8576982
DN25615_c0_g1_i1_1	1.69697	1.238688
DN25616_c0_g1_i1_2	495.3599	209.4555
DN25622_c0_g1_i1_1	0.9296304	0.6081484
DN25623_c0_g1_i1_2	1.013331	0.9425136
DN25624_c0_g1_i1_1	6.499611	9.710376
DN2562_c0_g1_i1_1	2.388901	2.409645

DN2562_c0_g2_i1_1	11.2856	16.02519
DN25633_c0_g1_i1_2	1.645669	0.1119502
DN25638_c0_g1_i1_1	1.086398	1.297501
DN2563_c0_g1_i1_2	3.516185	1.180281
DN25640_c0_g1_i1_1	0.9446222	2.445483
DN25643_c0_g1_i1_1	3.274336	9.763114
DN25645_c0_g1_i1_1	0.3073349	0.7324073
DN25645_c0_g1_i1_2	235.9185	73.80197
DN25646_c0_g1_i1_1	0	1.536705
DN25649_c0_g1_i1_1	0.7792444	2.241838
DN25659_c0_g1_i1_1	0	0.3331063
DN25659_c0_g1_i1_2	1.587962	0.1527775
DN25660_c0_g1_i1_1	3.351728	1.961633
DN25661_c0_g1_i1_2	17.3435	3.268688
DN25662_c0_g1_i1_1	1.013799	2.565626
DN25663_c0_g1_i1_1	0.6061534	0.1775644
DN25663_c0_g1_i1_2	60.93038	29.81849
DN25664_c0_g1_i1_1	0.6731545	1.277755
DN25667_c0_g1_i1_1	4.888293	2.335184
DN2566_c0_g1_i1_1	3.924531	5.132845
DN25671_c0_g1_i1_1	13.05862	15.79247
DN25671_c0_g1_i1_2	129.1353	62.41465
DN25673_c0_g1_i1_2	1.455043	0
DN25675_c0_g1_i1_1	1.853418	0.759467

DN25680_c0_g1_i1_1	0.1921865	0.6687176
DN25681_c0_g1_i1_1	1.291686	1.828232
DN25682_c0_g1_i1_2	2.795744	2.827451
DN25685_c0_g1_i1_1	3.678015	4.040551
DN25686_c0_g1_i1_1	0	1.584226
DN25689_c0_g1_i1_1	0.3941204	0.5649627
DN25689_c0_g1_i1_2	1.748706	1.544417
DN2568_c0_g1_i2_2	0	0
DN25690_c0_g1_i1_1	0.8751559	0.4493367
DN25691_c0_g1_i1_2	1.070187	0
DN25693_c0_g1_i1_2	0	0.515962
DN25701_c0_g1_i1_1	3.183074	2.191983
DN25701_c0_g1_i1_2	0.6241223	0.6668739
DN25707_c0_g1_i1_1	7.607717	3.725345
DN25708_c0_g1_i1_1	0.349242	0.142566
DN25710_c0_g1_i1_2	4.209896	2.528707
DN25713_c0_g1_i1_2	1.858547	0
DN25716_c0_g1_i1_2	69.89913	30.04218
DN25724_c0_g1_i1_2	11.43323	6.996582
DN25728_c0_g1_i1_1	0.4849262	1.1575
DN25733_c0_g1_i1_1	21.42284	19.82573
DN25734_c0_g1_i1_1	0.948151	1.704091
DN25737_c0_g1_i1_1	1.536898	1.093185
DN25738_c0_g1_i1_1	0.5650085	0

DN25738_c0_g1_i1_2	1.570775	1.123987
DN25745_c0_g1_i1_1	4.018555	1.362015
DN25745_c0_g1_i1_2	1.980696	1.067306
DN25746_c0_g1_i1_1	1.975388	1.568147
DN25747_c0_g1_i1_1	2.184955	8.804509
DN25748_c0_g1_i1_1	16.90054	3.882808
DN25749_c0_g1_i1_2	3.359948	1.17659
DN25750_c0_g1_i1_1	2.093564	2.893694
DN25750_c0_g1_i1_2	0.1323175	2.313204
DN25751_c0_g1_i1_2	3.00612	0.665501
DN25752_c0_g1_i1_1	1.597083	1.502829
DN25754_c0_g1_i1_1	3.549091	7.068805
DN25755_c0_g1_i1_1	0	0.1423011
DN25756_c0_g1_i1_1	0.7717332	0.1934852
DN25760_c0_g1_i1_1	0.3045171	0.6883664
DN25762_c0_g1_i1_2	4.771235	2.739579
DN25763_c0_g1_i1_2	0.9688823	0.5120266
DN25765_c0_g1_i1_2	1.75651	0.2286609
DN25766_c0_g1_i1_2	1.197477	1.069967
DN25767_c0_g1_i1_1	1.068186	0.5883818
DN25768_c0_g1_i1_1	3.209646	0.838493
DN25769_c0_g1_i1_2	3.713912	0.8347803
DN25770_c0_g1_i1_1	4.160295	6.634232
DN25773_c0_g1_i1_2	3.463392	0

DN25778_c0_g1_i1_1	0.8646974	1.161876
DN25780_c0_g1_i1_2	0.6154269	0.5925142
DN25791_c0_g1_i1_1	1.476488	0.5831444
DN25792_c0_g1_i1_2	1.175683	0.3987605
DN25793_c0_g1_i1_1	0.6224598	0.566575
DN25794_c0_g1_i1_1	0	2.229522
DN25799_c0_g1_i1_1	0.4229271	0.719041
DN25801_c0_g1_i1_2	6.381552	2.213549
DN25802_c0_g1_i1_1	1.168593	1.307586
DN25803_c0_g1_i1_1	0.6844753	0.2163659
DN25804_c0_g1_i1_1	0.2810794	0.9271498
DN25810_c0_g1_i1_1	1.826498	1.424243
DN25814_c0_g1_i1_2	1.602877	1.891905
DN25816_c0_g1_i1_1	1.615823	4.023967
DN25818_c0_g1_i1_1	0.4504188	0.3504499
DN25821_c0_g1_i1_2	11.74629	10.53515
DN25822_c0_g1_i1_1	1.668464	1.175326
DN25822_c0_g1_i1_2	13.81352	7.007785
DN25824_c0_g1_i1_1	0.7805792	2.088186
DN25824_c0_g1_i1_2	1.843019	2.306348
DN25826_c0_g1_i1_1	0.2823852	0.7896939
DN25828_c0_g1_i1_2	1.424405	0.5879215
DN25829_c0_g1_i1_1	1.432751	2.827379
DN2582_c0_g1_i1_1	5.692	5.207323

DN25836_c0_g1_i1_2	0.8973989	0.1577096
DN25838_c0_g1_i1_1	3.132905	1.306311
DN25839_c0_g1_i1_2	2.696128	2.236005
DN25844_c0_g1_i1_2	10.86382	6.206544
DN25850_c0_g1_i1_1	2.887495	7.78E-28
DN25851_c0_g1_i1_1	2.066769	2.847994
DN25858_c0_g1_i1_1	0	1.252759
DN25860_c0_g1_i1_1	0.19764	0
DN25860_c0_g1_i1_2	0.8406219	0
DN25862_c0_g1_i1_1	0	0.201042
DN25865_c0_g1_i1_1	0	0.3776752
DN25866_c0_g1_i1_2	5.408326	4.575363
DN25869_c0_g1_i1_2	0.6276153	0.7120093
DN25871_c0_g1_i1_1	2.442233	2.145677
DN25872_c0_g1_i1_1	1.760474	0.6331668
DN25874_c0_g1_i1_1	46.78559	89.85828
DN25875_c0_g1_i1_1	0.9367894	0.554401
DN25879_c0_g1_i1_1	0.9993569	0.6151856
DN25881_c0_g1_i1_1	0.9595387	2.379909
DN25885_c0_g1_i1_2	7.74265	12.90144
DN25886_c0_g1_i1_1	0.7688403	3.482168
DN25886_c0_g1_i1_2	0.4096979	0
DN25890_c0_g1_i1_1	0.7312428	0.3814394
DN25891_c0_g1_i1_1	0.2030095	0

DN25891_c0_g1_i1_2	2.156374	0.8968114
DN25893_c0_g1_i1_1	0.447853	2.355076
DN25894_c0_g1_i1_2	0.3399369	0
DN25898_c0_g1_i1_1	0	1.377084
DN25898_c0_g1_i1_2	1.977282	1.751975
DN25901_c0_g1_i1_2	0	1.746988
DN25904_c0_g1_i1_1	0.438132	0.5105255
DN25906_c0_g1_i1_1	0.3634951	0.339711
DN25907_c0_g1_i1_2	4.17226	1.117179
DN25910_c0_g1_i1_2	1.095003	0.2912238
DN25913_c0_g1_i1_1	0.5541902	1.731521
DN25918_c0_g1_i1_1	0	1.887215
DN2591_c0_g1_i1_1	0.2179383	1.295698
DN25920_c0_g1_i1_1	2.141691	1.198212
DN25921_c0_g1_i1_2	0	0
DN25922_c0_g1_i1_1	0.8812859	0.6969453
DN25923_c0_g1_i1_1	46.2278	60.44121
DN25925_c0_g1_i1_2	2.391902	1.265832
DN25929_c0_g1_i1_1	0.6387126	0.8082928
DN25933_c0_g1_i1_1	4.488078	4.42857
DN25934_c0_g1_i1_1	1.344286	0.9131314
DN25938_c0_g1_i1_1	1.666491	6.209039
DN25941_c0_g1_i1_1	0	1.71918
DN25941_c0_g1_i1_2	0	0

DN25942_c0_g1_i1_2	30.94357	93.87788
DN25947_c0_g1_i1_1	1.858495	1.571424
DN25948_c0_g1_i1_2	2.705825	0.9917504
DN25950_c0_g1_i1_2	0	0
DN25951_c0_g1_i1_2	1.788106	0.4435146
DN25956_c0_g1_i1_2	0	0
DN25958_c0_g1_i1_1	0.7561221	0.7106233
DN25958_c0_g1_i1_2	0.945593	0.8007007
DN25965_c0_g1_i1_2	1.767827	0.003023878
DN2596_c0_g1_i1_2	2.219393	0.4924127
DN25972_c0_g1_i1_1	0.3663829	0.5358233
DN25975_c0_g1_i1_1	3.12024	3.404524
DN25977_c0_g1_i1_2	1.094454	2.571061
DN25981_c0_g1_i1_2	1.796632	3.446736
DN25982_c0_g1_i1_2	0.498692	0.340422
DN25984_c0_g1_i1_1	1.533196	1.509457
DN25985_c0_g1_i1_1	1.260272	0.7271576
DN25985_c0_g1_i1_2	4.979733	3.688055
DN25987_c0_g1_i1_1	104.8599	84.38468
DN25988_c0_g1_i1_1	3.161719	3.167224
DN25991_c0_g1_i1_2	3.346744	1.773397
DN25995_c0_g1_i1_2	1.422855	0
DN25_c0_g1_i1_1	0.3163978	0.8636071
DN25_c0_g2_i1_1	0.290079	2.371409

DN26006_c0_g1_i1_2	0.320316	0.1465767
DN26010_c0_g1_i1_2	1.088509	0
DN26011_c0_g1_i1_1	0	2.183573
DN26012_c0_g1_i1_1	34.80584	10.23986
DN26015_c0_g1_i1_2	4.998897	0.5771759
DN26018_c0_g1_i1_1	0.48669	1.470623
DN26018_c0_g1_i1_2	0.4944324	0
DN26019_c0_g1_i1_1	7.293691	10.59804
DN2601_c0_g1_i1_1	1.801595	1.526357
DN26021_c0_g1_i1_1	0.8219884	0.8757193
DN26026_c0_g1_i1_2	3.728132	3.876238
DN26029_c0_g1_i1_1	0.9855778	0.8786735
DN26031_c0_g1_i1_1	0	0.9095845
DN26031_c0_g1_i1_2	0.5395354	0.2074623
DN26033_c0_g1_i1_1	0.6189148	0.2403336
DN26033_c0_g1_i1_2	2.801809	0.7415954
DN26038_c0_g1_i1_1	1.985496	1.396128
DN2603_c0_g1_i1_1	0.1090129	1.768416
DN2603_c0_g1_i2_2	1.556204	0.3001606
DN26040_c0_g1_i1_1	0.5121937	2.063145
DN26043_c0_g1_i1_1	10.12577	13.45571
DN26044_c0_g1_i1_1	3.537425	1.251911
DN26046_c0_g1_i1_2	5.581215	0.6728272
DN26049_c0_g1_i1_1	0.4672268	3.684916

DN26052_c0_g1_i1_2	1.98835	0.9773629
DN26053_c0_g1_i1_1	2.325193	3.912636
DN26057_c0_g1_i1_1	1.753454	3.781634
DN26058_c0_g1_i1_2	1.520085	0
DN26061_c0_g1_i1_1	1.1905	0.7211458
DN26061_c0_g1_i1_2	0.2951261	2.426951
DN26062_c0_g1_i1_1	0	0
DN26063_c0_g1_i1_2	1.595748	0.1600771
DN26065_c0_g1_i1_1	23.09893	14.99609
DN26067_c0_g1_i1_1	35.05177	8.261182
DN26068_c0_g1_i1_1	3.807336	4.407566
DN26068_c0_g1_i1_2	2.905333	2.659712
DN26069_c0_g1_i1_1	1.504475	1.634642
DN26074_c0_g1_i1_1	0.07982852	2.260087
DN26077_c0_g1_i1_2	2.377649	4.274423
DN26079_c0_g1_i1_2	3.699934	1.521721
DN26082_c0_g1_i1_1	0.6998441	0.1822614
DN26086_c0_g1_i1_1	5.478325	3.61725
DN26087_c0_g1_i1_2	3.941833	3.498513
DN26088_c0_g1_i1_1	7.287228	1.703238
DN26089_c0_g1_i1_2	12.29111	8.737145
DN26090_c0_g1_i1_2	0.532989	0
DN26094_c0_g1_i1_2	1.64692	0.5590272
DN26096_c0_g1_i1_1	1.83E-31	0.6937107

DN26097_c0_g1_i1_1	1.361241	1.079279
DN26097_c0_g1_i1_2	0	0
DN26099_c0_g1_i1_1	1.312499	1.361071
DN2609_c0_g1_i1_2	2.849589	1.548221
DN26106_c0_g1_i1_2	0.8072707	0.3161789
DN26108_c0_g1_i1_1	1.826022	1.625859
DN26109_c0_g1_i1_1	3.672018	4.634117
DN26109_c0_g1_i1_2	0.3375162	0
DN26117_c0_g1_i1_1	0	1.127135
DN26118_c0_g1_i1_2	1.309067	0
DN26119_c0_g1_i1_2	0.1409255	0.221517
DN2611_c0_g1_i1_1	1.340502	2.345606
DN2611_c0_g1_i1_2	1.300737	0.2374857
DN26121_c0_g1_i1_1	0.5458368	2.278919
DN26123_c0_g1_i1_2	417.9901	75.58108
DN26124_c0_g1_i1_2	20.60834	7.471331
DN26129_c0_g1_i1_1	0.7906777	1.07979
DN26130_c0_g1_i1_2	1.276694	1.753156
DN26131_c0_g1_i1_2	0.3959778	0.8305178
DN26132_c0_g1_i1_1	4.305429	3.118984
DN26139_c0_g1_i1_1	15.9592	16.66623
DN26140_c0_g1_i1_2	0	0
DN26142_c0_g1_i1_1	10.07112	9.388755
DN26142_c0_g1_i1_2	1.429262	0.4536629

DN26143_c0_g1_i1_1	1.779868	6.617366
DN26146_c0_g1_i1_2	0.9128418	0.4601098
DN26149_c0_g1_i1_1	0.5303824	0.703732
DN26152_c0_g1_i1_1	2.206488	10.39545
DN26153_c0_g1_i1_2	5.710588	9.366213
DN26154_c0_g1_i1_1	2.46412	5.844068
DN26154_c0_g1_i1_2	2.268938	3.543864
DN26157_c0_g1_i1_1	16.07037	7.032683
DN26158_c0_g1_i1_1	7.915294	1.484328
DN26161_c0_g1_i1_2	18.60465	29.21831
DN26163_c0_g1_i1_1	280.3978	292.7129
DN26167_c0_g1_i1_2	0.543849	0.9751704
DN26168_c0_g1_i1_1	0	1.011424
DN26169_c0_g1_i1_2	5.530942	6.479332
DN26170_c0_g1_i1_1	2.443336	0.4911059
DN26170_c0_g1_i1_2	0	0
DN26171_c0_g1_i1_2	1.030255	0.9495268
DN26172_c0_g1_i1_2	4.386349	5.77582
DN26174_c0_g1_i1_1	0.1033295	1.767704
DN26174_c0_g1_i1_2	1.568308	2.03E-31
DN26177_c0_g1_i1_2	2.647098	1.263791
DN26180_c0_g1_i1_1	6.441573	4.313368
DN26185_c0_g1_i1_2	2.137558	1.615008
DN26186_c0_g1_i1_2	0.7826125	0

DN26187_c0_g1_i1_2	5.00852	1.438986
DN26189_c0_g1_i1_1	1.737089	1.315779
DN26189_c0_g1_i1_2	1.821987	2.941503
DN26190_c0_g1_i1_1	3.572306	2.820559
DN26194_c0_g1_i1_1	0.4805025	1.027839
DN26194_c0_g1_i1_2	81.16678	34.6514
DN26201_c0_g1_i1_2	3.770658	1.881033
DN26203_c0_g1_i1_2	2.681919	1.225264
DN26206_c0_g1_i1_2	4.045612	1.185102
DN26207_c0_g1_i1_2	0.9250737	0.6506563
DN26209_c0_g1_i1_1	1.900999	1.850537
DN26209_c0_g1_i1_2	0.3919672	0.7622317
DN2620_c0_g1_i1_2	1.10304	0.4573042
DN26212_c0_g1_i1_1	4.148693	1.558876
DN26215_c0_g1_i1_2	2.632071	1.949631
DN26217_c0_g1_i1_1	1.407502	1.816161
DN2621_c0_g1_i1_1	3.160257	1.174703
DN26220_c0_g1_i1_1	0.8407543	0.596529
DN26221_c0_g1_i1_2	0.7232022	0
DN26223_c0_g1_i1_2	2.782615	5.840839
DN26229_c0_g1_i1_2	2.859888	0.6786562
DN2622_c0_g1_i2_1	3.345448	4.805871
DN26230_c0_g1_i1_1	0.2473693	0.8745323
DN26236_c0_g1_i1_2	14.49056	26.9894

DN26238_c0_g1_i1_1	0.7863703	0.4058458
DN2623_c0_g2_i1_1	2.858793	3.422211
DN26240_c0_g1_i1_2	3.482838	4.257277
DN26241_c0_g1_i1_2	0.1233834	0.6759641
DN26244_c0_g1_i1_1	1.081213	1.007813
DN26246_c0_g1_i1_2	0.8499492	0.3092826
DN26247_c0_g1_i1_1	1.063225	1.29805
DN26249_c0_g1_i1_1	2.448779	2.754416
DN26251_c0_g1_i1_1	0.8610439	10.84141
DN26254_c0_g1_i1_1	2.42181	2.162463
DN26254_c0_g1_i1_2	0.6266865	0.7128422
DN26255_c0_g1_i1_2	0.5487622	0.08247968
DN26256_c0_g1_i1_1	3.589011	0
DN26258_c0_g1_i1_1	0.6337552	0.5198877
DN26259_c0_g1_i1_1	10.97943	13.04851
DN26263_c0_g1_i1_1	0.4599957	3.215763
DN26265_c0_g1_i1_1	1.155674	1.03234
DN26266_c0_g1_i1_2	2.704813	3.894029
DN26270_c0_g1_i1_2	6.811389	3.281637
DN26280_c0_g1_i1_1	6.276371	16.68063
DN26281_c0_g1_i1_2	1.018009	0.3808284
DN26283_c0_g1_i1_1	2.3932	1.520179
DN26285_c0_g1_i1_1	1.108687	1.322102
DN2628_c0_g2_i1_1	1.302279	3.880176

DN26290_c0_g1_i1_2	1.664972	0.7260075
DN26296_c0_g1_i1_1	1.788051	3.736547
DN26300_c0_g1_i1_2	1.278263	4.535454
DN26302_c0_g1_i1_1	1.979888	1.761049
DN26304_c0_g1_i1_2	7.334694	5.72036
DN26306_c0_g1_i1_1	0.8521279	1.569719
DN26311_c0_g1_i1_2	0.8005329	0.4799754
DN26318_c0_g1_i1_1	0.6076161	0.8164357
DN2631_c0_g1_i1_1	0.4710736	1.309403
DN26320_c0_g1_i1_1	2.361591	2.278217
DN26324_c0_g1_i1_1	3.020726	2.027366
DN26325_c0_g1_i1_1	1.462519	1.286405
DN26326_c0_g1_i1_1	1.48377	1.330148
DN26331_c0_g1_i1_1	1.17932	0.8602411
DN26332_c0_g1_i1_1	0.5506904	12.12456
DN26341_c0_g1_i1_1	0.2502807	0.6798083
DN26343_c0_g1_i1_1	5.055116	4.673448
DN26350_c0_g1_i1_1	11.28072	3.429098
DN26358_c0_g1_i1_1	0.8265557	2.10702
DN26360_c0_g1_i1_1	2.310684	4.325454
DN26361_c0_g1_i1_2	1.498306	1.348503
DN26364_c0_g1_i1_2	34.55856	28.75015
DN26371_c0_g1_i1_1	2.103202	2.195782
DN26372_c0_g1_i1_1	52.55163	23.97051

DN26373_c0_g1_i1_1	0.4464112	0.21743
DN26377_c0_g1_i1_1	0.433304	2.814075
DN26378_c0_g1_i1_1	1.863144	2.984428
DN2637_c0_g1_i1_1	0.9349977	1.591391
DN26387_c0_g1_i1_2	5.857798	4.169337
DN26393_c0_g1_i1_1	0.6828329	0.867891
DN26396_c0_g1_i1_1	8.526426	9.975117
DN26397_c0_g1_i1_1	6.113798	7.590094
DN26398_c0_g1_i1_2	1.407027	0.7057362
DN26399_c0_g1_i1_1	2.783919	3.286605
DN26408_c0_g1_i1_1	0.6530504	0.754254
DN2640_c0_g1_i1_1	1.114534	2.920547
DN26412_c0_g1_i1_1	1.852849	1.161321
DN26412_c0_g1_i1_2	2.797785	0.254388
DN26417_c0_g1_i1_1	0.5078929	0.7765485
DN26417_c0_g1_i1_2	3.782487	2.819684
DN26430_c0_g1_i1_1	1.957177	3.444229
DN26430_c0_g1_i1_2	13.16091	5.836758
DN26431_c0_g1_i1_1	0.9809025	2.351481
DN26433_c0_g1_i1_1	0.8361549	1.135302
DN26440_c0_g1_i1_1	0	1.458726
DN26441_c0_g1_i1_2	20.70678	3.689151
DN26448_c0_g1_i1_1	9.01287	2.394763
DN26449_c0_g1_i1_2	3.193052	1.174625

DN26450_c0_g1_i1_1	0.5135622	1.579388
DN26459_c0_g1_i1_1	7.065326	6.979895
DN2645_c0_g1_i1_2	1.193635	2.367504
DN26466_c0_g1_i1_1	2.259537	2.605477
DN26471_c0_g1_i1_2	23.35947	9.930211
DN26473_c0_g1_i1_1	1.949923	2.818518
DN26478_c0_g1_i1_2	0.5558674	0.7679283
DN2647_c0_g1_i1_1	1.491012	0.890967
DN26481_c0_g1_i1_1	1.051457	1.504771
DN26485_c0_g1_i1_1	2.384357	1.989101
DN26491_c0_g1_i1_1	13.50747	65.94816
DN26493_c0_g1_i1_1	0	8.670387
DN26501_c0_g1_i1_1	0.1086791	0.4145963
DN26502_c0_g1_i1_1	0.1781472	0.6946521
DN26504_c0_g1_i1_1	0.1840363	1.99909
DN2650_c0_g1_i1_1	1.243286	0.9595134
DN26513_c0_g1_i1_2	13.7724	4.746556
DN26515_c0_g1_i1_1	0.4315711	1.758959
DN26518_c0_g1_i1_2	1.908387	1.739805
DN26521_c0_g1_i1_2	5.536292	1.461233
DN26523_c0_g1_i1_1	8.910063	8.78048
DN26524_c0_g1_i1_1	0.4173701	1.883829
DN26525_c0_g1_i1_1	0.648584	0.7574909
DN26528_c0_g1_i1_1	2.960073	5.129276

DN26528_c0_g1_i1_2	0.542665	0.397273
DN26529_c0_g1_i1_1	3.644036	2.639818
DN26536_c0_g1_i1_1	1.383394	1.707724
DN26537_c0_g1_i1_2	3.522511	1.739929
DN26538_c0_g1_i1_1	0.5531325	7.251792
DN26542_c0_g1_i1_2	3.322396	1.302197
DN26543_c0_g1_i1_1	0.3574985	1.439883
DN26544_c0_g1_i1_1	1.450257	3.338198
DN26546_c0_g1_i1_1	0.8592735	0.8089108
DN26549_c0_g1_i1_2	0	0
DN2654_c0_g1_i1_2	0	0
DN26554_c0_g1_i1_1	3.545885	10.65201
DN26557_c0_g1_i1_2	0.2490428	0.1366608
DN26558_c0_g1_i1_1	0.8955367	1.402317
DN26558_c0_g1_i1_2	2.085628	0
DN26562_c0_g1_i1_2	1.006639	0
DN26563_c0_g1_i1_1	3.495991	14.65276
DN26568_c0_g1_i1_1	0.6314567	1.22926
DN26568_c0_g1_i1_2	0.6490517	0.8016065
DN26573_c0_g1_i1_2	1.595682	0.6866427
DN26575_c0_g1_i1_1	0.2630555	0.1663439
DN26576_c0_g1_i1_2	5.422463	6.884232
DN26581_c0_g1_i1_1	1.048987	1.262515
DN26582_c0_g1_i1_2	0.3487245	0.3252442

DN26584_c0_g1_i1_1	2.588533	9.142985
DN26586_c0_g1_i1_1	7.27125	13.19359
DN26587_c0_g1_i1_1	0.623291	2.017801
DN26588_c0_g1_i1_2	19.95279	9.180968
DN26592_c0_g1_i1_1	1.289732	1.278442
DN26593_c0_g1_i1_1	3.176534	1.137841
DN26595_c0_g1_i1_1	10.284	128.546
DN26598_c0_g1_i1_1	0.1722539	1.460329
DN26601_c0_g1_i1_1	1.749137	1.995464
DN26603_c0_g1_i1_2	0.5646686	0.8946011
DN26606_c0_g1_i1_2	0	0.08358505
DN26607_c0_g1_i1_1	0.1247198	1.758952
DN26614_c0_g1_i1_2	3.03859	1.118403
DN26615_c0_g1_i1_1	3.469738	3.263583
DN26617_c0_g1_i1_1	2.860555	3.042731
DN26617_c0_g1_i1_2	8.46351	2.612981
DN26621_c0_g1_i1_1	3.988854	3.605589
DN26623_c0_g1_i1_1	10.78127	4.509373
DN26627_c0_g1_i1_1	2.186117	3.215618
DN26628_c0_g1_i1_2	3.320488	1.391826
DN26631_c0_g1_i1_1	3.604735	1.488049
DN26632_c0_g1_i1_2	5.272929	2.870788
DN26640_c0_g1_i1_2	1.153204	0.4977971
DN26644_c0_g1_i1_1	0.4440849	0.8241423

DN2664_c0_g1_i1_2	12.08071	9.098675
DN26652_c0_g1_i1_1	1.310411	12.11453
DN26659_c0_g1_i1_2	2.74088	0.7717615
DN26661_c0_g1_i1_1	1.735984	1.650636
DN26661_c0_g1_i1_2	1.018773	0.6306869
DN26666_c0_g1_i1_2	1.558804	0.4324256
DN26668_c0_g1_i1_1	5.307454	1.764942
DN26673_c0_g1_i1_1	0	0
DN26674_c0_g1_i1_1	0.09066795	1.991748
DN26679_c0_g1_i1_1	0.3922534	1.777431
DN26683_c0_g1_i1_2	8.9796	6.282724
DN26684_c0_g1_i1_2	1.245495	0.9982046
DN26686_c0_g1_i1_2	1.71935	1.23793
DN26689_c0_g1_i1_1	0.6270949	3.33469
DN26690_c0_g1_i1_1	0.2119678	0.5363928
DN26695_c0_g1_i1_1	6.176669	7.008071
DN26705_c0_g1_i1_1	0.3031181	0.5190583
DN26715_c0_g1_i1_1	11.34721	130.1231
DN2671_c0_g1_i1_1	2.107426	4.348675
DN26723_c0_g1_i1_1	1.910094	2.450057
DN26725_c0_g1_i1_1	2.750891	2.646732
DN26729_c0_g1_i1_2	0.6506182	0.2441396
DN26730_c0_g1_i1_2	26.80006	22.73358
DN26744_c0_g1_i1_1	0.5725428	0.7593235

DN26745_c0_g1_i1_2	8.910076	7.475214
DN26746_c0_g1_i1_1	6.400877	8.886179
DN26749_c0_g1_i1_2	95.7015	0.4288835
DN26753_c0_g1_i1_2	2.284375	3.594959
DN26755_c0_g1_i1_2	20.81976	17.8458
DN26759_c0_g1_i1_2	0.1689187	0.5639114
DN26761_c0_g1_i1_2	5.604531	5.132513
DN26762_c0_g1_i1_2	1.381926	0.1175939
DN26765_c0_g1_i1_2	2.702254	0.6030855
DN26767_c0_g1_i1_2	2.748563	1.614852
DN26768_c0_g1_i1_1	1.029187	0.7172254
DN26769_c0_g1_i1_1	0.874988	3.11214
DN26769_c0_g1_i1_2	11.18214	8.535727
DN2676_c0_g1_i1_1	2.575705	12.78863
DN26771_c0_g1_i1_1	1.671775	2.257384
DN26774_c0_g1_i1_1	13.25533	31.9237
DN2677_c0_g1_i1_2	15.71117	54.99948
DN26782_c0_g1_i1_2	3.785132	1.669507
DN26785_c0_g1_i1_2	2.313061	0.3408615
DN26787_c0_g1_i1_1	1.149265	3.346291
DN26789_c0_g1_i1_1	2.128506	2.168674
DN2678_c0_g1_i1_2	5.042059	1.411616
DN26790_c0_g1_i1_2	3.958914	1.439348
DN26791_c0_g1_i1_1	1.760246	0.860272

DN26793_c0_g1_i1_2	3.021958	0.5875929
DN26804_c0_g1_i1_1	1.808289	11.79625
DN26805_c0_g1_i1_1	0.7300294	0.2973312
DN26806_c0_g1_i1_2	9.930437	3.858005
DN26813_c0_g1_i1_1	0.7482763	0.3497527
DN26814_c0_g1_i1_1	3.577364	2.173031
DN26815_c0_g1_i1_1	1.111728	3.257375
DN26816_c0_g1_i1_1	0.4644198	0.3816199
DN26816_c0_g1_i1_2	1.672201	3.421705
DN26817_c0_g1_i1_2	2.312435	0
DN26818_c0_g1_i1_1	1.08675	1.395144
DN26819_c0_g1_i1_1	2.342571	2.876002
DN26822_c0_g1_i1_1	1.097921	2.062967
DN26823_c0_g1_i1_1	1.885244	3.110944
DN26825_c0_g1_i1_1	52.35312	151.6998
DN26827_c0_g1_i1_1	0	2.132135
DN26832_c0_g1_i1_1	1.451974	1.9068
DN26835_c0_g1_i1_1	18.21536	2.823309
DN26836_c0_g1_i1_1	0	0
DN26838_c0_g1_i1_1	1.64549	0.5392463
DN26839_c0_g1_i1_1	4.128403	4.071347
DN26840_c0_g1_i1_2	32.25488	7.984907
DN26844_c0_g1_i1_1	32.88935	23.03201
DN26847_c0_g1_i1_1	0.3326134	2.317147

DN26850_c0_g1_i1_1	1.010294	4.394612
DN26853_c0_g1_i1_1	0	0
DN26858_c0_g1_i1_1	0.6491318	1.011401
DN26858_c0_g1_i1_2	39.84746	23.90528
DN2685_c0_g1_i1_2	2.77104	1.301149
DN26861_c0_g1_i1_1	1.661629	3.780954
DN26862_c0_g1_i1_2	2.770454	1.234735
DN2686_c0_g1_i1_2	4.721505	4.108292
DN26870_c0_g1_i1_1	0.9440849	1.857702
DN26870_c0_g1_i1_2	5.413846	1.018286
DN26871_c0_g1_i1_1	4.057216	1.195252
DN26873_c0_g1_i1_1	8.48128	6.39772
DN26877_c0_g1_i1_2	8.2198	2.983529
DN26881_c0_g1_i1_1	0.8086212	3.205692
DN26882_c0_g1_i1_1	3.932077	5.414559
DN26884_c0_g1_i1_1	6.955796	14.14646
DN26887_c0_g1_i1_2	79.78194	29.53537
DN26890_c0_g1_i1_1	0	1.125905
DN26890_c0_g1_i1_2	1.073018	1.995303
DN26892_c0_g1_i1_1	4.891779	1.761201
DN26894_c0_g1_i1_2	6.1896	2.430633
DN26900_c0_g1_i1_1	0.5010855	1.087946
DN26903_c0_g1_i1_1	6.549109	2.56005
DN26903_c0_g1_i1_2	1.066681	0

DN26904_c0_g1_i1_2	3.47113	3.689303
DN26911_c0_g1_i1_2	0.1294722	0
DN26913_c0_g1_i1_1	0.7543525	2.24005
DN26913_c0_g1_i1_2	0.6985126	0.826856
DN26914_c0_g1_i1_1	0	0.3275556
DN26917_c0_g1_i1_1	0.2211196	0.8400332
DN26918_c0_g1_i1_1	1.501789	5.274034
DN2691_c0_g1_i1_1	4.484745	3.018543
DN26920_c0_g1_i1_1	7.223568	3.112111
DN26931_c0_g1_i1_1	3.073086	4.520702
DN26935_c0_g1_i1_1	1.234217	1.401913
DN26935_c0_g1_i1_2	51.09727	2.29468
DN26936_c0_g1_i1_2	96.55791	197.484
DN26937_c0_g1_i1_1	0.8792014	0.8089826
DN2693_c0_g1_i1_1	3.363461	1.469072
DN26941_c0_g1_i1_2	0.443069	4.55E-50
DN26942_c0_g1_i1_1	1.221318	2.568547
DN26948_c0_g1_i1_1	16.51087	29.24997
DN26949_c0_g1_i1_1	14.15077	5.275401
DN26951_c0_g1_i1_2	18.59852	10.98551
DN26958_c0_g1_i1_2	2.624613	0.9157476
DN2695_c0_g1_i1_1	8.495524	4.24624
DN2695_c0_g1_i1_2	2.983868	1.976269
DN26960_c0_g1_i1_1	26.52009	11.52792

DN26960_c0_g1_i1_2	15.89923	0.9527991
DN26961_c0_g1_i1_1	1.693502	1.306385
DN26962_c0_g1_i1_1	0	0.5431902
DN26967_c0_g1_i1_1	0.9127122	2.8528
DN26968_c0_g1_i1_1	1.12356	2.368528
DN26969_c0_g1_i1_1	2.867436	8.58574
DN26973_c0_g1_i1_1	0.9202093	1.092493
DN26973_c0_g1_i1_2	1.296935	0.2497848
DN26974_c0_g1_i1_2	3.41822	2.319351
DN26976_c0_g1_i1_2	11.24464	8.442714
DN26981_c0_g1_i1_1	0.9824973	1.45986
DN26981_c0_g1_i1_2	20.69257	11.73684
DN26983_c0_g1_i1_2	6.857841	1.998018
DN26984_c0_g1_i1_1	5.722067	1.714719
DN26985_c0_g1_i1_1	0.3434196	1.22657
DN26986_c0_g1_i1_1	0.61009	3.638912
DN26986_c0_g1_i1_2	2.457648	0.6046955
DN26989_c0_g1_i1_1	1.278266	2.581919
DN26991_c0_g1_i1_1	1.393321	1.613617
DN26991_c0_g1_i1_2	4.716413	1.830001
DN26992_c0_g1_i1_1	3.386245	4.783023
DN26993_c0_g1_i1_1	0.4687547	0.696291
DN26999_c0_g1_i1_1	0.7993766	0.5263298
DN26999_c0_g1_i1_2	0	0

DN27000_c0_g1_i1_1	0.5697693	2.080257
DN27003_c0_g1_i1_1	2.361134	2.199829
DN27004_c0_g1_i1_1	0.4184551	0.5176448
DN27004_c0_g1_i1_2	25.97283	46.24136
DN27006_c0_g1_i1_2	4.710003	2.625948
DN27010_c0_g1_i1_1	11.64661	16.90887
DN27013_c0_g1_i1_1	1.685578	0
DN27015_c0_g1_i1_1	1.336179	1.599618
DN27019_c0_g1_i1_2	2.255214	0.4896057
DN27023_c0_g1_i1_1	3.55899	2.410453
DN27024_c0_g1_i1_1	0.6637778	3.900043
DN27025_c0_g1_i1_2	1.111416	1.471144
DN27041_c0_g1_i1_2	2.689632	2.239379
DN27042_c0_g1_i1_2	7.795721	3.605482
DN27046_c0_g1_i1_2	1.278167	0.748679
DN27048_c0_g1_i1_1	0.1995221	0.5545274
DN27048_c0_g1_i1_2	1.825898	0
DN27049_c0_g1_i1_1	2.180728	4.708259
DN27053_c0_g1_i1_2	3.152484	6.163251
DN27055_c0_g1_i1_1	0.5933452	1.115151
DN27058_c0_g1_i1_2	1.90597	1.722442
DN27059_c0_g1_i1_1	2.344832	2.488267
DN27060_c0_g1_i1_2	13.35999	8.302956
DN27063_c0_g1_i1_2	1.671064	0.3838874

DN27064_c0_g1_i1_1	1.77966	1.259046
DN27065_c0_g1_i1_2	1.103041	0.6634692
DN27070_c0_g1_i1_1	33.02445	29.21575
DN27075_c0_g1_i1_1	0.556082	1.372115
DN27076_c0_g1_i1_2	1.99188	0.9469015
DN27078_c0_g1_i1_1	4.626988	4.989159
DN2707_c0_g1_i1_1	0.4183569	0.2337864
DN2707_c0_g2_i1_1	0.5980194	0.676118
DN27086_c0_g1_i1_1	0.1655158	0.8069601
DN27086_c0_g1_i1_2	4.37299	0.4938925
DN27088_c0_g1_i1_1	3.391813	1.982754
DN2708_c0_g1_i1_2	0.2681496	0.2061758
DN27093_c0_g1_i1_1	0.8024037	2.009255
DN27094_c0_g1_i1_1	2.277997	3.552616
DN27094_c0_g1_i1_2	18.81176	9.196437
DN27096_c0_g1_i1_2	1.30774	0.4292355
DN2709_c0_g1_i1_1	0.4556601	0.5064081
DN2709_c0_g2_i1_1	1.963115	1.414793
DN27104_c0_g1_i1_1	1.333197	2.166101
DN27105_c0_g1_i1_2	0.2504628	0.6570848
DN27110_c0_g1_i1_1	0.9667892	0.5649628
DN27111_c0_g1_i1_1	10.58355	3.147384
DN27116_c0_g1_i1_1	0	0.3302445
DN27118_c0_g1_i1_1	4.440773	1.762146

DN2711_c0_g1_i1_2	9.558739	5.118582
DN27123_c0_g1_i1_2	0.9834278	0.9156523
DN27127_c0_g1_i1_2	1.542914	0.4029633
DN27128_c0_g1_i1_1	0.9344734	1.309695
DN27129_c0_g1_i1_1	1.272729	0
DN27130_c0_g1_i1_1	1.302017	1.552553
DN27132_c0_g1_i1_1	11.32608	8.568944
DN27133_c0_g1_i1_1	4.763208	35.17623
DN27137_c0_g1_i1_2	4.573032	0.7820924
DN27139_c0_g1_i1_1	0.5533995	2.236824
DN2713_c0_g1_i1_1	2.943766	5.933579
DN27141_c0_g1_i1_1	1.63968	3.077278
DN27144_c0_g1_i1_1	0.6636308	0.7168797
DN27145_c0_g1_i1_1	0.0496946	3.14342
DN27148_c0_g1_i1_2	3.13055	1.487543
DN27153_c0_g1_i1_1	11.83481	7.853333
DN27155_c0_g1_i1_1	8.951627	10.08987
DN27158_c0_g1_i1_2	0	0
DN27159_c0_g1_i1_1	0.1605004	11.60912
DN27162_c0_g1_i1_1	1.516507	14.03777
DN27163_c0_g1_i1_1	0.3004819	0.3250332
DN27164_c0_g1_i1_2	4.743675	1.072038
DN27165_c0_g1_i1_1	0.686319	2.783005
DN27165_c0_g1_i1_2	1.396313	0.2474489

DN27166_c0_g1_i1_2	8.047824	6.134192
DN27167_c0_g1_i1_1	1.160088	0.8559354
DN27167_c0_g1_i1_2	0.7356837	3.651905
DN27168_c0_g1_i1_2	1.913465	1.53585
DN27170_c0_g1_i1_1	1.252351	1.636056
DN27174_c0_g1_i1_2	3.985305	1.06056
DN27175_c0_g1_i1_1	0.7536178	14.52082
DN27175_c0_g1_i1_2	3.774761	0.4708761
DN27178_c0_g1_i1_1	9.369472	7.895154
DN27178_c0_g1_i1_2	7.632564	4.598412
DN27179_c0_g1_i1_1	9.020079	28.03781
DN27183_c0_g1_i1_1	0.7167605	1.771833
DN27188_c0_g1_i1_1	0.2646703	1.273238
DN27189_c0_g1_i1_1	0.1999405	0.4385612
DN27189_c0_g1_i1_2	1.820107	1.324859
DN2718_c0_g1_i1_1	0.3934787	1.659959
DN27193_c0_g1_i1_2	0.3503115	1.37413
DN27197_c0_g1_i1_2	3.91317	4.094008
DN27199_c0_g1_i1_1	0.1640548	2.369211
DN27199_c0_g1_i1_2	1.386357	0.3365997
DN271_c0_g1_i1_1	2.108784	4.76437
DN27200_c0_g1_i1_1	0.8552844	2.210132
DN27201_c0_g1_i1_2	2.667832	0.5303409
DN27204_c0_g1_i1_1	0.8039591	0.5304123

DN27209_c0_g1_i1_1	1.222807	1.31677
DN27213_c0_g1_i1_1	10.10779	6.284931
DN27215_c0_g1_i1_1	0.9998054	0.520483
DN27219_c0_g1_i1_1	0	0
DN27221_c0_g1_i1_2	9.111046	8.104923
DN27223_c0_g1_i1_1	4.028368	9.129529
DN27225_c0_g1_i1_2	3.442295	1.857205
DN27226_c0_g1_i1_1	0.5394262	2.055163
DN27229_c0_g1_i1_1	0.1939597	0.9754979
DN2722_c0_g1_i1_2	0	0
DN2722_c0_g1_i2_1	0.4176277	3.409114
DN27231_c0_g1_i1_1	0.4060471	5.106325
DN27232_c0_g1_i1_2	1.644943	2.474097
DN27235_c0_g1_i1_1	1.156108	1.188311
DN27238_c0_g1_i1_1	0.4778017	1.238397
DN27239_c0_g1_i1_1	0.2420331	1.651376
DN2723_c0_g1_i1_1	3.915702	0.9770772
DN27243_c0_g1_i1_1	4.419435	7.718339
DN27249_c0_g1_i1_1	1.698467	6.958439
DN27254_c0_g1_i1_1	0.9666288	0.4263261
DN27255_c0_g1_i1_1	1.195356	0.386728
DN27258_c0_g1_i1_2	3.108549	1.779633
DN27259_c0_g1_i1_1	0.302495	1.831541
DN27261_c0_g1_i1_1	1.424519	5.703932

DN27263_c0_g1_i1_1	2.836366	1.260698
DN27264_c0_g1_i1_1	6.551972	4.327824
DN27266_c0_g1_i1_1	1.029464	17.32298
DN27268_c0_g1_i1_1	0	0
DN27269_c0_g1_i1_1	1.82453	2.202029
DN27272_c0_g1_i1_1	0.4853173	2.147742
DN27277_c0_g1_i1_1	1.26154	0.7592235
DN27282_c0_g1_i1_1	3.009421	2.972375
DN27292_c0_g1_i1_1	0.9478424	0.8496007
DN2729_c0_g1_i1_1	8.145559	10.65021
DN27301_c0_g1_i1_1	1.424283	0.8530901
DN27301_c0_g1_i1_2	7.978404	24.53603
DN27302_c0_g1_i1_1	3.131573	2.074553
DN27306_c0_g1_i1_2	109.6265	57.47757
DN27308_c0_g1_i1_1	0.1139656	2.025706
DN27313_c0_g1_i1_1	0.4650213	0.2923442
DN27315_c0_g1_i1_1	1.119687	2.35693
DN27316_c0_g1_i1_1	14.57877	10.25575
DN27321_c0_g1_i1_2	13.14841	9.576979
DN27323_c0_g1_i1_1	38.47248	9.562872
DN27325_c0_g1_i1_1	0.571945	3.072623
DN27326_c0_g1_i1_2	1.292659	1.434034
DN27328_c0_g1_i1_2	5.136241	2.727873
DN27329_c0_g1_i1_1	1.820989	1.181669

DN27330_c0_g1_i1_2	0.2769687	2.089698
DN27331_c0_g1_i1_2	0.8696073	0.4959633
DN27332_c0_g1_i1_1	6.568811	18.30915
DN27333_c0_g1_i1_1	0.5780597	2.398073
DN27336_c0_g1_i1_1	0.7279472	1.630629
DN27342_c0_g1_i1_2	1.422973	0.6340383
DN27344_c0_g1_i1_2	19.35259	6.421313
DN27346_c0_g1_i1_1	0.2421117	0.671942
DN27348_c0_g1_i1_1	0.3822971	0.6271581
DN27352_c0_g1_i1_2	4.659515	2.472403
DN27353_c0_g1_i1_2	1.040983	0.3910188
DN27354_c0_g1_i1_1	0.5852661	1.001126
DN27354_c0_g1_i1_2	0.255216	1.914244
DN27355_c0_g1_i1_1	5.502055	3.8721
DN27358_c0_g1_i1_1	14.86425	15.98811
DN27362_c0_g1_i1_1	0.3490287	0.6449292
DN27364_c0_g1_i1_1	40.72774	30.94279
DN27367_c0_g1_i1_1	0.1612903	1.25219
DN27370_c0_g1_i1_1	1.412775	1.409319
DN27371_c0_g1_i1_1	0.4180953	1.803467
DN27371_c0_g1_i1_2	0.5792875	0.5425475
DN27372_c0_g1_i1_1	0.2182051	0.2950847
DN27372_c0_g1_i1_2	1.076323	0.3255542
DN27376_c0_g1_i1_1	2.080314	1.560798

DN27377_c0_g1_i1_1	3.732392	2.448949
DN27379_c0_g1_i1_1	2.952131	11.45092
DN27381_c0_g1_i1_2	0	0
DN27386_c0_g1_i1_1	4.429332	2.585548
DN27387_c0_g1_i1_1	27.20149	25.94345
DN27387_c0_g1_i1_2	0.8802394	0.8202925
DN27388_c0_g1_i1_1	2.812857	4.15E-15
DN27392_c0_g1_i1_2	0.8013464	1.012578
DN27393_c0_g1_i1_2	10.15235	14.25625
DN27394_c0_g1_i1_2	1.630139	0
DN27401_c0_g1_i1_2	0.7109259	0.5524461
DN27402_c0_g1_i1_2	3.701935	1.416067
DN27406_c0_g1_i1_1	1.389791	1.329644
DN27407_c0_g1_i1_1	0.4367979	2.237749
DN2740_c0_g1_i1_1	1.226079	3.738399
DN2740_c0_g1_i1_2	0.5305036	2.089244
DN27415_c0_g1_i1_2	1.573741	0
DN27418_c0_g1_i1_1	1.636963	1.130598
DN27421_c0_g1_i1_1	1.146152	0.643163
DN27422_c0_g1_i1_2	0.1434915	0
DN27426_c0_g1_i1_1	0	0.4599751
DN27427_c0_g1_i1_1	0.6579156	0.6326005
DN27433_c0_g1_i1_1	0.3873909	2.286721
DN27439_c0_g1_i1_1	0.512976	0.9377272

DN27440_c0_g1_i1_1	0.7492035	2.011333
DN27445_c0_g1_i1_1	0	2.040394
DN27447_c0_g1_i1_1	0.9172806	0.3619286
DN27452_c0_g1_i1_1	2.522398	4.29473
DN27453_c0_g1_i1_1	0.625857	1.349357
DN27454_c0_g1_i1_1	0.2304292	0.8199248
DN27455_c0_g1_i1_1	0	1.859263
DN27457_c0_g1_i1_1	2.617357	2.40661
DN27459_c0_g1_i1_1	3.894049	2.643701
DN27461_c0_g1_i1_1	18.85178	7.810515
DN27464_c0_g1_i1_1	1.171085	1.545886
DN27466_c0_g1_i1_1	0.2258574	11.82551
DN27468_c0_g1_i1_1	5.110732	8.884579
DN27468_c0_g1_i1_2	2.623923	4.392901
DN27469_c0_g1_i1_1	0.7313466	0.9038019
DN27471_c0_g1_i1_1	0.1633832	2.422582
DN27471_c0_g1_i1_2	0.3762302	1.01E-21
DN27477_c0_g1_i1_2	2.642802	1.547786
DN27478_c0_g1_i1_1	0.741916	0.000740493
DN27481_c0_g1_i1_1	6.289153	2.094159
DN27482_c0_g1_i1_1	13.31065	8.001249
DN27483_c0_g1_i1_1	2.533133	2.351748
DN27485_c0_g1_i1_1	0.8924758	1.950532
DN27488_c0_g1_i1_2	77.35349	51.18207

DN27494_c0_g1_i1_1	23.0791	16.29216
DN27494_c0_g1_i1_2	6.062337	5.413372
DN27496_c0_g1_i1_2	1.327483	1.308683
DN27497_c0_g1_i1_1	3.588055	2.799779
DN27498_c0_g1_i1_1	15.17171	6.733669
DN2749_c0_g1_i1_2	0.88405	0
DN27502_c0_g1_i1_1	0.06328642	0.4228475
DN27504_c0_g1_i1_1	3.454263	1.932009
DN27504_c0_g1_i1_2	87.384	0.03891095
DN27505_c0_g1_i1_2	4.373779	2.731939
DN27506_c0_g1_i1_1	1.406745	2.314727
DN27507_c0_g1_i1_2	1.874743	1.981596
DN2750_c0_g1_i1_1	2.690919	4.758687
DN27510_c0_g1_i1_1	0	2.780711
DN27512_c0_g1_i1_1	0.5415525	1.763162
DN27521_c0_g1_i1_1	0.1010057	0.9931287
DN27524_c0_g1_i1_2	1.74871	1.275463
DN27525_c0_g1_i1_1	2.191238	5.322574
DN27530_c0_g1_i1_1	0.8485845	0.221293
DN27530_c0_g1_i1_2	4.407293	2.581113
DN27532_c0_g1_i1_2	2.630641	0.7405197
DN27536_c0_g1_i1_1	1.558589	2.137534
DN27537_c0_g1_i1_2	2.115343	0.2464362
DN2753_c0_g1_i1_1	0.5506962	0.3969891

DN27543_c0_g1_i1_1	1.191489	2.620018
DN27548_c0_g1_i1_2	0.6970776	2.086997
DN27549_c0_g1_i1_2	2.160836	0.7227496
DN27550_c0_g1_i1_1	1.238483	0.2472892
DN27551_c0_g1_i1_2	0.8925619	0
DN27552_c0_g1_i1_1	0.2865376	0.9659729
DN27556_c0_g1_i1_1	1.137935	2.893971
DN27557_c0_g1_i1_1	1.403173	0.5064748
DN27558_c0_g1_i1_1	410.2977	945.0608
DN27558_c0_g1_i1_2	2.28257	0.8099896
DN2755_c0_g1_i1_2	1.408368	0.9719516
DN27562_c0_g1_i1_1	2.246222	3.077243
DN27564_c0_g1_i1_1	0.2292428	2.852627
DN27565_c0_g1_i1_1	0.6425212	0.4152937
DN27570_c0_g1_i1_2	1.719247	1.545458
DN27572_c0_g1_i1_2	0.4677922	0.2699181
DN27574_c0_g1_i1_1	1.165495	2.534447
DN27576_c0_g1_i1_1	10.01631	8.188729
DN27578_c0_g1_i1_1	1.057479	1.688549
DN27581_c0_g1_i1_2	1.651893	0.3812196
DN27583_c0_g1_i1_2	0.7238005	0.9394258
DN27587_c0_g1_i1_2	0	0
DN27588_c0_g1_i1_2	1.158417	0.3798765
DN27589_c0_g1_i1_1	9.95E-31	0

DN27591_c0_g1_i1_1	0.6576951	0.583406
DN27594_c0_g1_i1_2	1.25771	0.5615219
DN27596_c0_g1_i1_1	1.253832	2.41887
DN2759_c0_g1_i1_1	4.039078	13.67446
DN275_c0_g1_i1_1	1.471605	1.605975
DN27600_c0_g1_i1_1	1.170937	1.908528
DN27602_c0_g1_i1_1	1.429619	0.4373655
DN27603_c0_g1_i1_2	6.572024	3.022886
DN27605_c0_g1_i1_1	1.250979	2.037685
DN27608_c0_g1_i1_2	0.9421689	0.72862
DN27609_c0_g1_i1_2	8.364654	2.216182
DN27611_c0_g1_i1_1	1.132363	0.4813309
DN27614_c0_g1_i1_2	1.823777	0.4876189
DN27615_c0_g1_i1_1	0.332055	2.923648
DN27618_c0_g1_i1_2	2.722562	0.8616936
DN27619_c0_g1_i1_2	3.800397	0.2406098
DN27622_c0_g1_i1_1	1.232525	1.413644
DN27622_c0_g1_i1_2	1.156654	0.2939417
DN27629_c0_g1_i1_2	0.7159513	0.4722139
DN27630_c0_g1_i1_2	15.94265	9.917241
DN27634_c0_g1_i1_2	2.166131	1.749645
DN27637_c0_g1_i1_1	3.078214	2.025116
DN27640_c0_g1_i1_1	0.3768271	0
DN27641_c0_g1_i1_1	3.036476	1.986367

DN27644_c0_g1_i1_2	0.777807	0.4400835
DN27646_c0_g1_i1_2	1.270931	0.9931264
DN27652_c0_g1_i1_1	10.73023	7.926082
DN27653_c0_g1_i1_1	0.5127728	1.178955
DN27655_c0_g1_i1_2	0	0
DN27656_c0_g1_i1_1	1.343104	3.254085
DN27658_c0_g1_i1_2	2.948075	0.6433937
DN27660_c0_g1_i1_1	63.0832	44.56371
DN27662_c0_g1_i1_2	0.9967069	0.5799631
DN27664_c0_g1_i1_1	1.087174	4.400981
DN27667_c0_g1_i1_1	1.848966	1.416521
DN2766_c0_g1_i1_2	0.6943939	0.1998817
DN27672_c0_g1_i1_1	0	0
DN27675_c0_g1_i1_2	9.458534	3.551924
DN27678_c0_g1_i1_1	0.8892715	0.6954682
DN27681_c0_g1_i1_1	0.2397788	1.357704
DN27683_c0_g1_i1_1	0.5097969	2.320512
DN27684_c0_g1_i1_1	0	2.524186
DN27689_c0_g1_i1_1	2.03679	0.7362968
DN2768_c0_g1_i1_1	3.571988	6.149858
DN27690_c0_g1_i1_1	59.76949	47.42317
DN27692_c0_g1_i1_1	0.5343196	0.6890072
DN27693_c0_g1_i1_1	0.532511	0
DN27695_c0_g1_i1_1	10.74788	9.302483

DN27696_c0_g1_i1_1	0.550782	1.265732
DN27696_c0_g1_i1_2	47.46903	39.85053
DN27697_c0_g1_i1_2	1.473575	0.4888007
DN27699_c0_g1_i1_1	0.8232383	1.077287
DN2769_c0_g1_i1_1	0.8275991	1.302935
DN27701_c0_g1_i1_2	19.74707	19.32951
DN27702_c0_g1_i1_1	1.229964	3.005066
DN27702_c0_g1_i1_2	1.662308	1.434664
DN27705_c0_g1_i1_2	2.665	1.049102
DN27707_c0_g1_i1_1	0.1585006	2.162753
DN27707_c0_g1_i1_2	0.9066511	0.3242233
DN27712_c0_g1_i1_1	0.6454297	1.731598
DN27713_c0_g1_i1_1	3.416094	1.298528
DN27715_c0_g1_i1_1	1.686705	1.189332
DN27716_c0_g1_i1_2	3.536238	0.9277199
DN27718_c0_g1_i1_1	0.4112835	1.569997
DN27718_c0_g1_i1_2	3.264395	0.5801694
DN27727_c0_g1_i1_1	0.5353438	0.5019242
DN27727_c0_g1_i1_2	1.434732	0.4638962
DN27729_c0_g1_i1_2	1.78575	0.351149
DN27730_c0_g1_i1_1	1.596741	1.899106
DN27737_c0_g1_i1_2	2.281589	2.655518
DN27740_c0_g1_i1_2	4.49916	2.755778
DN27743_c0_g1_i1_2	2.800834	1.639061

DN27749_c0_g1_i1_1	0.6289569	3.131815
DN2774_c0_g1_i1_2	0	0
DN27751_c0_g1_i1_1	0.3275808	2.413603
DN27752_c0_g1_i1_1	0.1578178	1.647886
DN27753_c0_g1_i1_2	0	0
DN27755_c0_g1_i1_1	0.3153724	0.01664305
DN27756_c0_g1_i1_1	0.8684289	2.898626
DN27757_c0_g1_i1_2	1.452813	2.693719
DN27758_c0_g1_i1_1	1.372639	0.6918963
DN27759_c0_g1_i1_1	1.604536	1.115221
DN27761_c0_g1_i1_2	37.43444	54.8256
DN27763_c0_g1_i1_1	1.360071	2.848293
DN27765_c0_g1_i1_1	0.4008136	0.6092861
DN27767_c0_g1_i1_1	20.07221	12.84306
DN27769_c0_g1_i1_2	2.802513	0.3651507
DN27771_c0_g1_i1_1	0.5590726	0.2868745
DN27772_c0_g1_i1_1	13.12168	11.49034
DN27773_c0_g1_i1_1	7.702727	5.039631
DN27774_c0_g1_i1_1	4.124834	1.323319
DN27776_c0_g1_i1_1	0	3.84914
DN27777_c0_g1_i1_1	0.9262996	0.1257406
DN27778_c0_g1_i1_1	1.572091	2.762987
DN2777_c0_g1_i1_1	1.708474	2.528778
DN27783_c0_g1_i1_1	0.3831309	0.7741366

DN27783_c0_g1_i1_2	0	0.2745806
DN27784_c0_g1_i1_2	2.408363	1.678945
DN27786_c0_g1_i1_1	0.6895902	2.873558
DN27787_c0_g1_i1_1	0.6979087	2.45E-30
DN27789_c0_g1_i1_1	1.038812	1.638572
DN27790_c0_g1_i1_1	433.4557	148.3377
DN27791_c0_g1_i1_1	0.562044	0.4160996
DN27797_c0_g1_i1_1	0.5377278	1.356945
DN27799_c0_g1_i1_1	0.5574991	1.115366
DN27799_c0_g1_i1_2	1.12286	1.40982
DN27802_c0_g1_i1_1	24.03133	58.41945
DN27802_c0_g1_i1_2	0	0
DN27807_c0_g1_i1_1	3.12198	1.299635
DN27808_c0_g1_i1_1	0.8888381	0.4751752
DN27809_c0_g1_i1_1	1.721125	1.758416
DN27809_c0_g1_i1_2	0.8504305	0.3886289
DN27811_c0_g1_i1_1	0.8644023	2.046135
DN27811_c0_g1_i1_2	2.18122	2.371311
DN27813_c0_g1_i1_2	1.99739	1.670668
DN27814_c0_g1_i1_1	0.3363755	0
DN27816_c0_g1_i1_1	1.601602	0.7945126
DN27819_c0_g1_i1_1	0.7519651	1.842513
DN27819_c0_g1_i1_2	4.92373	0.5822582
DN27824_c0_g1_i1_1	5.036858	2.512736

DN2782_c0_g1_i1_2	4.071929	2.307156
DN27831_c0_g1_i1_2	949.4851	0.7306205
DN27833_c0_g1_i1_1	1.56E-31	0.5421269
DN27835_c0_g1_i1_2	1.964688	2.242682
DN27838_c0_g1_i1_1	4.088468	5.125281
DN27839_c0_g1_i1_1	1.752341	5.976525
DN27843_c0_g1_i1_2	0.9626331	0.6366791
DN27844_c0_g1_i1_1	2.534572	7.367783
DN27851_c0_g1_i1_1	0.9917059	3.248732
DN27853_c0_g1_i1_2	0	0
DN27855_c0_g1_i1_1	0.2419125	1.051622
DN27858_c0_g1_i1_1	0	0
DN27859_c0_g1_i1_1	0.9428522	0
DN27860_c0_g1_i1_1	0	0.3026084
DN27864_c0_g1_i1_1	0.9026186	1.722404
DN27865_c0_g1_i1_1	0	0.6206665
DN27865_c0_g1_i1_2	3.509113	1.585832
DN27870_c0_g1_i1_2	0.9177918	0.9639687
DN27871_c0_g1_i1_2	4.325625	1.659311
DN27872_c0_g1_i1_1	42.16588	37.85584
DN27873_c0_g1_i1_1	0.6193574	2.101568
DN27874_c0_g1_i1_1	1.386174	2.245007
DN27876_c0_g1_i1_2	9.820387	22.0348
DN27880_c0_g1_i1_1	0.3215015	0.4878878

DN27884_c0_g1_i1_1	1.797326	10.82915
DN27891_c0_g1_i1_2	1.050875	0.4723245
DN27901_c0_g1_i1_2	2.066892	0.97575
DN27902_c0_g1_i1_2	5.12161	0.4394058
DN27903_c0_g1_i1_2	3.414483	1.400633
DN27910_c0_g1_i1_2	1.067766	0.6157274
DN27916_c0_g1_i1_1	0.330357	1.848604
DN27916_c0_g1_i1_2	0.750315	0
DN27918_c0_g1_i1_1	0.3600966	0.05220142
DN27921_c0_g1_i1_2	14.87248	16.49668
DN27922_c0_g1_i1_2	2.828941	0.4681831
DN27924_c0_g1_i1_2	2.269707	0.319481
DN27925_c0_g1_i1_2	2.065781	1.132914
DN27927_c0_g1_i1_1	0.5977136	1.183432
DN27930_c0_g1_i1_2	2.096119	0
DN27931_c0_g1_i1_1	0.5826603	1.42963
DN27934_c0_g1_i1_1	0.8260117	0.4860734
DN27936_c0_g1_i1_2	1.900126	2.801993
DN27940_c0_g1_i1_2	8.238655	5.993033
DN27946_c0_g1_i1_1	3.366031	0.856154
DN27954_c0_g1_i1_2	2.331547	1.946805
DN27955_c0_g1_i1_2	3.752234	0.8273239
DN27964_c0_g1_i1_1	11.17976	5.367494
DN27970_c0_g1_i1_1	0.8651084	0.7038661

DN27973_c0_g1_i1_2	0	0
DN27977_c0_g1_i1_2	2.128728	1.006248
DN27978_c0_g1_i1_2	1.01816	9.84E-31
DN27979_c0_g1_i1_1	1.391664	0.5330808
DN27982_c0_g1_i1_2	4.26671	1.294146
DN27984_c0_g1_i1_2	3.032684	1.054512
DN27985_c0_g1_i1_1	0.3877535	1.761194
DN27987_c0_g1_i1_2	0.9150504	0.3746693
DN27989_c0_g1_i1_2	0.8838364	0
DN27992_c0_g1_i1_2	1.758704	2.368297
DN27996_c0_g1_i1_1	1.393652	3.977921
DN27997_c0_g1_i1_1	0.3424355	5.372646
DN279_c0_g1_i1_2	16.54526	0
DN28000_c0_g1_i1_2	1.848819	0.9002787
DN28007_c0_g1_i1_1	24.38874	10.53659
DN28013_c0_g1_i1_1	3.164856	4.889739
DN28013_c0_g1_i1_2	2.867835	0.8649638
DN28016_c0_g1_i1_2	4.800394	0.7629993
DN28017_c0_g1_i1_2	0.2607328	0.9094549
DN28020_c0_g1_i1_1	23.92666	23.54361
DN28024_c0_g1_i1_1	0.6088636	0.8873674
DN28025_c0_g1_i1_2	24.34969	12.8153
DN2802_c0_g1_i2_1	1.877982	4.630335
DN28032_c0_g1_i1_1	0.9313286	0.8438529

DN28036_c0_g1_i1_1	11.88559	54.03985
DN28037_c0_g1_i1_2	4.814161	1.881499
DN28038_c0_g1_i1_1	9.463191	3.733628
DN28045_c0_g1_i1_2	4.940815	1.139823
DN28046_c0_g1_i1_2	0.60844	0.0493935
DN28048_c0_g1_i1_2	0.9874726	1.082054
DN28048_c0_g1_i2_1	1.770024	7.962027
DN28058_c0_g1_i1_1	2.698807	2.409434
DN28060_c0_g1_i1_2	0.7777624	0
DN28061_c0_g1_i1_1	1.357169	2.112699
DN28065_c0_g1_i1_2	4.128763	2.70367
DN2806_c0_g1_i1_1	0.755358	5.839741
DN28072_c0_g1_i1_2	1.740976	0
DN28074_c0_g1_i1_2	5.478691	3.537711
DN2807_c0_g1_i1_2	0.6911883	0.3851706
DN28080_c0_g1_i1_2	0.6435586	0.04470298
DN28084_c0_g1_i1_1	56.08881	38.55651
DN28091_c0_g1_i1_1	3.224976	5.428163
DN28096_c0_g1_i1_1	0	0.8034653
DN28097_c0_g1_i1_1	2.511256	1.739416
DN280_c0_g1_i1_1	78.89828	87.3775
DN28101_c0_g1_i1_1	0.3380075	1.487627
DN28104_c0_g1_i1_2	0	0.8756798
DN28107_c0_g1_i1_1	1.671716	1.062063

DN28110_c0_g1_i1_1	0.8615281	0.6558297
DN28110_c0_g1_i1_2	0	3.37E-31
DN28113_c0_g1_i1_1	3.029973	7.534186
DN28116_c0_g1_i1_1	1.572769	5.744335
DN28122_c0_g1_i1_2	1.241018	0.089792
DN28124_c0_g1_i1_1	1.267965	1.737797
DN28124_c0_g1_i1_2	2.586497	3.050957
DN28131_c0_g1_i1_2	3.580907	5.263596
DN28137_c0_g1_i1_1	263.0034	263.2604
DN28146_c0_g1_i1_1	0.2227207	1.684202
DN28146_c0_g1_i1_2	1.096318	0.7737595
DN28150_c0_g1_i1_2	3.355327	3.48802
DN28159_c0_g1_i1_2	2.353927	0.5534113
DN28170_c0_g1_i1_1	0.1689194	1.885904
DN28173_c0_g1_i1_2	1.558537	1.110864
DN28179_c0_g1_i1_2	7.265939	19.15579
DN28180_c0_g1_i1_1	3.059845	2.708657
DN28185_c0_g1_i1_2	2.129546	0.5619974
DN28187_c0_g1_i1_2	2.762712	0
DN2818_c0_g1_i1_2	0	0
DN28192_c0_g1_i1_1	1.791579	4.315158
DN28192_c0_g1_i1_2	2.679765	2.852996
DN28194_c0_g1_i1_2	2.213025	2.383303
DN28195_c0_g1_i1_2	0.1898485	0

DN28196_c0_g1_i1_1	9.184351	9.867679
DN28198_c0_g1_i1_2	3.036747	0.9739282
DN2819_c0_g1_i1_1	1.582883	1.706374
DN281_c0_g2_i1_2	0	0
DN28205_c0_g1_i1_2	0.9511108	0.6422931
DN28207_c0_g1_i1_1	3.677839	6.333868
DN28212_c0_g1_i1_2	0.5175006	0.4607289
DN28217_c0_g1_i1_2	1.078224	1.27991
DN28219_c0_g1_i1_2	5.06E-25	1.585332
DN28221_c0_g1_i1_1	0	1.866636
DN28222_c0_g1_i1_2	2.519389	5.814836
DN28223_c0_g1_i1_1	2.213331	5.367713
DN28229_c0_g1_i1_2	5.355835	6.004161
DN28232_c0_g1_i1_1	1.544306	0.6178156
DN28233_c0_g1_i1_1	5.91029	4.445682
DN28234_c0_g1_i1_1	8.867577	6.025096
DN28234_c0_g1_i1_2	0.8624029	0.9126565
DN28236_c0_g1_i1_2	2.333709	1.256231
DN28239_c0_g1_i1_1	0.3012176	0.3068924
DN28243_c0_g1_i1_2	3.686297	1.440469
DN28246_c0_g1_i1_2	0.5672523	0
DN28247_c0_g1_i1_1	1.022908	1.307733
DN28248_c0_g1_i1_1	0.787813	0.9382654
DN28252_c0_g1_i1_2	0.6998929	0.1553612

DN28253_c0_g1_i1_2	0.4018279	0.1173715
DN28254_c0_g1_i1_1	0.1316703	0.4161251
DN28257_c0_g1_i1_1	0.0875431	4.33683
DN28260_c0_g1_i1_2	2.34162	0.1948116
DN28261_c0_g1_i1_1	0.3640878	2.59469
DN28262_c0_g1_i1_1	0	0.943618
DN28265_c0_g1_i1_2	4.252806	4.813114
DN28269_c0_g1_i1_1	0.3852703	1.972291
DN2826_c0_g1_i1_1	0.05861576	4.08884
DN28270_c0_g1_i1_2	2.479367	2.056935
DN28271_c0_g1_i1_1	3.199792	8.010642
DN28275_c0_g1_i1_2	2.305015	1.681298
DN28276_c0_g1_i1_1	3.334819	15.06681
DN28277_c0_g1_i1_1	0	1.70774
DN28278_c0_g1_i1_1	0.5964694	1.116772
DN28279_c0_g1_i1_1	0.9392243	1.211679
DN28279_c0_g1_i1_2	0	0
DN28283_c0_g1_i1_1	0.8577782	2.944431
DN28283_c0_g1_i1_2	1.768122	0.8356403
DN28284_c0_g1_i1_1	2.051947	2.355883
DN28286_c0_g1_i1_1	5.916482	4.277042
DN28286_c0_g1_i1_2	0	0
DN28287_c0_g1_i1_2	1.646062	0.9771302
DN28288_c0_g1_i1_1	8.073144	11.05751

DN28288_c0_g1_i1_2	0	0.007434044
DN2828_c0_g1_i1_1	1.277264	2.298001
DN28291_c0_g1_i1_1	0.6065281	2.057683
DN28291_c0_g1_i1_2	23.96748	7.742259
DN28292_c0_g1_i1_1	1.158832	1.341172
DN28294_c0_g1_i1_2	11.65	3.470645
DN28297_c0_g1_i1_1	1.02094	0.526441
DN28301_c0_g1_i1_2	2.816177	2.772515
DN28302_c0_g1_i1_2	2.872328	2.146187
DN28310_c0_g1_i1_2	11.20804	6.568757
DN28313_c0_g1_i1_2	0.4453642	0.1338652
DN28315_c0_g1_i1_1	0.6861319	0.108204
DN28318_c0_g1_i1_2	9.864804	7.234528
DN28320_c0_g1_i1_2	0.9360241	0.5847156
DN28323_c0_g1_i1_1	1.96686	1.174216
DN28329_c0_g1_i1_2	0	0
DN2832_c0_g1_i1_1	2.053395	1.443853
DN28330_c0_g1_i1_1	1.777396	1.222524
DN28333_c0_g1_i1_1	0.2943308	1.888583
DN28333_c0_g1_i1_2	8.651199	44.97173
DN28337_c0_g1_i1_2	1.119849	0
DN28338_c0_g1_i1_1	1.101188	1.445857
DN28340_c0_g1_i1_2	4.201699	0.6122983
DN28341_c0_g1_i1_2	0.8540884	1.523839

DN28342_c0_g1_i1_2	0	0
DN28343_c0_g1_i1_2	1.000822	0.1905031
DN28344_c0_g1_i1_2	15.82583	1.998263
DN28346_c0_g1_i1_1	0	1.026704
DN28348_c0_g1_i1_1	0.1058537	2.548735
DN28353_c0_g1_i1_2	1.334506	1.838931
DN28356_c0_g1_i1_1	0.6852194	0.7203801
DN28358_c0_g1_i1_2	0	0
DN28361_c0_g1_i1_2	0.6140259	0.1509182
DN28362_c0_g1_i1_1	1.354151	1.572267
DN28362_c0_g1_i1_2	3.862589	1.134077
DN28363_c0_g1_i1_1	0.2542056	4.198706
DN28364_c0_g1_i1_2	3.533731	3.496282
DN28368_c0_g1_i1_2	12.37294	8.705906
DN28369_c0_g1_i1_1	1.116214	2.115751
DN2836_c0_g1_i1_1	0	2.912325
DN28374_c0_g1_i1_1	1.665197	2.007145
DN28375_c0_g1_i1_2	6.204769	1.590021
DN28378_c0_g1_i1_1	1.303455	2.781865
DN28378_c0_g1_i1_2	1.453151	0.6328498
DN2837_c0_g1_i1_2	1.238208	0.8389957
DN28383_c0_g1_i1_1	0	12.28391
DN28388_c0_g1_i1_1	2.305735	5.102051
DN28389_c0_g1_i1_2	0	0

DN2838_c0_g1_i1_1	2.281138	1.778033
DN28392_c0_g1_i1_1	1.107495	1.520903
DN28393_c0_g1_i1_2	0	0.3025065
DN28394_c0_g1_i1_2	0.8540955	0.3286596
DN28396_c0_g1_i1_1	6.704235	2.45993
DN28399_c0_g1_i1_2	0.9257714	0
DN28407_c0_g1_i1_1	0.4226401	4.205228
DN28408_c0_g1_i1_1	0	0.4975593
DN28408_c0_g1_i1_2	373.2534	190.8135
DN28413_c0_g1_i1_2	0.779722	2.057803
DN28416_c0_g1_i1_1	0.1705614	2.036463
DN28420_c0_g1_i1_2	0	0
DN28424_c0_g1_i1_1	0	0.8362716
DN28424_c0_g1_i1_2	0	0
DN28427_c0_g1_i1_1	3.630458	6.125109
DN28429_c0_g1_i1_1	8.541539	39.59709
DN2842_c0_g1_i1_2	1.491126	1.374405
DN28435_c0_g1_i1_1	2.965867	9.126438
DN28439_c0_g1_i1_2	1.809848	1.431792
DN2843_c0_g1_i1_2	14.10044	16.12019
DN28441_c0_g1_i1_2	0.3730947	0.2927659
DN28442_c0_g1_i1_1	0.8011812	2.65E-29
DN28443_c0_g1_i1_1	0.5635537	0.7565677
DN28444_c0_g1_i1_1	0.7792785	2.038755

DN28445_c0_g1_i1_2	0.5617008	0.6908993
DN28446_c0_g1_i1_1	6.556921	4.511152
DN28447_c0_g1_i1_1	0.6696127	1.796538
DN28449_c0_g1_i1_1	0.5018319	0.3543393
DN28453_c0_g1_i1_1	0.9510134	0.9873168
DN28455_c0_g1_i1_1	1.005746	2.135522
DN28457_c0_g1_i1_2	0	0
DN28458_c0_g1_i1_1	2.133833	2.918811
DN28458_c0_g1_i1_2	0.41746	0.3703015
DN28459_c0_g1_i1_1	17.79232	13.74056
DN2845_c0_g1_i1_1	1.501205	1.302946
DN28460_c0_g1_i1_2	2.496609	0.2630583
DN28462_c0_g1_i1_1	0.3482207	1.302405
DN28465_c0_g1_i1_2	2.30411	2.244797
DN28466_c0_g1_i1_2	5.513249	1.328334
DN28467_c0_g1_i1_1	0.3010994	0.6819316
DN28469_c0_g1_i1_1	0.439164	0.6953307
DN28474_c0_g1_i1_2	0	0
DN28477_c0_g1_i1_1	3.176033	5.375967
DN28478_c0_g1_i1_2	1.123671	0.12434
DN28479_c0_g1_i1_2	35.12622	45.31595
DN28481_c0_g1_i1_1	0.5631809	0.7118769
DN28485_c0_g1_i1_2	4.308447	2.611142
DN28488_c0_g1_i1_2	2.304084	1.188758

DN2848_c0_g1_i1_2	1.866753	2.025825
DN28492_c0_g1_i1_2	1.954067	1.430622
DN28495_c0_g1_i1_1	1.141145	2.579181
DN28495_c0_g1_i1_2	1.194087	0.2010091
DN28498_c0_g1_i1_2	0	0
DN28499_c0_g1_i1_1	0.2767213	3.788459
DN28500_c0_g1_i1_2	0	0
DN28502_c0_g1_i1_2	1.033177	1.171397
DN28504_c0_g1_i1_1	0.3897599	0.5613831
DN28504_c0_g1_i1_2	1.49633	1.774352
DN28508_c0_g1_i1_2	2.279887	0.2329024
DN28515_c0_g1_i1_1	1.622785	2.617038
DN28516_c0_g1_i1_1	4.31792	5.124482
DN2851_c0_g1_i1_2	1.393136	0.8938612
DN28522_c0_g1_i1_2	126.7497	12.03835
DN28524_c0_g1_i1_1	0.3352084	0.4600661
DN28529_c0_g1_i1_2	16.51035	1.49526
DN28533_c0_g1_i1_1	14.06226	6.480173
DN28534_c0_g1_i1_1	1.668182	3.698747
DN28535_c0_g1_i1_1	7.319782	3.321292
DN28537_c0_g1_i1_1	1.79787	5.037563
DN28537_c0_g1_i1_2	2.903156	0
DN2853_c0_g1_i1_2	1.300453	1.202383
DN28542_c0_g1_i1_1	0.9688906	2.375143

DN28543_c0_g1_i1_1	1.604418	1.79592
DN28547_c0_g1_i1_1	0.3872659	1.038742
DN28553_c0_g1_i1_2	1.740464	0.3450512
DN28554_c0_g1_i1_1	1.043718	2.388582
DN28568_c0_g1_i1_1	1.659432	1.397502
DN28570_c0_g1_i1_2	1.308321	0
DN28573_c0_g1_i1_1	0.3458364	1.076727
DN28578_c0_g1_i1_1	1.340566	0.2528558
DN28578_c0_g1_i1_2	0.7647359	1.225521
DN28579_c0_g1_i1_1	2.268418	1.530171
DN28581_c0_g1_i1_2	1.932857	0.3680461
DN28582_c0_g1_i1_2	1.635121	1.98E-29
DN28584_c0_g1_i1_1	4.865088	2.037409
DN28585_c0_g1_i1_1	0.8378408	2.6103
DN28587_c0_g1_i1_2	2.133512	1.770766
DN28590_c0_g1_i1_2	3.512434	0
DN28591_c0_g1_i1_1	1.463027	3.941584
DN28594_c0_g1_i1_2	1.284954	0.3072267
DN28595_c0_g1_i1_2	0	0
DN28597_c0_g1_i1_1	15.71514	7.994415
DN28598_c0_g1_i1_1	0.8652914	3.121734
DN28598_c0_g1_i1_2	4.591602	1.769026
DN28603_c0_g1_i1_2	10.7696	0.6734668
DN28606_c0_g1_i1_2	3.968535	2.150785

DN28608_c0_g1_i1_1	2.298598	0.9665263
DN28611_c0_g1_i1_2	3.63213	0.2794746
DN28621_c0_g1_i1_1	0.5610507	5.396724
DN28622_c0_g1_i1_2	3.170466	2.217944
DN28624_c0_g1_i1_2	1.62569	1.242856
DN28625_c0_g1_i1_1	1.176919	0.9411014
DN28626_c0_g1_i1_2	5.568131	6.853283
DN28627_c0_g1_i1_2	9.094139	4.809196
DN28630_c0_g1_i1_1	2.28136	1.894585
DN28630_c0_g1_i1_2	5.365601	1.247022
DN28633_c0_g1_i1_1	3.061696	18.29007
DN28638_c0_g1_i1_1	34.97531	31.62682
DN2863_c0_g1_i1_1	1.730174	3.739074
DN28643_c0_g1_i1_1	0.9213536	0.6996678
DN28643_c0_g1_i1_2	0.7707372	0.5205317
DN28648_c0_g1_i1_1	0.5119294	2.677929
DN28652_c0_g1_i1_1	0.7390195	2.207099
DN28653_c0_g1_i1_1	1.246092	1.660127
DN28653_c0_g1_i1_2	7.113742	1.219601
DN28657_c0_g1_i1_1	14.48449	18.66651
DN28661_c0_g1_i1_1	0	0.4728456
DN28663_c0_g1_i1_1	25.82971	12.69621
DN28666_c0_g1_i1_2	1.779716	1.608327
DN28667_c0_g1_i1_1	1.699378	2.235214

DN28670_c0_g1_i1_1	1.065415	0
DN28673_c0_g1_i1_2	72.90562	44.76175
DN28674_c0_g1_i1_2	6.725754	2.193334
DN28676_c0_g1_i1_1	2.883476	2.460374
DN28677_c0_g1_i1_2	21.59922	89.03917
DN28678_c0_g1_i1_1	2.173413	2.609642
DN28679_c0_g1_i1_1	1.368905	2.473213
DN28687_c0_g1_i1_2	0.9284392	0.9345102
DN28688_c0_g1_i1_1	1.430368	2.68857
DN28706_c0_g1_i1_1	5.243314	1.980691
DN2870_c0_g1_i1_2	3.157961	0.8964123
DN28711_c0_g1_i1_1	0.4328957	0.6192931
DN28716_c0_g1_i1_2	15.12996	6.990557
DN28717_c0_g1_i1_1	10.75712	16.11801
DN28723_c0_g1_i1_2	2.009597	0.9649043
DN28727_c0_g1_i1_1	0.6880015	1.660443
DN28727_c0_g1_i1_2	2.613944	2.017323
DN28728_c0_g1_i1_1	0.5620182	5.410994
DN28729_c0_g1_i1_1	1753.915	1055.642
DN28734_c0_g1_i1_1	0.2872107	0.9352815
DN28737_c0_g1_i1_1	1.686717	1.69895
DN28740_c0_g1_i1_2	2.58779	1.865059
DN28742_c0_g1_i1_1	3.649384	1.915257
DN28744_c0_g1_i1_1	9.576212	8.530256

DN28747_c0_g1_i1_2	6.487975	5.046326
DN28750_c0_g1_i1_1	19.12325	11.42473
DN28750_c0_g1_i1_2	8.006697	24.68381
DN28751_c0_g1_i1_1	2.292527	4.073782
DN28752_c0_g1_i1_2	0.6275202	1.101789
DN28753_c0_g1_i1_2	1.769108	0.6991699
DN28756_c0_g1_i1_1	0	2.299592
DN28758_c0_g1_i1_1	1.075034	0.1760067
DN28761_c0_g1_i1_1	5.261178	23.13126
DN28762_c0_g1_i1_1	2.559084	7.68744
DN28769_c0_g1_i1_1	4.738057	1.590135
DN28771_c0_g1_i1_1	156.7239	137.8099
DN28773_c0_g1_i1_1	0.567139	0.9912233
DN28775_c0_g1_i1_1	0.6485049	0.8059484
DN28777_c0_g1_i1_2	0.6130452	1.981365
DN28779_c0_g1_i1_1	2.622595	4.147606
DN28779_c0_g1_i1_2	9.328242	2.063365
DN28782_c0_g1_i1_1	0	3.30532
DN28787_c0_g1_i1_1	1.70983	3.840818
DN28787_c0_g1_i1_2	3.490248	2.293152
DN28790_c0_g1_i1_2	5.735987	2.951605
DN28793_c0_g1_i1_1	4.265913	6.191562
DN28794_c0_g1_i1_2	0.5942081	1.154823
DN28798_c0_g1_i1_1	4.042706	1.184458

DN28802_c0_g1_i1_1	0.7494439	1.051546
DN28802_c0_g1_i1_2	2.278215	0.9300572
DN28804_c0_g1_i1_1	2.906578	11.44767
DN28812_c0_g1_i1_2	1.855573	1.334712
DN28813_c0_g1_i1_1	39.58638	69.88734
DN28816_c0_g1_i1_1	1.437235	0.4976009
DN28821_c0_g1_i1_2	1.485657	0.1653406
DN28822_c0_g1_i1_2	0	0
DN28825_c0_g1_i1_1	3.310807	7.40096
DN28825_c0_g1_i1_2	89.48432	32.30865
DN28828_c0_g1_i1_1	1.053039	0.276604
DN28831_c0_g1_i1_1	0.705724	1.797259
DN28833_c0_g1_i1_1	10.38363	8.240746
DN2883_c0_g1_i1_1	0.627526	0.527261
DN28840_c0_g1_i1_2	5.775409	17.49679
DN28842_c0_g1_i1_2	4.865325	1.722852
DN28848_c0_g1_i1_1	0.7960442	0.884405
DN28849_c0_g1_i1_1	1.223705	3.745733
DN28850_c0_g1_i1_1	2.533558	0.8762076
DN28851_c0_g1_i1_2	6.941155	3.809454
DN28854_c0_g1_i1_2	2.182582	1.028025
DN28855_c0_g1_i1_1	2.567943	1.952045
DN28856_c0_g1_i1_1	3.868644	4.662347
DN28860_c0_g1_i1_1	22.13995	24.55772

DN28867_c0_g1_i1_1	2.756535	1.532007
DN28867_c0_g1_i1_2	2.881575	1.296715
DN28868_c0_g1_i1_2	3.973187	0
DN28873_c0_g1_i1_2	28.01507	16.37169
DN28877_c0_g1_i1_1	0.3454503	1.558625
DN28877_c0_g1_i1_2	2.883146	1.184349
DN28878_c0_g1_i1_2	15.65511	2.513171
DN28879_c0_g1_i1_1	19.69877	23.53608
DN28885_c0_g1_i1_1	2.47987	2.088466
DN28885_c0_g1_i1_2	5.004258	2.223735
DN28891_c0_g1_i1_1	1.638322	2.236963
DN28896_c0_g1_i1_1	1.260009	3.713433
DN28896_c0_g1_i1_2	1.702792	0.4832574
DN28899_c0_g1_i1_1	1.791462	5.34223
DN28900_c0_g1_i1_1	1.191947	2.281134
DN28901_c0_g1_i1_1	0.1434816	13.27087
DN28902_c0_g1_i1_1	1.103689	0.9484874
DN28902_c0_g1_i1_2	47.35592	20.97633
DN28906_c0_g1_i1_1	0	0.2251161
DN28911_c0_g1_i1_1	16.29495	9.707751
DN28911_c0_g1_i1_2	2.289041	1.253068
DN28914_c0_g1_i1_2	2.278636	0.5969986
DN28917_c0_g1_i1_2	2.342596	1.361252
DN28918_c0_g1_i1_1	2.05005	4.014365

DN28920_c0_g1_i1_1	5.254114	3.348891
DN28921_c0_g1_i1_2	0	0
DN28923_c0_g1_i1_2	3.145514	0.8937388
DN28928_c0_g1_i1_1	33.5284	48.00933
DN28929_c0_g1_i1_1	2.310588	0
DN28935_c0_g1_i1_2	0.9685892	0.2233658
DN28936_c0_g1_i1_2	9.719488	8.862636
DN28938_c0_g1_i1_2	6.186823	1.787444
DN28939_c0_g1_i1_1	2.187308	12.47787
DN2893_c0_g1_i1_1	0.9191075	0.3692835
DN28940_c0_g1_i1_2	1.75631	0.405098
DN28948_c0_g1_i1_1	1.555322	1.419231
DN28948_c0_g1_i1_2	4.251082	1.399831
DN28950_c0_g1_i1_1	1.498598	3.905925
DN28952_c0_g1_i1_1	0.8260995	0.6294542
DN28955_c0_g1_i1_1	3.484764	1.929271
DN28957_c0_g1_i1_1	0.3112422	2.375213
DN28958_c0_g1_i1_1	1.72552	3.335801
DN28961_c0_g1_i1_1	0.3128454	1.809611
DN28961_c0_g1_i1_2	3.838065	3.706854
DN28962_c0_g1_i1_1	1.685437	1.951297
DN28967_c0_g1_i1_1	5.51196	1.734117
DN28968_c0_g1_i1_1	2.009027	4.200093
DN28971_c0_g1_i1_1	0.4593333	0.4526846

DN28980_c0_g1_i1_2	38.08254	0.1657165
DN28983_c0_g1_i1_1	2.558542	0.950587
DN28985_c0_g1_i1_2	2.116429	0.5855908
DN28987_c0_g1_i1_2	11.06129	6.402436
DN28988_c0_g1_i1_1	8.065581	5.544667
DN2898_c0_g1_i1_1	1.261812	1.13541
DN28991_c0_g1_i1_2	0.9522976	0.7220126
DN28992_c0_g1_i1_1	1.035314	0.9065165
DN28992_c0_g1_i1_2	10.63766	4.106762
DN28995_c0_g1_i1_2	20.63708	24.60523
DN28998_c0_g1_i1_2	5.889585	4.345555
DN28999_c0_g1_i1_1	1.40819	2.002338
DN29000_c0_g1_i1_1	0.2549557	0.5776824
DN29002_c0_g1_i1_2	2.07299	1.014015
DN29005_c0_g1_i1_1	1.246159	1.838515
DN29006_c0_g1_i1_1	0.9321061	0.4599927
DN29016_c0_g1_i1_1	3.858842	10.88732
DN29018_c0_g1_i1_1	1.37704	1.454656
DN29019_c0_g1_i1_2	1.322414	0.9495123
DN29025_c0_g1_i1_1	0	4.495987
DN29029_c0_g1_i1_2	0.1010219	0.2348934
DN29035_c0_g1_i1_1	3.266862	24.5033
DN29039_c0_g1_i1_1	4.145817	6.096855
DN29039_c0_g1_i1_2	1.456285	0.1228401

DN29041_c0_g1_i1_1	0.5235592	1.744554
DN29044_c0_g1_i1_1	1.629793	2.805053
DN29044_c0_g1_i1_2	0	0
DN29051_c0_g1_i1_1	0.1601294	0.4920076
DN29051_c0_g1_i1_2	4.561388	5.03549
DN29052_c0_g1_i1_2	1.778528	0.624503
DN29060_c0_g1_i1_1	7.609194	5.390092
DN29065_c0_g1_i1_1	103.8956	58.67784
DN29070_c0_g1_i1_2	0.8851019	0
DN29073_c0_g1_i1_1	0.6034361	1.182571
DN29081_c0_g1_i1_1	1.634102	1.536714
DN29082_c0_g1_i1_1	38.21361	1533.198
DN29083_c0_g1_i1_2	7.304741	24.21713
DN29084_c0_g1_i1_2	3.441328	1.466869
DN29085_c0_g1_i1_2	0	0.3189109
DN29086_c0_g1_i1_1	1.052484	1.258731
DN29087_c0_g1_i1_2	3.635549	0.8285324
DN29088_c0_g1_i1_1	0.8550026	0.7316494
DN2908_c0_g1_i1_1	0.3191143	0.1282995
DN29092_c0_g1_i1_1	2.303643	2.47651
DN29096_c0_g1_i1_1	1.031898	1.259485
DN29097_c0_g1_i1_1	282.5464	521.1606
DN29099_c0_g1_i1_1	4.901184	34.56319
DN29100_c0_g1_i1_1	11499	2913.561

DN29102_c0_g1_i1_1	8.255971	6.632005
DN29103_c0_g1_i1_2	7.851352	5.799339
DN29109_c0_g1_i1_1	24.28209	16.55934
DN29113_c0_g1_i1_1	2.761054	5.843993
DN29114_c0_g1_i1_1	0	5.22488
DN29115_c0_g1_i1_2	1.735149	1.177749
DN29116_c0_g1_i1_1	87.32254	75.35413
DN29128_c0_g1_i1_1	0	1.109747
DN29130_c0_g1_i1_1	0.6265839	0.4186854
DN29131_c0_g1_i1_1	1.428103	3.386822
DN29132_c0_g1_i1_1	0.2971056	0.6084908
DN29134_c0_g1_i1_1	2.382352	1.625276
DN29137_c0_g1_i1_2	0.8102694	0
DN29143_c0_g1_i1_1	0.6805676	1.608575
DN29144_c0_g1_i1_1	0.7211934	2.128043
DN29146_c0_g1_i1_2	4.305851	1.853535
DN29148_c0_g1_i1_1	0.8401169	1.145133
DN29153_c0_g1_i1_1	1.883277	23.5764
DN29155_c0_g1_i1_1	12.01011	23.81361
DN29157_c0_g1_i1_2	3.014147	5.442084
DN29159_c0_g1_i1_1	0.9938322	0.559988
DN29160_c0_g1_i1_2	12.5196	1.240028
DN29166_c0_g1_i1_1	25.51469	4.423951
DN29167_c0_g1_i1_2	6.44835	4.401598

DN29169_c0_g1_i1_1	0	0.8635205
DN29173_c0_g1_i1_1	1.156714	0.8120962
DN29175_c0_g1_i1_1	0.09164293	3.008769
DN29177_c0_g1_i1_2	1.085104	1.080556
DN29190_c0_g1_i1_1	0.8842365	3.19652
DN29195_c0_g1_i1_1	0.8769637	4.694405
DN2919_c0_g1_i2_1	2.403282	1.882234
DN29200_c0_g1_i1_1	1.47E-28	1.115914
DN29202_c0_g1_i1_1	3.772829	6.902798
DN29202_c0_g1_i1_2	1.628171	0.2684613
DN29204_c0_g1_i1_1	2.000474	7.123948
DN29205_c0_g1_i1_2	1.348354	1.271411
DN29217_c0_g1_i1_1	1.943861	15.7957
DN29218_c0_g1_i1_1	2.639643	7.932313
DN29221_c0_g1_i1_1	4.854433	2.962997
DN29222_c0_g1_i1_1	0.4987901	0.5964653
DN29225_c0_g1_i1_1	1.204234	0.6063841
DN29234_c0_g1_i1_2	3.243788	0.9369393
DN29238_c0_g1_i1_2	1.480906	1.170191
DN29242_c0_g1_i1_1	1.30254	0.9968158
DN29245_c0_g1_i1_1	11.47224	13.56724
DN29250_c0_g1_i1_1	0.1499468	3.363528
DN29251_c0_g1_i1_1	0.5691469	1.619486
DN29254_c0_g1_i1_2	2.026759	2.47541

DN29256_c0_g1_i1_2	0.8945967	0.8038338
DN29257_c0_g1_i1_1	0.7975237	1.523605
DN29258_c0_g1_i1_1	0.3501928	0
DN29266_c0_g1_i1_2	1.887229	0.4460347
DN29270_c0_g1_i1_1	0.9438288	0.7082169
DN29275_c0_g1_i1_2	1.995551	0.246971
DN29276_c0_g1_i1_1	1.565212	2.0304
DN29278_c0_g1_i1_1	18.01206	20.3805
DN29286_c0_g1_i1_1	0.9249986	7.166976
DN29287_c0_g1_i1_1	0.2511639	0.1590675
DN29287_c0_g1_i1_2	0	0
DN29292_c0_g1_i1_1	1.922177	3.785676
DN29292_c0_g1_i1_2	617.4778	0.1481115
DN29293_c0_g1_i1_1	0.4164462	1.693289
DN29299_c0_g1_i1_2	2.694264	0.3337498
DN29300_c0_g1_i1_1	0	1.784416
DN29304_c0_g1_i1_1	0.2532682	7.211218
DN2930_c0_g1_i1_1	0.9671622	4.508372
DN29310_c0_g1_i1_1	1.27E-23	1.395316
DN29313_c0_g1_i1_1	0.2879052	1.258619
DN29314_c0_g1_i1_1	0.8469084	1.14789
DN29314_c0_g1_i1_2	5.191876	3.005056
DN29316_c0_g1_i1_2	5.673484	6.597111
DN29317_c0_g1_i1_1	1.398051	3.164945

DN29319_c0_g1_i1_1	0	5.116237
DN2931_c0_g1_i1_2	21.66152	26.48349
DN29320_c0_g1_i1_1	1.657721	0.9797135
DN29322_c0_g1_i1_2	2.615091	0
DN29324_c0_g1_i1_1	0	0
DN29326_c0_g1_i1_1	0	2.246588
DN29328_c0_g1_i1_2	0.2199901	0.05035738
DN29330_c0_g1_i1_1	2.00663	2.614944
DN29334_c0_g1_i1_2	0.8910344	0.1045725
DN29335_c0_g1_i1_1	0.8921192	0.3167262
DN29340_c0_g1_i1_2	2.293232	0.5844893
DN29341_c0_g1_i1_1	6.661827	5.518913
DN29342_c0_g1_i1_1	0.1435729	1.008735
DN29343_c0_g1_i1_1	1.138039	0.1409025
DN29346_c0_g1_i1_1	1.14012	15.57331
DN29347_c0_g1_i1_2	1.564878	1.099022
DN29349_c0_g1_i1_1	0.2117909	1.970548
DN29352_c0_g1_i1_2	0	0
DN29355_c0_g1_i1_1	23.3903	11.33208
DN29356_c0_g1_i1_1	0.5680043	1.111557
DN29357_c0_g1_i1_1	0.5768788	0.9839625
DN29359_c0_g1_i1_1	20.67907	11.22266
DN29362_c0_g1_i1_1	0.6503198	1.124786
DN29364_c0_g1_i1_1	0	23.40328

DN29364_c0_g1_i1_2	2.938881	1.625756
DN29365_c0_g1_i1_2	9.236247	8.554437
DN29373_c0_g1_i1_1	0.1441746	2.231991
DN29374_c0_g1_i1_1	1.454334	2.899069
DN29376_c0_g1_i1_2	1.286968	0.9274476
DN29381_c0_g1_i1_1	3.378093	2.061314
DN29382_c0_g1_i1_1	1.295432	2.855654
DN29384_c0_g1_i1_1	1.269021	1.48193
DN29385_c0_g1_i1_1	0.5580835	0.4757752
DN29387_c0_g1_i1_1	21.56699	27.08799
DN2938_c0_g1_i1_1	0.8420691	1.332977
DN29390_c0_g1_i1_2	1.707348	1.006677
DN29392_c0_g1_i1_1	1.611703	1.969213
DN29394_c0_g1_i1_2	12.69176	6.262763
DN29397_c0_g1_i1_1	2.599717	0.2661556
DN29398_c0_g1_i1_1	0.7151978	0.6728545
DN29400_c0_g1_i1_1	0	0.4636356
DN29405_c0_g1_i1_1	9.598801	6.977602
DN29411_c0_g1_i1_1	0.2203536	0.1581781
DN29414_c0_g1_i1_2	2.507807	0.4759804
DN29415_c0_g1_i1_1	0.06133311	2.034887
DN29416_c0_g1_i1_1	2.215691	2.059499
DN29417_c0_g1_i1_1	0.8824444	1.213819
DN29417_c0_g1_i1_2	6.169524	6.100654

DN29422_c0_g1_i1_1	0.2738191	1.593812
DN29425_c0_g1_i1_1	39.66568	23.0082
DN29425_c0_g1_i1_2	2.93783	1.588579
DN29426_c0_g1_i1_1	0.2586078	0.6127537
DN29427_c0_g1_i1_1	1.532971	1.598811
DN29433_c0_g1_i1_1	3.54381	3.148098
DN29436_c0_g1_i1_1	0.9925744	1.835588
DN29438_c0_g1_i1_1	1.379315	1.254653
DN29440_c0_g1_i1_2	1.766711	0.2416755
DN29443_c0_g1_i1_2	3.254748	3.186378
DN29445_c0_g1_i1_1	59.47686	76.60877
DN29451_c0_g1_i1_1	0	1.644284
DN29452_c0_g1_i1_1	0.4861498	1.328568
DN29452_c0_g1_i1_2	10.07638	59.39381
DN29453_c0_g1_i1_2	0	1.723913
DN29460_c0_g1_i1_2	1.480674	0.2291692
DN29463_c0_g1_i1_2	8.641476	12.14899
DN29469_c0_g1_i1_2	8.366726	5.347194
DN2946_c0_g1_i1_1	4.246189	1.995971
DN29472_c0_g1_i1_1	6.634512	13.86851
DN29473_c0_g1_i1_1	11.50592	6.737215
DN29474_c0_g1_i1_1	0.7127586	0.9589241
DN29475_c0_g1_i1_2	18.45129	9.026264
DN29478_c0_g1_i1_1	0.2165297	0.9780693

DN29480_c0_g1_i1_1	0	1.305394
DN29482_c0_g1_i1_1	0.6819601	1.065836
DN29484_c0_g1_i1_1	0.7585572	1.686979
DN29486_c0_g1_i1_1	2.475958	1.497279
DN29492_c0_g1_i1_2	0	0
DN29497_c0_g1_i1_1	1.202823	2.986116
DN29501_c0_g1_i1_1	0.3405691	1.309337
DN29502_c0_g1_i1_2	3.845946	2.661424
DN29503_c0_g1_i1_1	0.1832943	0.5834287
DN29503_c0_g1_i1_2	2.632729	1.235535
DN29505_c0_g1_i1_1	0.1878031	0.2375628
DN29507_c0_g1_i1_2	3.168246	4.788585
DN29509_c0_g1_i1_2	0.1751094	2.605805
DN2950_c0_g1_i1_1	1.946846	3.286168
DN29510_c0_g1_i1_1	1.32E-16	1.616158
DN29511_c0_g1_i1_1	0.4712331	1.055201
DN29512_c0_g1_i1_1	0.9324438	0.6971461
DN29513_c0_g1_i1_1	0.6649163	4.605039
DN29517_c0_g1_i1_1	1.848613	0.6444997
DN29518_c0_g1_i1_1	2.467769	2.551737
DN29519_c0_g1_i1_1	3.181191	4.814453
DN2951_c0_g1_i1_2	0	0
DN29520_c0_g1_i1_1	0.6168318	4.872135
DN29521_c0_g1_i1_1	0.9615374	1.568407

DN29523_c0_g1_i1_1	2.19719	1.380443
DN29526_c0_g1_i1_2	3.104017	1.072
DN29527_c0_g1_i1_1	0.7993132	0.9490881
DN29529_c0_g1_i1_1	1.767068	2.855281
DN29530_c0_g1_i1_1	0.6070066	0.9191525
DN29533_c0_g1_i1_2	7.346734	3.391979
DN29534_c0_g1_i1_2	1.989009	0
DN29537_c0_g1_i1_1	0.9316313	1.95459
DN29538_c0_g1_i1_1	1.086531	21.40443
DN29542_c0_g1_i1_2	2.349136	0.5607174
DN29543_c0_g1_i1_2	1.974704	0.9203917
DN29546_c0_g1_i1_1	0	1.688598
DN29548_c0_g1_i1_1	2.523281	4.264626
DN29548_c0_g1_i1_2	1.641226	1.387427
DN29563_c0_g1_i1_1	0	0
DN29563_c0_g1_i1_2	10.16832	8.933088
DN29566_c0_g1_i1_2	423.025	331.7613
DN29569_c0_g1_i1_1	19.22073	7.78028
DN29569_c0_g1_i1_2	0	0
DN29577_c0_g1_i1_1	6.483988	6.208844
DN2957_c0_g1_i1_1	1.475096	2.191911
DN29581_c0_g1_i1_1	0.678459	0.9734351
DN29582_c0_g1_i1_1	2.070041	1.201393
DN29585_c0_g1_i1_1	11.2488	8.865531

DN29586_c0_g1_i1_2	3.757868	0.9696539
DN29588_c0_g1_i1_1	0.9713442	3.858371
DN29593_c0_g1_i1_1	5.254955	11.27282
DN29596_c0_g1_i1_1	0.9913062	1.42338
DN29596_c0_g1_i1_2	1.645927	1.214812
DN29597_c0_g1_i1_1	1.359023	1.248532
DN29597_c0_g1_i1_2	1.294777	1.73258
DN2959_c0_g2_i1_2	2.771158	2.323504
DN295_c0_g1_i1_1	2.290781	0.667711
DN29600_c0_g1_i1_1	1.653505	1.693434
DN29605_c0_g1_i1_1	33.17091	35.77789
DN29606_c0_g1_i1_1	0.09977171	0.1575675
DN29606_c0_g1_i1_2	5.239682	3.076122
DN29607_c0_g1_i1_1	0.1266962	1.021624
DN29609_c0_g1_i1_1	2.766059	3.584504
DN2960_c0_g1_i1_1	0.4929808	3.157889
DN29612_c0_g1_i1_1	0.6614879	0.9999196
DN29613_c0_g1_i1_2	3.826303	1.966255
DN29619_c0_g1_i1_1	0.5720618	1.19838
DN2961_c0_g1_i1_1	0.4132426	1.556703
DN29621_c0_g1_i1_1	1.953922	3.281341
DN29622_c0_g1_i1_1	3.542946	2.273563
DN29628_c0_g1_i1_1	1.500626	9.466683
DN29629_c0_g1_i1_2	1.689421	0

DN29633_c0_g1_i1_2	3.296206	0.936827
DN29635_c0_g1_i1_2	0.3434257	0.8065158
DN29636_c0_g1_i1_1	0.2754286	2.940996
DN29637_c0_g1_i1_1	0.7806589	1.58E-27
DN29637_c0_g1_i1_2	1.644934	0
DN29638_c0_g1_i1_2	2.716009	1.495575
DN29639_c0_g1_i1_1	0.2372798	0.101012
DN29639_c0_g1_i1_2	1.844708	1.193305
DN29640_c0_g1_i1_2	0.8419544	0.9751659
DN29641_c0_g1_i1_1	2.127316	2.000445
DN29642_c0_g1_i1_1	0.3929347	0.3106014
DN29645_c0_g1_i1_2	0.1291011	0
DN29646_c0_g1_i1_1	3.393782	0.9541261
DN29649_c0_g1_i1_2	5.887533	6.857745
DN2964_c0_g1_i1_2	1.121582	0.2855734
DN29650_c0_g1_i1_1	0.2422226	0
DN29650_c0_g1_i1_2	0.5344048	0.6293286
DN29652_c0_g1_i1_1	0.02152628	0.1650997
DN29653_c0_g1_i1_2	2.711613	0.4986389
DN29655_c0_g1_i1_1	0.3891732	1.718266
DN29656_c0_g1_i1_2	1.372756	0.7650821
DN29659_c0_g1_i1_1	1.050745	0.640075
DN29659_c0_g1_i1_2	6.756328	2.179894
DN2965_c0_g1_i1_2	2.407028	0.937992

DN29662_c0_g1_i1_2	2.484781	0.8185949
DN29663_c0_g1_i1_1	1.388338	1.32171
DN29664_c0_g1_i1_1	1.205008	0.995556
DN29665_c0_g1_i1_1	2.077382	1.725434
DN29666_c0_g1_i1_1	1.66458	1.851092
DN29672_c0_g1_i1_1	2.180203	8.158462
DN29674_c0_g1_i1_1	0	1.025152
DN29675_c0_g1_i1_1	0.6907942	0.3763949
DN29679_c0_g1_i1_2	2.266143	1.109076
DN29684_c0_g1_i1_1	0.5176725	1.64657
DN29687_c0_g1_i1_1	0.825148	1.234633
DN29688_c0_g1_i1_1	0.8814559	1.395597
DN29688_c0_g1_i1_2	4.370261	2.264541
DN29689_c0_g1_i1_1	1.592474	3.797579
DN29690_c0_g1_i1_1	2.083102	1.317942
DN29691_c0_g1_i1_2	9.833714	11.89744
DN29692_c0_g1_i1_2	3.596507	0.9745307
DN29693_c0_g1_i1_2	1.405956	0.3209171
DN29694_c0_g1_i1_1	1.26553	0.7090952
DN29698_c0_g1_i1_1	0.34909	2.487912
DN29698_c0_g1_i1_2	1.829957	0
DN29699_c0_g1_i1_2	4.579315	2.915536
DN29702_c0_g1_i1_2	2.009361	2.638243
DN29705_c0_g1_i1_1	0	0.4341235

DN29710_c0_g1_i1_2	0.6715632	0.2087557
DN29712_c0_g1_i1_1	1.496948	7.27382
DN29714_c0_g1_i1_1	0.5748395	0.6375467
DN29716_c0_g1_i1_1	3.488422	3.375286
DN29717_c0_g1_i1_1	1.193056	2.769365
DN29718_c0_g1_i1_2	3.976669	5.308667
DN2971_c0_g1_i1_1	2.044502	4.135911
DN29721_c0_g1_i1_1	0.5010698	0.3441681
DN29723_c0_g1_i1_2	0.607484	0.816285
DN29729_c0_g1_i1_2	52.27518	28.83354
DN2972_c0_g1_i1_2	0	0
DN29732_c0_g1_i1_1	0.3947172	2.125497
DN29737_c0_g1_i1_2	5.290373	5.657229
DN29739_c0_g1_i1_1	0.812718	3.239423
DN29740_c0_g1_i1_2	0.2746167	0.8576536
DN29741_c0_g1_i1_1	0.6083192	1.145653
DN29745_c0_g1_i1_1	0.4326684	0.3261147
DN29751_c0_g1_i1_1	1.028154	4.932468
DN29754_c0_g1_i1_1	0.5282111	0.6973162
DN29754_c0_g1_i1_2	1.447125	0.3289363
DN29756_c0_g1_i1_1	2.775587	2.507434
DN29760_c0_g1_i1_1	1.066277	0.6008356
DN29762_c0_g1_i1_1	0.292196	6.591555
DN29764_c0_g1_i1_1	0.7683276	3.006155

DN29765_c0_g1_i1_1	0.1887509	0.3125386
DN29769_c0_g1_i1_1	0.8147693	3.802922
DN2976_c0_g1_i2_2	4.382522	3.710948
DN29771_c0_g1_i1_1	0.2327761	0.9312022
DN29772_c0_g1_i1_1	3.395093	1.788789
DN29777_c0_g1_i1_1	2.231179	3.300743
DN29778_c0_g1_i1_2	14.78437	6.756824
DN2977_c0_g1_i1_1	1.352444	6.110349
DN29781_c0_g1_i1_1	0.67265	3.889036
DN29784_c0_g1_i1_1	0.421723	1.21727
DN29784_c0_g1_i1_2	1.68749	0.4688696
DN29787_c0_g1_i1_2	11.35786	3.076287
DN29790_c0_g1_i1_2	5.332913	2.259798
DN29793_c0_g1_i1_1	2.041624	3.689915
DN29798_c0_g1_i1_1	1.218517	1.517391
DN29799_c0_g1_i1_1	5.603153	14.80263
DN29799_c0_g1_i1_2	2.842714	0.8459535
DN2979_c0_g1_i1_1	1.358818	2.067449
DN29800_c0_g1_i1_1	0.9184518	1.348862
DN29802_c0_g1_i1_1	2.911727	1.484124
DN29803_c0_g1_i1_1	0.4078851	1.304749
DN29805_c0_g1_i1_2	4.862279	2.530799
DN29809_c0_g1_i1_1	0.28469	2.537677
DN29813_c0_g1_i1_2	1.27985	0.8767472

DN29815_c0_g1_i1_2	4.881843	2.368145
DN29818_c0_g1_i1_1	10.15004	6.319978
DN29820_c0_g1_i1_2	3.671757	5.310023
DN29823_c0_g1_i1_2	7.08994	3.553444
DN29825_c0_g1_i1_1	2.94E-07	0.8301603
DN29826_c0_g1_i1_1	0	3.302573
DN29828_c0_g1_i1_1	1.957005	3.993587
DN29830_c0_g1_i1_1	0	0.212658
DN29832_c0_g1_i1_1	9.93E-06	0.6249109
DN29835_c0_g1_i1_1	0.9249637	3.675466
DN29836_c0_g1_i1_1	2.289734	0.5581098
DN29837_c0_g1_i1_1	0.4217528	0
DN29838_c0_g1_i1_1	0	0.9518776
DN29838_c0_g1_i1_2	1.487415	1.100288
DN29839_c0_g1_i1_1	0.05530947	7.319864
DN29840_c0_g1_i1_1	0.4209672	6.324443
DN29841_c0_g1_i1_1	129.3834	147.9017
DN29841_c0_g1_i1_2	20.09638	4.832707
DN29843_c0_g1_i1_1	1.808109	3.749122
DN29853_c0_g1_i1_1	0.5103454	6.134254
DN29856_c0_g1_i1_1	0.5891751	2.014469
DN29863_c0_g1_i1_1	0	0
DN29864_c0_g1_i1_1	4.982822	11.07459
DN29866_c0_g1_i1_1	1.791221	1.435026

DN29867_c0_g1_i1_1	0.3828701	1.084703
DN29868_c0_g1_i1_1	0.1979867	0.3889136
DN29870_c0_g1_i1_1	17.72305	5.143144
DN29871_c0_g1_i1_1	1.131513	0.7086276
DN29877_c0_g1_i1_1	0.4650855	0.194643
DN29878_c0_g1_i1_1	1.319057	0.3063897
DN29879_c0_g1_i1_1	0.2026656	0.545299
DN2987_c0_g1_i1_2	1.578845	2.369398
DN29881_c0_g1_i1_2	2.355189	1.635324
DN29885_c0_g1_i1_2	1.323023	0.4869264
DN29886_c0_g1_i1_2	3.130445	2.34834
DN29891_c0_g1_i1_1	10.01609	5.477688
DN29893_c0_g1_i1_1	0	0.1780517
DN29894_c0_g1_i1_1	3.565394	6.155006
DN29894_c0_g1_i1_2	1.561833	0
DN29896_c0_g1_i1_1	1.052499	2.238337
DN29897_c0_g1_i1_1	0.8673685	2.875476
DN29897_c0_g1_i1_2	0.7523499	0.8239282
DN29899_c0_g1_i1_1	0.8650213	2.053623
DN29899_c0_g1_i1_2	5.336809	1.631249
DN2989_c0_g1_i1_1	4.635913	3.695752
DN29902_c0_g1_i1_1	0.687991	0.9914037
DN29904_c0_g1_i1_1	1.293102	2.568152
DN29905_c0_g1_i1_1	0.9956963	1.004811

DN29905_c0_g1_i1_2	8.773501	0.5215109
DN29907_c0_g1_i1_1	0.4298758	0
DN29907_c0_g1_i1_2	0.6598921	0
DN29911_c0_g1_i1_2	6.799255	0.9755919
DN29914_c0_g1_i1_1	3.971995	4.259481
DN29914_c0_g1_i1_2	5.146478	0
DN29915_c0_g1_i1_2	0	0
DN29918_c0_g1_i1_2	2.111697	0.2262657
DN29920_c0_g1_i1_1	9.552407	43.10564
DN29922_c0_g1_i1_2	1.545694	0.8813909
DN29928_c0_g1_i1_2	0	0.6462259
DN29932_c0_g1_i1_2	0.7411159	0.1612377
DN29934_c0_g1_i1_2	1.687927	1.380249
DN29937_c0_g1_i1_1	278.566	123.7686
DN29939_c0_g1_i1_1	3.194321	1.666659
DN29941_c0_g1_i1_2	1.037805	0.4612718
DN29943_c0_g1_i1_2	0.1982185	2.835022
DN29944_c0_g1_i1_1	0.5444163	0.5294511
DN29944_c0_g1_i1_2	1.289073	0.4465377
DN29945_c0_g1_i1_1	0.6315623	1.190763
DN29945_c0_g1_i1_2	5.177247	5.394595
DN29948_c0_g1_i1_1	0.9144122	3.227707
DN29955_c0_g1_i1_1	17.97215	6.66877
DN29956_c0_g1_i1_2	0.5261371	0.8211719

DN29957_c0_g1_i1_2	0.2683392	0.5716704
DN29958_c0_g1_i1_2	6.079118	8.336182
DN29960_c0_g1_i1_1	17.96462	5.262537
DN29961_c0_g1_i1_1	3.409733	3.697523
DN29961_c0_g1_i1_2	1.390904	2.257097
DN29964_c0_g1_i1_1	3.823452	4.623684
DN29966_c0_g1_i1_2	1.866656	0.2639051
DN29967_c0_g1_i1_1	0.6673343	0.6109107
DN29969_c0_g1_i1_1	0.310779	0.4201217
DN2996_c0_g1_i1_1	0.2985533	2.362632
DN29971_c0_g1_i1_2	0	0.6136644
DN29974_c0_g1_i1_2	26.53998	0
DN29979_c0_g1_i1_2	0.8109151	0
DN29980_c0_g1_i1_2	2.997687	1.606624
DN29981_c0_g1_i1_1	1.612022	8.936296
DN29982_c0_g1_i1_2	0	0
DN29985_c0_g1_i1_2	1.782376	0.818887
DN29991_c0_g1_i1_1	1.939982	1.395086
DN29994_c0_g1_i1_1	0	0.874048
DN29998_c0_g1_i1_1	3.382241	4.070453
DN2999_c0_g2_i1_2	0.5416133	0.2774705
DN2_c0_g2_i1_1	2.284998	4.277046
DN30001_c0_g1_i1_2	99.47714	89.59345
DN30003_c0_g1_i1_1	7.012925	10.61639

DN30004_c0_g1_i1_1	1.354109	1.417289
DN30004_c0_g1_i1_2	3.353066	0.8566853
DN30011_c0_g1_i1_2	1.37225	0.2602157
DN30012_c0_g1_i1_2	0	0
DN30019_c0_g1_i1_2	2.673388	1.273146
DN30022_c0_g1_i1_2	1.206701	0.9494411
DN30024_c0_g1_i1_2	2.910194	0.09424663
DN30027_c0_g1_i1_1	0.8148194	1.024685
DN30028_c0_g1_i1_1	1.565994	10.38385
DN3002_c0_g1_i1_2	0	0
DN30034_c0_g1_i1_2	7.630595	4.127356
DN30035_c0_g1_i1_1	3.211564	3.384265
DN30037_c0_g1_i1_1	1.254158	0.3032976
DN30040_c0_g1_i1_1	0.6937313	1.662836
DN30046_c0_g1_i1_2	0.4619755	0.6538575
DN30051_c0_g1_i1_1	0.1556354	6.030588
DN30052_c0_g1_i1_1	169.4857	99.52764
DN30057_c0_g1_i1_2	2.250904	0.4018055
DN30065_c0_g1_i1_1	0.2477615	0.4389575
DN30066_c0_g1_i1_2	2.227702	0.5060876
DN30067_c0_g1_i1_1	8.952684	15.69077
DN30067_c0_g1_i1_2	1.308956	0
DN30068_c0_g1_i1_1	1.124716	2.550838
DN30068_c0_g1_i1_2	2.649584	1.620922

DN30070_c0_g1_i1_1	0	0
DN30075_c0_g1_i1_2	0.6352089	0.3497631
DN30080_c0_g1_i1_2	15.59702	11.34907
DN30081_c0_g1_i1_1	1.79513	2.13461
DN30083_c0_g1_i1_2	5.064768	5.988641
DN30094_c0_g1_i1_1	3.665154	3.930042
DN30099_c0_g1_i1_1	1.36764	0.414315
DN30100_c0_g1_i1_2	2.060565	0.187872
DN30102_c0_g1_i1_2	2.367716	1.62241
DN30104_c0_g1_i1_2	2.127462	0
DN30106_c0_g1_i1_2	10.98064	23.98189
DN30110_c0_g1_i1_1	1.471264	1.460953
DN30113_c0_g1_i1_1	8.995416	2.879401
DN30121_c0_g1_i1_2	28.48196	17.47278
DN30122_c0_g1_i1_2	2.728374	1.973982
DN30129_c0_g1_i1_1	0.689865	0.6305757
DN3012_c0_g1_i1_2	1.485062	0.184381
DN30133_c0_g1_i1_2	2.17629	0.7584054
DN30140_c0_g1_i1_2	8.960806	1.97876
DN30141_c0_g1_i1_1	1.913496	4.757726
DN30144_c0_g1_i1_1	22.17756	15.09571
DN30146_c0_g1_i1_2	2.054483	2.77048
DN30147_c0_g1_i1_2	0.8653112	0.2242035
DN30148_c0_g1_i1_1	0.3396297	0.2411762

DN30148_c0_g1_i1_2	4.641785	3.747851
DN3014_c0_g1_i1_2	0.2016038	0
DN3014_c0_g2_i1_2	0.7393599	0
DN30158_c0_g1_i1_1	0.6650199	0.6883886
DN30163_c0_g1_i1_1	0.9658107	0.6655558
DN30167_c0_g1_i1_2	129.7677	28.83659
DN3016_c0_g1_i1_1	0.316229	1.271253
DN30170_c0_g1_i1_1	1.75539	3.610533
DN30171_c0_g1_i1_1	0.7270758	2.900918
DN30172_c0_g1_i1_1	0.573339	1.582468
DN30173_c0_g1_i1_2	8.653796	1.866692
DN30174_c0_g1_i1_2	1.209488	0.2300376
DN30177_c0_g1_i1_1	1.262974	2.562131
DN30181_c0_g1_i1_1	27.44048	40.57807
DN30185_c0_g1_i1_1	0.6367507	2.19662
DN30189_c0_g1_i1_2	3.290348	1.503639
DN30190_c0_g1_i1_1	53.97697	33.11077
DN30193_c0_g1_i1_2	1.995587	0.69781
DN30195_c0_g1_i1_1	0.1486529	0.9515594
DN30195_c0_g1_i1_2	0.2229027	7.28E-31
DN30201_c0_g1_i1_2	1.118648	0.2301931
DN30203_c0_g1_i1_1	0.6708952	3.44472
DN30203_c0_g1_i1_2	2.599103	1.435486
DN30205_c0_g1_i1_2	0	0

DN30206_c0_g1_i1_2	17.80669	33.62297
DN30210_c0_g1_i1_2	2.666276	1.561608
DN30212_c0_g1_i1_2	2.308695	0.6728185
DN30216_c0_g1_i1_1	1.198099	1.385667
DN30216_c0_g1_i1_2	1.479016	0.3557367
DN30218_c0_g1_i1_1	1.993725	1.574463
DN30220_c0_g1_i1_1	52.63927	35.07975
DN30221_c0_g1_i1_1	1.422575	0.6734571
DN30222_c0_g1_i1_2	3.293293	0.5034427
DN30234_c0_g1_i1_1	0	3.00024
DN30240_c0_g1_i1_2	1.186636	0.2981277
DN30247_c0_g1_i1_1	2.237096	2.183689
DN30253_c0_g1_i1_1	1.232275	4.024396
DN30255_c0_g1_i1_2	2.242416	1.453671
DN30256_c0_g1_i1_2	2.444278	1.061076
DN30259_c0_g1_i1_1	0.3133956	0.9434333
DN30262_c0_g1_i1_1	36.04187	17.7718
DN30263_c0_g1_i1_1	155.539	127.7364
DN30264_c0_g1_i1_1	2.042667	2.299154
DN30270_c0_g1_i1_1	10.45345	7.019884
DN30270_c0_g1_i1_2	4.446028	2.373482
DN30273_c0_g1_i1_2	1.852488	0.2862104
DN30274_c0_g1_i1_2	2.003393	1.751866
DN30276_c0_g1_i1_2	1.043473	0

DN30277_c0_g1_i1_2	3.449529	1.881642
DN30280_c0_g1_i1_1	1.785102	3.333001
DN30284_c0_g1_i1_1	5.997996	6.26268
DN3028_c0_g1_i1_2	2.346125	0
DN30299_c0_g1_i1_2	3.885362	0
DN30304_c0_g1_i1_1	0.8801448	2.42809
DN30305_c0_g1_i1_2	0.5374099	2.689986
DN30313_c0_g1_i1_2	1.087508	0.5037614
DN30314_c0_g1_i1_2	1.477861	1.127413
DN30316_c0_g1_i1_2	4.700522	3.423339
DN30317_c0_g1_i1_2	2.847266	2.01833
DN30319_c0_g1_i1_1	0.653937	0.768917
DN30320_c0_g1_i1_1	0.9684851	0.625735
DN30326_c0_g1_i1_2	7.701348	7.527623
DN30327_c0_g1_i1_2	8.91332	3.001148
DN30330_c0_g1_i1_2	2.179449	0.6324345
DN30331_c0_g1_i1_1	2.866542	5.127932
DN30337_c0_g1_i1_1	1.141368	1.618721
DN30339_c0_g1_i1_1	8.978823	9.752864
DN30342_c0_g1_i1_1	0.9308016	0.5546825
DN30345_c0_g1_i1_2	162.3845	165.0467
DN30346_c0_g1_i1_1	1.676829	2.602024
DN30348_c0_g1_i1_1	0.129303	0.3516213
DN30348_c0_g1_i1_2	0.9841405	0.7935787

DN30350_c0_g1_i1_2	2.749252	11.88682
DN30354_c0_g1_i1_1	7.805434	4.621297
DN30354_c0_g1_i1_2	1.092194	0.4463846
DN30356_c0_g1_i1_2	4.886414	1.546487
DN30360_c0_g1_i1_1	0	1.093255
DN30360_c0_g1_i1_2	0.6915821	0.09779282
DN30361_c0_g1_i1_1	27.40303	28.19302
DN30365_c0_g1_i1_1	0.374629	0.6516719
DN30365_c0_g1_i1_2	0.6679125	0.8131489
DN30367_c0_g1_i1_1	94.04061	26.44322
DN30367_c0_g1_i1_2	2.060087	0
DN30370_c0_g1_i1_1	0.09344362	1.756666
DN30377_c0_g1_i1_2	1.649079	0.4702611
DN30378_c0_g1_i1_2	10.848	6.81837
DN30379_c0_g1_i1_2	1.764158	0.565047
DN30384_c0_g1_i1_1	1.87392	1.523893
DN30386_c0_g1_i1_1	0.2238254	1.190234
DN30387_c0_g1_i1_1	1.538593	1.758379
DN30389_c0_g1_i1_1	0.4100438	0.632623
DN30394_c0_g1_i1_2	1.256052	0.3692138
DN30397_c0_g1_i1_2	21.20372	7.208016
DN3039_c0_g1_i1_2	0	0
DN30402_c0_g1_i1_1	1.017085	0.7215621
DN30408_c0_g1_i1_1	2.48671	1.15454

DN30409_c0_g1_i1_1	1.685773	4.086379
DN30413_c0_g1_i1_1	0.8090013	1.282473
DN30413_c0_g1_i1_2	2.342048	0.839498
DN30414_c0_g1_i1_1	1.338555	1.409054
DN30417_c0_g1_i1_1	1.334618	1.690651
DN30418_c0_g1_i1_1	0.7561832	1.129156
DN30421_c0_g1_i1_1	0.6704552	4.489977
DN30422_c0_g1_i1_2	2.77006	1.120558
DN30425_c0_g1_i1_2	3.7826	0.7325704
DN30427_c0_g1_i1_2	0	0
DN30431_c0_g1_i1_2	0	0
DN30433_c0_g1_i1_1	0	1.752531
DN30436_c0_g1_i1_1	1.957684	4.043805
DN30438_c0_g1_i1_2	0.1936673	0.5737496
DN30443_c0_g1_i1_2	1.438155	1.176441
DN30446_c0_g1_i1_2	1.275928	0
DN30453_c0_g1_i1_1	0.4379455	0.5126712
DN30456_c0_g1_i1_1	2.58597	1.616405
DN30458_c0_g1_i1_1	6.911309	4.520281
DN30458_c0_g1_i1_2	0.6478424	0.32781
DN30462_c0_g1_i1_1	0.07374703	2.343399
DN30463_c0_g1_i1_1	2.572519	1.474141
DN30463_c0_g1_i1_2	1.452699	0.795397
DN30468_c0_g1_i1_1	1.537169	2.533828

DN30469_c0_g1_i1_1	1.517273	13.93041
DN30470_c0_g1_i1_1	1.485685	1.609261
DN30473_c0_g1_i1_2	1.363967	3.365437
DN30476_c0_g1_i1_1	1.447017	4.167569
DN30478_c0_g1_i1_2	2.434753	0.2721162
DN30479_c0_g1_i1_1	0.4329595	1.455354
DN30480_c0_g1_i1_2	3.125402	1.375249
DN30485_c0_g1_i1_2	1.825342	0.9644753
DN30488_c0_g1_i1_2	5.586986	3.457902
DN30491_c0_g1_i1_2	6.202399	3.340836
DN30492_c0_g1_i1_2	0.2454765	0.6407227
DN30495_c0_g1_i1_1	0.7863242	2.526078
DN30502_c0_g1_i1_1	0.3792662	0.2199463
DN30503_c0_g1_i1_2	1.522609	1.201514
DN30505_c0_g1_i1_1	0.8192702	1.102361
DN30507_c0_g1_i1_1	0.1774706	5.676362
DN30507_c0_g1_i1_2	2.553286	1.313438
DN30509_c0_g1_i1_2	2.605614	85.14427
DN30516_c0_g1_i1_2	1.271068	0.9789103
DN30517_c0_g1_i1_1	0.644819	0.2547963
DN30520_c0_g1_i1_2	9.347899	11.17065
DN30521_c0_g1_i1_2	3.574001	2.21639
DN30522_c0_g1_i1_1	0.5421992	1.91274
DN30528_c0_g1_i1_2	2.147305	1.266916

DN30529_c0_g1_i1_1	0.4850288	3.693204
DN30530_c0_g1_i1_1	3.890257	1.279016
DN30538_c0_g1_i1_1	0.9314745	1.729368
DN30542_c0_g1_i1_2	1.112992	1.163596
DN30543_c0_g1_i1_2	1.61397	0.7890258
DN30545_c0_g1_i1_2	2.927488	0.5341414
DN30548_c0_g1_i1_2	2.44E-05	0.4042714
DN3054_c0_g1_i1_2	0.48878	0.4447909
DN30550_c0_g1_i1_1	0.1635027	1.669236
DN30553_c0_g1_i1_2	0.7114533	0
DN30554_c0_g1_i1_2	0.1630632	0
DN30556_c0_g1_i1_1	0.4446379	2.57093
DN30557_c0_g1_i1_2	0	0
DN30563_c0_g1_i1_2	0.6264295	0.8181176
DN30564_c0_g1_i1_2	3.218721	1.001926
DN30574_c0_g1_i1_2	0.8928634	1.316009
DN30576_c0_g1_i1_1	0.5932315	0.7085533
DN30579_c0_g1_i1_1	1.337285	8.544404
DN30585_c0_g1_i1_1	3.094094	4.556607
DN30586_c0_g1_i1_1	0.8957475	1.108212
DN30590_c0_g1_i1_2	0	0
DN30598_c0_g1_i1_2	1.993534	2.37647
DN30599_c0_g1_i1_1	0	0
DN305_c0_g1_i1_2	0	0

DN30602_c0_g1_i1_2	0.5062844	0
DN30605_c0_g1_i1_2	1.914124	5.326491
DN30607_c0_g1_i1_2	0	0
DN30615_c0_g1_i1_2	0.9178066	0.3920852
DN30618_c0_g1_i1_2	0	0
DN3061_c0_g1_i1_1	4.500534	5.520239
DN30620_c0_g1_i1_1	0.7887876	1.321587
DN30624_c0_g1_i1_1	1.712311	1.68432
DN30624_c0_g1_i1_2	15.4189	18.86479
DN30626_c0_g1_i1_1	2.262718	2.880404
DN30629_c0_g1_i1_1	1.09986	1.324222
DN30629_c0_g1_i1_2	0.7821774	0.4085045
DN30631_c0_g1_i1_1	0.1596578	2.136977
DN30632_c0_g1_i1_2	19.58967	69.37392
DN30635_c0_g1_i1_1	1.802005	6.039397
DN30635_c0_g1_i1_2	3.52613	0.5927673
DN30636_c0_g1_i1_1	12.54309	8.093405
DN30636_c0_g1_i1_2	0.2991768	0.1104376
DN30637_c0_g1_i1_1	13.0752	7.398651
DN30637_c0_g1_i1_2	0.480024	1.321596
DN30638_c0_g1_i1_1	6.279786	4.299616
DN30644_c0_g1_i1_1	8.743714	5.627157
DN30647_c0_g1_i1_2	5.84728	1.311464
DN30648_c0_g1_i1_1	0.4597728	0.2089147

DN30650_c0_g1_i1_1	2.177476	4.120263
DN30652_c0_g1_i1_2	2.21977	0.7607702
DN30654_c0_g1_i1_2	2.093863	1.828136
DN30656_c0_g1_i1_1	0.3343787	1.318592
DN30656_c0_g1_i1_2	4.085611	1.288439
DN30657_c0_g1_i1_1	0.9239559	2.478982
DN30657_c0_g1_i1_2	3.074497	0.6489436
DN30658_c0_g1_i1_1	2.826639	1.48E-06
DN30659_c0_g1_i1_2	2.846755	0.1287297
DN30660_c0_g1_i1_1	4.576083	7.67781
DN30663_c0_g1_i1_1	0.1365314	1.426753
DN30665_c0_g1_i1_1	1.013007	2.889041
DN30665_c0_g1_i1_2	0.9415455	1.223219
DN30666_c0_g1_i1_1	0.5020498	1.075433
DN30667_c0_g1_i1_1	1.042128	1.010653
DN30669_c0_g1_i1_1	0.837595	1.034312
DN30670_c0_g1_i1_2	2.062093	1.518783
DN30671_c0_g1_i1_1	0.7437581	1.276646
DN30672_c0_g1_i1_2	1.14043	5.302668
DN30678_c0_g1_i1_2	0.1532627	2.206493
DN30679_c0_g1_i1_2	1.558521	0.8325318
DN30680_c0_g1_i1_1	3.086913	5.614265
DN30682_c0_g1_i1_1	0.3050032	0.5584114
DN30692_c0_g1_i1_2	2.646607	2.442171

DN30694_c0_g1_i1_2	1.29144	0.4046466
DN30697_c0_g1_i1_2	0.5890973	0
DN30698_c0_g1_i1_1	15.85232	8.828001
DN30699_c0_g1_i1_1	0.4481552	2.176705
DN30699_c0_g1_i1_2	1.849296	1.258867
DN306_c0_g1_i1_2	29.31422	13.34182
DN30701_c0_g1_i1_1	0.4108772	1.223126
DN30703_c0_g1_i1_1	3.044118	10.13339
DN30703_c0_g1_i1_2	10.24079	4.383849
DN30709_c0_g1_i1_1	0.6521394	0.9105984
DN30712_c0_g1_i1_2	0.9772674	0.4434535
DN30715_c0_g1_i1_2	0.427259	0.1536401
DN30717_c0_g1_i1_2	1.300993	0
DN30718_c0_g1_i1_1	6.203724	4.707108
DN30723_c0_g1_i1_1	1.373143	2.309357
DN30723_c0_g1_i1_2	3.179429	0.3761148
DN30726_c0_g1_i1_1	4.435521	3.464357
DN30729_c0_g1_i1_1	1.554891	3.176414
DN30730_c0_g1_i1_2	0.4033693	2.447985
DN30732_c0_g1_i1_2	0.8162775	0
DN30738_c0_g1_i1_2	0.7125866	1.228867
DN30739_c0_g1_i1_1	2.265119	4.328468
DN30739_c0_g1_i1_2	2.460859	1.452883
DN30741_c0_g1_i1_2	1.338149	2.195987

DN30742_c0_g1_i1_1	1.09702	1.949905
DN30743_c0_g1_i1_1	2.739487	1.162201
DN30745_c0_g1_i1_1	1.497436	0.5251024
DN30745_c0_g1_i1_2	0	3.75973
DN30746_c0_g1_i1_1	0.7983212	1.599521
DN30747_c0_g1_i1_1	1.984214	2.028981
DN30751_c0_g1_i1_1	0.8005617	0.8253501
DN30754_c0_g1_i1_2	4.672352	0
DN30756_c0_g1_i1_2	0.9228098	0
DN30758_c0_g1_i1_1	1.611784	0.8918313
DN30759_c0_g1_i1_2	0.7899168	0.2839212
DN30760_c0_g1_i1_2	3.577532	0.8773896
DN30763_c0_g1_i1_1	0.7273096	1.717303
DN30767_c0_g1_i1_1	0	0
DN30770_c0_g1_i1_2	1.043904	0.275671
DN30775_c0_g1_i1_1	1.102664	0.5909051
DN30776_c0_g1_i1_1	3.077624	3.389564
DN30778_c0_g1_i1_1	0.1271555	0.01165275
DN30778_c0_g1_i1_2	1.349677	1.165749
DN30779_c0_g1_i1_1	1.202066	2.166971
DN30779_c0_g1_i1_2	1.920524	3.450605
DN30783_c0_g1_i1_2	3.744784	0
DN30784_c0_g1_i1_2	8.683812	0
DN30786_c0_g1_i1_2	1.076261	0.5369212

DN30787_c0_g1_i1_2	1.435322	2.805414
DN30789_c0_g1_i1_1	0.3550213	1.182843
DN30789_c0_g1_i1_2	0.5051851	0
DN3078_c0_g1_i1_1	3.898245	1.803632
DN30792_c0_g1_i1_2	2.214972	0.9752708
DN30795_c0_g1_i1_1	2.093668	3.041894
DN30797_c0_g1_i1_2	3.112549	0
DN30798_c0_g1_i1_2	0.397099	0.4522959
DN30801_c0_g1_i1_1	10.85093	3.657138
DN30803_c0_g1_i1_1	0.5465065	0.9951075
DN30803_c0_g1_i1_2	6.729031	1.441009
DN30806_c0_g1_i1_1	0.788434	2.018499
DN30809_c0_g1_i1_1	2.301943	2.441125
DN30809_c0_g1_i1_2	3.155676	0
DN30811_c0_g1_i1_2	0.7828904	1.39824
DN30812_c0_g1_i1_2	0	0
DN30813_c0_g1_i1_2	2.653357	4.292243
DN30815_c0_g1_i1_1	1.348398	0.4004449
DN30816_c0_g1_i1_1	1.153615	0.6395645
DN30817_c0_g1_i1_1	0.8254152	1.077146
DN30826_c0_g1_i1_2	2.353907	1.437026
DN30828_c0_g1_i1_2	23.52285	30.14777
DN30830_c0_g1_i1_2	14.91661	15.47948
DN30832_c0_g1_i1_1	0	2.602

DN30832_c0_g1_i1_2	12.25408	11.73047
DN30833_c0_g1_i1_1	0.8086072	1.199648
DN30836_c0_g1_i1_2	0.6232369	0.9131447
DN30842_c0_g1_i1_2	0	0
DN30847_c0_g1_i1_2	1.754156	0.3577978
DN30848_c0_g1_i1_2	0	0.3539622
DN30850_c0_g1_i1_2	3.616829	0.7035821
DN30851_c0_g1_i1_1	0.4728279	16.5332
DN30855_c0_g1_i1_1	0.0936397	1.565623
DN30855_c0_g1_i1_2	3.101195	3.78448
DN30856_c0_g1_i1_2	3.639604	0
DN30857_c0_g1_i1_2	2.526912	2.426379
DN3085_c0_g1_i1_2	0.9650497	0
DN30860_c0_g1_i1_1	4.597894	0.921496
DN30866_c0_g1_i1_2	2.233551	1.954639
DN30867_c0_g1_i1_1	0	9.876296
DN30868_c0_g1_i1_1	0	0.5568507
DN30869_c0_g1_i1_1	0.768471	0.7961246
DN30872_c0_g1_i1_2	19.64639	21.39845
DN30877_c0_g1_i1_1	0.5669227	0.1936853
DN30879_c0_g1_i1_1	0.8260311	2.900986
DN3087_c0_g1_i1_2	4.060625	0.8121391
DN30881_c0_g1_i1_1	1.538809	1.273253
DN30885_c0_g1_i1_1	0.1403407	0.2578834

DN30891_c0_g1_i1_1	1.333331	2.383457
DN30893_c0_g1_i1_1	0.3882899	0.8145813
DN30895_c0_g1_i1_1	0.3106884	1.460057
DN30898_c0_g1_i1_1	0.3169912	0.238372
DN30901_c0_g1_i1_1	0.7247234	2.797028
DN30904_c0_g1_i1_1	0.2564155	0.2855061
DN30907_c0_g1_i1_1	0.2901443	0.2255271
DN30908_c0_g1_i1_1	1.570802	0.7407231
DN30912_c0_g1_i1_1	7.113013	5.176598
DN30915_c0_g1_i1_1	0.5959852	1.325964
DN30916_c0_g1_i1_2	1.597714	0.5030967
DN30919_c0_g1_i1_1	0	0.13369
DN30923_c0_g1_i1_1	0.4221585	2.417228
DN30924_c0_g1_i1_1	1.174857	6.567967
DN30929_c0_g1_i1_1	8.586886	28.62387
DN3092_c0_g1_i1_2	0.7310358	0.511475
DN30934_c0_g1_i1_1	3.343064	1.090596
DN30936_c0_g1_i1_1	0	1.226636
DN30950_c0_g1_i1_1	0	0.9272494
DN30951_c0_g1_i1_2	3.735273	1.282603
DN30953_c0_g1_i1_2	0.4734095	3.539088
DN30955_c0_g1_i1_1	3.199999	2.592689
DN30956_c0_g1_i1_2	7.950446	2.031682
DN30959_c0_g1_i1_1	0.6283329	0.6871299

DN3095_c0_g1_i1_2	4.979038	1.630747
DN30963_c0_g1_i1_2	0.9756003	0.5909868
DN30966_c0_g1_i1_1	0	0
DN30969_c0_g1_i1_1	0.3284681	0.663377
DN3096_c0_g1_i1_1	0.122042	0.2975391
DN3096_c0_g1_i2_2	7.170136	0.9071133
DN30970_c0_g1_i1_1	4.104772	3.464645
DN30975_c0_g1_i1_1	1.677644	1.070083
DN30977_c0_g1_i1_1	0.9082083	0.9608281
DN30978_c0_g1_i1_1	15.48384	8.818624
DN30979_c0_g1_i1_2	1.03216	2.17624
DN30993_c0_g1_i1_1	9.2759	28.4799
DN30994_c0_g1_i1_1	0.8842571	1.929218
DN30994_c0_g1_i1_2	4.507297	1.602344
DN30996_c0_g1_i1_1	6.290909	16.87046
DN30999_c0_g1_i1_1	1.53269	3.118113
DN31005_c0_g1_i1_1	1.833929	0.8723704
DN31007_c0_g1_i1_1	0.07453525	1.48095
DN31007_c0_g1_i1_2	0.9106483	0.518992
DN31009_c0_g1_i1_1	2.379704	2.307964
DN31010_c0_g1_i1_2	2.39225	0.06165734
DN31013_c0_g1_i1_1	0.6624079	2.28638
DN31016_c0_g1_i1_1	0	1.669177
DN31019_c0_g1_i1_1	29.90888	6.735624

DN31027_c0_g1_i1_1	1.143465	2.568806
DN31029_c0_g1_i1_2	11.64686	6.342843
DN31032_c0_g1_i1_2	11.72757	2.197289
DN31037_c0_g1_i1_2	38.39183	44.71608
DN31039_c0_g1_i1_1	0.6400215	0.2101852
DN31049_c0_g1_i1_2	1.764989	0.5154774
DN31053_c0_g1_i1_1	249.5882	96.54338
DN31055_c0_g1_i1_1	0	2.356205
DN31057_c0_g1_i1_2	2.833501	2.572314
DN31059_c0_g1_i1_2	1.945908	0.3968776
DN31061_c0_g1_i1_1	1.057879	0.7989909
DN31062_c0_g1_i1_1	240.4588	178.7074
DN31063_c0_g1_i1_1	1.009686	1.010742
DN31064_c0_g1_i1_2	1.572116	0.5952405
DN31065_c0_g1_i1_1	1.304884	5.913151
DN31068_c0_g1_i1_1	2.122487	2.189671
DN31069_c0_g1_i1_2	0.5699715	0.4576327
DN3106_c0_g1_i1_1	4.824252	2.145066
DN31070_c0_g1_i1_1	1.445887	1.090018
DN31076_c0_g1_i1_2	0.7345202	0.6222751
DN31078_c0_g1_i1_1	0.8631984	1.082893
DN31079_c0_g1_i1_1	11.67106	6.237187
DN31083_c0_g1_i1_1	1.325529	1.531045
DN31087_c0_g1_i1_1	1.568414	2.374768

DN3108_c0_g1_i1_1	1.769607	4.560064
DN31091_c0_g1_i1_2	295.3176	159.9898
DN31093_c0_g1_i1_2	0.2623119	0.2867413
DN31094_c0_g1_i1_2	3.917093	0.850476
DN31098_c0_g1_i1_1	1.24153	1.912202
DN31099_c0_g1_i1_1	2.504394	3.988595
DN3109_c0_g1_i1_2	128.6007	106.7675
DN31100_c0_g1_i1_1	0.8340734	1.079872
DN31104_c0_g1_i1_1	21.29846	12.12513
DN31107_c0_g1_i1_2	3.223828	1.516196
DN3110_c0_g1_i1_1	2.150592	2.123865
DN3110_c0_g1_i1_2	0.2015723	0.1911272
DN31110_c0_g1_i1_1	1.447692	2.022596
DN31113_c0_g1_i1_1	0.8603279	1.665128
DN31115_c0_g1_i1_2	0.7219546	0.09091731
DN31116_c0_g1_i1_1	0.3312907	0.8747914
DN31117_c0_g1_i1_1	1.224655	1.038017
DN31118_c0_g1_i1_2	11.55421	2.957107
DN31121_c0_g1_i1_1	3.356166	2.107986
DN31126_c0_g1_i1_1	0.8087176	0.8505561
DN31126_c0_g1_i1_2	0.737111	0.9088166
DN31130_c0_g1_i1_1	0.8846237	1.147267
DN31135_c0_g1_i1_2	0.4262733	0.2249136
DN31139_c0_g1_i1_2	0.9687081	0.329618

DN3113_c0_g1_i1_2	2.48922	1.590904
DN31140_c0_g1_i1_2	4.904682	3.635302
DN31141_c0_g1_i1_2	17.06814	29.21338
DN31143_c0_g1_i1_1	0.2942474	0.9403363
DN31145_c0_g1_i1_1	7.65778	3.896914
DN31146_c0_g1_i1_1	0.9025524	2.025896
DN31147_c0_g1_i1_2	1.761122	1.125438
DN31148_c0_g1_i1_1	3.074841	0.5429269
DN3114_c0_g1_i1_2	2.356688	0.6161367
DN31153_c0_g1_i1_2	14.61814	8.725417
DN31155_c0_g1_i1_1	0.9725232	1.616338
DN31156_c0_g1_i1_1	0	0.7502468
DN31157_c0_g1_i1_1	1.130676	0.2290921
DN31158_c0_g1_i1_1	0.1381954	2.673227
DN31160_c0_g1_i1_1	1.608552	1.597929
DN31165_c0_g1_i1_1	1.384926	1.59487
DN31166_c0_g1_i1_1	1.497035	0
DN31167_c0_g1_i1_2	2.25E-07	0.1555312
DN31168_c0_g1_i1_1	19.69483	12.48766
DN31169_c0_g1_i1_2	8.682507	2.835558
DN31173_c0_g1_i1_1	2.814544	5.030995
DN31174_c0_g1_i1_1	5.381488	38.62454
DN31176_c0_g1_i1_2	6.364158	2.955861
DN31178_c0_g1_i1_1	1.277974	2.643145

DN31179_c0_g1_i1_1	1.677306	3.515204
DN3117_c0_g1_i1_2	0	0
DN31180_c0_g1_i1_1	0.05375597	2.141766
DN31181_c0_g1_i1_1	1.628175	0.9058649
DN31185_c0_g1_i1_2	1.477344	1.738061
DN3118_c0_g1_i1_1	3.102507	2.177833
DN3118_c0_g2_i1_1	1.081718	1.144854
DN31191_c0_g1_i1_1	0.9302304	1.2316
DN31192_c0_g1_i1_2	1.154627	0
DN31193_c0_g1_i1_1	0	4.095333
DN31194_c0_g1_i1_1	0.9168456	0.6807702
DN31197_c0_g1_i1_1	39.19368	27.78672
DN31204_c0_g1_i1_1	1.513619	1.861199
DN31205_c0_g1_i1_1	0.04258456	0.7227395
DN31209_c0_g1_i1_2	2.21587	1.751453
DN31210_c0_g1_i1_1	0.1068687	0.4676039
DN31216_c0_g1_i1_1	8.069389	4.761455
DN31216_c0_g1_i1_2	2.960092	2.401742
DN31218_c0_g1_i1_1	6.265113	4.249411
DN31219_c0_g1_i1_2	1.345626	0.1682201
DN31226_c0_g1_i1_2	4.636762	0.6177533
DN3122_c0_g1_i1_2	4.239668	3.088371
DN31231_c0_g1_i1_2	0	0
DN31232_c0_g1_i1_2	3.030509	2.070424

DN31233_c0_g1_i1_1	0.5074574	1.761141
DN31234_c0_g1_i1_1	6.815207	2.854804
DN31235_c0_g1_i1_1	1.078838	1.93698
DN31237_c0_g1_i1_1	1.997171	1.658433
DN31237_c0_g1_i1_2	2.769485	0.6741138
DN31238_c0_g1_i1_1	6.486052	6.530755
DN3123_c0_g1_i2_1	1.910775	2.599567
DN31242_c0_g1_i1_1	1.070874	2.206517
DN31245_c0_g1_i1_2	6.935478	1.802542
DN31249_c0_g1_i1_1	1.965757	1.38353
DN3124_c0_g1_i1_1	1.109158	0.3427386
DN3124_c0_g1_i1_2	4.623365	1.122375
DN31256_c0_g1_i1_2	1.189116	1.92E-30
DN31260_c0_g1_i1_1	1.508754	1.048946
DN31261_c0_g1_i1_1	0.8321019	1.651968
DN31267_c0_g1_i1_1	2.423639	11.96093
DN31267_c0_g1_i1_2	166.7496	88.65362
DN31274_c0_g1_i1_2	6.356729	3.721872
DN31280_c0_g1_i1_1	0.2977809	0.6402198
DN31282_c0_g1_i1_1	1.711462	1.694375
DN31284_c0_g1_i1_1	0.1249473	1.20838
DN31290_c0_g1_i1_2	1.080344	0
DN31294_c0_g1_i1_1	4.384688	6.294825
DN31295_c0_g1_i1_1	0.2278926	0.9142559

DN31295_c0_g1_i1_2	0.3677435	0
DN31296_c0_g1_i1_1	0.4355557	0.2770914
DN31297_c0_g1_i1_1	0.6705995	0.2372879
DN31297_c0_g1_i1_2	4.360713	2.230106
DN31298_c0_g1_i1_1	7.211438	10.77062
DN312_c0_g2_i1_1	0.297429	1.327977
DN31309_c0_g1_i1_2	3.767696	2.422813
DN31310_c0_g1_i1_1	40.63166	46.45271
DN31312_c0_g1_i1_1	0	0
DN31313_c0_g1_i1_1	3.695243	4.54601
DN31319_c0_g1_i1_1	0	0.3428045
DN31320_c0_g1_i1_1	0.2565149	0.3551296
DN31326_c0_g1_i1_1	2.350058	1.005695
DN31328_c0_g1_i1_2	3.903137	3.116323
DN3132_c0_g1_i1_1	1.715457	0.4753892
DN31340_c0_g1_i1_2	81.8283	0.06722851
DN31341_c0_g1_i1_1	0.3822679	2.737153
DN31342_c0_g1_i1_1	5.148966	6.141615
DN31344_c0_g1_i1_2	3.284625	2.789055
DN31345_c0_g1_i1_1	3.089334	2.041816
DN31347_c0_g1_i1_1	0.1957278	0.595353
DN31348_c0_g1_i1_1	1.862679	17.02103
DN31350_c0_g1_i1_2	2.98897	0.9722886
DN31356_c0_g1_i1_2	155.3455	57.38798

DN31358_c0_g1_i1_1	0	0.4410241
DN31359_c0_g1_i1_2	86.30501	0.1311263
DN31364_c0_g1_i1_1	0.6693518	4.623936
DN31366_c0_g1_i1_1	0.5569233	2.486649
DN31367_c0_g1_i1_1	2.956595	1.273538
DN31373_c0_g1_i1_1	1.207577	0.9926586
DN31377_c0_g1_i1_1	0.6979156	1.307945
DN31377_c0_g1_i1_2	9.626648	3.61716
DN31380_c0_g1_i1_2	2.679434	0.6592052
DN31381_c0_g1_i1_1	0.6221958	0.2165732
DN31383_c0_g1_i1_1	1.483585	0.6118924
DN31386_c0_g1_i1_1	0.3063792	1.108713
DN31388_c0_g1_i1_1	1.701175	1.927678
DN31390_c0_g1_i1_1	2.378031	0.9923812
DN31393_c0_g1_i1_1	1.241057	1.894161
DN31395_c0_g1_i1_1	1.776599	1.830457
DN31396_c0_g1_i1_1	10.39559	10.83083
DN31398_c0_g1_i1_2	12.16988	7.578783
DN31404_c0_g1_i1_1	0.2516658	0.652691
DN31404_c0_g1_i1_2	3.326851	1.278313
DN31406_c0_g1_i1_1	2.617548	7.35295
DN31409_c0_g1_i1_1	0	2.754024
DN31416_c0_g1_i1_1	1.614586	0.8074463
DN31416_c0_g1_i1_2	3.268262	2.182929

DN31419_c0_g1_i1_1	1.596535	7.169269
DN31422_c0_g1_i1_1	7.932994	6.495008
DN31422_c0_g1_i1_2	1.447714	0.6330841
DN31423_c0_g1_i1_1	0.8329688	1.138106
DN31427_c0_g1_i1_2	3.923735	1.6581
DN31431_c0_g1_i1_1	3.444159	4.843061
DN31433_c0_g1_i1_1	17.15795	9.450081
DN31434_c0_g1_i1_1	0.9062829	1.416649
DN31436_c0_g1_i1_1	1.329776	0.6567221
DN31443_c0_g1_i1_1	0.7566272	0.581155
DN31447_c0_g1_i1_1	0	2.20473
DN31449_c0_g1_i1_1	160.0646	174.8804
DN31459_c0_g1_i1_1	1.441274	1.481478
DN31460_c0_g1_i1_1	0.5679983	0.8964379
DN31468_c0_g1_i1_1	0.7758709	1.644735
DN31469_c0_g1_i1_1	0.8492563	0
DN31472_c0_g1_i1_1	0	0
DN31473_c0_g1_i1_2	4.375389	5.527393
DN31476_c0_g1_i1_2	2.558049	0.4937559
DN31478_c0_g1_i1_1	0.6082962	2.976931
DN31478_c0_g1_i1_2	1.598893	1.52623
DN31479_c0_g1_i1_1	4.323882	0.6592152
DN3147_c0_g1_i1_1	1.089674	0.8853388
DN31482_c0_g1_i1_1	1.879555	2.368512

DN31483_c0_g1_i1_1	0.6777082	1.705036
DN31484_c0_g1_i1_1	76.01374	65.27329
DN31484_c0_g1_i1_2	2.910481	0.4788606
DN31485_c0_g1_i1_1	4.060967	3.277212
DN31486_c0_g1_i1_1	3.821997	2.042262
DN31487_c0_g1_i1_2	9.457089	17.19161
DN31489_c0_g1_i1_1	1.612313	0.8926741
DN31495_c0_g1_i1_1	0.5284446	1.509508
DN3149_c0_g1_i1_2	1.536399	3.30847
DN3149_c0_g2_i1_2	1.551629	2.106726
DN31502_c0_g1_i1_2	0.9757625	0.4049476
DN31503_c0_g1_i1_1	0.1318749	1.758032
DN31504_c0_g1_i1_1	0.4690722	0
DN31505_c0_g1_i1_1	0.6642318	0.5534464
DN31509_c0_g1_i1_1	19.055	5.248173
DN31510_c0_g1_i1_2	1.423473	11.2643
DN31511_c0_g1_i1_1	1.540434	1.132425
DN31513_c0_g1_i1_1	0.07479611	0.4951644
DN31519_c0_g1_i1_1	2074.254	94.16646
DN3151_c0_g1_i1_1	1.04697	2.028821
DN31520_c0_g1_i1_1	0.5661384	3.861384
DN31521_c0_g1_i1_1	2.428132	4.126138
DN31527_c0_g1_i1_1	0.06715067	0.8534622
DN31527_c0_g1_i1_2	13.01603	168.2329

DN31528_c0_g1_i1_1	0.1742843	1.521205
DN31528_c0_g1_i1_2	2.864096	1.084847
DN31531_c0_g1_i1_1	0.3586258	0.9667832
DN31532_c0_g1_i1_1	11.18773	10.41474
DN31533_c0_g1_i1_1	3.283495	2.498861
DN31535_c0_g1_i1_1	0.8146164	1.631241
DN31538_c0_g1_i1_1	0.8360097	0.2855128
DN31540_c0_g1_i1_1	7.645907	15.10075
DN31542_c0_g1_i1_1	0	0.5990521
DN31545_c0_g1_i1_1	0.6831583	0.6088601
DN31547_c0_g1_i1_2	0.7591935	0.4417991
DN31553_c0_g1_i1_1	2.17954	3.893744
DN31559_c0_g1_i1_1	0.9727711	2.1335
DN3155_c0_g1_i2_2	6.450467	3.502826
DN31561_c0_g1_i1_2	7.062002	6.219443
DN31562_c0_g1_i1_1	2.861428	9.346599
DN31562_c0_g1_i1_2	5.479741	2.865938
DN31567_c0_g1_i1_1	0.909763	1.425097
DN31568_c0_g1_i1_1	0.1728243	3.474609
DN31569_c0_g1_i1_2	7.308859	3.892241
DN3156_c0_g1_i1_2	1.524299	2.909891
DN31571_c0_g1_i1_1	1.618723	3.49027
DN31573_c0_g1_i1_1	2.057321	2.855335
DN31574_c0_g1_i1_1	1.885524	0.8243425

DN31574_c0_g1_i1_2	0.4572295	0
DN31576_c0_g1_i1_1	0.1628142	1.74909
DN31579_c0_g1_i1_2	6.146477	6.098323
DN31581_c0_g1_i1_1	2.160486	12.40115
DN31581_c0_g1_i1_2	0.16453	0
DN31584_c0_g1_i1_1	0.2844199	0.9662009
DN31586_c0_g1_i1_1	3.028009	3.241373
DN31589_c0_g1_i1_2	2.073941	7.386863
DN31591_c0_g1_i1_1	0.8901384	1.470577
DN31595_c0_g1_i1_1	0.413787	1.330838
DN31599_c0_g1_i1_1	0.2460274	0.9629438
DN31606_c0_g1_i1_1	0.4158023	1.045899
DN31609_c0_g1_i1_1	1.04065	0.2879328
DN31614_c0_g1_i1_1	0	0
DN31615_c0_g1_i1_2	0.1526225	0.6687585
DN31617_c0_g1_i1_2	1.128032	0
DN31621_c0_g1_i1_1	1.382502	0.3304581
DN31622_c0_g1_i1_1	8.808251	102.1595
DN31624_c0_g1_i1_2	2.835044	6.371579
DN31628_c0_g1_i1_1	5.760649	3.860238
DN31628_c0_g1_i1_2	2.770483	0
DN31629_c0_g1_i1_1	2.208714	9.832867
DN3162_c0_g1_i1_1	2.080379	0.6695272
DN3162_c0_g1_i1_2	1.092122	0.1512824

DN3162_c0_g2_i1_2	4.041411	1.729801
DN31630_c0_g1_i1_2	1.829934	1.089918
DN31631_c0_g1_i1_1	0.8606929	0.8564575
DN31631_c0_g1_i1_2	1.064874	1.801806
DN31632_c0_g1_i1_1	1.085433	0.3030992
DN31634_c0_g1_i1_1	18.30367	9.370899
DN31635_c0_g1_i1_1	0.3028273	2.463206
DN31636_c0_g1_i1_1	0.9635163	2.445959
DN31640_c0_g1_i1_1	0.2618081	1.83813
DN31642_c0_g1_i1_1	0	1.142452
DN31644_c0_g1_i1_1	0.5833538	1.383943
DN31646_c0_g1_i1_1	3.006526	5.284142
DN31647_c0_g1_i1_1	2.069746	0
DN31648_c0_g1_i1_1	0.716769	3.647918
DN3164_c0_g1_i1_1	0.2427621	0.6950539
DN31654_c0_g1_i1_1	0	0
DN31655_c0_g1_i1_2	8.993451	2.354569
DN31656_c0_g1_i1_1	2.525575	2.420193
DN31658_c0_g1_i1_1	1.655418	1.132688
DN31659_c0_g1_i1_2	0.6519356	2.04E-11
DN31661_c0_g1_i1_1	6.492451	0.8420509
DN31661_c0_g1_i1_2	11.97372	12.07143
DN31663_c0_g1_i1_1	0.5620777	0.9606826
DN31664_c0_g1_i1_1	5.794262	3.70374

DN31665_c0_g1_i1_1	0.4255621	0.1490951
DN31669_c0_g1_i1_1	1.356108	3.421426
DN31671_c0_g1_i1_1	3.856208	4.801147
DN31672_c0_g1_i1_2	0.9908417	0.4756978
DN31673_c0_g1_i1_1	0.2735232	1.155468
DN31673_c0_g1_i1_2	1.152614	0.5410016
DN31674_c0_g1_i1_1	1.748192	2.281986
DN31675_c0_g1_i1_1	2.984221	10.07715
DN31677_c0_g1_i1_1	1.715758	2.860064
DN31678_c0_g1_i1_2	5.158454	4.91356
DN31682_c0_g1_i1_1	1.450764	2.843651
DN31683_c0_g1_i1_2	2.181143	1.861528
DN31684_c0_g1_i1_2	0.5568188	0.07094077
DN31685_c0_g1_i1_1	0.7788656	1.494948
DN31688_c0_g1_i1_1	0.1989457	0.6540191
DN31690_c0_g1_i1_1	8.14522	3.25056
DN31691_c0_g1_i1_2	1.857276	1.209617
DN31694_c0_g1_i1_2	6.533761	2.231205
DN31695_c0_g1_i1_2	8.875577	7.226057
DN31696_c0_g1_i1_1	0.6994714	1.297673
DN31696_c0_g1_i1_2	1.29364	0.5029981
DN31697_c0_g1_i1_2	1.894153	1.400484
DN31698_c0_g1_i1_1	1.853691	3.371128
DN31699_c0_g1_i1_2	1.692031	0.5243519

DN31700_c0_g1_i1_2	1.296827	1.145336
DN31701_c0_g1_i1_1	3.169205	3.013758
DN31703_c0_g1_i1_1	5.44632	2.781567
DN31705_c0_g1_i1_1	0.8207925	2.293846
DN31706_c0_g1_i1_1	0.5450415	0.7320765
DN31706_c0_g1_i1_2	13.81755	1.870192
DN31708_c0_g1_i1_1	0.7063363	2.015813
DN31711_c0_g1_i1_1	0	0.9002145
DN31712_c0_g1_i1_1	1.306893	2.095883
DN31714_c0_g1_i1_2	66.36064	10.31405
DN31718_c0_g1_i1_1	0.9280267	4.34037
DN31719_c0_g1_i1_1	0	2.581714
DN3171_c0_g1_i1_2	2.892849	2.375497
DN31722_c0_g1_i1_1	1.108565	2.892709
DN31722_c0_g1_i1_2	0.8649651	0.6193203
DN31723_c0_g1_i1_2	0.6944935	0.9569031
DN31724_c0_g1_i1_1	0.6259499	1.237715
DN31725_c0_g1_i1_2	2.24741	0.55952
DN31726_c0_g1_i1_2	1.366582	1.098998
DN31729_c0_g1_i1_1	1.122309	1.985688
DN31732_c0_g1_i1_2	1.271175	0.8260329
DN31733_c0_g1_i1_1	0.2565483	3.096826
DN31734_c0_g1_i1_1	1.575637	4.80606
DN31739_c0_g1_i1_2	5.077617	8.968561

DN3173_c0_g1_i1_1	0.2180359	1.395666
DN31744_c0_g1_i1_2	2.143464	0.3702006
DN31746_c0_g1_i1_1	0.3497361	1.057509
DN31747_c0_g1_i1_2	1.465438	0.5550194
DN31748_c0_g1_i1_1	3.063182	3.426934
DN31751_c0_g1_i1_1	0.5691655	0.4599241
DN31753_c0_g1_i1_1	0.9249085	2.567796
DN31754_c0_g1_i1_1	1.203399	0.0007492
DN31756_c0_g1_i1_1	1.774163	1.473802
DN31759_c0_g1_i1_1	1.723136	2.0127
DN31759_c0_g1_i1_2	2.123118	0.8077872
DN31760_c0_g1_i1_2	4.26889	0.2282944
DN31763_c0_g1_i1_1	0.6066215	3.272233
DN31764_c0_g1_i1_1	0.2145812	0.4749495
DN31767_c0_g1_i1_2	52.51133	72.51504
DN31769_c0_g1_i1_1	3.729776	5.736135
DN31770_c0_g1_i1_1	0	1.818852
DN31773_c0_g1_i1_1	2.43257	0
DN31775_c0_g1_i1_1	2.252502	2.072251
DN31776_c0_g1_i1_1	0.1514471	0.9432308
DN31778_c0_g1_i1_2	1.374289	1.144143
DN31782_c0_g1_i1_2	1.736902	1.423946
DN31783_c0_g1_i1_2	7.432067	4.399517
DN31786_c0_g1_i1_1	3.310016	3.334832

DN31788_c0_g1_i1_1	13.72506	5.066917
DN3178_c0_g1_i1_1	0.8400948	0.9564693
DN31790_c0_g1_i1_1	1.116128	1.638317
DN31790_c0_g1_i1_2	2.388189	2.49665
DN31791_c0_g1_i1_1	0.3350157	1.916121
DN31794_c0_g1_i1_1	0.6491063	0.9190117
DN31794_c0_g1_i1_2	0.5376271	0.6135894
DN31795_c0_g1_i1_1	0.6349768	0.6067971
DN31796_c0_g1_i1_2	8.930426	12.42027
DN31797_c0_g1_i1_1	0.5634879	1.596025
DN31799_c0_g1_i1_1	20.31437	20.34054
DN31802_c0_g1_i1_2	3.084904	1.472746
DN31803_c0_g1_i1_1	0.6224418	1.129672
DN31804_c0_g1_i1_2	1.261457	0.2622201
DN31806_c0_g1_i1_1	0.8753419	1.23312
DN31807_c0_g1_i1_1	3.119349	4.479969
DN3180_c0_g1_i1_1	0.9765294	0.410897
DN31810_c0_g1_i1_2	0.6958007	1.048064
DN31812_c0_g1_i1_1	0.3342909	0.8586462
DN31814_c0_g1_i1_1	0	0
DN31815_c0_g1_i1_1	1.755516	2.128943
DN31816_c0_g1_i1_1	0.8497401	1.746818
DN31817_c0_g1_i1_1	0.5583788	0
DN31818_c0_g1_i1_1	7.180458	11.04048

DN31822_c0_g1_i1_2	2.269974	0
DN31826_c0_g1_i1_2	1.33132	4.390031
DN31833_c0_g1_i1_2	0.8100167	0.6678174
DN31835_c0_g1_i1_1	0.2346558	0
DN3183_c0_g1_i1_1	0.3645448	1.002211
DN31840_c0_g1_i1_1	0.6690187	2.098333
DN31841_c0_g1_i1_1	1.31688	2.301139
DN31842_c0_g1_i1_1	1.744229	12.76612
DN31843_c0_g1_i1_1	1.57711	2.309076
DN31846_c0_g1_i1_1	0	0.3620901
DN31846_c0_g1_i1_2	4.919287	2.411242
DN31853_c0_g1_i1_1	0.710552	0.6153353
DN31854_c0_g1_i1_1	0.3462436	0.4964717
DN31855_c0_g1_i1_2	0.934186	1.100432
DN31857_c0_g1_i1_1	0.3888117	1.712705
DN31858_c0_g1_i1_1	0.3312664	0.6256588
DN31858_c0_g1_i1_2	5.321453	3.946858
DN3185_c0_g1_i1_2	1.028262	0.4976982
DN31860_c0_g1_i1_1	0.3786716	0.4616585
DN31860_c0_g1_i1_2	2.85353	1.290166
DN31863_c0_g1_i1_1	2.055164	1.799577
DN31864_c0_g1_i1_1	1.452118	0
DN31867_c0_g1_i1_1	1.83045	1.14511
DN31870_c0_g1_i1_1	0.9450665	0.6064505

DN31872_c0_g1_i1_1	0.2637238	1.096902
DN31875_c0_g1_i1_2	0.6881294	0
DN31879_c0_g1_i1_1	0	0.482346
DN31880_c0_g1_i1_1	0.2588249	0.5457086
DN31884_c0_g1_i1_2	0	0
DN31887_c0_g1_i1_1	1.661862	0.7958927
DN31887_c0_g1_i1_2	1.516781	0.3933602
DN31889_c0_g1_i1_1	0.3143105	0
DN31894_c0_g1_i1_1	6.18752	9.579643
DN31897_c0_g1_i1_1	29.70899	40.78548
DN31898_c0_g1_i1_1	0.1948036	0.7772274
DN31899_c0_g1_i1_1	1.261142	0.2825633
DN318_c0_g1_i1_1	0.8872862	0.6462588
DN31904_c0_g1_i1_1	3.052618	2.665066
DN31906_c0_g1_i1_1	0	0.717035
DN31908_c0_g1_i1_1	3.674125	3.502331
DN31911_c0_g1_i1_1	0.1552387	0
DN31912_c0_g1_i1_2	6.697541	2.091549
DN31913_c0_g1_i1_1	0.1325584	0.1486177
DN31914_c0_g1_i1_2	1.71602	0.2065671
DN31917_c0_g1_i1_1	1.59618	3.573065
DN31919_c0_g1_i1_2	2.987083	1.418645
DN3191_c0_g1_i1_1	1.089337	3.334708
DN31920_c0_g1_i1_2	3.012681	4.810979

DN31922_c0_g1_i1_2	0.7385997	1.408079
DN31926_c0_g1_i1_1	1.686003	7.10779
DN31929_c0_g1_i1_1	2.107977	1.573233
DN31931_c0_g1_i1_1	0.6383797	1.290026
DN31932_c0_g1_i1_1	0.810573	2.740851
DN31935_c0_g1_i1_1	0.8650591	1.574405
DN31935_c0_g1_i1_2	0.8383057	0
DN31937_c0_g1_i1_2	5.537651	3.059818
DN31940_c0_g1_i1_1	1.208767	0
DN31942_c0_g1_i1_1	0.2188027	0.8136727
DN31943_c0_g1_i1_1	3.196402	2.589883
DN31946_c0_g1_i1_2	39.18422	145.9598
DN31947_c0_g1_i1_1	0.6213737	0
DN31948_c0_g1_i1_1	3.202755	2.323374
DN31953_c0_g1_i1_2	3.762197	2.527462
DN31954_c0_g1_i1_1	0.2887356	1.503051
DN31956_c0_g1_i1_1	1.366254	1.971986
DN31958_c0_g1_i1_1	0	0
DN31959_c0_g1_i1_2	3.36754	5.871243
DN31960_c0_g1_i1_2	3.133922	0.6918566
DN31963_c0_g1_i1_1	1.90608	0.5577926
DN31964_c0_g1_i1_1	0.3444247	1.957736
DN31965_c0_g1_i1_1	0	4.09316
DN3196_c0_g1_i1_2	1.558609	2.261994

DN31970_c0_g1_i1_2	2.477762	1.665985
DN31971_c0_g1_i1_2	2.911467	2.546782
DN31981_c0_g1_i1_2	2.008937	1.96678
DN31995_c0_g1_i1_2	1.220169	1.893485
DN31999_c0_g1_i1_2	1.977634	1.535268
DN319_c0_g1_i1_1	0.9220054	1.32637
DN32010_c0_g1_i1_2	4.811827	0.4420201
DN32025_c0_g1_i1_2	0.9591651	0.5860714
DN32045_c0_g1_i1_2	27.64071	17.72548
DN32050_c0_g1_i1_2	3.237641	0.9810517
DN32058_c0_g1_i1_2	10.34223	36.79496
DN32068_c0_g1_i1_2	6.004229	5.10496
DN32075_c0_g1_i1_2	3.839131	1.43127
DN32081_c0_g1_i1_2	2.003372	0.9871125
DN32083_c0_g1_i1_2	6.430477	5.658616
DN32087_c0_g1_i1_2	3.047968	2.591464
DN32089_c0_g1_i1_2	0.9601709	3.140845
DN32097_c0_g1_i1_2	1.939871	0.8743674
DN32099_c0_g1_i1_2	6.342787	1.912848
DN32101_c0_g1_i1_2	1.950393	0.3440012
DN32127_c0_g1_i1_2	21.28819	7.391304
DN32140_c0_g1_i1_2	5.791132	5.862993
DN32146_c0_g1_i1_2	0.6866655	0
DN32155_c0_g1_i1_2	7.834964	2.238241

DN32174_c0_g1_i1_2	4.864453	7.500423
DN32175_c0_g1_i1_2	0.4863384	0
DN32176_c0_g1_i1_2	4.997949	2.779924
DN32182_c0_g1_i1_2	2.18574	0
DN32184_c0_g1_i1_2	2.899759	3.064741
DN32185_c0_g1_i1_2	0.5882729	0.1083678
DN32189_c0_g1_i1_2	11.62181	10.04038
DN32192_c0_g1_i1_2	16.24405	11.58282
DN32198_c0_g1_i1_2	1.019346	0.5912446
DN32202_c0_g1_i1_2	2.087504	0.8122127
DN32203_c0_g1_i1_2	0.5970233	0.1747853
DN32208_c0_g1_i1_2	1.440164	1.018008
DN32216_c0_g1_i1_2	1.208349	1.225226
DN3221_c0_g1_i1_1	0	0.3801168
DN32226_c0_g1_i1_2	5.21623	4.072816
DN32239_c0_g1_i1_2	0.07639196	0.2830445
DN32245_c0_g1_i1_2	0.887642	0.2014978
DN32246_c0_g1_i1_2	5.792642	3.61283
DN32256_c0_g1_i1_2	1.267549	0.4551904
DN32259_c0_g1_i1_2	2.868149	1.445012
DN32269_c0_g1_i1_2	738.6495	828.5178
DN32270_c0_g1_i1_2	0	5.386854
DN32275_c0_g1_i1_2	6.5158	3.890256
DN3227_c0_g1_i1_1	0.626284	0.837656

DN32281_c0_g1_i1_2	14.06028	48.85407
DN32294_c0_g1_i1_2	8.608507	5.476514
DN32298_c0_g1_i1_2	9.243855	16.3151
DN322_c0_g1_i1_1	0.9900251	0.6140036
DN32306_c0_g1_i1_2	1.306872	0.3454532
DN32316_c0_g1_i1_2	4.708887	1.429231
DN32317_c0_g1_i1_2	1.781508	1.131875
DN32328_c0_g1_i1_2	6.309872	1.458651
DN3233_c0_g1_i1_1	29.71877	49.09476
DN3233_c0_g1_i1_2	70.30614	64.07261
DN32351_c0_g1_i1_2	1.204744	0.7332239
DN32353_c0_g1_i1_2	0.8639958	0
DN32363_c0_g1_i1_2	0.9183144	0.4086842
DN32364_c0_g1_i1_2	44.44204	10.80528
DN32366_c0_g1_i1_2	0	0.1344505
DN3237_c0_g1_i1_1	0.4841814	0.53245
DN32386_c0_g1_i1_2	1.109126	0.3684266
DN323_c0_g1_i1_1	5.307336	8.276321
DN323_c0_g1_i1_2	4.859215	3.536393
DN32402_c0_g1_i1_2	1.293948	0.8527847
DN32405_c0_g1_i1_2	2.06674	1.991186
DN3240_c0_g1_i1_2	2.823679	1.217834
DN32413_c0_g1_i1_2	1.55535	0
DN32417_c0_g1_i1_2	3.176227	0.9188384

DN32430_c0_g1_i1_2	66.72996	25.45197
DN32432_c0_g1_i1_2	338.2947	0.230235
DN32435_c0_g1_i1_2	1.076712	0.3734324
DN32436_c0_g1_i1_2	0.569355	2.769374
DN32445_c0_g1_i1_2	0.398472	0.8138334
DN3244_c0_g1_i1_2	13.21435	2.276416
DN32451_c0_g1_i1_2	0.9914402	0.834413
DN32452_c0_g1_i1_2	0.2582287	3.44E-30
DN32457_c0_g1_i1_2	1.203995	0.9488144
DN32458_c0_g1_i1_2	2.975306	1.969145
DN32461_c0_g1_i1_2	0.9704282	0
DN32466_c0_g1_i1_2	30.56259	8.782397
DN32467_c0_g1_i1_2	3.505118	1.449044
DN32469_c0_g1_i1_2	2.057063	0.9801656
DN32479_c0_g1_i1_2	1.283633	0
DN32484_c0_g1_i1_2	3.057886	0.7546725
DN3248_c0_g1_i1_2	66.43199	67.70243
DN32490_c0_g1_i1_2	0.8522464	0.5972803
DN32495_c0_g1_i1_2	2.957756	7.31022
DN32500_c0_g1_i1_2	1.428949	2.00452
DN32504_c0_g1_i1_2	3.292556	3.25003
DN32508_c0_g1_i1_2	2.98397	1.038512
DN32513_c0_g1_i1_2	1.295002	3.19129
DN32519_c0_g1_i1_2	0.3993877	1.326454

DN32520_c0_g1_i1_2	0.8457893	0
DN32522_c0_g1_i1_2	12.89031	2.129391
DN32530_c0_g1_i1_2	4.401768	2.220224
DN32531_c0_g1_i1_2	0.7578915	0
DN32541_c0_g1_i1_2	0.1877083	0.265794
DN32547_c0_g1_i1_2	1.441724	0.3638984
DN32548_c0_g1_i1_2	1.223252	0.9919453
DN3255_c0_g1_i1_1	0	2.944863
DN32571_c0_g1_i1_2	4.363611	0.4565714
DN32579_c0_g1_i1_2	74.3832	0.2114981
DN32583_c0_g1_i1_2	2.207917	0.1509543
DN32587_c0_g1_i1_2	2.48186	1.574165
DN32594_c0_g1_i1_2	0.6294882	0.4164149
DN32598_c0_g1_i1_2	1.300347	0.2645609
DN3259_c0_g1_i1_1	0.6122283	1.163715
DN32600_c0_g1_i1_2	4.299702	2.602723
DN32604_c0_g1_i1_2	1.585271	0.5232185
DN32613_c0_g1_i1_2	0.5056562	0.4279288
DN32618_c0_g1_i1_2	1.557437	0
DN32634_c0_g1_i1_2	2.217833	2.963323
DN32635_c0_g1_i1_2	1.559789	0.1108842
DN32637_c0_g1_i1_2	2.073206	2.168852
DN32640_c0_g1_i1_2	3.99952	7.629651
DN32646_c0_g1_i1_2	3.360414	0

DN3264_c0_g1_i1_1	1.596145	3.398522
DN32661_c0_g1_i1_2	5.17899	5.804358
DN32665_c0_g1_i1_2	2.582545	0.9849857
DN32666_c0_g1_i1_2	1.522589	0.198318
DN32677_c0_g1_i1_2	0.395484	0.9800601
DN32685_c0_g1_i1_2	0.02480392	1.893359
DN32689_c0_g1_i1_2	2.74671	0.8820689
DN32695_c0_g1_i1_2	0	0.229447
DN32706_c0_g1_i1_2	2.625845	0
DN32715_c0_g1_i1_2	5.860447	1.923286
DN32725_c0_g1_i1_2	0.941545	0.1355975
DN32726_c0_g1_i1_2	8.6166	4.656783
DN32728_c0_g1_i1_2	0.9608438	0.1435567
DN32729_c0_g1_i1_2	0.2622136	0.7079777
DN32732_c0_g1_i1_2	6.138967	2.725367
DN32741_c0_g1_i1_2	1.929696	3.290757
DN32745_c0_g1_i1_2	3.176955	1.449939
DN32753_c0_g1_i1_2	1.532798	0.8307417
DN32756_c0_g1_i1_2	3.957431	2.011657
DN32757_c0_g1_i1_2	1.396306	0.4845698
DN3275_c0_g1_i1_1	0.3990349	14.90762
DN32760_c0_g1_i1_2	1.963707	0
DN32761_c0_g1_i1_2	2.345873	1.327241
DN32766_c0_g1_i1_2	1.352624	0.815363

DN32777_c0_g1_i1_2	0	2.277978
DN32781_c0_g1_i1_2	6.865571	1.544037
DN32784_c0_g1_i1_2	21.99521	36.51996
DN32792_c0_g1_i1_2	0	0
DN32804_c0_g1_i1_2	1.533125	1.121028
DN3280_c0_g1_i1_2	2.826369	3.630123
DN32816_c0_g1_i1_2	0.9303046	0.6158045
DN32825_c0_g1_i1_2	0.5464643	0.9975034
DN32826_c0_g1_i1_2	1.228376	0
DN32836_c0_g1_i1_2	0.8707051	1.897559
DN32837_c0_g1_i1_2	7.567407	2.647658
DN3283_c0_g1_i1_2	5.254899	0.5878837
DN32848_c0_g1_i1_2	1.597984	0.3413362
DN32856_c0_g1_i1_2	2.647092	1.588085
DN32857_c0_g1_i1_2	50.86388	61.23999
DN32859_c0_g1_i1_2	1.033493	1.989933
DN32865_c0_g1_i1_2	0.8980997	0.8872293
DN32875_c0_g1_i1_2	7.229339	1.27213
DN32877_c0_g1_i1_2	4.653868	3.375303
DN32878_c0_g1_i1_2	3.364551	2.435149
DN32884_c0_g1_i1_2	0.8088966	0.2619085
DN32892_c0_g1_i1_2	10.92505	17.01388
DN32898_c0_g1_i1_2	3.110825	2.498303
DN32899_c0_g1_i1_2	0.265145	0.2237243

DN328_c0_g2_i1_2	0.8897137	0.6210195
DN32900_c0_g1_i1_2	1.217422	0.3499958
DN32903_c0_g1_i1_2	5.863925	1.72871
DN32906_c0_g1_i1_2	3.567704	3.286558
DN3290_c0_g1_i1_1	1.114201	0.6916283
DN32918_c0_g1_i1_2	0	0.3762489
DN32927_c0_g1_i1_2	3.381919	1.887467
DN32936_c0_g1_i1_2	0.9020394	1.104782
DN32939_c0_g1_i1_2	2.84106	1.143075
DN32945_c0_g1_i1_2	1.71546	1.493901
DN32948_c0_g1_i1_2	2.457333	0.4227437
DN3294_c0_g1_i1_1	1.894704	1.567187
DN3295_c0_g1_i1_1	1.075449	0.8132424
DN32962_c0_g1_i1_2	1.081307	0.3088989
DN32963_c0_g1_i1_2	3.061487	1.513166
DN3296_c0_g1_i1_1	0.4034821	0.5998749
DN32972_c0_g1_i1_2	2.138588	0.7653503
DN32973_c0_g1_i1_2	0.3539649	0.3750768
DN32983_c0_g1_i1_2	3.048797	0.3676393
DN3298_c0_g1_i1_1	0.2931652	1.19551
DN32991_c0_g1_i1_2	0	0
DN32992_c0_g1_i1_2	2.704286	1.231773
DN32996_c0_g1_i1_2	2.9571	0.9279851
DN33000_c0_g1_i1_2	3.922582	0.8128786

DN33017_c0_g1_i1_2	0.7894642	0.2868841
DN33030_c0_g1_i1_2	0.5409574	0.363683
DN33040_c0_g1_i1_2	3.961229	0.8391102
DN33045_c0_g1_i1_2	2.387201	18.39381
DN33049_c0_g1_i1_2	1.633755	0.4644205
DN33052_c0_g1_i1_2	45.79573	8.957552
DN33053_c0_g1_i1_2	11.0446	6.785415
DN33058_c0_g1_i1_2	2.421811	1.58368
DN33059_c0_g1_i1_2	0.7027194	0
DN33062_c0_g1_i1_2	3.259672	1.167173
DN33067_c0_g1_i1_2	1.091163	0.2547097
DN33068_c0_g1_i1_2	1.97653	0.7457648
DN3306_c0_g1_i1_2	1.377784	3.283763
DN33073_c0_g1_i1_2	2.336802	1.876946
DN33080_c0_g1_i1_2	0	0.3577013
DN33083_c0_g1_i1_2	0	0
DN33086_c0_g1_i1_2	1.360965	0.712187
DN33101_c0_g1_i1_2	1.906699	0.3465068
DN33107_c0_g1_i1_2	2.365511	0.2351096
DN33109_c0_g1_i1_2	8.988713	2.176429
DN33110_c0_g1_i1_2	3.154909	1.829518
DN33113_c0_g1_i1_2	4.103244	2.119333
DN33115_c0_g1_i1_2	1.115698	0
DN33116_c0_g1_i1_2	1.376675	5.102622

DN33125_c0_g1_i1_2	1.451812	0
DN33129_c0_g1_i1_2	3.572998	0.3090971
DN33131_c0_g1_i1_2	157.731	130.8994
DN33132_c0_g1_i1_2	0.6485519	1.530422
DN33133_c0_g1_i1_2	1.895494	2.202859
DN33137_c0_g1_i1_2	1.77318	0
DN33138_c0_g1_i1_2	2.639731	1.182254
DN3313_c0_g1_i1_2	0.7164569	0.2352171
DN33141_c0_g1_i1_2	0.9692748	0.1859453
DN33147_c0_g1_i1_2	0	0
DN33150_c0_g1_i1_2	4.785076	1.057227
DN3315_c0_g1_i1_1	1.109991	0
DN33160_c0_g1_i1_2	2.187068	0.7403961
DN33162_c0_g1_i1_2	1.750391	1.373959
DN33166_c0_g1_i1_2	1.954841	1.818279
DN33167_c0_g1_i1_2	1.54862	0.5157761
DN33168_c0_g1_i1_2	2.854513	1.918904
DN33171_c0_g1_i1_2	8.94684	5.611464
DN33177_c0_g1_i1_2	0.5755588	0
DN33190_c0_g1_i1_2	0.2995366	0.2254253
DN33195_c0_g1_i1_2	0	0
DN33199_c0_g1_i1_2	101.1709	35.66134
DN3319_c0_g1_i1_2	1.670313	2.232168
DN33205_c0_g1_i1_2	1.540893	0

DN33206_c0_g1_i1_2	0	0
DN33207_c0_g1_i1_2	1.787069	0
DN33208_c0_g1_i1_2	1.860382	6.34E-31
DN33217_c0_g1_i1_2	0	0
DN3321_c0_g1_i1_1	0.5540482	7.860465
DN33223_c0_g1_i1_2	3.725111	0.452836
DN33225_c0_g1_i1_2	1.222311	0
DN3322_c0_g1_i1_1	3.948133	4.546557
DN33233_c0_g1_i1_2	1.012254	0.7186998
DN33234_c0_g1_i1_2	1.195902	0.442085
DN33236_c0_g1_i1_2	1.033845	0
DN33237_c0_g1_i1_2	5.276534	6.252436
DN33239_c0_g1_i1_2	0.5940612	0.3275167
DN33250_c0_g1_i1_2	16.84209	15.55991
DN33255_c0_g1_i1_2	71.66796	130.7391
DN33260_c0_g1_i1_2	6.214625	8.3379
DN33279_c0_g1_i1_2	9.889591	2.813398
DN33286_c0_g1_i1_2	4.781882	2.403497
DN33289_c0_g1_i1_2	3.21773	6.026308
DN3328_c0_g1_i1_1	0.3177883	0.2874657
DN33296_c0_g1_i1_2	10.45317	8.187255
DN33313_c0_g1_i1_2	1.265334	0.8100274
DN33318_c0_g1_i1_2	1.021842	0.8516045
DN33353_c0_g1_i1_2	1.44159	0.5348333

DN33357_c0_g1_i1_2	4.69371	4.03204
DN33363_c0_g1_i1_2	1.451867	0.7540612
DN33365_c0_g1_i1_2	1.314735	0.2865947
DN33373_c0_g1_i1_2	0.5778023	1.526412
DN33378_c0_g1_i1_2	3.392923	1.568425
DN33389_c0_g1_i1_2	1.753702	2.004682
DN33392_c0_g1_i1_2	1.775947	0.8912316
DN33395_c0_g1_i1_2	22.61261	17.60038
DN33405_c0_g1_i1_2	1.34544	0.3759337
DN3340_c0_g1_i1_1	1.498987	2.48436
DN3340_c0_g1_i1_2	2.088961	2.07397
DN33415_c0_g1_i1_2	2.070792	5.453981
DN33425_c0_g1_i1_2	2.258528	0.6915162
DN33427_c0_g1_i1_2	122.5508	63.05162
DN33428_c0_g1_i1_2	1.367024	3.170286
DN3342_c0_g1_i1_1	36.14048	34.42161
DN33430_c0_g1_i1_2	163.8535	81.82549
DN33436_c0_g1_i1_2	10.67945	22.74423
DN33442_c0_g1_i1_2	1.839151	0.09240058
DN33447_c0_g1_i1_2	1.306059	0.9581144
DN33448_c0_g1_i1_2	1.196243	0.3454183
DN33469_c0_g1_i1_2	8.434741	9.275221
DN33472_c0_g1_i1_2	4.033597	1.988901
DN33474_c0_g1_i1_2	2.781316	2.181603

DN33475_c0_g1_i1_2	13.67577	11.85347
DN33478_c0_g1_i1_2	3.686144	0.471784
DN3347_c0_g1_i1_1	1.302093	0
DN33482_c0_g1_i1_2	0.1890841	0.4189561
DN33489_c0_g1_i1_2	9.020155	8.379551
DN3348_c0_g1_i2_1	5.4908	7.154452
DN33493_c0_g1_i1_2	2.310858	1.304947
DN33496_c0_g1_i1_2	1.394392	0.8628837
DN33499_c0_g1_i1_2	30.23388	19.43631
DN334_c0_g2_i1_2	1.377845	0
DN33500_c0_g1_i1_2	2.178787	1.545325
DN33505_c0_g1_i1_2	0.4017506	0
DN3351_c0_g1_i1_2	4.184796	0.9214201
DN33520_c0_g1_i1_2	14.24095	11.95659
DN33543_c0_g1_i1_2	1.187221	0
DN33548_c0_g1_i1_2	0.2924968	0.1560739
DN3354_c0_g1_i1_1	1.224132	1.033135
DN33569_c0_g1_i1_2	2.461254	2.480336
DN3356_c0_g1_i1_1	0.1796647	2.280517
DN33577_c0_g1_i1_2	8.843141	4.69897
DN33578_c0_g1_i1_2	8.518292	2.345426
DN33579_c0_g1_i1_2	1.808654	0
DN3357_c0_g1_i1_1	0.2459735	2.944678
DN33581_c0_g1_i1_2	51.44463	25.60535

DN33595_c0_g1_i1_2	61.3246	10.88625
DN33599_c0_g1_i1_2	6.708917	4.456583
DN335_c0_g1_i2_1	13.15885	31.40322
DN33608_c0_g1_i1_2	0.3847892	0.3067163
DN33609_c0_g1_i1_2	1.484081	0.5632737
DN33617_c0_g1_i1_2	0	0
DN33626_c0_g1_i1_2	2.889812	0.4991078
DN33628_c0_g1_i1_2	4.504051	0.352481
DN33637_c0_g1_i1_2	3.137101	3.251068
DN33638_c0_g1_i1_2	1.81162	0.4881217
DN3363_c0_g1_i1_1	0.8732127	0
DN3363_c0_g1_i1_2	1.887537	0.6706227
DN33654_c0_g1_i1_2	0.365443	0.01768139
DN3365_c0_g1_i1_1	1.59608	1.365331
DN33667_c0_g1_i1_2	2.796201	3.070112
DN33676_c0_g1_i1_2	1.183366	0.8838001
DN33677_c0_g1_i1_2	2.81013	0.8425331
DN33684_c0_g1_i1_2	6.62046	8.350858
DN33692_c0_g1_i1_2	1.354647	1.012259
DN336_c0_g1_i1_1	0.5112978	2.394402
DN33701_c0_g1_i1_2	1.18418	1.972904
DN33705_c0_g1_i1_2	4.93049	1.620121
DN3371_c0_g1_i1_1	0.4147313	0.3117327
DN33724_c0_g1_i1_2	4.141104	3.313595

DN33737_c0_g1_i1_2	11.37823	12.40537
DN33739_c0_g1_i1_2	2.478153	0.4515358
DN3373_c0_g1_i1_1	0.3242971	0.7895052
DN33748_c0_g1_i1_2	2.603954	0.9844029
DN33752_c0_g1_i1_2	1.193074	0
DN33758_c0_g1_i1_2	2.898169	1.268222
DN3375_c0_g1_i1_1	0.8137617	1.761194
DN3375_c0_g2_i1_1	0.8319806	2.540943
DN33761_c0_g1_i1_2	1.182099	0.4604943
DN33763_c0_g1_i1_2	1.591862	0.8789039
DN33779_c0_g1_i1_2	2.885859	1.202604
DN33783_c0_g1_i1_2	8.830434	16.72893
DN337_c0_g1_i1_2	1.650884	1.738114
DN33801_c0_g1_i1_2	3.494666	5.490166
DN33815_c0_g1_i1_2	1.761176	0.7958931
DN33826_c0_g1_i1_2	0	0
DN33830_c0_g1_i1_2	2.594147	0.8162875
DN33832_c0_g1_i1_2	2.934201	3.330528
DN33833_c0_g1_i1_2	0	0
DN33847_c0_g1_i1_2	3.755318	2.966097
DN33849_c0_g1_i1_2	3.419934	2.90452
DN33861_c0_g1_i1_2	1.571114	2.245172
DN33871_c0_g1_i1_2	4.545239	2.403615
DN33888_c0_g1_i1_2	1.226827	1.627343

DN3388_c0_g1_i1_2	10.05962	7.726684
DN33893_c0_g1_i1_2	1.377161	3.754684
DN33894_c0_g1_i1_2	2.109822	0.4929787
DN3389_c0_g1_i1_1	1.193127	4.700428
DN338_c0_g1_i1_1	0.7508454	1.473038
DN33901_c0_g1_i1_2	2.202619	1.059358
DN33910_c0_g1_i1_2	1.808362	0.8621503
DN33911_c0_g1_i1_2	15.85903	6.759211
DN33915_c0_g1_i1_2	5.031012	2.377073
DN33921_c0_g1_i1_2	3.674733	11.59948
DN33926_c0_g1_i1_2	0	0
DN33932_c0_g1_i1_2	3.432329	1.714786
DN33935_c0_g1_i1_2	6.987453	9.882518
DN33937_c0_g1_i1_2	5.639424	15.88786
DN3393_c0_g1_i1_1	0	3.984924
DN33951_c0_g1_i1_2	0	0
DN33955_c0_g1_i1_2	5.048491	0.838346
DN3395_c0_g1_i1_1	0.6742079	0.7591128
DN33966_c0_g1_i1_2	2.807852	0.6575257
DN33984_c0_g1_i1_2	299.7795	0.2877799
DN33994_c0_g1_i1_2	2.604223	0.4166222
DN33995_c0_g1_i1_2	5.147605	2.950487
DN3399_c0_g1_i1_1	1.774267	1.716436
DN34008_c0_g1_i1_2	1.691227	1.312049

DN34009_c0_g1_i1_2	2.612541	1.18857
DN3400_c0_g1_i1_1	0.2553011	0.3517031
DN3400_c0_g1_i1_2	6.513292	5.627769
DN34019_c0_g1_i1_2	0.3444885	0.4302552
DN3401_c0_g1_i1_2	86.38454	0.1306792
DN34023_c0_g1_i1_2	1.526231	3.789895
DN34029_c0_g1_i1_2	1.885541	0.66275
DN34031_c0_g1_i1_2	7.557421	4.961148
DN34040_c0_g1_i1_2	3.322943	2.12466
DN34048_c0_g1_i1_2	3.599139	0.4282948
DN34052_c0_g1_i1_2	0.7080739	1.606178
DN34057_c0_g1_i1_2	1.281285	0.6549927
DN3405_c0_g1_i1_1	2.260169	8.465766
DN34068_c0_g1_i1_2	10.1084	10.00069
DN34069_c0_g1_i1_2	5.939434	3.625535
DN34072_c0_g1_i1_2	13.13409	15.98348
DN34086_c0_g1_i1_2	0.6840673	0.2813735
DN34098_c0_g1_i1_2	6.049594	2.424814
DN34100_c0_g1_i1_2	1.041399	0.3566247
DN34110_c0_g1_i1_2	4.05703	1.019536
DN34115_c0_g1_i1_2	6.805429	3.427597
DN34126_c0_g1_i1_2	5.923447	1.614775
DN34153_c0_g1_i1_2	1.525158	0.9319273
DN34159_c0_g1_i1_2	1.324423	1.545575

DN34162_c0_g1_i1_2	2.117389	0.6637512
DN3417_c0_g1_i1_2	6.267947	2.591856
DN34182_c0_g1_i1_2	1.722629	0.2979577
DN34191_c0_g1_i1_2	4.933865	2.063425
DN34192_c0_g1_i1_2	19.0397	13.20823
DN34194_c0_g1_i1_2	78.75464	98.01701
DN34195_c0_g1_i1_2	11.3293	5.713216
DN34197_c0_g1_i1_2	3.173915	0.5234502
DN341_c0_g1_i1_1	1.397229	3.267008
DN341_c0_g1_i1_2	3.25195	0.7561735
DN341_c0_g2_i1_1	0.5911902	0.61864
DN3420_c0_g1_i1_1	1.309106	2.694492
DN34219_c0_g1_i1_2	27.31528	24.37442
DN34224_c0_g1_i1_2	1.718591	1.859933
DN34236_c0_g1_i1_2	2.096495	0.8687121
DN34243_c0_g1_i1_2	1.210344	0.1537193
DN34248_c0_g1_i1_2	5.353422	3.32972
DN34257_c0_g1_i1_2	0.1476648	0.2713994
DN34262_c0_g1_i1_2	2.866829	2.155918
DN3426_c0_g1_i1_1	0.6753167	4.798528
DN3426_c0_g2_i1_1	0.4448888	4.763523
DN34277_c0_g1_i1_2	1.444995	0.8949346
DN34280_c0_g1_i1_2	0.7216746	0
DN34286_c0_g1_i1_2	6.228173	7.793486

DN34288_c0_g1_i1_2	1.421051	0.08766115
DN34295_c0_g1_i1_2	10.38286	7.716173
DN342_c0_g1_i1_2	23.87329	18.22669
DN34315_c0_g1_i1_2	2.053181	0.5597628
DN34327_c0_g1_i1_2	43.67225	0
DN3432_c0_g1_i1_2	3.388729	3.624531
DN34333_c0_g1_i1_2	0.7096234	0
DN34349_c0_g1_i1_2	2.201478	0.7086312
DN34351_c0_g1_i1_2	0.7024492	1.152416
DN3435_c0_g1_i1_1	0.3086897	1.352997
DN34368_c0_g1_i1_2	1.458267	1.434948
DN3436_c0_g1_i1_1	0.4317305	0.8929579
DN34375_c0_g1_i1_2	0.7149005	0
DN34379_c0_g1_i1_2	4.124726	1.470438
DN34386_c0_g1_i1_2	1.622461	1.353865
DN34391_c0_g1_i1_2	2.904269	19.41888
DN34394_c0_g1_i1_2	0.9872793	0.5820246
DN34395_c0_g1_i1_2	0.8906198	0.9376091
DN34396_c0_g1_i1_2	15.98523	28.08156
DN34397_c0_g1_i1_2	2.773727	1.87141
DN34400_c0_g1_i1_2	1.462156	1.710039
DN34410_c0_g1_i1_2	7.860063	4.056549
DN34414_c0_g1_i1_2	0	0
DN34422_c0_g1_i1_2	1.801089	1.727359

DN34426_c0_g1_i1_2	3.696634	3.318869
DN34438_c0_g1_i1_2	7.250833	0.6452931
DN3443_c0_g1_i1_2	0.950236	2.213774
DN34442_c0_g1_i1_2	3.721482	0.76952
DN34444_c0_g1_i1_2	5.765514	4.827589
DN34447_c0_g1_i1_2	4.790219	2.523859
DN34469_c0_g1_i1_2	0.7019323	4.02E-19
DN34470_c0_g1_i1_2	2.732855	4.427682
DN34471_c0_g1_i1_2	1.834748	1.589669
DN34473_c0_g1_i1_2	0.6439502	0.4668544
DN34479_c0_g1_i1_2	0	0
DN3447_c0_g1_i1_2	1.761217	0.5469852
DN34485_c0_g1_i1_2	0	0
DN34488_c0_g1_i1_2	25.49171	22.78514
DN3449_c0_g1_i1_2	2.521372	0.811323
DN34504_c0_g1_i1_2	1.094924	0.04779684
DN34505_c0_g1_i1_2	7.268044	3.512395
DN34508_c0_g1_i1_2	8.241542	5.020474
DN3450_c0_g1_i1_2	1.711656	2.297355
DN34518_c0_g1_i1_2	0.9805189	0.3138722
DN3451_c0_g1_i1_1	0	2.227223
DN34525_c0_g1_i1_2	16.15575	6.297645
DN3452_c0_g1_i1_2	4.287563	1.382265
DN34535_c0_g1_i1_2	2.49498	2.319245

DN34537_c0_g1_i1_2	1.37E-29	3.29E-28
DN34539_c0_g1_i1_2	4.895582	6.355418
DN34546_c0_g1_i1_2	1.387696	0.2388756
DN34548_c0_g1_i1_2	1.961573	1.965132
DN3454_c0_g1_i1_1	0.7127993	1.154035
DN34550_c0_g1_i1_2	7.184249	8.074047
DN34551_c0_g1_i1_2	1.783014	0.8430043
DN34561_c0_g1_i1_2	76.64566	151.0439
DN34566_c0_g1_i1_2	3.116517	1.059738
DN34567_c0_g1_i1_2	2.737842	2.505813
DN34568_c0_g1_i1_2	1.519051	2.166794
DN34572_c0_g1_i1_2	1.748361	0
DN34578_c0_g1_i1_2	1.212149	0.6682618
DN3457_c0_g1_i1_1	0.231545	1.631408
DN34593_c0_g1_i1_2	0.3023748	0.3963884
DN34595_c0_g1_i1_2	0.764904	0.270138
DN34598_c0_g1_i1_2	0.4095574	2.010061
DN34606_c0_g1_i1_2	29.4551	20.859
DN34616_c0_g1_i1_2	3.795426	6.502269
DN34619_c0_g1_i1_2	2.297878	0
DN34624_c0_g1_i1_2	1.061229	0.2995537
DN34627_c0_g1_i1_2	4.204784	1.122133
DN34639_c0_g1_i1_2	76.89882	48.78642
DN34648_c0_g1_i1_2	2.022255	0.8385389

DN34653_c0_g1_i1_2	1.952955	1.253633
DN34654_c0_g1_i1_2	1.102453	0.2847454
DN34659_c0_g1_i1_2	4.870976	1.177345
DN3465_c0_g1_i1_1	0.7596084	2.229138
DN34663_c0_g1_i1_2	2.536431	1.762523
DN34669_c0_g1_i1_2	0.5829357	0.3194997
DN3467_c0_g1_i1_1	0.7395696	0.4075401
DN34694_c0_g1_i1_2	2.191507	0.8144006
DN34696_c0_g1_i1_2	3.941595	2.019154
DN34698_c0_g1_i1_2	41.26458	15.52629
DN34703_c0_g1_i1_2	32.96461	10.27569
DN34705_c0_g1_i1_2	53.60501	24.63967
DN34716_c0_g1_i1_2	0.8831398	0
DN34730_c0_g1_i1_2	249.0732	0
DN34739_c0_g1_i1_2	0	0
DN3474_c0_g1_i1_2	3.235925	8.301396
DN34751_c0_g1_i1_2	3.901461	0.9759418
DN34758_c0_g1_i1_2	1.076695	0.4244153
DN34766_c0_g1_i1_2	4.229896	2.199297
DN34769_c0_g1_i1_2	1.041369	0
DN34770_c0_g1_i1_2	0.7803825	0.1735478
DN34774_c0_g1_i1_2	5.961744	2.488408
DN34780_c0_g1_i1_2	4.213039	1.68703
DN34789_c0_g1_i1_2	0.3534299	0.5535296

DN34791_c0_g1_i1_2	1.194728	1.304115
DN34793_c0_g1_i1_2	0.1974935	0.6582824
DN34796_c0_g1_i1_2	0	0
DN34804_c0_g1_i1_2	1.744612	0.09407847
DN34807_c0_g1_i1_2	1.552238	0.3655134
DN34810_c0_g1_i1_2	12.16798	6.804691
DN34817_c0_g1_i1_2	2.820544	0.3020016
DN34822_c0_g1_i1_2	3.259698	3.304264
DN34823_c0_g1_i1_2	0.5389565	0.3812755
DN34827_c0_g1_i1_2	5.008076	6.578268
DN34833_c0_g1_i1_2	1.432722	0.814906
DN34844_c0_g1_i1_2	2.164297	0.7417487
DN34846_c0_g1_i1_2	1.845725	0.5713277
DN34848_c0_g1_i1_2	0.3962313	0
DN34850_c0_g1_i1_2	0.6098327	0.1929483
DN34854_c0_g1_i1_2	0.9455863	3.238735
DN34856_c0_g1_i1_2	5.700796	0.3557388
DN34857_c0_g1_i1_2	0.1784367	3.84E-31
DN34859_c0_g1_i1_2	1.762782	0
DN34866_c0_g1_i1_2	2.216601	0.935543
DN34869_c0_g1_i1_2	0.3032957	0.0613698
DN34870_c0_g1_i1_2	1.462908	1.012942
DN34872_c0_g1_i1_2	2.486248	5.134409
DN34881_c0_g1_i1_2	2.125415	0.5534714

DN34885_c0_g1_i1_2	3.758684	0.8995697
DN34891_c0_g1_i1_2	0.4990577	0
DN34894_c0_g1_i1_2	2.914707	0
DN34903_c0_g1_i1_2	74.62123	13.37665
DN34906_c0_g1_i1_2	0.5667977	0.03560491
DN34914_c0_g1_i1_2	1.123192	4.358417
DN34937_c0_g1_i1_2	1.618215	0.2038435
DN34944_c0_g1_i1_2	2.912433	6.795201
DN34945_c0_g1_i1_2	0	0.8565366
DN34946_c0_g1_i1_2	0.3865654	2.293947
DN34950_c0_g1_i1_2	89.64727	74.96125
DN34951_c0_g1_i1_2	5.774209	1.581642
DN34954_c0_g1_i1_2	0.445461	0
DN34962_c0_g1_i1_2	0	0
DN34964_c0_g1_i1_2	1.187187	0
DN34968_c0_g1_i1_2	1.232991	1.497461
DN34990_c0_g1_i1_2	3.93679	9.23E-22
DN34994_c0_g1_i1_2	0.4384445	0
DN35002_c0_g1_i1_2	2.898196	5.59E-27
DN3500_c0_g1_i1_1	1.089604	1.820779
DN35024_c0_g1_i1_2	4.00992	0
DN3503_c0_g1_i1_2	0	0
DN35041_c0_g1_i1_2	20.82015	21.22528
DN35042_c0_g1_i1_2	0	0

DN35043_c0_g1_i1_2	1.440364	0
DN35045_c0_g1_i1_2	0.9354546	0
DN35046_c0_g1_i1_2	11.10414	12.05164
DN35048_c0_g1_i1_2	11.79299	5.365216
DN35050_c0_g1_i1_2	2.132356	0.6217706
DN3505_c0_g1_i1_2	1.211684	0.146994
DN35065_c0_g1_i1_2	1.474772	0.3383159
DN35073_c0_g1_i1_2	2.329468	0.8942708
DN3507_c0_g1_i1_1	8.610082	3.770244
DN35081_c0_g1_i1_2	2.777888	3.466758
DN3508_c0_g1_i1_2	3.90267	1.740192
DN35090_c0_g1_i1_2	1.340007	0
DN35091_c0_g1_i1_2	4.587781	1.370722
DN35110_c0_g1_i1_2	1.133773	0
DN35111_c0_g1_i1_2	0	0
DN35121_c0_g1_i1_2	2.732768	1.090088
DN35122_c0_g1_i1_2	342.3539	551.7793
DN35126_c0_g1_i1_2	2.040406	0.473834
DN3512_c0_g1_i1_1	1.521855	1.79552
DN35134_c0_g1_i1_2	18.43942	31.74928
DN35139_c0_g1_i1_2	0.1930488	0.2517728
DN35148_c0_g1_i1_2	0.9674111	2.42563
DN35151_c0_g1_i1_2	6.487365	6.195944
DN35157_c0_g1_i1_2	283.7673	132.9351

DN35165_c0_g1_i1_2	5.551357	3.592136
DN35169_c0_g1_i1_2	2.363657	1.174816
DN35172_c0_g1_i1_2	3.063445	2.339246
DN35174_c0_g1_i1_2	2.005522	0.5503837
DN35177_c0_g1_i1_2	3.857329	1.815371
DN35182_c0_g1_i1_2	1.769006	0.8431536
DN35187_c0_g1_i1_2	0	0
DN35188_c0_g1_i1_2	7.872869	3.195481
DN35189_c0_g1_i1_2	6.194617	2.964561
DN35192_c0_g1_i1_2	0.1015064	3.861987
DN35193_c0_g1_i1_2	2.331145	0.8610741
DN35195_c0_g1_i1_2	0.7374118	0.3272106
DN35197_c0_g1_i1_2	4.204045	0
DN35199_c0_g1_i1_2	2.67013	1.587924
DN3519_c0_g1_i1_2	0.6261937	2.104111
DN351_c0_g1_i1_1	0.05309873	2.184411
DN3520_c0_g1_i1_1	0	1.067529
DN35211_c0_g1_i1_2	2.997844	0
DN35213_c0_g1_i1_2	3.797215	0.1926458
DN35215_c0_g1_i1_2	22.57529	7.407436
DN35220_c0_g1_i1_2	34.49502	46.70112
DN35222_c0_g1_i1_2	3.995746	0.771766
DN3522_c0_g1_i1_1	1.054159	3.468458
DN35245_c0_g1_i1_2	1.518738	0.6848628

DN35252_c0_g1_i1_2	0.7788053	0.6125608
DN35254_c0_g1_i1_2	7.968488	2.214556
DN35267_c0_g1_i1_2	0.6129395	1.057234
DN35268_c0_g1_i1_2	2.716868	0.4712506
DN35274_c0_g1_i1_2	2.346437	0.533833
DN35281_c0_g1_i1_2	0.1821244	0
DN35283_c0_g1_i1_2	6.807772	1.973474
DN35285_c0_g1_i1_2	0.9724352	0.1110635
DN35288_c0_g1_i1_2	0	0
DN35306_c0_g1_i1_2	3.860299	1.685993
DN35311_c0_g1_i1_2	7.170923	5.052304
DN35315_c0_g1_i1_2	5.417764	1.81154
DN35318_c0_g1_i1_2	2.200696	1.128377
DN35328_c0_g1_i1_2	0.9040926	0.2097151
DN35333_c0_g1_i1_2	4.430441	1.078288
DN35334_c0_g1_i1_2	2.741951	1.950044
DN35336_c0_g1_i1_2	18.26478	8.735655
DN3533_c0_g1_i1_1	2.677069	1.95146
DN35343_c0_g1_i1_2	2.67567	1.719265
DN35349_c0_g1_i1_2	1.021594	0.5382117
DN35358_c0_g1_i1_2	1.417237	2.080442
DN35360_c0_g1_i1_2	3.382783	2.940733
DN35364_c0_g1_i1_2	2.443934	0.7493271
DN35367_c0_g1_i1_2	35.64506	19.50192

DN35368_c0_g1_i1_2	4.39314	0.2729255
DN35374_c0_g1_i1_2	3.928298	1.210189
DN3537_c0_g1_i1_1	0	3.823783
DN3538_c0_g1_i1_1	0.2249764	1.731549
DN3538_c0_g2_i1_1	0.637394	1.875533
DN35391_c0_g1_i1_2	0.9891573	0.4585405
DN35392_c0_g1_i1_2	46.18489	27.01102
DN35395_c0_g1_i1_2	30.48306	11.49282
DN35401_c0_g1_i1_2	3.615691	3.222187
DN35404_c0_g1_i1_2	90.30795	8.027597
DN35407_c0_g1_i1_2	0.8907108	0.1952149
DN35411_c0_g1_i1_2	0.3716755	0.2385753
DN35416_c0_g1_i1_2	1.96867	0
DN35417_c0_g1_i1_2	10.28775	2.630207
DN35418_c0_g1_i1_2	3.885791	2.668477
DN35423_c0_g1_i1_2	0.9290667	4.915926
DN35430_c0_g1_i1_2	0.6082465	2.914144
DN35442_c0_g1_i1_2	1.081729	0
DN35446_c0_g1_i1_2	0.2567817	0.846294
DN35449_c0_g1_i1_2	1.490962	0.728637
DN3544_c0_g1_i1_1	2.129199	0.9772141
DN35464_c0_g1_i1_2	0.1140974	0.4158978
DN35473_c0_g1_i1_2	3.244295	0.1961927
DN3547_c0_g1_i1_1	0.7173836	2.155304

DN35480_c0_g1_i1_2	1.705646	3.737208
DN35483_c0_g1_i1_2	1.399475	1.554371
DN35489_c0_g1_i1_2	1.571341	0
DN35497_c0_g1_i1_2	1.793004	1.842476
DN3549_c0_g1_i1_2	5.121686	2.640135
DN35500_c0_g1_i1_2	7.166422	2.863072
DN35503_c0_g1_i1_2	75.85254	71.18805
DN35505_c0_g1_i1_2	30.1215	34.3727
DN35515_c0_g1_i1_2	2.010314	0.7726221
DN35516_c0_g1_i1_2	26.38642	17.75095
DN3551_c0_g1_i1_2	0.6254993	0
DN35523_c0_g1_i1_2	2.472527	1.154746
DN35527_c0_g1_i1_2	2.07174	0
DN35529_c0_g1_i1_2	0.7352905	1.469601
DN35533_c0_g1_i1_2	1.953388	2.655335
DN35534_c0_g1_i1_2	0.5194755	0
DN35537_c0_g1_i1_2	0.114772	0.6768626
DN3553_c0_g1_i1_2	0.7719914	1.964942
DN35549_c0_g1_i1_2	1.636341	0.7802247
DN35552_c0_g1_i1_2	1.053828	0.2481335
DN35558_c0_g1_i1_2	0	0
DN35567_c0_g1_i1_2	7.462816	6.760827
DN35569_c0_g1_i1_2	3.166797	4.162212
DN3556_c0_g1_i1_1	1.771171	9.280011

DN3556_c0_g2_i1_1	2.083156	9.081255
DN35581_c0_g1_i1_2	0.6481895	0.8128043
DN35583_c0_g1_i1_2	4.265113	1.985903
DN35590_c0_g1_i1_2	1.742861	0.6126069
DN35592_c0_g1_i1_2	3.339777	4.878184
DN35604_c0_g1_i1_2	4.187241	5.330582
DN35606_c0_g1_i1_2	0.4502453	2.580912
DN35616_c0_g1_i1_2	6.08273	1.309154
DN3566_c0_g1_i1_1	0.9908743	4.117762
DN3567_c0_g1_i1_2	5.236291	0.4235191
DN356_c0_g1_i1_1	6.38279	6.291716
DN356_c0_g1_i1_2	1.37667	0.8280637
DN356_c0_g2_i1_2	3.775844	3.057535
DN3572_c0_g1_i1_1	3.668923	2.44556
DN3572_c0_g2_i1_1	0.9801044	1.927931
DN3573_c0_g1_i1_1	0.6069056	2.381274
DN3578_c0_g1_i1_2	40.28218	56.24525
DN3593_c0_g1_i1_1	1.635692	5.27224
DN3595_c0_g1_i1_1	0.9369111	3.383454
DN3606_c0_g1_i1_1	0.8770688	1.516484
DN360_c0_g1_i1_1	1.812911	5.330603
DN3610_c0_g1_i1_1	3.05602	3.376036
DN3613_c0_g1_i1_1	1.79022	2.597596
DN3613_c0_g1_i1_2	1.394337	3.92E-61

DN3614_c0_g1_i1_1	0.1428801	0.5468352
DN3614_c0_g2_i1_1	0.37054	0.5264563
DN3616_c0_g1_i1_2	8.072298	3.136109
DN3618_c0_g1_i1_1	0.5235187	0.5067155
DN3620_c0_g1_i1_1	2.81571	1.78257
DN3623_c0_g1_i1_1	1.023883	3.629871
DN3625_c0_g1_i1_2	1.411956	0.9285172
DN3628_c0_g1_i1_1	3.332108	9.367653
DN3638_c0_g1_i1_1	0.3897814	1.761552
DN3639_c0_g1_i1_1	1.755595	1.915756
DN3639_c0_g2_i1_1	3.047312	3.942438
DN363_c0_g1_i1_1	0.4910367	0.3092843
DN363_c0_g2_i1_1	0.1387501	0.4394788
DN3641_c0_g1_i1_1	2.193805	30.28676
DN3649_c0_g1_i1_1	0.4262905	1.012484
DN3650_c0_g1_i1_1	1.513422	1.154941
DN3655_c0_g1_i1_1	0.63849	0.6668413
DN3662_c0_g1_i1_1	0.7841798	3.505659
DN3662_c0_g1_i1_2	5.863208	4.027766
DN3665_c0_g1_i1_1	0.2542778	0.7084808
DN3669_c0_g1_i1_1	0.380626	2.904722
DN3670_c0_g1_i1_2	0	0
DN3673_c0_g2_i1_1	0.8493596	2.013583
DN3674_c0_g1_i1_2	1.862058	0

DN3675_c0_g1_i1_1	1.658855	0.6598041
DN3678_c0_g1_i1_2	2.525321	1.86017
DN3679_c0_g1_i1_1	1.141138	1.17692
DN3679_c0_g2_i1_1	0.6791192	0.9500788
DN3680_c0_g1_i1_2	3.470923	3.406185
DN3683_c0_g1_i1_1	0.6963693	1.561919
DN3684_c0_g1_i1_1	0.6724699	0.1252906
DN3688_c0_g2_i1_1	1.720002	3.547295
DN3690_c0_g1_i1_1	0.6097467	0.8478901
DN369_c0_g1_i1_1	3.001633	7.3029
DN3700_c0_g1_i1_1	1.210132	2.587748
DN3702_c0_g1_i1_1	10.23519	8.976864
DN3705_c0_g1_i1_1	0.4618058	3.576975
DN3706_c0_g1_i1_2	10.57172	11.12679
DN3709_c0_g1_i1_1	1.296201	2.809592
DN3718_c0_g1_i1_1	1.483744	2.397886
DN3725_c0_g1_i1_2	3.338637	0.9960238
DN3730_c0_g1_i1_1	2.43E-10	0.7919729
DN373_c0_g1_i1_1	7.483577	11.3827
DN3740_c0_g1_i2_1	1.856546	2.155312
DN3749_c0_g1_i1_1	0.7023166	1.141065
DN3750_c0_g1_i1_2	0	1.616571
DN3752_c0_g1_i1_2	0.4843999	0
DN3762_c0_g1_i1_1	0.5551206	1.006208

DN3766_c0_g1_i1_1	1.602959	2.162334
DN3776_c0_g1_i1_2	1.804999	2.649473
DN3776_c0_g1_i2_1	0.085724	0.5307563
DN3782_c0_g1_i1_1	0	2.14E-111
DN3783_c0_g1_i1_1	2.530206	4.645111
DN3784_c0_g1_i1_1	0.6625031	1.402324
DN3785_c0_g2_i1_1	1.138048	10.85718
DN3787_c0_g1_i1_1	0.3221984	1.851966
DN3794_c0_g1_i1_1	4.134404	1.873672
DN3796_c0_g1_i1_1	1.403434	4.091815
DN3799_c0_g1_i1_2	4.450346	4.349781
DN3800_c0_g1_i1_1	1.391312	1.670941
DN3806_c0_g1_i1_1	0.9492958	0.7960608
DN3816_c0_g1_i1_1	0.1983732	0.270129
DN3820_c0_g1_i1_2	3.60853	1.686796
DN3826_c0_g1_i1_1	0.8726063	3.062711
DN3835_c0_g1_i1_1	0.1144181	1.204647
DN3837_c0_g1_i1_1	4.488038	3.217431
DN3838_c0_g1_i1_1	9.240047	4.221038
DN3839_c0_g1_i1_2	0	0
DN3840_c0_g1_i2_1	3.91446	5.541414
DN3842_c0_g1_i1_2	2.321134	0.2745199
DN3848_c0_g1_i1_1	1.23261	1.471882
DN3854_c0_g1_i2_1	5.37301	10.92954

DN3861_c0_g1_i1_2	0.9399355	0.1532667
DN3864_c0_g1_i1_2	0.5904549	0
DN3869_c0_g1_i1_1	0.3305949	2.466104
DN3871_c0_g1_i1_1	2.053468	1.247741
DN3872_c0_g1_i1_2	2.483167	1.338059
DN3875_c0_g1_i1_1	0.6245363	0.5854003
DN3877_c0_g1_i1_1	2.689282	1.486434
DN3880_c0_g1_i1_1	0	1.434534
DN3881_c0_g1_i1_2	4.051336	3.722918
DN3889_c0_g1_i1_1	1.740765	1.371913
DN3889_c0_g1_i1_2	1.328747	0.1086866
DN3890_c0_g1_i1_1	3.555491	1.767656
DN3892_c0_g1_i1_1	1.58742	9.985481
DN3895_c0_g1_i1_1	0.9470008	1.106576
DN38_c0_g1_i1_1	2.97513	2.870358
DN3900_c0_g1_i1_1	89.81699	20.68448
DN3901_c0_g1_i1_1	0.04170217	4.785035
DN3901_c0_g1_i1_2	0.1275045	0.1076109
DN3904_c0_g1_i1_1	1.997022	2.235631
DN3908_c0_g1_i1_1	0	1.002911
DN3909_c0_g1_i1_1	0.442636	1.371876
DN390_c0_g1_i1_2	2.538207	1.090623
DN3910_c0_g1_i1_1	1.193674	3.685872
DN3911_c0_g1_i1_1	0.8201883	2.315755

DN3917_c0_g1_i1_2	1.722353	1.814582
DN391_c0_g1_i1_1	1.798121	1.816591
DN3921_c0_g1_i1_1	0.2138739	1.275382
DN3924_c0_g1_i1_1	4.305505	7.960771
DN3939_c0_g1_i1_2	2.272498	0
DN3942_c0_g1_i1_1	1.127175	0.7610441
DN3944_c0_g1_i1_1	0.2631065	9.561777
DN3947_c0_g1_i1_1	0.5755549	3.742571
DN3948_c0_g1_i1_1	0.1358954	1.180191
DN3948_c0_g1_i1_2	6.885283	5.474194
DN3949_c0_g1_i1_2	14.80816	20.79575
DN394_c0_g1_i1_2	1.337159	1.054475
DN3959_c0_g1_i1_1	0.743037	1.359871
DN395_c0_g1_i1_2	0.3510618	1.42E-10
DN3965_c0_g1_i1_1	2.370098	4.869628
DN3969_c0_g1_i1_1	0.4179488	1.306112
DN3969_c0_g1_i1_2	7.578751	2.718893
DN3971_c0_g1_i1_1	1.23777	2.758001
DN3983_c0_g1_i1_2	0.6071467	0.3710269
DN3984_c0_g1_i1_2	5.635926	2.044978
DN3986_c0_g1_i1_1	1.556214	13.07105
DN3987_c0_g2_i1_1	7.29967	3.822047
DN3991_c0_g1_i1_1	0.4693368	0.3931393
DN3991_c0_g1_i1_2	0.3399222	0.14454

DN3994_c0_g1_i1_1	6.425178	7.637801
DN3995_c0_g1_i1_2	9.042052	2.199529
DN4001_c0_g1_i1_2	1.00204	0.3582052
DN4005_c0_g1_i1_1	1.034969	1.928779
DN4008_c0_g1_i1_1	0.8853384	2.160716
DN400_c0_g1_i1_2	0	0
DN4015_c0_g1_i1_1	7.943336	52.25557
DN4015_c0_g2_i1_1	1.908513	40.96762
DN401_c0_g1_i1_1	1.451953	2.17165
DN4023_c0_g1_i1_1	1.115092	1.651604
DN4035_c0_g1_i1_1	5.412379	4.082824
DN4040_c0_g1_i1_2	3.436259	1.223733
DN4045_c0_g1_i1_2	4.285756	1.50963
DN4045_c0_g2_i1_1	0.786823	1.29314
DN4049_c0_g1_i1_1	0.8010612	0.9670304
DN404_c0_g1_i1_1	0.8235991	1.548352
DN4053_c0_g1_i1_1	0.6447632	0.5494427
DN4055_c0_g1_i1_1	4.078034	4.243004
DN4057_c0_g1_i1_1	2.350782	1.867881
DN4068_c0_g1_i1_1	4.327165	8.607691
DN4079_c0_g1_i1_2	1.321565	2.068872
DN407_c0_g1_i1_2	2.016226	0.2146975
DN4083_c0_g1_i1_1	0.278906	4.920814
DN4083_c0_g2_i1_1	0.150575	4.646778

DN4091_c0_g1_i1_1	1.052867	1.263387
DN4092_c0_g1_i1_2	0.4120552	5.73E-31
DN4096_c0_g1_i1_1	2.654062	2.874672
DN4102_c0_g1_i1_2	0	0
DN4104_c0_g1_i1_2	0.8772027	0.6159934
DN4107_c0_g1_i1_2	1.451305	1.33722
DN4107_c0_g2_i1_2	2.658382	1.383184
DN410_c0_g1_i1_2	2.325861	1.794992
DN4110_c0_g1_i1_1	1.211586	0
DN4111_c0_g1_i1_1	2.29184	4.416962
DN4120_c0_g1_i1_1	0.868771	0.9401566
DN4124_c0_g1_i1_1	0.3415172	1.269178
DN4127_c0_g1_i1_1	0.1101647	2.266376
DN4127_c0_g1_i1_2	2.702737	0.6843213
DN412_c0_g1_i1_2	0.5916186	0
DN4130_c0_g1_i1_1	8.561203	17.67407
DN4131_c0_g2_i1_1	2.198503	1.448266
DN4138_c0_g1_i1_1	5.287004	7.433805
DN413_c0_g1_i1_1	0.4917885	1.112116
DN4141_c0_g1_i1_2	0	0
DN4145_c0_g1_i1_1	6.931745	6.056655
DN4145_c0_g1_i1_2	0.6321782	0.7314399
DN4146_c0_g1_i1_1	0.5248738	0.1832066
DN4150_c0_g1_i1_1	0.07023758	0.4601947

DN4152_c0_g1_i1_1	0.7469848	1.892139
DN4152_c0_g2_i1_1	1.252187	1.477443
DN4159_c0_g2_i1_2	1.697521	0
DN415_c0_g1_i1_2	5.940136	0.2770806
DN4161_c0_g1_i1_2	0	0
DN4163_c0_g2_i1_1	3.489539	6.19633
DN4169_c0_g1_i1_1	1.528096	3.129567
DN4169_c0_g2_i1_1	1.811995	1.982815
DN4170_c0_g1_i1_1	4.506655	6.08803
DN4172_c0_g1_i1_1	0.4103366	3.510717
DN4172_c0_g1_i1_2	1.513471	1.666635
DN4174_c0_g1_i1_1	0.3174358	0.5519874
DN4174_c0_g2_i1_1	0.4648214	1.022863
DN4175_c0_g1_i1_2	10.44581	2.921179
DN4176_c0_g1_i1_1	2.769515	12.60994
DN4182_c0_g1_i1_2	5.863927	0.7581912
DN4183_c0_g1_i2_1	8.634588	13.73933
DN4197_c0_g1_i1_2	1.929533	1.265911
DN419_c0_g1_i1_1	0.3063975	0.6145947
DN419_c0_g1_i1_2	0.7407457	0.9401928
DN4202_c0_g1_i1_1	0.9216843	0.9131043
DN4204_c0_g1_i1_2	1.67916	1.076251
DN4206_c0_g1_i1_2	1.365964	0.3080904
DN420_c0_g1_i1_1	1.938747	3.027438

DN4217_c0_g1_i1_1	15.56426	23.70884
DN4218_c0_g1_i1_1	1.248947	1.457087
DN4219_c0_g1_i1_1	4.465574	7.250724
DN4219_c0_g1_i1_2	18.81373	3.073938
DN4226_c0_g1_i1_2	1.160338	0.4804032
DN4235_c0_g1_i1_1	1.06116	3.599622
DN4235_c0_g1_i1_2	1.48188	0.1776184
DN4236_c0_g1_i1_2	0.9987044	0.8403318
DN4237_c0_g1_i1_1	3.643278	2.298854
DN4238_c0_g1_i1_1	0.4979126	0
DN4242_c0_g1_i1_1	18.92251	6.783534
DN4254_c0_g1_i1_1	1.775046	0.7413696
DN4256_c0_g1_i1_1	0	0.2022372
DN4257_c0_g1_i1_1	1.23186	2.376321
DN4266_c0_g1_i1_1	0.9863952	0.9309339
DN426_c0_g1_i1_1	0.2976013	2.152214
DN426_c0_g2_i1_1	0	1.421039
DN4272_c0_g1_i1_2	0	0
DN4277_c0_g1_i1_1	2.141112	0.7555956
DN4277_c0_g2_i1_1	2.096001	1.282507
DN427_c0_g1_i1_2	0	0
DN4281_c0_g1_i1_2	0.2199491	0.6953829
DN4288_c0_g1_i1_1	0.9325972	2.847756
DN4290_c0_g1_i1_2	2.196768	0

DN4293_c0_g1_i1_1	0.4595454	1.444059
DN4304_c0_g1_i1_1	3.397628	2.264528
DN4307_c0_g1_i1_1	0.5165725	2.419643
DN4308_c0_g1_i1_2	1.057447	0.3218148
DN430_c0_g1_i1_1	0	2.709372
DN4314_c0_g1_i1_1	0.5191103	1.897331
DN4319_c0_g1_i1_2	1.040581	0.5037804
DN431_c0_g1_i1_2	1.399163	0
DN4332_c0_g1_i1_2	1.864881	1.971734
DN4332_c0_g2_i1_2	1.149913	1.154928
DN4333_c0_g1_i1_1	0.5893414	1.586132
DN4343_c0_g1_i1_1	1.356126	1.332686
DN4346_c0_g1_i2_1	6.196239	7.106775
DN4349_c0_g1_i1_2	4.609719	5.319233
DN4350_c0_g1_i1_2	1.457706	0.8779382
DN4351_c0_g1_i1_1	112.9143	79.67532
DN4352_c0_g1_i1_1	0.2841312	0.8955855
DN4361_c0_g1_i1_1	0.7183198	0.6164637
DN436_c0_g1_i1_1	0.5199428	2.146275
DN4372_c0_g1_i1_2	0	0
DN4382_c0_g1_i1_2	1.771819	0.1881844
DN4388_c0_g1_i1_2	1.931571	0.5683069
DN4394_c0_g1_i1_2	1.647624	1.457727
DN4395_c0_g1_i1_1	0.1741775	0.2609868

DN4397_c0_g1_i1_1	0.802504	0.4720753
DN4397_c0_g2_i1_1	1.654988	5.280756
DN4399_c0_g2_i1_1	2.05318	0.8177108
DN4402_c0_g1_i1_1	0	1.125906
DN4405_c0_g1_i1_1	0.433826	0.2313784
DN4406_c0_g1_i1_1	1.173076	1.754784
DN4409_c0_g1_i1_1	2.045484	3.784846
DN4411_c0_g1_i1_1	1.157654	1.769325
DN4424_c0_g1_i1_1	2.487918	1.493182
DN4425_c0_g1_i1_1	0.8181454	1.542268
DN4428_c0_g1_i2_1	2.244647	1.949456
DN4430_c0_g1_i1_1	1.435768	0.9961881
DN4435_c0_g1_i1_1	1.024223	1.559142
DN4435_c0_g1_i1_2	6.574459	55.84617
DN4437_c0_g1_i1_1	0	2.067454
DN4438_c0_g1_i1_1	0.05049787	0.5355762
DN443_c0_g1_i1_1	0	2.727938
DN4443_c0_g1_i1_1	0	3.726286
DN4443_c0_g1_i1_2	1.147135	0
DN444_c0_g1_i1_1	2.293542	0.3747805
DN4455_c0_g2_i1_1	0.5934862	1.587096
DN4458_c0_g1_i1_1	0.3166754	1.369236
DN4469_c0_g1_i1_2	6.407219	6.771362
DN4473_c0_g1_i1_1	2.021258	3.982482

DN4475_c0_g1_i1_1	0.419893	1.187753
DN4477_c0_g1_i1_2	0.6283656	1.59435
DN4480_c0_g1_i1_2	1.357408	0
DN4483_c0_g1_i1_1	0	0.3378605
DN4488_c0_g1_i1_1	1.29E-31	1.564915
DN4493_c0_g1_i1_2	2.512484	2.400162
DN4494_c0_g1_i1_2	1.299174	0
DN4494_c0_g2_i1_2	0.9605009	1.493315
DN4502_c0_g1_i2_2	8.445678	7.502099
DN4505_c0_g1_i1_1	0.7894121	3.914591
DN4506_c0_g1_i1_2	0	0.002444048
DN4507_c0_g1_i1_1	4.216136	2.850222
DN4508_c0_g1_i1_1	0.7910728	1.314011
DN4513_c0_g1_i1_1	0	0.94391
DN4515_c0_g1_i1_1	0.6312494	1.523658
DN4517_c0_g1_i2_1	3.454496	5.782257
DN4518_c0_g1_i1_1	14.60779	12.55394
DN4518_c0_g2_i1_2	2.093671	1.320088
DN4531_c0_g1_i1_1	4.154837	3.366249
DN4539_c0_g1_i1_2	2.167851	1.846778
DN4551_c0_g1_i1_2	2.536793	1.312523
DN4553_c0_g1_i1_2	0	0
DN4555_c0_g1_i1_1	1.927612	15.99954
DN4556_c0_g1_i1_2	3.432962	0.9889082

DN455_c0_g1_i1_1	1.229725	1.5378
DN455_c0_g1_i2_2	153.0384	188.1886
DN4566_c0_g1_i1_1	2.82835	2.380289
DN4569_c0_g1_i1_1	0.4596795	0.3695338
DN4570_c0_g1_i1_1	0.8084273	1.721708
DN4574_c0_g1_i1_2	1.664181	0.6926698
DN4577_c0_g1_i1_1	4.525116	7.910134
DN457_c0_g1_i2_2	1.891343	0.3186937
DN4584_c0_g1_i1_1	2.553827	3.303645
DN4585_c0_g1_i1_2	24.05043	7.704613
DN4586_c0_g1_i1_1	1.344546	1.319582
DN4586_c0_g1_i1_2	0.4822405	0
DN4591_c0_g1_i1_1	1.28763	2.311513
DN4592_c0_g1_i1_2	6.782804	5.870938
DN4594_c0_g1_i1_1	1.649244	1.516001
DN4595_c0_g1_i1_1	1.063727	0.7715062
DN4607_c0_g1_i1_2	0.8314786	1.03E-26
DN4613_c0_g1_i1_1	7.088553	5.642586
DN4619_c0_g1_i1_1	2.692609	1.926173
DN4621_c0_g1_i1_1	10.65502	5.520328
DN4623_c0_g1_i1_1	1.577139	2.106658
DN4624_c0_g1_i1_1	0.9815546	2.150895
DN4625_c0_g1_i1_1	0.3606306	1.616503
DN4638_c0_g1_i1_1	3.172329	13.25558

DN4641_c0_g1_i1_2	2.487112	1.593251
DN4642_c0_g1_i1_1	5.15E-31	1.850147
DN4647_c0_g1_i2_1	1.965538	3.757804
DN464_c0_g1_i1_1	0.2286354	0.4801248
DN464_c0_g1_i1_2	0.2421739	0.1386878
DN4663_c0_g1_i1_1	0.414418	2.839795
DN4671_c0_g1_i1_2	0.1167121	1.888471
DN4673_c0_g1_i1_1	0.1635734	0.3132622
DN4675_c0_g1_i1_1	1.531947	1.866309
DN4680_c0_g1_i1_1	54.94572	36.75576
DN4681_c0_g1_i1_2	19.52813	13.19766
DN4681_c0_g2_i1_2	8.141901	0.9573126
DN4684_c0_g1_i2_2	41.09516	0.514977
DN4687_c0_g1_i2_2	2.793154	2.88E-08
DN4688_c0_g1_i1_1	0.7897234	1.529842
DN468_c0_g1_i1_1	2.033899	0.3531888
DN4691_c0_g1_i1_1	11.10256	6.066607
DN4694_c0_g1_i1_2	0	2.276048
DN4712_c0_g1_i1_1	1.755454	0.9340783
DN4715_c0_g1_i1_1	0.8518797	3.605107
DN4717_c0_g1_i1_1	0.5614568	0.1192862
DN4718_c0_g1_i1_1	2.027306	0.8612537
DN4738_c0_g1_i1_2	2.510637	0.1534509
DN4740_c0_g1_i1_1	1.096971	0.5748683

DN4742_c0_g1_i1_2	3.093445	1.591764
DN4749_c0_g1_i1_2	3.041438	2.455444
DN4751_c0_g1_i1_2	7.131501	5.642632
DN4754_c0_g1_i1_1	2.239445	2.292635
DN4758_c0_g1_i1_2	3.048478	0.6897815
DN4777_c0_g1_i1_1	1.46122	0.8492139
DN4779_c0_g2_i1_2	1.687494	1.216664
DN477_c0_g1_i1_1	0.5033034	0.7057136
DN477_c0_g2_i1_1	0	1.200627
DN4781_c0_g1_i1_1	0.7756191	3.571431
DN4781_c0_g1_i1_2	8.988328	2.364829
DN4796_c0_g1_i1_2	0.8695101	0.1802123
DN4796_c0_g2_i1_2	0.5711244	0.1160076
DN4797_c0_g1_i1_1	0.6205303	0
DN4797_c0_g1_i1_2	4.808416	2.56609
DN4799_c0_g1_i1_1	1.027596	2.195793
DN4805_c0_g1_i1_2	5.541107	4.986684
DN4805_c0_g1_i2_1	15.09265	10.32289
DN4812_c0_g1_i1_1	0.4141086	0.8776319
DN4816_c0_g1_i2_1	29.62815	102.6125
DN4819_c0_g1_i1_1	3.488056	5.164316
DN4822_c0_g1_i1_2	2.465813	1.526818
DN4826_c0_g1_i1_1	0.5537901	0.8076975
DN4831_c0_g1_i1_2	1.886471	1.034214

DN4835_c0_g1_i1_1	2.237744	1.282079
DN4840_c0_g1_i1_1	1.83737	1.82984
DN4843_c0_g1_i1_2	50.27216	80.71298
DN4848_c0_g1_i1_1	2.752606	3.119815
DN4848_c0_g1_i1_2	6.007028	2.240999
DN4853_c0_g1_i1_1	0.574999	4.747661
DN4854_c0_g1_i1_2	1.385702	1.577356
DN4855_c0_g1_i1_1	6.778169	9.01626
DN4860_c0_g1_i1_1	0.9627351	0.8154931
DN486_c0_g1_i1_1	8.048877	24.08239
DN4870_c0_g2_i1_2	66.63176	121.911
DN4874_c0_g2_i1_2	0.927101	0
DN4876_c0_g1_i1_2	258.101	7.125197
DN4877_c0_g1_i1_2	1.560935	0.7454635
DN4885_c0_g1_i1_1	1.685541	1.688231
DN4888_c0_g1_i1_1	1.57717	0.6525183
DN4888_c0_g1_i1_2	2.137075	5.836881
DN4889_c0_g1_i1_2	1.947026	0.7405922
DN488_c0_g1_i1_1	0.9661655	4.508015
DN488_c0_g1_i1_2	1.32E-28	7.73E-31
DN4892_c0_g1_i1_2	152.7758	185.5878
DN4893_c0_g1_i1_1	1.8094	1.128731
DN4896_c0_g1_i1_1	0.1562197	0.4322922
DN4905_c0_g1_i1_1	0.5530268	1.100887

DN4905_c0_g2_i1_1	0.8157089	1.247991
DN4906_c0_g1_i1_1	1.553136	2.401499
DN4906_c0_g1_i1_2	15.34889	12.9832
DN4909_c0_g1_i1_1	0.2784045	1.820145
DN4912_c0_g1_i1_1	3.368534	2.15562
DN4913_c0_g1_i1_2	3.757787	1.540039
DN4914_c0_g1_i1_1	5.900143	2.375547
DN4915_c0_g1_i1_1	0.9877721	0.8870336
DN4920_c0_g1_i1_1	0.2545578	1.208612
DN4920_c0_g1_i1_2	1.625572	0.5306285
DN4920_c0_g2_i1_1	0.6732018	1.835931
DN4927_c0_g1_i1_1	5.13999	2.424538
DN4928_c0_g1_i1_1	2.015296	1.769957
DN4933_c0_g1_i1_1	7.556228	7.453796
DN4935_c0_g1_i1_1	0.1749867	0
DN4939_c0_g1_i1_1	2.213859	12.41011
DN4948_c0_g1_i2_1	9.582874	17.60257
DN4955_c0_g1_i1_1	1.305705	3.537586
DN4958_c0_g1_i1_1	0	1.845175
DN4958_c0_g2_i1_1	0	1.753687
DN495_c0_g1_i1_1	0.4643745	2.59E-12
DN4969_c0_g1_i1_1	2.255841	1.50918
DN4970_c0_g1_i1_1	6.431003	3.59348
DN4981_c0_g1_i1_1	4.134586	0.8495702

DN4982_c0_g2_i1_1	0.9418235	0.8703343
DN4985_c0_g1_i1_1	1.192358	1.937887
DN4986_c0_g1_i1_1	1.814986	2.033364
DN4986_c0_g1_i1_2	1.870523	1.020556
DN4993_c0_g1_i1_1	25.91073	20.85347
DN4996_c0_g1_i1_1	3.275571	1.9442
DN4998_c0_g1_i1_1	1.171856	1.402925
DN5002_c0_g1_i1_2	3.249521	4.412348
DN5004_c0_g1_i1_1	1.60338	2.151859
DN5004_c0_g2_i1_1	1.225187	2.153552
DN5006_c0_g1_i1_1	0.5137491	1.694853
DN5006_c0_g2_i1_1	0.4116801	2.475532
DN5007_c0_g1_i1_1	8.143087	4.607368
DN5008_c0_g1_i1_1	0.2388316	2.147371
DN5010_c0_g1_i1_1	14.55184	12.41818
DN5014_c0_g1_i1_1	1.829569	0.8530489
DN5018_c0_g1_i1_1	0	0
DN501_c0_g1_i1_1	0.1217629	1.280168
DN5025_c0_g1_i1_2	1.655657	1.394379
DN5027_c0_g1_i1_1	0.6644487	0.6613468
DN5029_c0_g1_i1_1	2.145482	1.795011
DN5033_c0_g1_i1_1	24.60763	340.0885
DN5035_c0_g1_i1_1	13.31648	4.256317
DN5038_c0_g1_i1_2	0.7960757	0

DN5042_c0_g1_i1_1	0.8379678	1.778155
DN5042_c0_g2_i1_1	0.7361057	1.901517
DN5043_c0_g1_i1_1	0.9090337	11.20178
DN5045_c0_g1_i2_1	12.06364	5.807836
DN5046_c0_g1_i1_1	2.312446	5.138765
DN5052_c0_g1_i1_1	0.2651389	1.518028
DN5057_c0_g1_i1_1	0.692341	2.159227
DN5058_c0_g1_i1_2	1.226862	0.6994805
DN5060_c0_g1_i1_1	0.1602044	2.417749
DN5062_c0_g1_i1_1	0.3775155	1.404727
DN5066_c0_g1_i1_1	0.2818642	2.103636
DN5068_c0_g1_i1_1	2.879816	2.297251
DN5074_c0_g1_i1_1	0.4196468	1.518564
DN5081_c0_g1_i2_1	3.823708	6.347414
DN5082_c0_g1_i1_1	0.4639759	0.5992321
DN5083_c0_g1_i1_2	2.37843	0.5402836
DN5084_c0_g1_i1_1	1.069383	2.067781
DN5089_c0_g1_i1_2	1.722841	1.224087
DN5090_c0_g1_i2_2	32.32422	3.873108
DN5098_c0_g1_i1_1	0.1638516	0.04965807
DN509_c0_g1_i1_1	4.664598	3.734444
DN509_c0_g1_i2_2	9.250513	13.35042
DN509_c0_g2_i1_1	3.775119	3.292672
DN50_c0_g1_i1_2	0.7815178	0.8054026

DN5101_c0_g1_i1_1	1.240964	1.451017
DN5102_c0_g1_i1_1	0.8844312	4.380952
DN5111_c0_g1_i1_2	1.72E-30	1.89E-16
DN5117_c0_g1_i1_1	0.4424891	1.933288
DN5117_c0_g2_i1_1	1.19649	2.643489
DN5118_c0_g1_i1_2	8.91164	6.710465
DN5118_c0_g2_i1_2	3.711013	2.173091
DN511_c0_g1_i1_1	0.4435029	1.234188
DN5129_c0_g1_i1_1	1.04952	3.518667
DN5135_c0_g1_i1_1	0.9093628	9.86E-06
DN5137_c0_g2_i1_1	0.2519676	2.337856
DN5138_c0_g1_i1_2	2.060944	0
DN513_c0_g1_i1_1	0.4769364	0.5181174
DN5142_c0_g1_i1_1	1.625223	0.8398169
DN5147_c0_g1_i1_1	0.7176695	2.134714
DN5149_c0_g1_i1_1	1.670114	0.2160174
DN5153_c0_g2_i1_2	2.918009	1.223
DN515_c0_g1_i1_1	0.6756889	1.130197
DN515_c0_g1_i1_2	6.290094	2.429034
DN515_c0_g2_i1_1	1.323723	1.59015
DN5161_c0_g2_i1_2	20.77919	17.7665
DN5162_c0_g1_i1_1	2.220368	1.220891
DN5165_c0_g1_i1_1	1.075663	0.3896877
DN5167_c0_g1_i1_2	1.414076	1.219482

DN516_c0_g1_i1_2	6.530965	15.54642
DN5172_c0_g1_i1_2	13.59821	43.63814
DN5174_c0_g1_i2_1	3.170379	3.042723
DN5185_c0_g1_i1_2	0.925056	0
DN5186_c0_g1_i1_2	4.851127	0.7682127
DN5192_c0_g1_i1_1	1.744712	1.206744
DN5205_c0_g1_i1_1	32.24147	48.84545
DN5206_c0_g1_i1_1	1.165226	1.406085
DN5206_c0_g1_i1_2	1.310183	1.468516
DN5208_c0_g1_i1_1	0.2465026	0.2470985
DN5209_c0_g1_i1_1	0	0.2143501
DN5211_c0_g1_i1_1	0	1.11E-99
DN5212_c0_g1_i1_2	0.2131461	5.65E-26
DN5217_c0_g1_i1_2	4.501291	3.584771
DN5218_c0_g1_i1_2	2.343204	1.345171
DN5218_c0_g2_i1_2	5.253761	1.706831
DN521_c0_g1_i1_2	0.586009	0
DN5221_c0_g1_i1_1	3.085961	4.660479
DN5223_c0_g1_i1_1	0	1.53742
DN5224_c0_g1_i1_1	1.111398	1.700836
DN5224_c0_g2_i1_2	2.273832	0.6824052
DN5225_c0_g1_i2_2	2.259032	2.334684
DN5237_c0_g1_i1_1	0.5298733	0.7279602
DN5238_c0_g1_i1_1	0.8486078	0.855399

DN5238_c0_g1_i1_2	0	0
DN5239_c0_g1_i1_2	2.286699	1.405059
DN5239_c0_g2_i1_2	5.714771	1.677792
DN524_c0_g1_i1_2	0	0
DN5256_c0_g1_i1_1	0	0.3102698
DN525_c0_g1_i1_1	1.952445	1.116128
DN5261_c0_g1_i1_1	2.068021	4.39531
DN5269_c0_g1_i1_1	4.371789	3.056344
DN5272_c0_g1_i1_2	0.917978	0.8787353
DN5277_c0_g1_i1_1	1.821134	0.6701913
DN527_c0_g1_i1_2	0	0
DN5280_c0_g1_i1_1	0	2.388315
DN5283_c0_g1_i1_1	0.2407791	1.243526
DN5286_c0_g1_i1_1	7.36157	2.757171
DN5287_c0_g1_i1_2	1.67401	0.8218262
DN528_c0_g1_i1_1	179.1117	118.1699
DN5291_c0_g1_i1_1	1.128584	0.8119754
DN5291_c0_g1_i1_2	950.847	799.4473
DN5298_c0_g1_i1_1	7.17E-22	1.009072
DN529_c0_g1_i1_1	1.027779	1.369544
DN529_c1_g1_i1_1	1.218189	2.196802
DN5300_c0_g1_i1_2	2.356246	0.9488518
DN5301_c0_g1_i1_2	1.560535	0.3682501
DN5302_c0_g1_i1_2	1.528089	1.533474

DN5303_c0_g1_i1_2	0	0
DN5307_c0_g1_i1_1	0	0
DN5309_c0_g1_i1_2	1.736407	0.686238
DN5309_c0_g1_i2_1	1.980108	1.514245
DN530_c0_g1_i1_1	0	6.829802
DN530_c0_g2_i1_1	1.198096	3.321885
DN5310_c0_g1_i1_1	3.917043	7.732824
DN5311_c0_g1_i1_1	1.183711	1.614162
DN5312_c0_g1_i1_2	0	0
DN5316_c0_g1_i1_2	1.458368	0.7200156
DN5317_c0_g1_i1_2	5.158877	3.53808
DN5323_c0_g2_i1_1	0.5397888	0.6835386
DN5327_c0_g1_i1_1	0.03112486	0
DN5330_c0_g1_i1_1	1.30467	1.248134
DN5331_c0_g1_i1_1	0.2048676	0.6507618
DN5332_c0_g1_i1_1	0.7264333	6.613422
DN5335_c0_g1_i1_1	1.194264	3.381803
DN5337_c0_g1_i1_2	2.128458	3.302319
DN533_c0_g1_i1_2	0.5123908	1.025407
DN5341_c0_g1_i1_1	1.750906	0.5706232
DN5343_c0_g1_i1_2	0	0
DN5344_c0_g1_i1_1	2.770494	1.865106
DN5346_c0_g1_i1_2	0.8583748	0
DN5348_c0_g1_i1_1	0.7194186	6.717259

DN5349_c0_g1_i1_2	1.752696	2.172589
DN5351_c0_g1_i1_2	7.759317	2.25093
DN5353_c0_g1_i1_1	0.7900714	3.14E-10
DN5356_c0_g1_i1_1	1.132065	1.499772
DN5357_c0_g1_i1_1	0.9023169	1.293711
DN5358_c0_g1_i1_1	0.2596798	2.809839
DN535_c0_g1_i1_1	0.1615788	0.1480342
DN5364_c0_g1_i1_2	0	1.092981
DN5365_c0_g1_i1_1	1.915297	2.071473
DN5367_c0_g1_i1_1	0.7267991	3.547132
DN5371_c0_g1_i2_1	0.6365246	1.409424
DN5373_c0_g1_i1_2	4.389486	0.1735086
DN5375_c0_g1_i1_2	0	0
DN5378_c0_g1_i2_1	0.6912811	0.6999269
DN5381_c0_g1_i1_1	2.194573	2.519344
DN5384_c0_g1_i1_1	3.280375	12.00979
DN5384_c0_g1_i1_2	0	0
DN538_c0_g1_i1_1	0.7284451	0.2758628
DN538_c0_g1_i1_2	2.797089	2.593021
DN5393_c0_g1_i1_1	1.390279	1.245777
DN5393_c0_g2_i1_1	1.296832	0.6292206
DN5400_c0_g1_i2_2	12.50563	27.7747
DN5411_c0_g1_i1_1	1.55767	1.633474
DN5420_c0_g1_i1_1	2.35715	6.386759

DN5421_c0_g1_i1_1	0.6208146	4.955504
DN5424_c0_g1_i1_1	0.7043363	2.090801
DN5424_c0_g1_i1_2	18.00653	28.23581
DN5426_c0_g1_i1_2	3.449893	0.2652702
DN5433_c0_g1_i1_1	0.2872314	3.870666
DN5434_c0_g1_i1_1	1.523638	1.710552
DN5442_c0_g1_i2_2	2.463882	10.23581
DN5449_c0_g1_i1_1	1.67082	3.397953
DN5449_c0_g1_i1_2	0.6253405	0
DN544_c0_g1_i1_2	0	0
DN5450_c0_g1_i1_1	1.599759	2.049264
DN5451_c0_g1_i1_1	10.35176	22.31138
DN5458_c0_g1_i1_1	0.7871129	1.493989
DN5459_c0_g1_i1_2	2.386621	0.7981788
DN5460_c0_g1_i1_2	0.8202232	1.726243
DN5467_c0_g1_i1_1	2.365024	2.034661
DN5468_c0_g1_i1_1	20.3745	20.02587
DN5472_c0_g1_i1_1	0.5901134	1.701829
DN5472_c0_g1_i1_2	2.832833	0.8267233
DN5486_c0_g1_i1_1	11.89853	21.06002
DN5486_c0_g1_i1_2	1.607596	0.7429204
DN5490_c0_g1_i1_1	0	14.92441
DN5495_c0_g1_i1_2	1.671842	1.125211
DN54_c0_g1_i1_2	3.428983	0.5256765

DN5501_c0_g1_i1_2	2.361034	2.180239
DN5502_c0_g1_i1_1	1.39037	1.7613
DN5505_c0_g1_i1_1	0.4781807	2.496112
DN5513_c0_g1_i1_2	1.471946	0
DN5514_c0_g1_i1_2	2.95451	1.702626
DN5515_c0_g1_i1_1	0.5763492	1.835148
DN5520_c0_g1_i1_1	1.219231	0.2664903
DN5522_c0_g1_i2_1	2.720841	7.188278
DN5535_c0_g1_i1_1	0.5453428	0.4190954
DN5541_c0_g1_i1_1	0	1.069805
DN5541_c0_g2_i1_1	0.1014358	0.9619656
DN5543_c0_g1_i1_1	0.7087352	1.880573
DN5543_c0_g1_i1_2	2.03982	1.165896
DN5548_c0_g1_i1_1	2.060327	0.9075576
DN5549_c0_g1_i1_1	0.4943296	14.4525
DN5550_c0_g1_i1_2	1.566501	1.882706
DN5551_c0_g1_i1_1	0.709435	1.016454
DN5553_c0_g1_i1_2	1.210275	1.323131
DN5555_c0_g1_i1_1	0.7875965	1.033018
DN5556_c0_g1_i1_1	5.01E-11	6.89E-06
DN5557_c0_g1_i1_2	1.151176	2.303431
DN5560_c0_g1_i1_1	110.7143	61.15398
DN5570_c0_g1_i1_1	0	1.32251
DN5571_c0_g1_i1_1	0	0

DN5573_c0_g1_i1_1	0.2969577	1.187855
DN5577_c0_g1_i1_1	1.594263	1.729818
DN5578_c0_g1_i1_2	16.10171	14.37098
DN5581_c0_g1_i1_1	2.046105	2.250452
DN5582_c0_g1_i1_1	1.141548	4.513745
DN5582_c0_g2_i1_1	1.176507	2.628398
DN5603_c0_g1_i1_1	1.425577	1.097121
DN5604_c0_g1_i1_2	5.568772	1.575623
DN5605_c0_g1_i1_1	1.682699	3.7108
DN5607_c0_g1_i1_1	1.285927	5.414877
DN5609_c0_g1_i1_1	0.4689257	1.00565
DN5613_c0_g1_i1_1	0.3794019	0.9939666
DN5617_c0_g1_i1_2	0	0
DN5618_c0_g1_i1_2	2.465354	3.333219
DN562_c0_g1_i1_1	1.927574	1.885034
DN5632_c0_g1_i2_1	1.303107	1.877314
DN5646_c0_g1_i1_1	2.096177	5.190569
DN5650_c0_g1_i1_1	210.1871	239.9352
DN5654_c0_g1_i1_2	3.267694	0
DN5657_c0_g1_i1_2	21.55819	12.55754
DN5660_c0_g1_i1_2	1.694661	2.589599
DN5661_c0_g1_i1_1	0.8805688	2.501524
DN5670_c0_g1_i1_1	0.6965838	3.505314
DN5671_c0_g1_i1_1	1.801791	1.032028

DN5674_c0_g1_i1_1	0	0.3153326
DN5679_c0_g1_i2_1	23.48958	90.97736
DN5684_c0_g1_i1_1	2.037632	0
DN5685_c0_g1_i1_2	1.576788	0.2750162
DN5689_c0_g1_i1_1	5.294538	5.508398
DN568_c0_g1_i1_2	6.83473	2.625071
DN5692_c0_g1_i1_1	1.066689	1.731485
DN5692_c0_g2_i1_1	0.7965167	1.765311
DN5696_c0_g1_i1_1	2.374107	2.725939
DN5697_c0_g1_i1_1	1.169554	2.037495
DN56_c0_g1_i1_1	1.397226	2.545713
DN56_c0_g1_i1_2	15.33407	9.367655
DN56_c0_g2_i1_1	2.439402	3.787536
DN5703_c0_g1_i2_1	0.7499148	3.837388
DN5704_c0_g1_i1_1	0.7481735	1.324098
DN5704_c0_g2_i1_1	0.6689212	1.353862
DN5709_c0_g1_i1_2	0	0
DN5710_c0_g1_i1_1	1.146449	0.09496214
DN5712_c0_g1_i1_1	0.9938882	0.2011425
DN5713_c0_g1_i1_2	50.96173	44.84489
DN5718_c0_g1_i1_1	1.603654	0.7274048
DN5718_c0_g2_i1_1	0	1.788028
DN5723_c0_g1_i1_1	2.976733	0.604543
DN5725_c0_g1_i1_1	7257.173	6229.689

DN5726_c0_g1_i2_2	6.207538	8.553027
DN5728_c0_g1_i1_1	0.881764	0.7389742
DN572_c0_g1_i1_1	2.647546	3.02309
DN5730_c0_g1_i1_1	3.619541	2.963654
DN573_c0_g1_i2_1	0.3046297	1.218664
DN5745_c0_g1_i1_1	2.271514	2.333051
DN5745_c0_g1_i1_2	1.092204	2.865722
DN5747_c0_g1_i1_1	1.767895	2.592008
DN5749_c0_g1_i1_2	10.26818	5.65477
DN574_c0_g1_i1_1	0.3885087	11.69338
DN5767_c0_g1_i1_1	0.9000703	3.203534
DN5771_c0_g1_i1_1	0.823944	0.0970024
DN5772_c0_g1_i1_2	4.774352	0.3760763
DN5776_c0_g1_i1_1	2.005522	1.78704
DN5776_c0_g1_i1_2	91.24231	18.15617
DN5788_c0_g1_i1_1	1.546554	1.176669
DN5788_c0_g2_i1_1	1.159343	1.303818
DN5795_c0_g1_i1_1	1.73096	3.149781
DN5797_c0_g2_i1_1	3.29887	3.883837
DN5807_c0_g2_i1_1	0.2816723	0.1664961
DN5819_c0_g1_i1_2	1.545432	0.3393609
DN5820_c0_g1_i1_1	0.7242008	0.6007397
DN5821_c0_g1_i2_1	5.97852	16.41206
DN5822_c0_g1_i1_2	0.531958	1.092968

DN5825_c0_g1_i1_1	1.410276	2.58562
DN5826_c0_g1_i1_1	0.2412106	0.4545718
DN5827_c0_g1_i1_2	0.6276229	0.2373266
DN5830_c0_g1_i1_1	1.467475	1.259247
DN5841_c0_g1_i1_1	27.00605	28.43253
DN5845_c0_g1_i1_1	0.3877806	0.6557355
DN5854_c0_g1_i1_1	1.548116	1.730395
DN5856_c0_g1_i2_2	1.99983	1.722394
DN5859_c0_g1_i1_1	0.8721903	1.784235
DN585_c0_g1_i2_2	12.48491	10.55657
DN5865_c0_g1_i2_1	2.973867	4.635492
DN5873_c0_g1_i1_2	1.766094	0.002575893
DN5878_c0_g1_i1_1	12.27974	12.46859
DN5882_c0_g1_i1_1	0.5323372	0.2382595
DN5884_c0_g1_i1_2	0	0
DN5888_c0_g1_i1_1	0.36965	1.117982
DN5888_c0_g2_i1_1	2.617638	1.218173
DN5896_c0_g1_i1_2	0	0
DN589_c0_g1_i1_1	10.2737	12.47341
DN58_c0_g1_i1_1	0.4663543	1.976388
DN5905_c0_g1_i1_1	0.5930876	0.9749092
DN5905_c1_g1_i1_1	1.028046	2.41269
DN5911_c0_g1_i1_2	7.844507	3.792075
DN5914_c0_g1_i1_1	0.9957383	2.140998

DN5923_c0_g1_i1_2	0.1874572	0.2970302
DN5928_c0_g1_i1_2	1.176356	0
DN5931_c0_g1_i1_2	2.495216	0.3785113
DN5935_c0_g1_i1_1	1.308574	1.419481
DN5937_c0_g1_i1_1	0.6935198	0.7897315
DN5937_c0_g2_i1_1	0.1333786	0.5052379
DN5951_c0_g1_i1_1	1.676172	3.381052
DN5953_c0_g1_i1_2	5.024718	2.980886
DN5955_c0_g1_i1_1	0.7193523	4.758567
DN5962_c0_g1_i1_2	2.231018	2.681995
DN5966_c0_g1_i1_2	2.026261	0.8181577
DN5968_c0_g1_i1_1	1.279081	3.672705
DN5968_c0_g1_i1_2	0.5266588	3.76E-29
DN5968_c0_g2_i1_1	2.228735	6.076844
DN596_c0_g1_i1_2	0.9249058	1.075179
DN5973_c0_g1_i1_1	0.3421564	18.29927
DN5973_c0_g1_i1_2	1.210853	0
DN5973_c0_g2_i1_1	0	41.28173
DN5973_c0_g2_i1_2	1.861266	2.433144
DN5974_c0_g1_i2_1	0.5176391	2.762953
DN5978_c0_g1_i1_1	2.865085	1.28172
DN5982_c0_g1_i1_2	2.188339	1.02806
DN5987_c0_g1_i1_1	0.7317294	1.08179
DN5987_c0_g2_i1_1	0.7315484	1.503792

DN5989_c0_g1_i1_1	0.4246523	0.6050654
DN5994_c0_g1_i1_1	1.851466	0.7857003
DN5995_c0_g1_i1_1	1.009081	2.137342
DN5999_c0_g1_i1_1	1.778388	0.818273
DN6007_c0_g1_i2_2	1.906041	1.07471
DN6008_c0_g1_i1_2	5.503262	3.962967
DN600_c0_g1_i1_1	3.931032	4.495753
DN6010_c0_g1_i1_1	0	1.490036
DN6010_c0_g2_i1_1	1.022689	0.8774933
DN6013_c0_g1_i1_1	0.9446663	2.825571
DN6015_c0_g1_i1_1	3.416478	2.937195
DN6019_c0_g1_i1_1	0.383761	0.7305985
DN6020_c0_g1_i1_2	0.2574368	0.6495663
DN6021_c0_g1_i1_1	15.50363	25.73114
DN6022_c0_g1_i1_1	0.307371	1.256063
DN6024_c0_g1_i2_1	1.594457	4.813262
DN6028_c0_g1_i2_1	3.9953	5.633671
DN6034_c0_g1_i1_1	4.70917	8.805411
DN6035_c0_g1_i1_1	0.2841175	1.896323
DN603_c0_g1_i1_1	2.880879	2.828122
DN6041_c0_g1_i1_2	1.752194	0
DN6044_c0_g1_i1_1	0.6132997	1.117841
DN604_c0_g1_i1_2	3.336676	1.259986
DN6051_c0_g1_i1_1	0.6595837	0.973577

DN6052_c0_g1_i1_1	0.7419143	0.6837794
DN6059_c0_g1_i1_1	2.42498	5.335944
DN6063_c0_g1_i1_2	2.816703	0.3593829
DN6068_c0_g1_i1_1	1.45348	3.744123
DN6068_c0_g2_i1_1	1.068522	2.819778
DN6070_c0_g1_i1_1	7.0682	4.904151
DN6072_c0_g1_i2_1	1.383353	1.495807
DN607_c0_g1_i1_2	0.8819739	0.6738682
DN6080_c0_g1_i1_2	1.048707	0.7747132
DN6081_c0_g1_i1_2	2.668757	1.19414
DN6085_c0_g1_i1_1	0.7318446	0.3006712
DN6088_c0_g1_i2_1	0.8231618	2.199139
DN608_c0_g1_i1_1	0.7538004	1.345893
DN6090_c0_g1_i1_1	0.3337513	0.6467646
DN6091_c0_g1_i1_1	13.74768	4.140015
DN6099_c0_g1_i1_1	1.020464	3.095992
DN609_c0_g2_i1_2	23.03297	2.89573
DN6103_c0_g1_i1_1	0.5519885	0.5888679
DN6105_c0_g1_i1_1	0.469791	2.038809
DN6108_c0_g2_i1_1	1.843209	2.221876
DN6109_c0_g1_i1_2	23.23491	3.605219
DN610_c0_g1_i1_2	3.818209	0.8682656
DN610_c0_g2_i1_2	10.72097	4.564568
DN6110_c0_g1_i1_1	1.53093	3.187762

DN6114_c0_g1_i2_1	3.09931	2.569346
DN6118_c0_g1_i1_2	2.597082	0.2018983
DN6121_c0_g1_i1_2	0	0.832993
DN6124_c0_g1_i1_1	0.903854	3.883424
DN6125_c0_g1_i2_1	2.037278	56.60104
DN6132_c0_g1_i1_1	1.275262	1.438386
DN6134_c0_g1_i1_2	1.789296	1.345022
DN6135_c0_g1_i1_1	2.171413	0.1699749
DN613_c0_g1_i1_1	0.4088473	0.8760955
DN6142_c0_g1_i2_1	1.8466	1.004428
DN6143_c0_g1_i1_1	1.665206	2.563677
DN6147_c0_g1_i1_1	1.528544	5.841481
DN6149_c0_g1_i1_1	0	0
DN6152_c0_g1_i1_1	0.7833227	2.480646
DN6152_c0_g1_i1_2	3.29E-27	0.221718
DN6154_c0_g2_i1_1	4.717848	5.448917
DN6155_c0_g1_i1_2	1.577124	1.518247
DN6161_c0_g1_i1_1	0.7401031	0.9552039
DN6167_c0_g1_i1_1	1.23262	0.6406058
DN6168_c0_g1_i1_2	2.615719	4.304671
DN616_c0_g1_i1_2	4.816069	2.997329
DN6171_c0_g1_i1_2	2.665766	0.7155083
DN6175_c0_g1_i1_1	1.47E-30	0.1608285
DN6176_c0_g1_i1_1	1.844798	0.4962621

DN6179_c0_g2_i1_2	1.384139	0.8102004
DN617_c0_g1_i1_1	0.1130512	0
DN6185_c0_g1_i1_2	0.5154698	1.241679
DN618_c0_g1_i1_2	2.752287	1.134057
DN6191_c0_g2_i1_1	0.1290077	0.9889045
DN6194_c0_g1_i1_2	0.5253882	0
DN6200_c0_g1_i1_1	0	1.687452
DN6203_c0_g1_i1_1	0.5496024	2.363028
DN6205_c0_g1_i1_1	0.7452358	0.05960087
DN6206_c0_g1_i1_1	0.3606373	2.490166
DN6207_c0_g1_i1_1	2.493938	2.125863
DN6209_c0_g1_i1_1	0.5329101	1.651657
DN6213_c0_g2_i1_1	1.829842	11.78935
DN6221_c0_g1_i1_1	1.396228	0.8577185
DN6222_c0_g1_i1_1	0	0.5972356
DN6229_c0_g1_i1_1	0.2214936	1.249447
DN6230_c0_g1_i1_2	0.4943749	0
DN6233_c0_g1_i1_1	13.18103	24.42904
DN6234_c0_g1_i1_2	0.6017644	0.3524769
DN6235_c0_g1_i1_1	0.9242533	1.263632
DN6236_c0_g1_i1_1	0.4232709	1.149648
DN6243_c0_g1_i1_1	0.1474758	0.9169416
DN6245_c0_g1_i1_1	1.496998	2.618742
DN6250_c0_g1_i1_1	0.2682905	0.1212828

DN6251_c0_g1_i1_1	50.55705	16.28938
DN6253_c0_g1_i1_1	0.1380777	0.8895193
DN6256_c0_g1_i1_2	3.19676	0.4178988
DN6269_c0_g1_i1_2	0.3888218	0.3774533
DN626_c0_g1_i1_2	1.939288	1.302448
DN6272_c0_g1_i1_1	0.4902114	1.03E-28
DN6278_c0_g1_i1_1	808.3223	1320.836
DN627_c0_g1_i1_2	5.118861	2.227092
DN6283_c0_g1_i1_1	0.2482433	1.750256
DN6289_c0_g1_i1_2	1.17633	0.5068106
DN6290_c0_g1_i1_1	2.68631	4.485884
DN6292_c0_g1_i1_1	0.3046419	2.41646
DN6295_c0_g1_i2_1	3.493007	2.227168
DN6297_c0_g1_i1_1	1.505991	0.9241093
DN629_c0_g1_i1_1	2.057312	4.916147
DN6302_c0_g1_i1_2	0.2992933	0
DN6307_c0_g1_i1_2	8.443257	6.457037
DN6311_c0_g1_i1_1	0.9519009	0.9258903
DN6312_c0_g1_i1_1	0	0
DN6314_c0_g1_i1_1	7.461824	3.984879
DN6322_c0_g1_i1_1	1.41552	2.582721
DN6324_c0_g1_i1_2	3.930704	1.481531
DN6329_c0_g1_i1_1	1.586962	2.024051
DN6333_c0_g1_i1_1	0.3075867	3.233821

DN6342_c0_g1_i1_1	3.627988	2.817044
DN6343_c0_g1_i1_1	3.172184	2.606171
DN6346_c0_g1_i1_1	0.3305552	0.3420698
DN634_c0_g1_i1_2	2.483951	2.706075
DN6351_c0_g1_i1_2	11.45723	7.291679
DN6356_c0_g1_i1_2	2.575423	2.980134
DN6358_c0_g1_i1_2	0.3495007	0
DN635_c0_g1_i1_1	1.065285	0
DN6365_c0_g1_i1_2	0.8145446	0.3443845
DN6368_c0_g1_i1_1	1.246342	2.652677
DN6368_c0_g1_i1_2	1.034201	0.2172479
DN6371_c0_g1_i1_1	2.431529	3.802133
DN6374_c0_g1_i1_1	1.650586	1.96306
DN6379_c0_g1_i1_2	0.2094119	0
DN6382_c0_g1_i1_2	1.162458	0.8389186
DN6384_c0_g2_i1_1	1.341224	2.351731
DN6389_c0_g1_i1_1	0.5802558	2.243868
DN638_c0_g1_i1_1	9.83E-15	7.39E-12
DN6398_c0_g1_i1_2	6.629434	1.139522
DN63_c0_g1_i1_1	0.3563584	1.939027
DN6411_c0_g1_i1_2	1.034919	0.602314
DN6412_c0_g1_i1_2	0.2091972	0.140297
DN6424_c0_g1_i1_2	3.681103	0.3599367
DN6438_c0_g1_i1_1	7.624661	1.826114

DN643_c0_g1_i1_1	3.609105	23.08311
DN6440_c0_g1_i1_1	0.3130998	2.343566
DN6444_c0_g1_i1_1	0.3859327	1.369566
DN6449_c0_g1_i1_1	1.041086	0.4771474
DN6451_c0_g1_i1_1	0.6942049	5.942596
DN6452_c0_g1_i1_1	2.698896	6.67984
DN6452_c0_g1_i1_2	1.555003	0.7942259
DN6452_c0_g2_i1_2	3.162763	0.7416878
DN6453_c0_g2_i1_2	1.670515	2.321619
DN6457_c0_g1_i1_1	39.51962	35.02932
DN6458_c0_g1_i1_1	0.5782776	2.745342
DN6464_c0_g1_i1_1	0.3236576	0.9544307
DN6469_c0_g1_i1_1	0.310372	1.104121
DN6470_c0_g1_i1_1	2.482682	0.6073284
DN6477_c0_g1_i1_1	10.9187	20.05489
DN6482_c0_g1_i1_1	0.4185605	0.8237802
DN6483_c0_g1_i1_2	1.603763	1.644006
DN6488_c0_g1_i1_2	3.433269	2.49081
DN6491_c0_g1_i2_2	5.557046	1.855715
DN6495_c0_g1_i1_1	0.4614764	0.6687953
DN6502_c0_g1_i1_2	2.003347	2.356708
DN6504_c0_g1_i1_1	1.071182	1.641232
DN6507_c0_g1_i1_1	1.052807	1.149967
DN6515_c0_g1_i1_1	0.5072525	2.803558

DN6515_c0_g2_i1_1	0.9990373	1.379788
DN6522_c0_g1_i1_1	0.1851163	1.994458
DN6522_c0_g2_i1_1	0.148347	1.818184
DN6528_c0_g1_i1_1	2.308385	4.370326
DN6529_c0_g1_i1_2	5.553692	0.9481602
DN6531_c0_g1_i1_1	0.7272059	1.036912
DN6536_c0_g1_i1_1	2.256178	2.157396
DN6538_c0_g1_i1_2	1.601408	0
DN6542_c0_g1_i1_1	1.765382	0.8623931
DN6543_c0_g1_i1_1	0.9188814	0.5766758
DN6556_c0_g1_i1_1	0.8986474	1.878738
DN6557_c0_g1_i1_2	17.1777	3.534298
DN6558_c0_g1_i1_2	3.000156	2.764662
DN6560_c0_g1_i1_1	1.327186	3.118674
DN6563_c0_g1_i2_1	0.4155484	2.315933
DN6565_c0_g1_i1_1	1.469561	3.355149
DN6569_c0_g1_i2_2	48.31407	52.43471
DN6577_c0_g1_i1_1	22.36565	12.46893
DN657_c0_g1_i2_2	7.749486	17.00551
DN6586_c0_g1_i1_1	0.3968932	2.326506
DN6593_c0_g1_i2_1	1.844413	3.258252
DN6597_c0_g1_i2_2	3.199186	0.5356313
DN659_c0_g1_i1_1	1.577669	0.9536179
DN6601_c0_g1_i2_1	2.354841	1.8509

DN6604_c0_g1_i1_1	3.095735	1.449238
DN6607_c0_g1_i3_1	0.8778777	6.409278
DN6611_c0_g1_i1_1	18.87523	11.38641
DN6614_c0_g1_i1_1	2.409948	1.289758
DN6618_c0_g1_i1_1	3.218356	5.929262
DN6619_c0_g1_i1_1	1.060756	2.444681
DN6620_c0_g1_i1_1	0.627993	0.3412007
DN6621_c0_g1_i1_1	12.43851	3.214269
DN6622_c0_g1_i1_1	2.425475	2.58858
DN6627_c0_g1_i1_1	0.2738802	0.5813004
DN6636_c0_g1_i1_1	1.611445	0.8576641
DN6642_c0_g1_i1_1	2.470488	6.208559
DN6648_c0_g1_i1_2	0.1075233	3.065437
DN6649_c0_g1_i1_1	0.3772915	1.094355
DN6649_c0_g1_i1_2	0.382449	0.5193135
DN6657_c0_g1_i1_1	0.2966333	0.5890204
DN6657_c0_g1_i1_2	1.925353	0.2931753
DN6658_c0_g1_i1_1	6.080888	3.90914
DN6659_c0_g1_i1_2	0	0
DN6661_c0_g1_i1_1	3.406891	2.028195
DN6664_c0_g1_i1_2	7.543561	4.144592
DN6665_c0_g1_i1_1	1.094826	1.538544
DN6668_c0_g1_i1_1	1.019019	1.901511
DN6669_c0_g1_i1_2	2.380847	1.020847

DN6673_c1_g1_i1_1	0	0.2003378
DN6674_c0_g1_i1_1	0.4031141	24.00575
DN6681_c0_g1_i1_2	1.911562	1.063686
DN6682_c0_g1_i1_1	1.091871	2.148125
DN6685_c0_g1_i1_2	0.9040988	0.6726254
DN6688_c0_g1_i1_1	0.8441499	13.28664
DN6688_c0_g2_i1_1	0.7750113	6.669897
DN6690_c0_g1_i1_1	2.316984	6.600506
DN6690_c0_g2_i1_1	0.454033	1.423398
DN6692_c0_g1_i1_1	3.841583	3.322274
DN6696_c0_g1_i1_1	0.08683872	1.55296
DN6698_c0_g1_i1_2	4.579109	5.858076
DN66_c0_g1_i1_2	10.83848	19.82175
DN6700_c0_g1_i1_1	1.515091	0.9026284
DN6700_c0_g1_i1_2	5.84029	4.421342
DN6704_c0_g1_i1_1	0.6953329	0.2698615
DN6705_c0_g1_i1_2	2.897607	0.481781
DN6706_c0_g1_i2_1	0.544933	1.597305
DN6708_c0_g1_i2_1	0.1042424	0.4897021
DN670_c0_g1_i1_1	0	1.052778
DN6712_c0_g1_i1_1	1.03669	1.42901
DN6714_c0_g1_i1_1	2.165164	3.681764
DN6714_c0_g1_i1_2	1.738246	0.1680815
DN671_c0_g1_i2_1	5.293308	2.968871

DN6728_c0_g1_i1_1	0.5303545	0.9440845
DN6733_c0_g1_i2_1	0.8799435	3.93746
DN6733_c0_g1_i2_2	1.088995	3.155856
DN6734_c0_g1_i1_1	0.08510213	0.8253072
DN6735_c0_g1_i1_1	0	0
DN6738_c0_g1_i1_1	0.3310096	1.878929
DN6739_c0_g1_i2_1	9.873309	20.94239
DN6741_c0_g1_i1_1	1.318048	0.9924208
DN6751_c0_g1_i1_2	0	0
DN6752_c0_g1_i1_2	28.02345	6.501911
DN6761_c0_g1_i1_1	1.499863	1.395093
DN6762_c0_g2_i1_1	1.243121	1.292973
DN6764_c0_g1_i1_1	2.350952	1.874642
DN6773_c0_g1_i1_1	0.8925598	0.242944
DN6774_c0_g1_i1_2	2.60164	0.8560339
DN6780_c0_g1_i2_1	1.183634	2.694038
DN6782_c0_g1_i1_1	1.996733	1.260107
DN6783_c0_g1_i1_2	1.759757	1.630641
DN6795_c0_g1_i1_1	1.13829	3.064056
DN6797_c0_g1_i1_1	2.190427	2.452011
DN67_c0_g1_i1_2	1.259379	0.3379961
DN6800_c0_g1_i1_1	2.669358	3.994986
DN6801_c0_g1_i1_2	0.5746273	1.506588
DN6803_c0_g2_i1_1	0.1660564	1.811382

DN6804_c0_g1_i1_1	3.083688	3.173414
DN6806_c0_g1_i1_2	2.639529	2.390479
DN6807_c0_g1_i1_1	8.051505	9.015513
DN680_c0_g1_i1_1	0.2579392	0.1434236
DN6825_c0_g1_i1_1	5.307289	6.204255
DN6827_c0_g1_i1_1	1.039366	0.7835448
DN6827_c0_g2_i1_1	2.765126	2.61345
DN6829_c0_g1_i1_1	0.6546642	0.8228779
DN6830_c0_g1_i1_1	0.7083837	0.2963202
DN6833_c0_g1_i1_1	0.84936	2.007775
DN6835_c0_g1_i1_1	4.414998	4.014385
DN6837_c0_g1_i1_1	2.012149	1.152
DN6838_c0_g1_i1_1	2.30519	2.095022
DN6840_c0_g1_i1_1	2.855644	2.252237
DN6843_c0_g1_i1_1	0.8713825	1.439223
DN6844_c0_g1_i1_1	0.7552057	2.334197
DN6845_c0_g1_i1_2	2.509031	1.017248
DN684_c0_g1_i2_2	2.481091	0.5187155
DN6851_c0_g1_i1_2	1.224439	0
DN6854_c0_g1_i2_1	9.621691	24.98741
DN6856_c0_g1_i1_2	1.485015	2.020148
DN6861_c0_g1_i1_2	1.188635	0.144079
DN6863_c0_g1_i1_1	0.6208705	0.3505995
DN6876_c0_g1_i1_1	0.390233	0.6569243

DN6878_c0_g1_i1_1	0.377968	0.9646575
DN687_c0_g1_i1_1	0.4081082	0
DN6882_c0_g1_i1_2	50.07257	113.7869
DN6885_c0_g1_i1_1	12.93774	11.39851
DN6889_c0_g1_i2_1	12.44664	7.464137
DN688_c0_g1_i1_2	5.53302	1.656432
DN6898_c0_g1_i1_1	0.2684838	0.1281142
DN6901_c0_g1_i1_2	3.508898	2.099598
DN6905_c0_g1_i1_2	3.455702	3.679151
DN6906_c0_g1_i1_2	1.897011	0.5066175
DN6907_c0_g1_i1_1	0	5.007295
DN6910_c0_g1_i1_1	4.130288	2.062495
DN6911_c0_g1_i1_2	2.028186	0.6251342
DN6913_c0_g1_i1_1	3.488629	1.394627
DN6922_c0_g1_i1_1	1.053915	4.70433
DN6927_c0_g1_i1_1	62.80222	84.60449
DN693_c0_g1_i1_1	0.636182	0.6273552
DN6946_c0_g1_i1_1	2.581172	2.733056
DN6946_c0_g2_i1_1	0	0.1954494
DN6953_c0_g1_i1_1	0.7937765	2.282742
DN6954_c0_g2_i2_1	2.794388	1.856535
DN6955_c0_g1_i1_1	0	0.9605082
DN6958_c0_g1_i1_1	3.27798	0.2969887
DN6959_c0_g1_i1_2	26.62397	2.456768

DN6974_c0_g1_i1_2	2.511296	1.045051
DN6975_c0_g1_i1_1	2.103492	0.7491463
DN6975_c0_g2_i1_1	0.3862704	0.2235072
DN6978_c0_g1_i1_1	0.1615229	1.494803
DN6980_c0_g3_i1_1	1.25203	1.472443
DN6983_c0_g1_i1_1	4.061886	4.237898
DN6983_c0_g1_i1_2	1.681506	1.317531
DN6998_c0_g1_i1_2	2.644307	1.488142
DN69_c0_g2_i1_1	0.1293945	3.05651
DN7001_c0_g1_i1_2	1.376762	0.4341985
DN7001_c0_g2_i1_2	2.115611	0.9152286
DN7007_c0_g1_i1_2	2.250825	1.44143
DN7012_c0_g1_i1_1	2.643134	2.003617
DN7013_c0_g1_i1_1	0.350827	9.981978
DN7013_c0_g1_i1_2	0.6630121	0.5620548
DN7017_c0_g1_i1_1	0.5531794	5.138811
DN7021_c0_g1_i1_2	0.6256172	0.7544661
DN7030_c0_g1_i1_1	0.7949123	0.2415636
DN7030_c0_g1_i1_2	12.83606	16.52779
DN7040_c0_g2_i1_2	2.139794	1.651648
DN7044_c0_g1_i1_2	3.543472	3.035807
DN7050_c0_g1_i1_1	18.28895	9.377022
DN7051_c0_g1_i1_1	0.525751	1.692437
DN7051_c0_g2_i1_1	0.7317124	1.184623

DN705_c0_g2_i1_1	0.2344281	0.5083783
DN7060_c0_g1_i1_1	0.5893204	2.020651
DN7061_c0_g1_i1_2	1.07047	0.3818783
DN7073_c0_g1_i1_1	106.9891	121.2208
DN7073_c0_g1_i1_2	4.30338	0.7413322
DN7074_c0_g1_i1_1	0.6806533	1.083087
DN7081_c0_g1_i1_2	0	0
DN7081_c0_g1_i3_1	2.576924	3.465394
DN7088_c0_g1_i2_2	20.20899	4.809313
DN7092_c0_g1_i1_1	0.8398577	1.789695
DN7094_c0_g1_i1_2	1.916853	0.2968767
DN7095_c0_g1_i2_2	1.6662	0.7621907
DN7099_c0_g1_i1_1	2.2385	3.72653
DN7100_c0_g1_i1_2	0.9180837	0.2103225
DN7102_c0_g1_i1_2	1.742297	0.1092918
DN7105_c0_g1_i1_1	0.684344	1.79915
DN7108_c0_g1_i1_2	2.172661	0.880245
DN7110_c0_g1_i1_2	4.888981	1.087634
DN7115_c0_g2_i1_1	1.154442	5.867399
DN7116_c0_g1_i1_1	0.1405145	0.9391307
DN7117_c0_g1_i1_2	1.503314	0.7209954
DN7119_c0_g1_i2_1	3.931322	12.63145
DN7128_c0_g1_i1_1	1.202153	2.819033
DN7130_c0_g1_i1_2	2.061006	0.2511696

DN7131_c0_g1_i1_1	1.758722	1.770527
DN7132_c0_g1_i1_2	0	0
DN7133_c0_g1_i1_2	1.98652	2.270736
DN7135_c0_g1_i1_2	0	0
DN7137_c0_g1_i1_1	0.9365431	1.132268
DN7138_c0_g1_i1_2	1.340843	1.875659
DN713_c0_g1_i1_2	3.921203	0.4990269
DN7141_c0_g1_i1_1	0.837498	0.7779838
DN7145_c0_g1_i1_2	0.59428	0.6856366
DN7149_c0_g1_i1_2	1.227429	0
DN7156_c0_g1_i1_1	0.8083586	0.6381592
DN7167_c0_g2_i1_1	1.868503	1.141678
DN7168_c0_g1_i1_1	1.528745	5.651568
DN7172_c0_g1_i1_1	5.36E-49	9.82E-42
DN7178_c0_g1_i1_1	0	0
DN7178_c0_g2_i1_1	1.151128	2.253349
DN7179_c0_g1_i1_1	1.166321	1.666943
DN7184_c0_g1_i1_1	0.9446803	2.033379
DN7194_c0_g1_i1_1	0	0.9036999
DN7202_c0_g1_i1_1	1.775066	0.6393056
DN7206_c0_g1_i1_2	4.713135	1.38564
DN7209_c0_g1_i1_1	4.186125	3.06182
DN7211_c0_g1_i1_2	0.5494987	3.53E-07
DN7212_c0_g1_i1_1	2.304207	4.122534

DN7224_c0_g1_i1_2	1.765562	2.40237
DN7227_c0_g1_i1_1	6.102901	27.44777
DN7229_c0_g1_i1_2	0.9584129	0.724621
DN722_c0_g1_i2_1	9.270151	4.154428
DN7231_c0_g1_i1_1	0.2714951	0.2702262
DN7235_c0_g1_i1_1	0.1830088	0.5095097
DN7239_c0_g1_i1_1	0.3565593	2.190936
DN723_c0_g1_i1_1	0.2563391	0.3365252
DN723_c0_g1_i1_2	3.824895	3.909535
DN7241_c0_g1_i1_1	0.801464	1.349823
DN7244_c0_g1_i1_1	0.9459262	0.3350781
DN7246_c0_g1_i1_1	0.4271579	1.429497
DN7247_c0_g1_i1_2	0.5542913	1.611624
DN7248_c0_g1_i1_1	1.410347	1.243179
DN7258_c0_g1_i1_1	0.4106617	0
DN7260_c0_g1_i1_1	1.273349	2.939066
DN7264_c0_g1_i1_1	1.546882	0.9468909
DN7265_c0_g1_i1_1	0	0.8360065
DN7269_c0_g1_i1_1	1.647189	3.244208
DN7270_c0_g1_i1_1	4.4966	5.321535
DN7273_c0_g1_i1_1	0	0
DN7278_c0_g1_i1_1	0.1321643	2.672587
DN727_c0_g2_i1_1	0.7093902	2.159863
DN7280_c0_g1_i1_1	1.733384	1.696467

DN7281_c0_g1_i1_2	1.18041	1.103219
DN7281_c1_g1_i1_2	3.967844	2.362861
DN7284_c0_g1_i1_1	57.78467	39.85943
DN728_c0_g1_i1_2	0	0
DN7291_c0_g1_i1_1	2.186381	0.7658231
DN7293_c0_g1_i1_1	1.35256	0.7519389
DN7298_c0_g1_i1_1	0.4085485	0.541255
DN7299_c0_g1_i1_2	5.632555	11.03018
DN7308_c0_g1_i2_1	4.299431	1.282183
DN7309_c0_g1_i1_1	0.7207067	1.553174
DN7310_c0_g1_i1_1	0	0
DN7317_c0_g1_i2_1	2.164412	3.605629
DN7318_c0_g1_i1_1	1.012583	0.7323161
DN731_c0_g1_i2_1	4.012146	2.830981
DN7321_c0_g1_i1_1	2.367246	0.8860831
DN7322_c0_g1_i1_1	1.738915	2.044102
DN7324_c0_g1_i1_1	27.16597	103.0307
DN7328_c0_g1_i1_1	1.876	0.8624483
DN7333_c0_g1_i1_1	1.624255	4.696639
DN7333_c0_g1_i2_2	1.835364	0.5482304
DN7333_c0_g2_i1_1	0.41221	1.522968
DN7340_c0_g1_i1_1	1.20359	1.672658
DN7341_c0_g1_i1_1	5.620388	2.287552
DN7343_c0_g1_i1_1	1.017194	1.748921

DN7346_c0_g1_i1_1	0	1.281778
DN7347_c0_g1_i1_1	0.935139	1.334525
DN734_c0_g1_i1_1	2.37902	1.846613
DN7350_c0_g1_i1_1	0.1180091	0.5424274
DN7351_c0_g1_i1_1	1.108918	5.590421
DN7354_c0_g2_i1_1	0.8115431	0.7137686
DN7355_c0_g1_i1_2	1.010894	2.0568
DN7356_c0_g1_i1_1	0.5882973	0.8764751
DN735_c0_g1_i1_1	0.8019675	0.674151
DN7361_c0_g1_i1_1	0.779151	1.545415
DN7364_c0_g1_i1_2	42.68815	727.6081
DN7365_c0_g1_i1_2	1.970098	0.3798331
DN7367_c0_g1_i1_1	0	3.854851
DN7373_c0_g1_i2_2	3.335315	1.425322
DN7376_c0_g1_i1_2	1.054926	0.06615869
DN7380_c0_g1_i1_1	0.7044201	1.512576
DN7386_c0_g1_i1_1	0.7919402	0.2873869
DN738_c0_g1_i1_1	1.23352	3.870437
DN738_c0_g1_i1_2	1.345358	0.3486336
DN7390_c0_g1_i1_2	18.88072	20.52026
DN7392_c0_g1_i1_1	9.110286	7.82209
DN7392_c0_g1_i1_2	3.11339	1.581061
DN7399_c0_g1_i1_1	1.998291	0.6825693
DN73_c0_g1_i1_2	15.23968	7.63552

DN7400_c0_g1_i1_1	2.441565	2.014285
DN7403_c0_g1_i2_1	1.079439	1.006871
DN7406_c0_g1_i1_1	0.7389628	1.893619
DN7408_c0_g1_i2_1	1.241851	6.08297
DN7417_c0_g1_i1_1	4.480725	6.859926
DN7419_c0_g1_i1_2	2.639347	1.142057
DN741_c0_g1_i1_1	3.058828	2.936053
DN741_c0_g1_i1_2	3.227781	1.825487
DN7423_c0_g2_i1_1	0.6968109	1.584705
DN7425_c0_g1_i1_1	5.6221	4.302773
DN7426_c0_g1_i1_1	1.021927	0.3952336
DN7434_c0_g1_i1_1	0.3013081	1.085388
DN7434_c0_g1_i1_2	1.317511	0.576557
DN7441_c0_g1_i1_1	0.4840493	0.7274411
DN7444_c0_g1_i2_1	0.3623662	1.356351
DN7445_c0_g1_i1_1	2.512846	6.39978
DN7446_c0_g1_i1_1	2.781069	6.967513
DN7446_c0_g1_i1_2	0	0
DN744_c0_g1_i1_1	2.136256	2.645577
DN7451_c0_g1_i1_2	1.572917	0.5941829
DN7455_c0_g1_i1_1	6.118041	3.031205
DN7456_c0_g2_i1_1	0.2501	1.911143
DN7457_c0_g2_i1_1	1.214173	1.947748
DN7457_c0_g3_i1_1	0.4643558	1.343726

DN7460_c0_g1_i1_2	0	0
DN7465_c0_g2_i1_1	4.770866	6.902514
DN7470_c0_g1_i1_2	3.94502	3.370186
DN7473_c0_g1_i1_1	0.4111311	1.598762
DN7480_c0_g1_i1_2	0	0
DN7481_c0_g1_i1_2	1.588068	0.405411
DN7484_c0_g1_i1_2	1.581597	0.4342443
DN7489_c0_g1_i1_1	0.03651861	2.471187
DN7497_c0_g1_i1_2	16.47461	38.57999
DN7502_c0_g1_i1_1	0.1459758	0
DN7504_c0_g1_i1_1	23.23017	17.37388
DN7504_c0_g1_i1_2	5.651172	14.42455
DN7505_c0_g1_i2_1	1.079871	2.083653
DN7506_c0_g1_i2_1	6.591389	3.795022
DN7509_c0_g1_i1_2	3.11412	0.4033197
DN7510_c0_g2_i1_1	0	0.414233
DN7514_c0_g1_i1_1	0.7478664	2.298718
DN7516_c0_g1_i1_1	0.2804402	0.3144333
DN7526_c0_g1_i2_2	6.868509	9.058101
DN7527_c0_g1_i1_1	0.5755127	2.958479
DN7528_c0_g1_i1_2	6.316508	13.96956
DN7530_c0_g1_i1_1	1.14812	0.748969
DN7532_c0_g1_i1_2	0	0
DN7534_c0_g1_i1_1	1.213902	2.003437

DN7537_c0_g1_i1_1	3.158163	3.276614
DN7541_c0_g2_i1_2	2.209125	1.25766
DN7542_c0_g1_i1_1	1.466125	1.91021
DN7543_c0_g1_i1_1	0.1526	0.9469579
DN7543_c0_g1_i1_2	0.5030971	0.4547437
DN7545_c0_g1_i1_1	1.618822	1.619932
DN7545_c0_g1_i1_2	2.193063	0.732894
DN7546_c0_g1_i1_1	0.6123413	0.9714565
DN7548_c0_g1_i2_2	3.092487	0.5759797
DN7552_c0_g1_i1_1	1.086619	1.769804
DN7552_c0_g2_i1_1	2.001549	3.726638
DN7559_c0_g1_i1_1	1.139139	0.771227
DN755_c0_g1_i1_2	0.6224827	0.6278288
DN755_c0_g2_i1_2	0.5941821	0.5269499
DN7561_c0_g1_i1_1	0	0.5204931
DN7563_c0_g1_i1_1	1.045374	0.8094629
DN7565_c0_g1_i1_2	1.067786	0.2591336
DN7566_c0_g1_i1_1	0.9136446	1.010136
DN7567_c0_g1_i1_1	27.63534	9.488539
DN7572_c0_g1_i1_1	2.85E-30	1.763577
DN7572_c0_g1_i1_2	1.797768	1.51E-06
DN7572_c0_g2_i1_2	3.531748	3.270036
DN7574_c0_g1_i1_1	0.3530463	2.201603
DN7574_c0_g2_i1_1	1.776454	3.746306

DN7584_c0_g1_i1_2	5.272435	4.053612
DN7585_c0_g1_i1_1	1.596947	6.796641
DN7586_c0_g1_i1_1	1.898616	2.287669
DN7586_c0_g1_i1_2	2.270647	1.275922
DN7591_c0_g1_i1_1	0.6110365	1.747792
DN7594_c0_g1_i1_1	0.9210558	1.153676
DN7598_c0_g2_i1_1	231.794	653.9956
DN7600_c0_g2_i1_1	0.5382509	1.132943
DN7601_c0_g1_i1_1	0.672353	1.151846
DN7606_c0_g1_i1_1	1.451816	2.212004
DN7609_c0_g1_i1_2	1.076656	2.780317
DN7611_c0_g1_i2_1	0.6962999	0.5773133
DN7612_c0_g1_i1_1	1.73572	3.793261
DN7613_c0_g1_i1_1	9.571304	10.37397
DN7615_c0_g1_i1_1	1.154905	1.210434
DN761_c0_g1_i1_2	5.08819	0.2984089
DN7620_c0_g1_i1_1	0.8384327	0.4224377
DN7624_c0_g1_i2_1	4.107153	3.601132
DN7630_c0_g1_i2_1	3.712528	2.550851
DN7640_c0_g1_i1_1	4.057963	1.638929
DN7640_c0_g1_i1_2	2.326966	0.7106564
DN7644_c0_g1_i2_1	7.208423	2.761475
DN7645_c0_g1_i1_1	2.028885	1.967718
DN7648_c0_g1_i2_1	0.9153925	1.590781

DN7652_c0_g1_i1_1	2.111553	1.278296
DN7653_c0_g1_i1_1	0.961756	0.8127835
DN7658_c0_g1_i1_1	0.738345	0.7238518
DN765_c0_g1_i1_1	0	0.3208498
DN7661_c0_g1_i1_1	3.29686	4.111663
DN7662_c0_g1_i1_1	4.993733	3.000721
DN7665_c0_g1_i1_1	0.1424085	1.82725
DN7668_c0_g1_i1_1	0.8431233	2.443502
DN7668_c0_g2_i1_1	0.1387561	2.502723
DN7669_c0_g1_i1_1	0.7522743	1.766599
DN7673_c0_g2_i1_1	0	6.072592
DN7675_c0_g1_i1_1	2.140077	1.780159
DN7684_c0_g1_i1_1	4.65775	3.242263
DN7684_c0_g1_i1_2	2.822966	2.033145
DN7685_c0_g1_i1_2	1.784354	1.397326
DN7686_c0_g1_i1_2	0.465156	0.2948137
DN7687_c0_g1_i1_1	0.6072907	5.146431
DN7688_c0_g1_i1_1	3.50E-26	1.994603
DN7693_c0_g1_i1_1	0.7296989	5.278465
DN7694_c0_g1_i1_1	1.687243	2.699222
DN769_c0_g1_i1_2	1.490306	1.069146
DN7702_c0_g1_i1_1	0.4967112	0.7393439
DN7707_c0_g1_i1_2	3.399262	0.2118056
DN7715_c0_g1_i1_1	0.2655205	0.5969508

DN7717_c0_g1_i1_1	0.9599452	2.281751
DN7718_c0_g1_i1_2	56.08476	35.82393
DN7721_c0_g1_i1_1	0.09887409	1.041084
DN7722_c0_g1_i1_1	0.8567195	0.9630995
DN7726_c0_g1_i3_1	11.12891	12.23589
DN7727_c0_g1_i1_1	1.273734	1.808093
DN7728_c0_g1_i1_2	0.5650093	0.3145251
DN7735_c0_g1_i1_2	9.769863	8.795622
DN7738_c0_g2_i1_1	0	0
DN7740_c0_g1_i1_1	38.0771	12.23774
DN7741_c0_g1_i1_1	0.2472807	1.826213
DN7741_c0_g1_i1_2	0.9948191	0.5121598
DN7742_c0_g1_i1_2	2.172035	0.7431877
DN7750_c0_g1_i1_1	2.574453	2.568952
DN7751_c0_g1_i2_1	5.588686	46.25645
DN7752_c0_g1_i1_2	0.7156149	1.037251
DN7756_c0_g1_i1_1	0.09918217	0
DN7761_c0_g1_i1_1	0.4327726	1.105219
DN7763_c0_g1_i1_1	1.244834	2.625363
DN7771_c0_g1_i1_1	146.4879	179.5498
DN7774_c0_g1_i2_1	4.236944	1.796155
DN7778_c0_g1_i2_1	3949.055	2001.324
DN7785_c0_g1_i1_1	2.353712	1.251218
DN7792_c0_g1_i1_1	0	2.046789

DN7792_c0_g1_i1_2	3.73834	2.105226
DN7794_c0_g1_i1_1	0.1723117	1.890477
DN7795_c0_g1_i1_1	0.2087722	0.9276247
DN7798_c0_g1_i2_1	2.252268	2.04924
DN77_c0_g1_i1_1	0.4664908	0.2442837
DN7801_c0_g1_i1_1	0.6246724	1.798635
DN7807_c0_g1_i1_1	0.4336706	0.9958943
DN7807_c0_g2_i1_1	1.19752	0.7716015
DN7810_c0_g1_i1_2	4.603949	5.034926
DN7811_c0_g1_i1_1	2.210388	3.981482
DN7812_c0_g1_i1_1	0.9701456	3.158375
DN7814_c0_g2_i1_2	2.119399	1.700158
DN7815_c0_g1_i1_1	12.86838	6.023866
DN7821_c0_g2_i1_1	1.362163	1.647633
DN7822_c0_g1_i1_1	2.522879	4.523836
DN7823_c0_g1_i1_1	1.360566	0.6514228
DN7828_c0_g1_i1_1	0.8955541	0.5444867
DN7829_c0_g1_i1_1	0.2620916	0.7343879
DN7832_c0_g1_i1_1	2.042037	3.933359
DN7833_c0_g1_i1_2	0.7408332	1.987446
DN7835_c0_g1_i1_2	2.254252	2.06851
DN7839_c0_g1_i1_1	0.3596595	1.674254
DN7840_c1_g1_i1_1	0	1.653915
DN7842_c0_g1_i1_1	1.518331	3.515036

DN7843_c0_g1_i1_1	0.9413738	0.8879835
DN7844_c0_g2_i1_1	3.657597	0.5339075
DN7845_c0_g1_i1_1	1.24873	0.5993622
DN7845_c0_g1_i1_2	2.577852	1.00995
DN7846_c0_g1_i1_1	0.6810556	0.6023611
DN784_c0_g1_i1_1	2.425367	2.324938
DN7855_c0_g1_i1_1	0.1718809	3.530446
DN7857_c0_g1_i1_2	0	0
DN7859_c0_g1_i1_1	1.432318	1.432886
DN7860_c0_g1_i1_2	10.72543	4.057755
DN7861_c0_g1_i1_1	0.6579425	1.330305
DN7869_c0_g1_i1_1	5.351438	2.685776
DN786_c0_g1_i1_1	0.9105752	1.452976
DN7871_c0_g1_i2_1	1.901494	3.898754
DN7874_c0_g1_i1_1	0.8038904	0.6364159
DN7876_c0_g1_i1_1	0.4862416	1.338682
DN7878_c0_g1_i1_2	4.589636	0.7901835
DN7879_c0_g1_i1_1	0.0113684	1.184036
DN7883_c0_g1_i1_1	0.08603303	2.218842
DN7884_c0_g1_i1_1	1.919006	2.966017
DN7884_c0_g1_i1_2	6.616773	2.606184
DN7889_c0_g1_i1_2	1.673993	0.6130339
DN7891_c0_g1_i1_1	0.8338064	3.065749
DN789_c0_g1_i1_1	2.702464	0.9581691

DN7903_c0_g1_i1_1	0.5844868	3.838424
DN7903_c0_g1_i1_2	1.580194	0
DN7904_c0_g2_i1_2	1.635566	0.5096959
DN7905_c0_g1_i1_1	2.972279	3.821983
DN7906_c0_g1_i1_2	2.763246	3.126013
DN790_c0_g1_i1_1	7.439173	6.157512
DN7917_c0_g1_i1_1	7.560021	7.665993
DN7919_c0_g1_i1_1	4.78E-20	0.378617
DN7920_c0_g1_i1_1	0.4675127	0.5481433
DN7920_c0_g1_i2_2	3.143328	1.378
DN7921_c0_g1_i1_1	0.3035909	1.106002
DN7924_c0_g1_i1_1	0.6521354	0.1686961
DN7924_c0_g1_i1_2	1.413179	0.6906253
DN7925_c0_g1_i1_2	2.255009	1.770474
DN7937_c0_g1_i1_2	0.9793777	1.475855
DN7940_c0_g1_i1_1	1.22271	2.817526
DN7942_c0_g1_i1_2	0.9203181	0.4660643
DN7946_c0_g1_i1_1	0	1.288708
DN7946_c0_g2_i1_1	0.5527323	0.795247
DN7948_c0_g1_i1_1	3.233745	11.86169
DN7949_c0_g1_i1_1	0.6610319	1.553702
DN794_c0_g1_i1_1	0.8503396	0.7774458
DN7951_c0_g1_i1_2	15.83899	9.178587
DN7952_c0_g1_i1_1	0.1305159	0.3352552

DN7952_c1_g1_i1_1	0	0.8206401
DN7953_c0_g1_i1_1	0.1274694	0.5331854
DN7955_c0_g1_i1_2	0	0
DN795_c0_g1_i1_1	0.584946	6.797393
DN795_c0_g1_i1_2	4.753091	3.931655
DN7960_c0_g1_i1_2	5.968426	4.466684
DN7961_c0_g1_i1_1	1.47395	1.799376
DN7962_c0_g1_i1_1	3.819901	3.735093
DN7964_c0_g1_i1_2	6.284896	0.3104566
DN7974_c0_g1_i1_1	0.7553998	3.21658
DN7975_c0_g1_i2_1	7.56644	7.229485
DN7976_c0_g1_i1_2	0.4485266	0
DN7980_c0_g1_i1_1	0.7153425	2.032696
DN7987_c0_g1_i1_1	0.2785895	0.5165129
DN7988_c0_g2_i1_1	8.20096	5.030831
DN7989_c0_g1_i1_1	0.2805097	1.889467
DN7996_c0_g1_i1_2	6.304498	5.878508
DN7999_c0_g1_i1_1	0.5389124	0.3229037
DN7_c0_g1_i1_1	9.409004	12.5049
DN8001_c0_g1_i1_2	5.165386	5.57887
DN8002_c0_g1_i1_1	3.345214	5.388583
DN8013_c0_g1_i1_2	2.364051	0.3418195
DN8013_c0_g2_i1_2	1.617545	0.136892
DN8025_c0_g1_i1_1	1.117495	1.30947

DN8027_c0_g1_i2_1	3.750641	3.320704
DN8028_c0_g1_i1_1	1.30829	1.674052
DN8033_c0_g1_i1_1	0.2850967	0.5262067
DN8042_c0_g1_i1_2	1.047663	0.9910712
DN8057_c0_g1_i1_2	0.5088891	0.7657571
DN8060_c0_g1_i1_1	0.3202988	7.947622
DN8061_c0_g1_i1_1	5.129673	2.683158
DN8065_c0_g1_i1_1	0.5354178	1.096445
DN8066_c0_g1_i1_2	2.211075	1.430356
DN8077_c0_g1_i1_2	1.237366	0.9241778
DN8078_c0_g1_i1_1	1.193079	1.757447
DN8079_c0_g2_i1_1	1.34722	2.344956
DN8083_c0_g1_i1_2	99.97051	78.94477
DN8087_c0_g1_i1_2	2.096545	0
DN8088_c0_g1_i1_1	1.446616	1.313492
DN8091_c0_g1_i1_1	0.9532637	0.7110327
DN8094_c0_g1_i1_1	2.301191	1.900543
DN8096_c0_g1_i1_1	0.5106961	3.232514
DN8097_c0_g2_i1_1	5.132415	4.052997
DN8099_c0_g1_i2_1	0.9324603	0.481121
DN809_c0_g1_i1_1	0.6921919	4.741014
DN80_c0_g1_i1_2	18.36306	12.17859
DN8101_c0_g1_i1_1	3.331413	5.856996
DN8101_c0_g1_i1_2	2.468597	1.707961

DN8102_c0_g1_i1_1	0.8649959	1.485364
DN8108_c0_g1_i1_2	5.56156	4.034239
DN8111_c0_g1_i1_2	2.088404	0.4834985
DN8113_c0_g1_i1_1	1.245594	1.732574
DN8115_c0_g2_i1_1	1.177091	6.029997
DN8125_c0_g1_i1_1	0.9492731	0.1394272
DN8136_c0_g1_i1_2	77.08	0.1073363
DN8138_c0_g1_i1_1	1.827105	1.178861
DN8139_c0_g1_i1_1	0.9953368	3.085291
DN8139_c0_g1_i1_2	1.503294	0.8905378
DN8141_c0_g1_i1_1	0.1812217	1.353328
DN8143_c0_g1_i1_2	1.823956	1.409674
DN8149_c0_g1_i1_1	0.4192967	1.002994
DN8151_c0_g1_i1_1	1.310418	1.656517
DN8152_c0_g1_i1_1	1.796542	0.9280123
DN8154_c0_g1_i1_1	61.31026	36.51298
DN8156_c0_g1_i1_2	74.50587	73.42179
DN8157_c0_g1_i1_1	1.051054	1.005317
DN8157_c0_g1_i1_2	0	0
DN8161_c0_g1_i1_2	1.557104	0.2151355
DN8162_c0_g1_i1_1	2.866959	0.7360336
DN8163_c0_g1_i1_1	0	0.4514992
DN8163_c0_g1_i1_2	0.4183301	0
DN8164_c0_g1_i1_2	1.141124	1.575535

DN8165_c0_g1_i1_1	0.7049509	0.9164741
DN8165_c0_g1_i1_2	2.281536	3.240245
DN8178_c0_g1_i1_1	0.8037774	0.6049357
DN8180_c0_g1_i1_2	1.011885	1.921393
DN8183_c0_g1_i1_1	180.184	79.36752
DN8192_c0_g1_i1_2	2.379115	1.062839
DN8194_c0_g1_i1_1	0.1945128	4.057318
DN8198_c0_g1_i1_1	0.5476704	1.174542
DN819_c0_g1_i1_1	1.230124	0.2881525
DN8207_c0_g1_i1_1	0.4278166	2.969992
DN8207_c0_g1_i1_2	1.81818	2.724423
DN8208_c0_g1_i1_2	2.302653	1.406766
DN8208_c0_g2_i1_2	3.50304	0
DN8209_c0_g1_i1_1	0.6997643	1.325375
DN8212_c0_g2_i1_1	1.418979	2.109388
DN8214_c0_g1_i1_1	1.154663	1.468583
DN8217_c0_g1_i1_1	5.081593	1.409531
DN8218_c0_g1_i1_1	5.157054	5.722117
DN8220_c0_g1_i1_1	0.4014584	0.4230153
DN8220_c0_g2_i1_1	0.9311147	2.819785
DN8222_c0_g1_i1_1	0.7936291	1.384129
DN8228_c0_g1_i1_1	1.011412	1.642786
DN8233_c0_g1_i1_1	1.280582	1.100827
DN8233_c0_g2_i1_1	0.8521402	0.9953308

DN8239_c0_g1_i1_1	3.78465	2.559491
DN8240_c0_g1_i2_1	5.027162	4.063667
DN824_c0_g2_i1_2	1.608646	0.9955141

Table S2. The top 30 co-expressed mRNAs with lncRNA DN20924.

**Num**	**gene_id**	**up_down**	**KEGG gene name**	**KEGG description**	**Pathway**	**Pathway definition**
1	DN35171_c0_g1_i1_2	Up	ftnA	ferritin	--	
2	DN2619_c0_g1_i1_2	Up	--	--	--	
3	DN17109_c0_g1_i1_2	Up	--	--	--	
4	DN17669_c0_g1_i1_1	Up	--	--	--	
5	DN24588_c0_g1_i1_2	Up	--	--	--	
6	DN13510_c0_g1_i1_2	Up	--	--	--	
7	DN31047_c0_g1_i1_2	Up	--	--	--	
8	DN3215_c0_g1_i1_2	Up	--	--	--	
9	DN19614_c0_g1_i1_1	Up	AGL	glycogen debranching enzyme	ko00500 Starch and sucrose metabolism	

10	DN24231_c0_g1_i1_2	Up	--	--	--	
11	DN26529_c0_g1_i1_2	Up	--	--	--	
12	DN20179_c0_g1_i1_2	Up	--	--	--	
13	DN15482_c0_g2_i1_2	Up	--	--	--	
14	DN34991_c0_g1_i1_2	Up	OSBPL5_8	oxysterol-binding protein-related protein 5	--	
15	DN33_c0_g1_i2_2	Up	ZIP9	zinc transporter member 9	--	
16	DN18710_c0_g2_i1_2	Up	--	--	--	
17	DN34458_c0_g1_i1_2	Up	--	--	--	
18	DN13932_c0_g1_i1_2	Up	--	--	--	
19	DN15180_c0_g1_i1_2	Up	--	--	--	
20	DN33774_c0_g1_i1_2	Up	TOP3	DNA topoisomerase III	ko03460|ko03440	Fanconi anemia pathway|Homologous recombination
21	DN28201_c0_g1_i1_1	Up	VAMP7	vesicle-associated membrane protein 7	ko04130	SNARE interactions in vesicular transport
22	DN14721_c	Up	EIF1	translation	ko03013	RNA transport

	0_g1_i1_2			initiation factor 1		
23	DN19473_c0_g1_i1_2	Up	--	--	--	
24	DN33352_c0_g1_i1_2	Up	UBE1	ubiquitin-activating enzyme E1	ko04120	Ubiquitin mediated proteolysis
25	DN2065_c0_g1_i1_2	Up	--	--	--	
26	DN5114_c0_g1_i1_2	Up	--	--	--	
27	DN32209_c0_g1_i1_2	Up	--	--	--	
28	DN15718_c0_g1_i1_2	Up	--	--	--	
29	DN22063_c0_g1_i1_2	Up	--	--	--	
30	DN16611_c0_g1_i1_2	Up	--	--	--	

Table S3. The top 30 co-expressed mRNAs with lncRNA DN30855.

**Num**	**gene_id**	**up_down**	**KEGG gene name**	**KEGG description**	**Pathway**	**Pathway definition**
1	DN19281_c0_g3_i1_2	Down	--	--	--	
2	DN24531_c0_g1_i1_1	Down	--	--	--	
3	DN11199_c0_g1_i1_2	Down	--	--	--	

4	DN21874_c0_g1_i1_1	Down	--	--	--	
5	DN6873_c0_g1_i4_1	Down	--	--	--	
6	DN499_c0_g1_i2_1	Down	--	--	--	
7	DN24655_c0_g1_i1_1	Down	--	--	--	
8	DN10211_c0_g1_i1_1	Down	GGH	gamma-glutamyl hydrolase	ko00790	Folate biosynthesis
9	DN25994_c0_g1_i1_1	Down	--	--	--	
10	DN18848_c0_g1_i2_2	Down	--	--	--	
11	DN26916_c0_g1_i1_1	Down	--	--	--	
12	DN6918_c0_g1_i3_1	Down	TTLL11	tubulin polyglutamylase TTLL11	--	
13	DN17208_c1_g4_i2_1	Down	groEL	chaperonin GroEL	ko03018	RNA degradation
14	DN28190_c0_g1_i1_1	Down	--	--	--	
15	DN24233_c0_g1_i1_1	Down	--	--	--	
16	DN13853_c0_g1_i1_1	Down	--	--	--	
17	DN10211_c0_g2_i1_1	Down	GGH	gamma-glutamyl hydrolase	ko00790	Folate biosynthesis

18	DN24134_c0_g1_i1_1	Down	--	--	--	
19	DN16539_c0_g1_i1_1	Down	--	--	--	
20	DN16623_c0_g1_i2_1	Down	--	--	--	
21	DN30341_c0_g1_i1_1	Down	--	--	--	
22	DN12284_c0_g1_i3_1	Down	CAMK	calcium/calmodulin-dependent protein kinase	ko04925	Aldosterone synthesis and secretion
23	DN28149_c0_g1_i1_1	Down	MELK	maternal embryonic leucine zipper kinase	--	
24	DN26429_c0_g1_i1_1	Down	amyA	alpha-amylase	ko04973|ko00500	Carbohydrate digestion and absorption|Starch and sucrose metabolism
25	DN14363_c0_g1_i1_1	Down	--	--	--	
26	DN28583_c0_g1_i1_1	Down	--	--	--	
27	DN28609_c0_g1_i1_1	Down	--	--	--	
28	DN20507_c0_g1_i1_1	Down	ICK	intestinal cell (MAK-like) kinase	--	
29	DN29071_c	Down	amyA	alpha-amylase	ko04973|	Carbohydrate digestion and

	0_g1_i1_1				ko00500	absorption|Starch and sucrose metabolism
30	DN15849_c0_g4_i1_1	Down	ATP2B	Ca2+ transporting ATPase, plasma membrane	ko04024|ko04020	cAMP signaling pathway|Calcium signaling pathway

Table S4. Primer sequences of real-time PCR for validation of the lncRNAs by quantitative RT-PCR anaysis.

**LncRNA Primer ID**	**Sequence (5' to 3')**
GADPH F	ctg gaa gag ctg cag gat gc
GADPH R	agt ggg aac tct gaa ggc ca
DN31340_c0_g1_i1_2 F	aac tcc aag tat ggt gcc agg aag
DN31340_c0_g1_i1_2 R	gga aga ggt tca gct cca aca cc
DN27831_c0_g1_i1_2 F	atg gtc gag ggt cct gca tt
DN27831_c0_g1_i1_2 R	cag cag gca ctg ggt aag tg
DN17472_c0_g2_i1_2 F	gct tgc tga gat gcc agg ac
DN17472_c0_g2_i1_2 R	tct ggg tct ccc aat ctg gtg
DN17958_c0_g1_i1_2 F	tct aca agg tac tag agt cgg ca
DN17958_c0_g1_i1_2 R	gtt gat gca aga ggt ttg cct t
DN15482_c0_g3_i1_2 F	tcc tga tgg aaa gcc tca agt c
DN15482_c0_g3_i1_2 R	ccc aca tct tct tca cct gac a
DN27504_c0_g1_i1_2 F	tgg gga tgc agg aaa tgt agc
DN27504_c0_g1_i1_2 R	tga aag gct ttg agt ggg ca

DN4876_c0_g1_i1_2 F	gcg gtg aag ctg ttc aaa atg t
DN4876_c0_g1_i1_2 R	agc gat gtt ggt gcc ttg ag
DN18905_c0_g1_i1_2 F	gca gga gct att gca gga gt
DN18905_c0_g1_i1_2 R	agc ccg ttg tca gta aag ca
DN10339_c0_g1_i2_1 F	cac gtc aca aga aca tcc acc t
DN10339_c0_g1_i2_1 R	agc gaa ggc tct acc aca ct
DN10845_c0_g1_i1_1 F	caa caa ggt cgc caa cac ct
DN10845_c0_g1_i1_1 R	ggg gca tag gca ggg tct aa
DN15145_c0_g1_i1_2 F	cga agg aag tta agg aga agg agc
DN15145_c0_g1_i1_2 R	agc ttc aca tcg tct tct gcc
DN11559_c0_g1_i1_1 F	tgg cta tgc tgt tgc tga tcc
DN11559_c0_g1_i1_1 R	gcc aac aca ggc aat aag cg
DN5033_c0_g1_i1_1 F	gct gaa act gtt gga taa gaa atg c
DN5033_c0_g1_i1_1 R	gct gag gat tgc tac ata ctt tgc
DN22701_c0_g1_i1_2 F	tgt cag cac atc agg agg ata c
DN22701_c0_g1_i1_2 R	agg gtg gca gga ttt tca gt
DN9038_c0_g1_i1_1 F	agc tgc acc tac aag cac tg
DN9038_c0_g1_i1_1 R	agg agc ttg tct tgg gtc ata ga
DN8645_c0_g1_i1_2 F	agg tgg aaa gag agc acc ca
DN8645_c0_g1_i1_2 R	gga agg ttt ctc act tct tgg ca
